# Nucleophile-induced ring contraction in pyrrolo[2,1-*c*][1,4]benzothiazines: access to pyrrolo[2,1-*b*][1,3]benzothiazoles

**DOI:** 10.3762/bjoc.19.46

**Published:** 2023-05-11

**Authors:** Ekaterina A Lystsova, Maksim V Dmitriev, Andrey N Maslivets, Ekaterina E Khramtsova

**Affiliations:** 1 Department of Chemistry, Perm State University, ul. Bukireva 15, Perm 614990, Russian Federation

**Keywords:** 1,4-benzothiazine, 1,3-benzothiazole, 1*H*-pyrrole-2,3-diones, nitrogen heterocycle, sulfur heterocycle

## Abstract

Pyrrolo[2,1-*b*][1,3]benzothiazoles are an important class of fused sulfur and nitrogen-containing heterocycles intensively studied in medicinal chemistry and pharmacology. In the present paper, a new synthetic approach to pyrrolobenzothiazoles is developed based on 1,4-thiazine ring contraction in 3-aroylpyrrolo[2,1-*c*][1,4]benzothiazine-1,2,4-triones under the action of nucleophiles. The proposed approach works well with alkanols, benzylamine, and arylamines. The scope and limitations of the developed approach are studied. The synthesized pyrrolobenzothiazole derivatives represent an interest to pharmaceutics, since their close analogs show CENP-E inhibitory activity, interesting for the targeted cancer therapy development.

## Introduction

**Figure 1:**
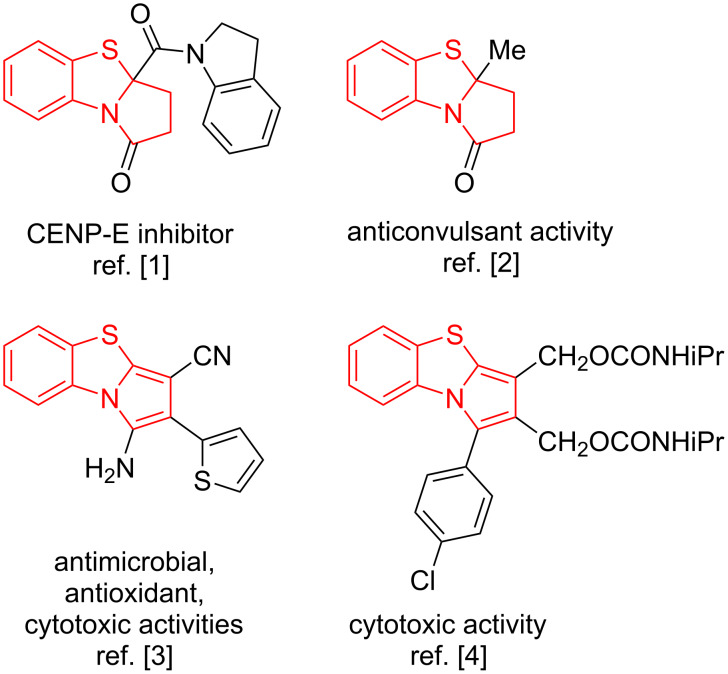
Biologically active PBTAs.

Pyrrolo[2,1-*b*][1,3]benzothiazole (PBTA) is an angularly fused sulfur and nitrogen-containing heterocyclic scaffold. Its derivatives are popular in medicinal chemistry and pharmacology as potential biologically active compounds. In particular, PBTAs were found to be promising inhibitors of centromere-associated protein E (CENP-E) (Figure 1), which is demanded for the development of targeted cancer therapy [1]. Furthermore, candidate anticonvulsant agents had been developed based on PBTA derivatives (Figure 1) [2]. In addition, series of PBTAs (Figure 1) were found to exhibit antibacterial, antifungal, antioxidant, and cytotoxic activities [3–4].

Several strategies have been developed for the synthesis of PBTA derivatives [2–32] to meet the needs of medicinal chemistry and pharmacology for PBTAs containing diverse substituents. In general, these synthetic strategies can be divided into four groups.

**Scheme 1:**
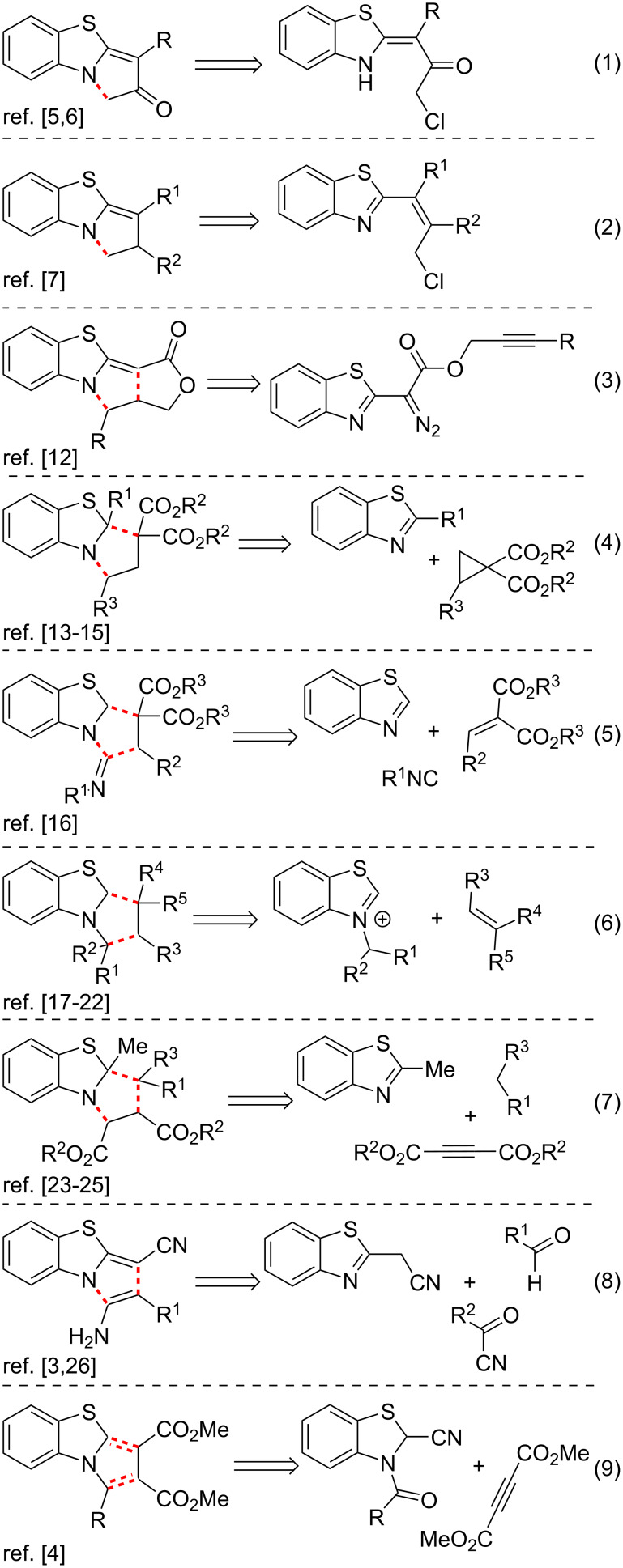
Approaches to PBTAs via annulation of benzothiazoles.

The first group of approaches to the PBTA scaffold is an annulation of benzothiazoles with a pyrrole moiety (Scheme 1). It includes intramolecular cyclizations of benzothiazoles bearing a 3'-chloro substituent at C*^2^* position (Scheme 1, entries 1 and 2) [5–7], intramolecular catalytic carbene cascade reactions of propargyl 1,3-benzothiazol-2-yl(diazo)acetates (Scheme 1, entry 3) [12], dearomative [3 + 2] cycloaddition reactions of benzothiazoles with cyclopropanes (Scheme 1, entry 4) [13–15], multicomponent reactions (MCRs) of benzothiazoles, isocyanides and 2-methylidenemalonates (Scheme 1, entry 5) [16], 1,3-dipolar cycloadditions of *N*-alkylbenzothiazolium salts (Scheme 1, entry 6) [17–22], MCRs of 2-methylbenzothiazole, acetylenedicarboxylates and active methylene compounds (Scheme 1, entry 7) [23–25], MCRs of (1,3-benzothiazol-2-yl)acetonitrile, aldehydes and acylcyanides (Scheme 1, enry 8) [3,26] and reactions of 3-acyl-2,3-dihydro-1,3-benzothiazole-2-carbonitriles with acetylenedicarboxylate (Scheme 1, entry 9) [4].

**Scheme 2:**
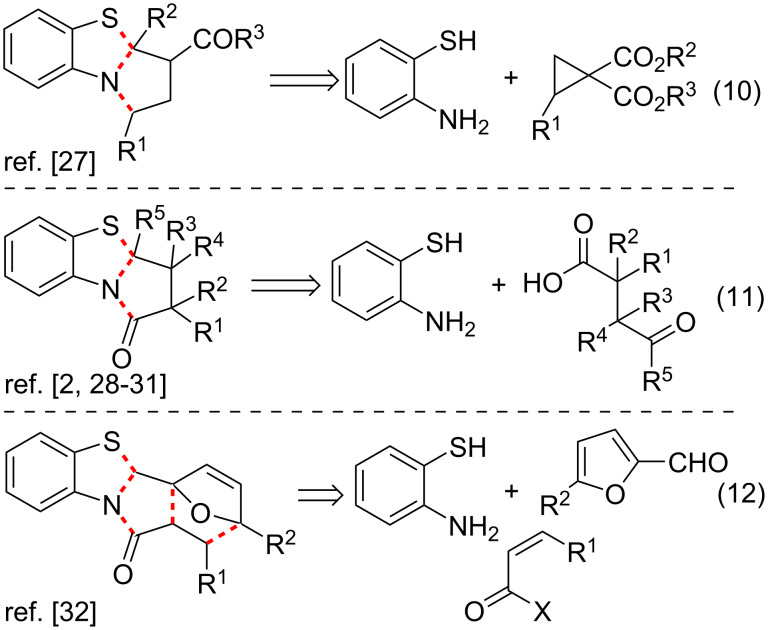
Approaches to PBTAs via annulation of *o*-aminothiophenols.

The second group of approaches to the PBTA scaffold is an annulation of *o*-aminothiophenol with a pyrrolothiazole moiety (Scheme 2). It includes catalytic cascade reactions of *o*-aminothiophenol with donor–acceptor cyclopropanes (Scheme 2, entry 10) [27], condensations of *o*-aminothiophenol with 4-oxo acids or their derivatives (Scheme 2, entry 11) [2,28–31] and cascade reactions of *o*-aminothiophenol, furfural and anhydrides of 2,3-unsaturated carboxylic acids (Scheme 2, entry 12) [32].

**Scheme 3:**
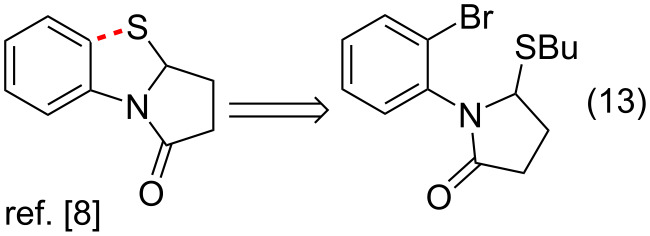
Approach to PBTAs via radical substitution reaction in 1-(2-bromophenyl)-5-(butylsulfanyl)pyrrolidin-2-one.

The third group of approaches to the PBTA scaffold includes only one example, the intramolecular radical substitution reaction in 1-(2-bromophenyl)-5-(butylsulfanyl)pyrrolidin-2-one (Scheme 3, entry 13) [8].

**Scheme 4:**
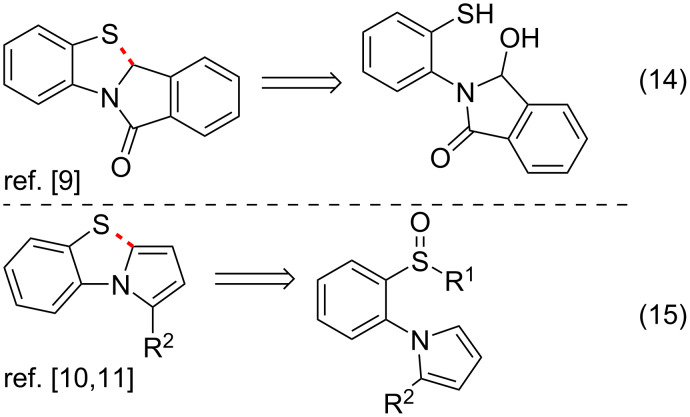
Approach to PBTAs via intramolecular cyclizations of 1-(2-thiophenyl)pyrroles.

The fourth group of approaches to the PBTA scaffold is the intramolecular cyclization of 1-(2-thiophenyl)pyrroles (Scheme 4). It includes intramolecular cationic π-cyclizations in 3-hydroxy-2-(2-sulfanylphenyl)-2,3-dihydro-1*H*-isoindol-1-ones (Scheme 4, entry 14) [9] and intramolecular cyclizations of 1-(2-(methylsulfinyl)phenyl)-1*H*-pyrroles under «interrupted Pummerer rearrangement» conditions (Scheme 4, entry 15) [10–11].

**Scheme 5:**
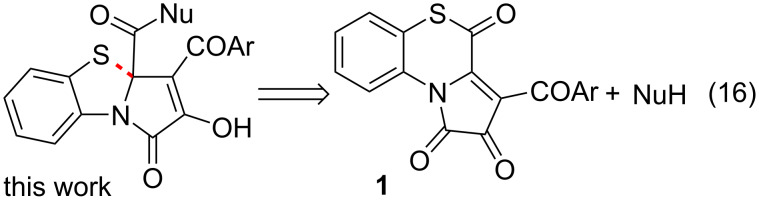
A new approach to PBTAs via nucleophile-induced ring contraction in pyrrolo[2,1-*c*][1,4]benzothiazines.

This work reports a new approach to PBTA derivatives via nucleophile-induced ring contraction in pyrrolo[2,1-*c*][1,4]benzothiazines **1** (Scheme 5, entry 16), which can generally be attributed as a new entry to the fourth group of approaches to the PBTA scaffold (Scheme 4).

## Results and Discussion

**Figure 2:**
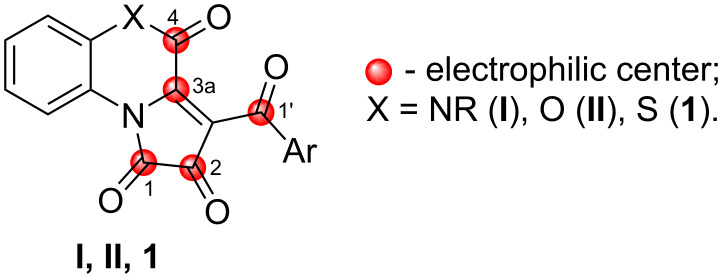
Electrophilic centers in FPDs.

It is known that [*e*]-fused 1*H*-pyrrole-2,3-diones (FPDs) (Figure 2) are versatile synthetic platforms enabling the synthesis of numerous heterocyclic species [33–36]. They are polyelectrophilic compounds, bearing five electrophilic centers, whose reactivity dramatically depends on the nature of the heteroatom X in FPDs **I**, **II**, **1** [33–34].

It should be noted that the reactions of 5-aza- and 5-oxa-FPDs **I** and **II** with nucleophiles are studied rather well [33–34], and their reactivity did not give us any insights for the development of new approaches to PBTAs. However, recently, we have reported a new class of FPDs, aroylpyrrolobenzothiazinetriones (APBTTs) **1** (Figure 2) [37–38], whose structural features allowed us to assume a possibility of the development of a new approach to PBTAs via a nucleophile-induced ring contraction in the 1,4-benzothiazine moiety of compounds **1** (Scheme 5). Firstly, FPDs **1** bear a 1,4-benzothiazine moiety that is known to be prone to undergo a ring contraction reaction to afford the corresponding 1,3-benzothiazole derivatives under the action of nucleophiles [39–42], oxidizing agents [43–48] or ultraviolet irradiation [49]. Secondly, the presence of a highly reactive thioester group C^4^=O [50] in FPDs **1** made us to expect the position C^4^ (Figure 2) to be the most reactive electrophilic center in these molecules, which would also contribute to the development of a new synthetic approach to PBTAs.

**Scheme 6:**
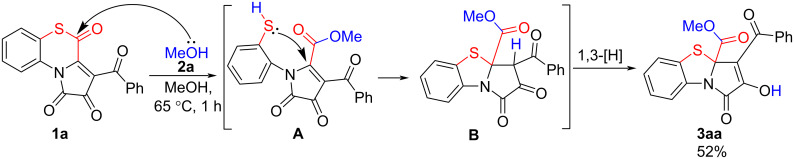
Reaction of APBTT **1a** with methanol (**2a**).

We started our research by conducting a test reaction of APBTT **1a** with anhydrous methanol **2a** (Scheme 6). As a result, we obtained the expected PBTA **3aa** in a good isolated yield (52%). The product **3aa** was isolated by simple recrystallization of the evaporated reaction mixture.

Apparently, the reaction proceeded according to the plausible pathway shown in Scheme 6. As we expected, the nucleophile **2a** attacked on the position C^4^ of the substrate **1a**, which resulted in the cleavage of the S^5^–C^4^ bond and the formation of a thiol intermediate **A** (1-(2-thiophenyl)pyrrole derivative generated in situ as a precursor analog for approaches from Scheme 4). Then, intermediate **A** underwent an intramolecular cyclization by the attack of the SH group on the C^5^ atom of the pyrrole-2,3-dione moiety to afford intermediate **B** that underwent a 1,3-prototropic shift to give product **3aa**.

Next, the conditions (Table 1) of the model reaction of APBTT **1a** with methanol (**2a**) were optimized. The best yield of PBTA **3aa** was observed when methanol was used both as a solvent and a reagent and heated at 65 °C for 1 h (entry 7, Table 1). It is useful to note that, under these conditions, an increase in the heating time (up to 6 h) did not affect the HPLC-UV yield of compound **3aa**. These conditions were taken as a standard for further reactions.

**Table 1:** Reaction of APBTT **1a** with methanol (**2a**) in different solvents.^a^

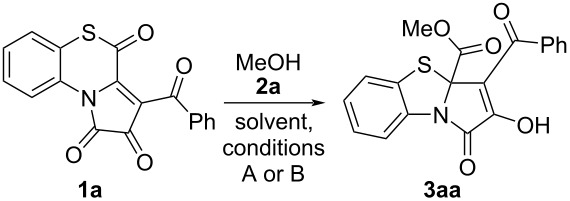			

Entry	Solvent	Conditions	

		A^b^	B^c^
		Yield,^d^ %	

1	acetone	13	17
2	acetonitrile	38	20
3	butyl acetate	16	29
4	chloroform	27	29
5	1,4-dioxane	33	29
6	hexane	35^e^	39^f^
7	methanol	64	81
8	toluene	23	10

^a^Reaction scale: a mixture of **1a** (10 mg, 29.8 µmol), solvent (500 µL), and **2a** (1.2 µL, 29.8 µmol) was stirred in an oven-dried closed microreaction V-vial. ^b^Conditions A: room temperature, 24 h. ^c^Conditions B: heating at the boiling point temperature of the solvent, 1 h. ^d^HPLC–UV yields (biphenyl was used as an internal standard; each entry was carried out in triplicate, and the yields are given as mean values). ^e^Reaction was monitored for 14 days. ^f^Reaction time was 5 h.

**Scheme 7:**
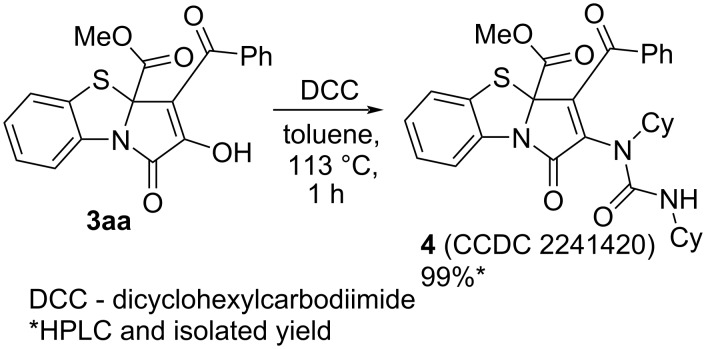
Derivatization of PBTA **3aa**.

Noteworthy, we had to derivatize product **3aa** to detect it by HPLC–UV during the optimization studies. For these purposes, compound **3aa** was converted to compound **4** by an earlier procedure developed by us (Scheme 7) [51]. Without this derivatization procedure, we could not accurately detect compound **3aa** by HPLC–UV, since the chromatographic signals of untreated compound **3aa** were broad and blurry.

**Scheme 8:**
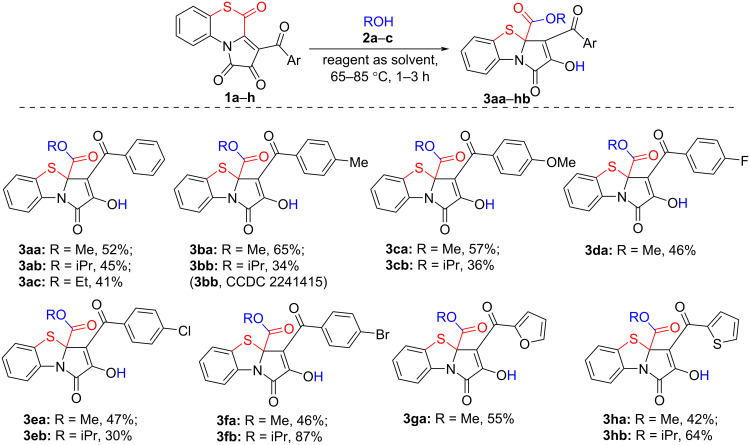
Reaction of APBTTs **1a–h** with alcohols **2a–c**. Isolated yields are given; reaction scale: a mixture of **1** (0.45 mmol) and alcohol **2** (5 mL) was stirred in an oven-dried closed microreaction V-vial at the boiling point temperature of the used alcohol **2**.

Next, the reactant scope of the reaction was explored by involving to the reaction APBTTs **1a**–**h**, bearing various aroyl substituents, and anhydrous alcohols **2a–c** (Scheme 8) [52].

As a result, we found that the proposed procedure afforded target products **3** in poor to very good isolated yields (Scheme 8). We also observed that the nature of the aroyl substituents in substrates **1a–h** did not significantly affect the yields of the corresponding products **3** and the general course of the reaction. However, the structure of the alcohols **2a–c** had an effect on the studied reaction. Reactions with isopropyl alcohol **2b** required longer reaction times (UPLC–UV–MS monitoring). This phenomenon could be due to the steric factors brought in by a bulky isopropyl substituent in alcohol **2b**.

**Scheme 9:**
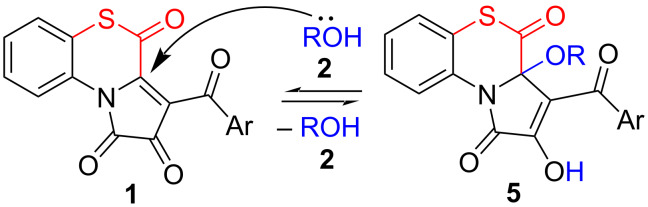
Side-reaction of APBTTs **1** with alcohols **2**.

In addition, in all studied cases we observed that the reaction of APBTTs **1** with alcohols **2** always afforded labile side-products **5** (Scheme 9). Compounds **5** were formed when the nucleophile **2** attacked on the position C^3a^ of the substrates **1**. Such a direction of nucleophilic addition of alcohols to FPD species was observed earlier on the example of 5-oxa-FPDs **II** and was found to be reversible [33,53–54].

**Scheme 10:**
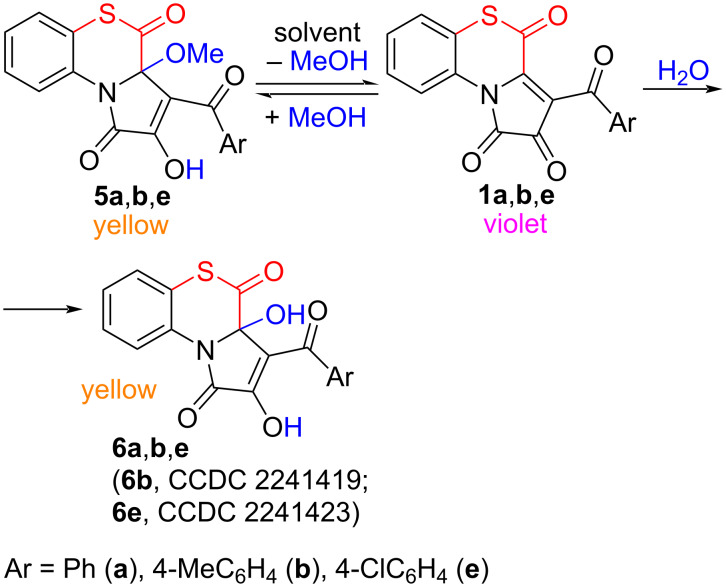
Transformations of compounds **5** in solutions.

We isolated products **5a**,**b**,**e** to study their chemical behavior in solutions. We found that when compounds **5a**,**b**,**e** were dissolved in anhydrous solvents (toluene, acetonitrile, DMSO-*d*_6_) at room temperature, or when these solutions were slightly heated, the compounds **5a**,**b**,**e** dissociated to form APBTTs **1** (the solutions got violet color, characteristic of compounds **1**) (Scheme 10). In the presence of water (including the atmospheric moisture), hydration products **6a**,**b**,**e** were formed (Scheme 10). These observations are in a full accordance with the studies of similar products of 5-oxa-FPDs **II** [33,53–54].

We assume compounds **5** to be products of the kinetic control of the reaction, and compounds **3**, of the thermodynamic one. In addition, the formation of compounds **5** is reversible, and the formation of compounds **3** is irreversible. These assumptions were proved experimentally. In the study of the reaction of APBTT **1a** with methanol (**2a**) by UPLC–UV–MS, we found that in 5 min at room temperature, the reaction mixture contained about 90% of product **5a**, and in 1 h of heating the reaction mixture at 65 °C, it contained trace amounts of product **5a** and 81 % of product **3aa**.

Additionally, we have examined the scaling of the reaction of APBTT **1a** with methanol (**2a**). We found that the proposed procedure could be readily scaled up to 1.5 mmol (0.5 g) of APBTT **1a**. The isolated yield of compound **3aa** was 50%. However, under such conditions, a longer reaction time was required (about 3 h, UPLC–UV–MS monitoring).

Then, to expand the scope of the developed approach to PBTAs, we examined several more groups of nucleophilic reagents.

**Scheme 11:**
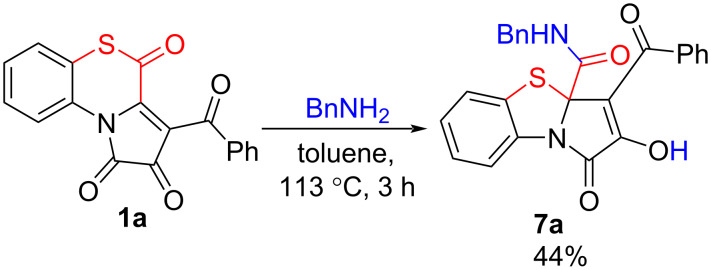
Reaction of APBTT **1a** with benzylamine.

For these purposes, we carried out a test reaction of APBTT **1a** with benzylamine (Scheme 11). As we expected, this reaction proceeded similarly to the reaction of APBTTs **1** with alcohols **2**, and we obtained the desired PBTA **7a** in a good isolated yield (44%). The product **7a** was isolated by simple crystallization from the reaction mixture.

Next, the conditions (Table 2) of the model reaction of APBTT **1a** and benzylamine were optimized. The best yield of PBTA **7a** was observed when acetonitrile was used as the solvent and heated at 85 °C for 3 h (entry 2, Table 2). Since the product **7a** isolation procedure proceeded more conveniently in toluene (the product could be isolated by simple filtration directly from the reaction mixture), and the yield of PBTA **7a** was satisfactory, we chose these conditions (entry 7, Table 2) as a standard for further reactions.

**Table 2:** Reaction of APBTT **1a** with benzylamine in different solvents.^a^

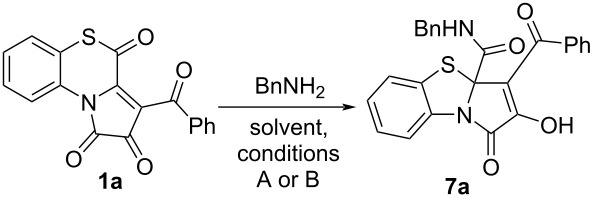			

Entry	Solvent	Conditions	

		A^b^	B^c^
		Yield,^d^ %	

1	acetone	31	35
2	acetonitrile	69	87
3	butyl acetate	45	61
4	chloroform	61	68
5	1,4-dioxane	32	53
6	hexane	traces^e^	traces^f^
7	toluene	40	50

^a^Reaction scale: a mixture of **1a** (10 mg, 29.8 µmol), solvent (500 µL), and benzylamine (3.3 µL, 29.8 µmol) was stirred in an oven-dried closed microreaction V-vial. ^b^Conditions A: room temperature, 24 h. ^c^Conditions B: heating at the boiling point temperature of the solvent, 3 h. ^d^HPLC–UV yields (biphenyl was used as an internal standard; each entry was carried out in triplicate, and the yields are given as mean values). ^e^Reaction was monitored for 10 days. ^f^Reaction time was 8 h.

**Scheme 12:**
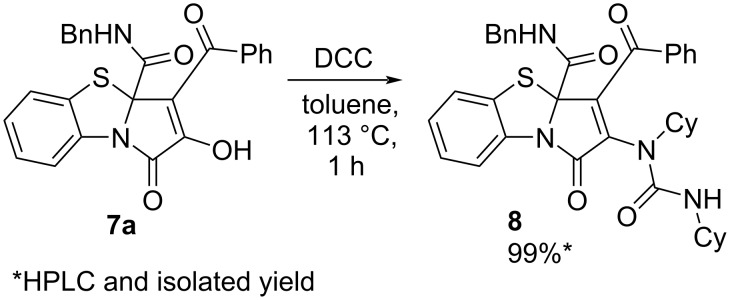
Derivatization of PBTA **7a**.

As in the case of the above studied reaction with alcohols (Scheme 7), we had to derivatize product **7a** (Scheme 12) by an earlier procedure developed by us [51] in order to investigate the reaction optimization by HPLC–UV.

**Scheme 13:**
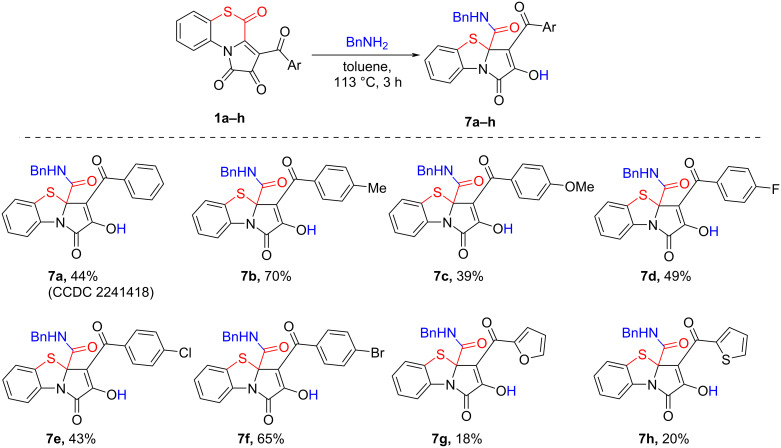
Reaction of APBTTs **1a–h** and benzylamine. Isolated yields are given; reaction scale: a mixture of **1** (0.45 mmol), benzylamine (0.49 mmol, 54 µL) and anhydrous toluene (3 mL) was stirred in an oven-dried closed microreaction V-vial.

Then, the reactant scope of the reaction was explored by involving to the reaction APBTTs **1a–h**, bearing various aroyl substituents, and benzylamine (Scheme 13) [55].

As a result, we found that the proposed procedure afforded target products **7** in poor to good isolated yields (Scheme 13). We also observed that the nature of the aroyl substituents in substrates **1a–h** did not significantly affect the yields of the corresponding products **7** and the general course of the reaction.

**Scheme 14:**
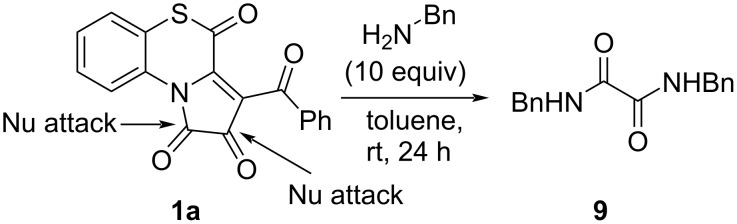
Reaction of APBTT **1a** with an excess of benzylamine.

We also examined the influence of an excess of benzylamine on the yields of PBTAs **7**. In the reaction of APBTT **1a** with benzylamine in a ratio of 1:2 (reaction conditions were the same as in Scheme 13), the isolated yield of the product **7a** was lower (35%) than in the case of the reaction in a ratio of 1:1.1 (Scheme 13). However, when conducting the reaction in a ratio of 1:10 at room temperature during 24 h, we observed the formation of *N*^1^,*N*^2^-dibenzyloxalamide (**9**) as a major product (NMR yield of about 90%) (Scheme 14).

**Scheme 15:**
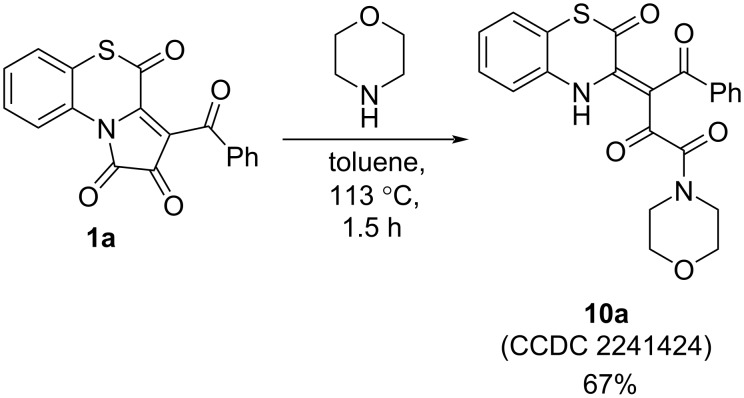
Reaction of APBTT **1a** with morpholine.

Our attempts to employ other alkylamines (diethylamine, morpholine, and cyclohexylamine) to the proposed approach to PBTAs were not successful. In the reaction of APBTT **1a** with diethylamine (**1a**/diethylamine ratio of 1:1; stirring in toluene at 90 °С for 2 h; at 113 °С for 2 h; at room temperature for 24 h) and cyclohexylamine (**1a**/cyclohexylamine ratio of 1:1 or 1:5; stirring in toluene at room temperature for 24 h), a mixture of unidentified substances was formed. Interestingly, in the reaction of APBTT **1a** with morpholine, we succeeded to isolate the product **10a** (Scheme 15) [56]. Product **10a** was formed in a result of a nucleophilic attack of morpholine on the C^1^ position of APBTT **1a**.

Such a change in the reaction selectivity could be explained by the influence of a higher nucleophilicity of the examined alkylamines in comparison with benzylamine.

Then, we tried to involve less nucleophilic amines to the proposed approach.

**Scheme 16:**
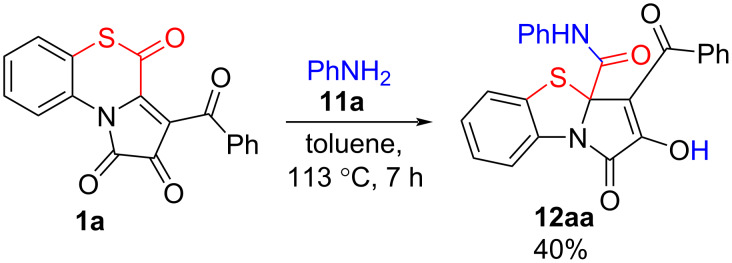
Reaction of APBTT **1a** with aniline (**11a**).

For these, we examined a reaction of APBTT **1a** with aniline (**11a**, Scheme 16). This reaction proceeded similarly to reactions of APBTTs **1** with alcohols **2** and benzylamine, and we obtained the desired PBTA **12aa** in a moderate isolated yield (40%). The product **12aa** was isolated by simple crystallization from the reaction mixture.

Next, the conditions (Table 3) of the model reaction of APBTT **1a** and aniline (**11a**) were optimized. The best yield of PBTA **12aa** was observed when butyl acetate was used as the solvent and heated at 130 °C for 3 h (entry 3, Table 3). Heating the reaction mixture in toluene (entry 7, Table 3) showed a good yield too. Since the product **12aa** isolation procedure proceeded more conveniently in toluene, we chose these conditions (entry 7, Table 3) as a standard for further reactions.

**Table 3:** Reaction of APBTT **1a** with aniline (**11a**) in different solvents.^a^

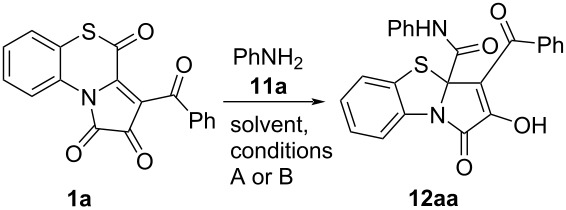			

Entry	Solvent	Conditions	

		A^b^	B^c^
		Yield,^d^ %	

1	acetone	40	0
2	acetonitrile	37	37
3	butyl acetate	34	83
4	chloroform	44	35
5	1,4-dioxane	44	47
6	hexane	40^e^	32^f^
7	toluene	36	73

^a^Reaction scale: a mixture of **1a** (10 mg, 29.8 µmol), solvent (500 µL), and **11a** (2.7 µL, 29.8 µmol) was stirred in an oven-dried closed microreaction V-vial. ^b^Conditions A: room temperature, 24 h. ^c^Conditions B: heating at the boiling point temperature of the solvent, 7 h. ^d^HPLC–UV yields. ^e^Reaction was monitored for 14 days. ^f^Reaction time was 6 h.

**Scheme 17:**
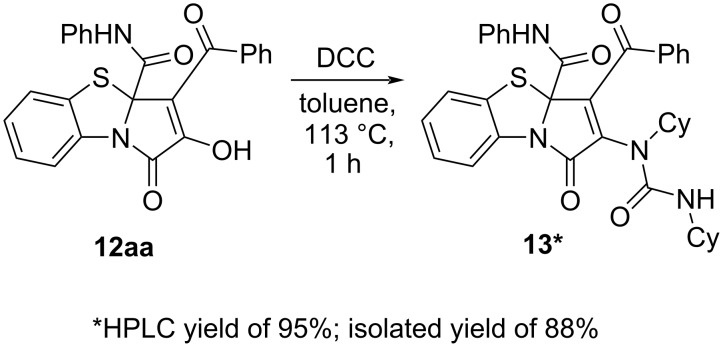
Derivatization of PBTA **12aa**.

As in the cases of the above studied reactions (Scheme 7, Scheme 12), we had to derivatize the product **12aa** (Scheme 17) by an earlier procedure developed by us [51] in order to investigate the reaction optimization by HPLC-UV.

**Scheme 18:**
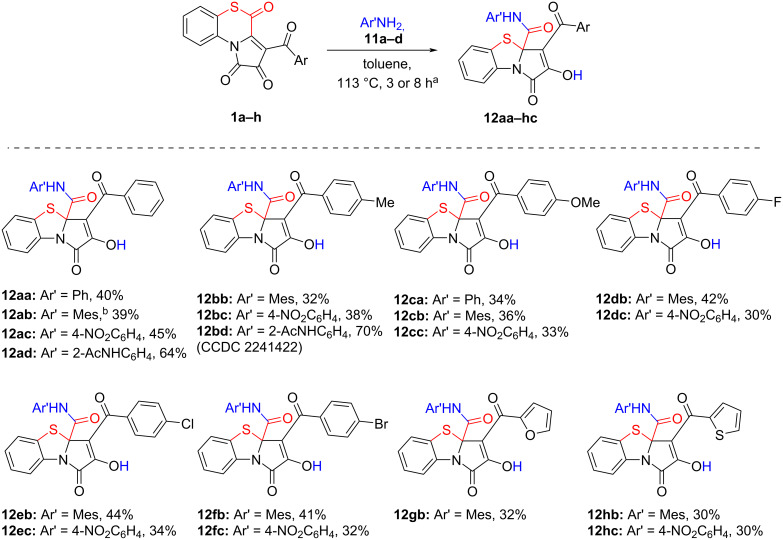
Reaction of APBTTs **1a–h** and arylamines **11a–d**. Isolated yields are given; reaction scale: a mixture of **1** (0.45 mmol), arylamine (0.45 mmol or 0.49 mmol), and anhydrous toluene (3–4 mL) was stirred in an oven-dried closed microreaction V-vial. ^a^Reaction time was 3 h for **11d** and 8 h for **11a–c**. ^b^Mes = 2,4,6-Me_3_C_6_H_2_.

After that, the reactant scope of the reaction was explored by involving to the reaction APBTTs **1a–h**, bearing various aroyl substituents, and arylamines **11a–d**, bearing aryl substituents with various electronic effects (Scheme 18).

As a result, we have found that the proposed procedure afforded target products **12** in poor to good isolated yields (Scheme 18). We also observed that the nature of the aroyl substituents in substrates **1a–h** and aryl substituents in amines **11a–c** did not significantly affect the yields of the corresponding products **12** and the general course of the reaction. However, the reaction with *o*-aminoacetanilide **11d** required shorter reaction times (visual monitoring of the reaction mixture color change and the precipitate formation) and afforded products **12** with higher isolated yields, which could probably be due to the solubility of the starting amine **11d** and the corresponding products **12**.

**Scheme 19:**
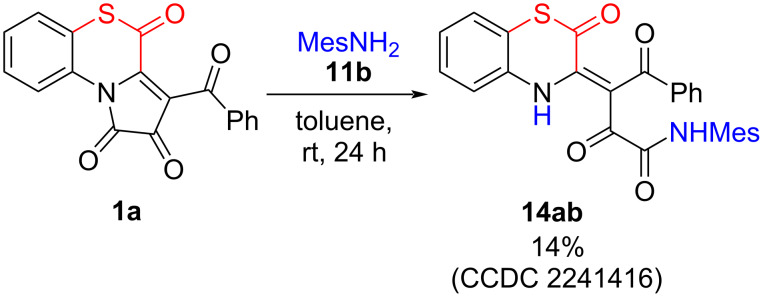
Side-reaction of APBTT **1a** with arylamine **11b**.

In addition, in the reaction of APBTT **1a** with mesidine (**11b**), we succeeded to isolate a side-product **14ab** (Scheme 19). Similar side-products **14** were observed in all reactions of APBTTs **1** with arylamines **11** (UPLC–UV–MS monitoring).

Apparently, the reaction of APBTTs **1** with examined amines (benzylamine, alkylamines, arylamines **11**) proceeded simultaneously in several directions: initial nucleophilic attack on positions C^1^, C^2^ or C^4^ of compounds **1**. The ratio of yields of competitive reaction products depended on the nucleophilicity of the amine.

**Scheme 20:**
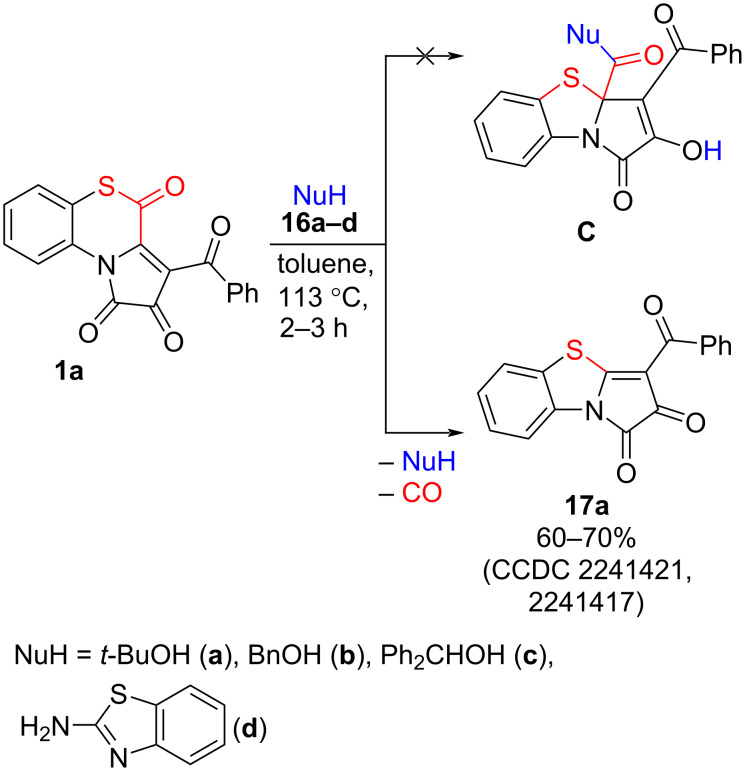
Reaction of APBTT **1a** with compounds **16a–d**.

Then, we examined the proposed approach to PBTAs by involving bulky nucleophilic compounds **16a–d** to the reaction with APBTT **1a**. Unexpectedly, product **17a** was formed instead of the anticipated PBTA **C** (Scheme 20). Compound **17a** was formed in good isolated yields (60–70%) and was isolated by a simple crystallization from the reaction mixture.

**Scheme 21:**
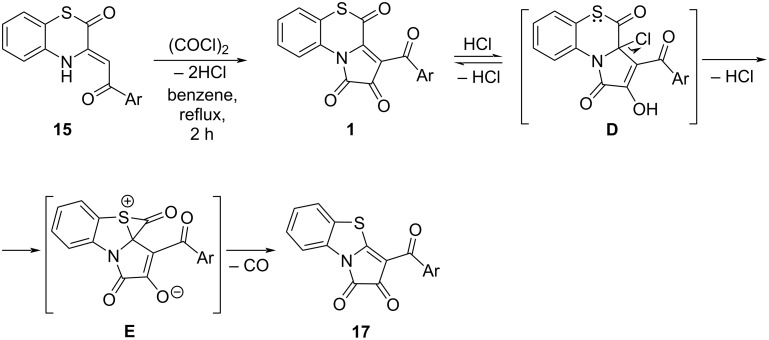
Formation of compounds **17** as an undesired process during the synthesis of APBTTs **1**.

Moreover, we observed the formation of compounds **17** during acylation of enamines **15** with oxalyl chloride to prepare the starting APBTTs **1** (Scheme 21). We noticed that in this case, compounds **17** were formed when HCl was not effectively removed from the reaction mixtures. Bubbling of anhydrous argon through the reaction mixtures facilitated the removal of HCl, and reduced the formation of products **17** to trace amounts. Because of this, we assume that the formation of compounds **17** was caused in the result of addition of HCl to APBTTs **1** to form intermediates **D** [57], which underwent intramolecular nucleophilic substitution of the chloro substituent at C^3a^ position with S^5^ [58] to give intermediates **E**. Then, intermediates **E** readily decarbonylated [59–60] to afford compounds **17** (Scheme 21). We suppose that in the reaction of APBTTs **1** with nucleophiles **16a–d**, the formation of compounds **17** could proceed via a similar pathway, since Nu-groups of compounds **16a–d** are good bulky leaving groups for nucleophilic substitution reactions. Nevertheless, the pathway of formation of compounds **17** is questionable and may become a subject of a new study.

## Conclusion

In conclusion, we have developed a new approach to pyrrolo[2,1-*b*][1,3]benzothiazoles **3**, **7**, and **12** via nucleophilic transformations of 3-aroylpyrrolo[2,1-*c*][1,4]benzothiazine-1,2,4-triones **1**. The studied process presents a nucleophile-induced 1,4-benzothiazine ring contraction in compounds **1** through the cleavage of the S–C bond of the 1,4-benzothiazine moiety under the action of the nucleophile to form in situ a 1-(2-thiophenyl)pyrrole derivative that undergoes an intramolecular cyclization to give the target pyrrolobenzothiazoles **3**, **7**, and **12**. The developed approach works well with alkanols **2**, benzylamine, and arylamines **11**, while alkylamines are unsuitable for it. Notable, the use of bulky nucleophiles (*tert*-butyl alcohol (**16a**), benzyl alcohol (**16b**), benzhydrol (**16c**), 2-aminobenzothiazole (**16d**), HCl) makes it possible to obtain pyrrolobenzothiazoles **17** from compounds **1**, but their formation proceeds through a different pathway from the one to pyrrolobenzothiazoles **3**, **7**, and **12**.

## Supporting Information

File 1 Further experimental details, copies of NMR spectra, X-ray crystallographic details, optimization by HPLC-UV details.

File 2 Crystallographic information files (CIF) of compounds **3bb** (CCDC 2241415), **4** (CCDC 2241420), **6b** (CCDC 2241419), **6e** (CCDC 2241423), **7a** (CCDC 2241418), **10a** (CCDC 2241424), **12bd** (CCDC 2241422), **14ab** (CCDC 2241416), **17a** (CCDC 2241417, 2241421).
