# Supplementary material for: Nucleophile-induced ring contraction in pyrrolo[2,1-c][1,4]benzothiazines: access to pyrrolo[2,1-b][1,3]benzothiazoles
Source: Beilstein J Org Chem. 2023 May 11;19:646–57. doi: 10.3762/bjoc.19.46 (PMC10186265; doi:10.3762/bjoc.19.46)
Supplement: File 1 — Further experimental details, copies of NMR spectra, X-ray crystallographic details, optimization by HPLC-UV details. [file Beilstein_J_Org_Chem-19-646-s001.pdf]

BEILSTEIN JOURNAL OF ORGANIC CHEMISTRY

Supporting Information

for

Nucleophile-induced ring contraction in pyrrolo[2,1-*c*][1,4]benzothiazines: access to pyrrolo[2,1-*b*][1,3]benzothiazoles

Ekaterina A. Lystsova, Maksim V. Dmitriev, Andrey N. Maslivets and Ekaterina E. Khramtsova

Beilstein J. Org. Chem. **2023,** *19,* 646–657. doi:10.3762/bjoc.19.46

Further experimental details, copies of NMR spectra, X-ray crystallographic details, optimization by HPLC-UV details

License and Terms: This is a supporting information file under the terms of the Creative Commons Attribution License (https://creativecommons.org/licenses/by/4.0). Please note that the reuse, redistribution and reproduction in particular requires that the author(s) and source are credited and that individual graphics may be subject to special legal provisions.
The license is subject to the *Beilstein Journal of Organic Chemistry* terms and conditions: (https://www.beilstein-journals.org/bjoc/terms)

Table of contents

General……S3

3-Aroylpyrrolo[2,1-*c*][1,4]benzothiazine-1,2,4-triones (**1c,g,h**). General procedure…………………………………………S4

Alkyl 3-aroyl-2-hydroxy-1-oxopyrrolo[2,1-*b*][1,3]benzothiazole-3a(1*H*)-carboxylates **3**. General procedure……………………S14

3-Aroyl-2-hydroxy-3a-methoxy-1*H*-pyrrolo[2,1-*c*][1,4]benzothiazine-1,4(3a*H*)-diones **5**. General procedure……………………S64

3-Aroyl-2,3a-dihydroxy-1*H*-pyrrolo[2,1-*c*][1,4]benzothiazine-1,4(3a*H*)-diones **6**. General procedure………………………………S74

3-Aroyl-*N*-benzyl-2-hydroxy-1-oxopyrrolo[2,1-*b*][1,3]benzothiazole-3a(1*H*)-carboxamides **7**. General procedure ………………S84

1-(Morpholin-4-yl)-3-(2-oxo-2*H*-1,4-benzothiazin-3(4*H*)-ylidene)-4-phenylbutane-1,2,4-trione (**10a**)……………………………S111

N-Aryl-3-aroyl-2-hydroxy-1-oxopyrrolo[2,1-*b*][1,3]benzothiazole-3a(1*H*)-carboxamides **12**. General procedure……………………S115

N-Mesityl-2,4-dioxo-3-(2-oxo-2*H*-1,4-benzothiazin-3(4*H*)-ylidene)-4-phenylbutanamide (**14ab**)…………………………………S178

3-Benzoylpyrrolo[2,1-*b*][1,3]benzothiazole-1,2-dione **17a**…………………………………………………………………S182

Preparation of samples for HPLC-UV analyses for optimization. General procedure to compounds **4, 8, 13**……………………S186

Crystal structure determination……………………………………………………………………………………S197

References………S215

Experimental

General

^{1}H and ^{13}C NMR spectra were acquired on a Bruker Avance-III spectrometer (400 and 100 MHz, respectively) in $CDCl_3$ or DMSO-d_6 using the HMDS signal (in ^{1}H NMR) or solvent residual signals (in ^{13}C NMR, 77.00 for $CDCl_3$, 39.52 for DMSO-d_6; in ^{1}H NMR, 7.27 for $CDCl_3$, 2.50 for DMSO-d_6) as internal standards. ^{13}C ssNMR spectra were acquired on a Bruker Avance III 400 WB NMR spectrometer (at 100 MHz). IR spectra were recorded on a Perkin–Elmer Spectrum Two spectrometer from mulls in mineral oil or from thin layers obtained from $CHCl_3$ solutions. Melting points were measured on a Mettler Toledo MP70 apparatus. Single crystal X-ray analyses were performed on an Xcalibur Ruby diffractometer. Elemental analyses were carried out on a Vario MICRO Cube analyzer. The reaction conditions were optimized using HPLC–UV [Hitachi Chromaster system; NUCLEODUR C18 Gravity column; grain size of 5 μm; acetonitrile–water as eluents; flow rate of 1.5 mL/min; Hitachi Chromaster 5430 diode array detector (wavelength range of 210–750 nm)] and UPLC–UV–MS [Waters ACQUITY UPLC I-Class system; Acquity UPLC BEH C18 column, grain size of 1.7 μm; acetonitrile–water as eluents; flow rate of 0.6 mL/min; ACQUITY UPLC PDA eλ Detector (wavelength range of 230–780 nm); Xevo TQD mass detector; electrospray ionization; positive and negative ion detection; ion source temperature of 150 °C; capillary voltage of 3500–4000 V; cone voltage of 20–70 V; vaporizer temperature of 200 °C]. Thin-layer chromatography (TLC) was performed on Merck silica gel 60 F_{254} plates using EtOAc/toluene, 1:5 v/v, toluene, EtOAc as eluents. Column chromatography was performed on silica gel (Chrom-lab, KSKG, 63–200 μm).

APBTTs **1a,b,d,e,f** were prepared according to a reported procedure [1] from enaminones **15** [2] and commercially available reagents. APBTTs **1c,g,h** were prepared according to a general procedure described below from enaminones **15** [2] and commercially available reagents. Toluene, benzene, hexane, and 1,4-dioxane were distilled over Na before the use. Butyl acetate, chloroform, and acetone were distilled over P_2O_5 before the use. Alkanols **2** were dried over molecular sieves 4Å before the use. All other solvents and reagents were purchased from commercial vendors and were used as received. All operations with compounds **1** were carried out in oven-dried glassware.

3-Aroylpyrrolo[2,1-*c*][1,4]benzothiazine-1,2,4-triones (1c,g,h). General procedure.

In a round-bottomed double-necked flask with a reflux condenser equipped with a drying tube ($CaCl_2$), the corresponding enaminone **15** (3.6 mmol) and anhydrous benzene (30 mL) are placed. Argon is bubbled through the reaction mixture throughout the reaction. Oxalyl chloride (5.4 mmol, 0.46 mL) is added to the obtained suspension. The mixture is refluxed until HCl evolution stops (about 6 h, indicator paper control). While the reaction mixture has not yet cooled down, the formed dark violet spongy precipitate of APBTT **1** is filtered off, washed with hot (60–65 °C) anhydrous benzene (10–15 mL), dried in a laboratory oven at 90–95 °C for 30 min. The obtained APBTT **1** is pure enough for the subsequent reactions and does not need any additional purification procedures. Compounds **1** should be stored in a desiccator. APBTTs **1** readily undergo to a hydration reaction, which is typical for such compounds [3]. By this reason, and because of poor solubility of APBTTs **1** in $CDCl_3$ and DMSO-d_6, we did not succeed to acquire their ^{13}C NMR spectra in solutions. Because of this, their ^{13}C NMR spectra were acquired as solid-state NMR (ssNMR) spectra, and their ^{1}H NMR spectra were acquired immediately after the dissolution in DMSO-d_6.

3-(4-Methoxybenzoyl)pyrrolo[2,1-*c*][1,4]benzothiazine-1,2,4-trione (1c)
Dark violet solid; yield (1118 mg, 85%); mp 155–157 °C (decomp., benzene).
^{1}H NMR (400 MHz, DMSO-d_6): δ = 8.72 (m, 1H), 8.04 (m, 2H), 7.50 (m, 2H), 7.37 (m, 1H), 7.05 (m, 2H), 3.87 (s, 3H) ppm.
^{13}C ssNMR (100 MHz): δ = 186.7, 179.9, 171.4, 167.6, 165.5, 163.5, 156.7, 149.1, 136.2, 134.1, 132.4, 130.9, 128.6, 125.9, 118.9, 117.4, 112.7, 110.1, 55.8 ppm.
IR (thin layer obtained from $CHCl_3$): 1741, 1703, 1646 cm^{-1}.
EA: Found: C, 62.66; H, 3.15; N, 3.92. Calc. for $C_{19}H_{11}NO_5S$: C, 62.46; H, 3.03; N, 3.83.

3-(4-Fluorobenzoyl)pyrrolo[2,1-*c*][1,4]benzothiazine-1,2,4-trione (1d)
Dark violet solid; yield (992 mg, 78%); mp 174–176 °C (decomp., benzene).
^{1}H NMR (400 MHz, DMSO-d_6): δ = 8.73 (m, 1H), 8.18 (m, 2H), 7.54 (m, 2H), 7.37 (m, 3H) ppm.
^{13}C ssNMR (100 MHz): δ = 187.5, 181.5, 180.7, 169.0, 168.6, 166.2, 156.4, 149.8, 137.3, 135.0, 132.6, 128.1, 125.3, 119.7, 119.2, 116.6, 116.2, 110.1 ppm.
IR (mineral oil): 1773, 1736, 1656 cm^{-1}.
EA: Found: C, 61.32; H, 2.30; N, 4.00. Calc. for $C_{18}H_8FNO_4S$: C, 61.19; H, 2.28; N, 3.96.

3-(Furan-2-carbonyl)pyrrolo[2,1-*c*][1,4]benzothiazine-1,2,4-trione (1g)
Dark violet solid; yield (1030 mg, 88%); mp 190–192 °C (decomp., benzene).
^{1}H NMR (400 MHz, DMSO-d_6): δ = 8.71 (m, 1H), 8.07 (s, 1H), 7.65 (m, 1H), 7.53 (m, 2H), 7.34 (m, 1H), 6.72 (m, 1H) ppm.

^{13}C ssNMR (100 MHz): δ = 182.4, 176.9, 176.1, 154.8, 152.6, 150.7, 132.7, 129.3, 127.8, 126.7, 119.9, 114.8, 107.8 ppm.
IR (mineral oil): 1774, 1727, 1631 cm^{-1}.
EA: Found: C, 59.23; H, 2.33; N, 4.42. Calc. for $C_{16}H_7NO_5S$: C, 59.08; H, 2.17; N, 4.31.

3-(Thiophene-2-carbonyl)pyrrolo[2,1-*c*][1,4]benzothiazine-1,2,4-trione (1h)
Dark violet solid; yield (1204 mg, 98%); mp 197–199 °C (decomp., benzene).
^{1}H NMR (400 MHz, DMSO-d_6): δ = 8.72 (m, 1H), 8.09 (m, 2H), 7.53 (m, 2H), 7.34 (m, 1H), 7.23 (m, 1H) ppm.
^{13}C ssNMR (100 MHz): δ = 181.1, 179.8, 155.3, 148.3, 145.2, 141.1, 140.0, 137.9, 129.4, 128.6, 127.6, 127.2, 120.5, 117.4, 110.6, 99.4 ppm.
IR (mineral oil): 1776, 1735, 1665, 1650 cm^{-1}.
EA: Found: C, 56.53; H, 2.31; N, 4.13. Calc. for $C_{16}H_7NO_4S_2$: C, 56.30; H, 2.07; N, 4.10.

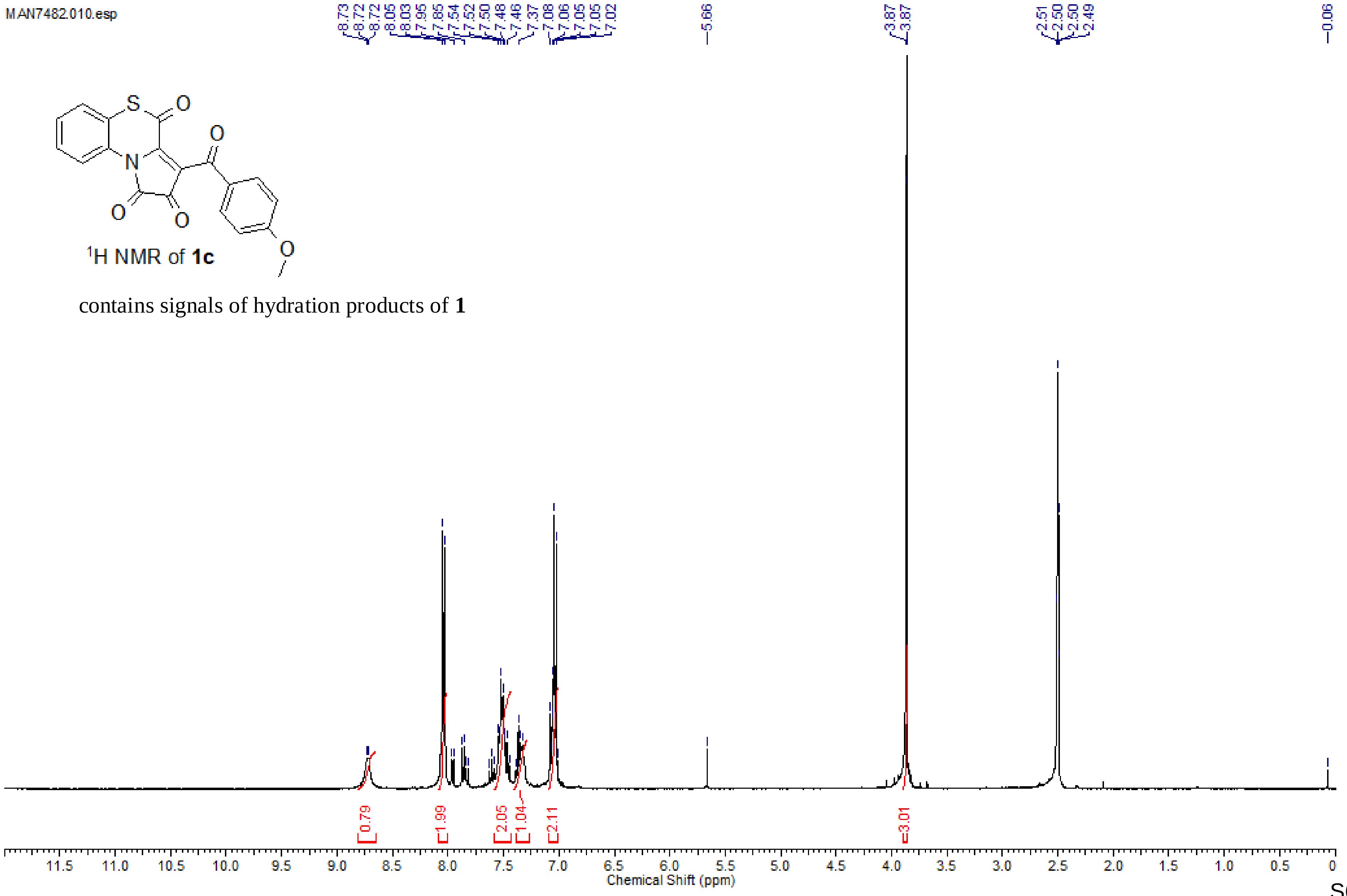

^{1}H NMR of **1c**

contains signals of hydration products of **1**

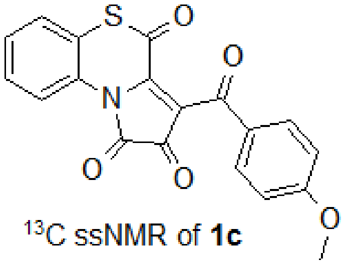

^{13}C ssNMR of **1c**

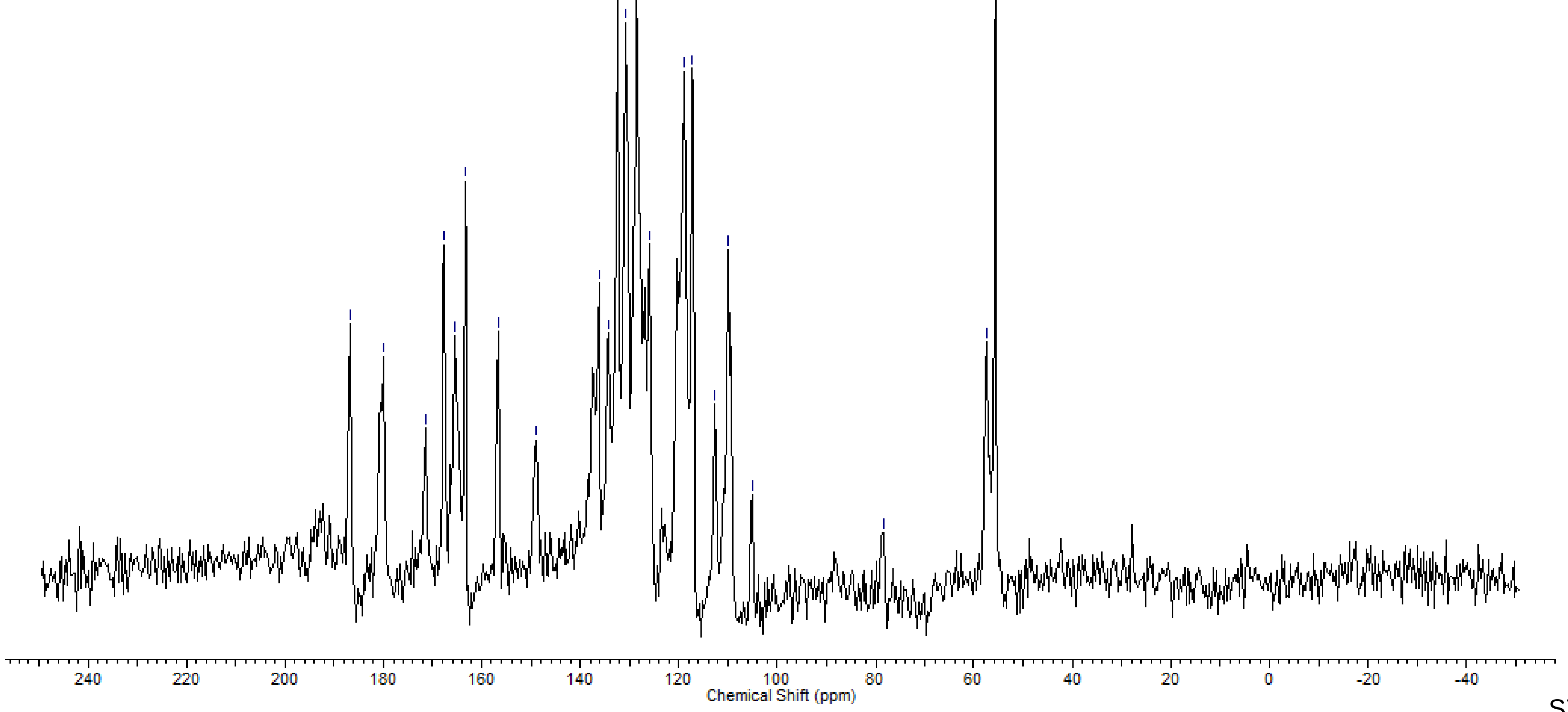

MAN7480.001.esp

^{1}H NMR of **1d**

contains signals of hydration products of **1**

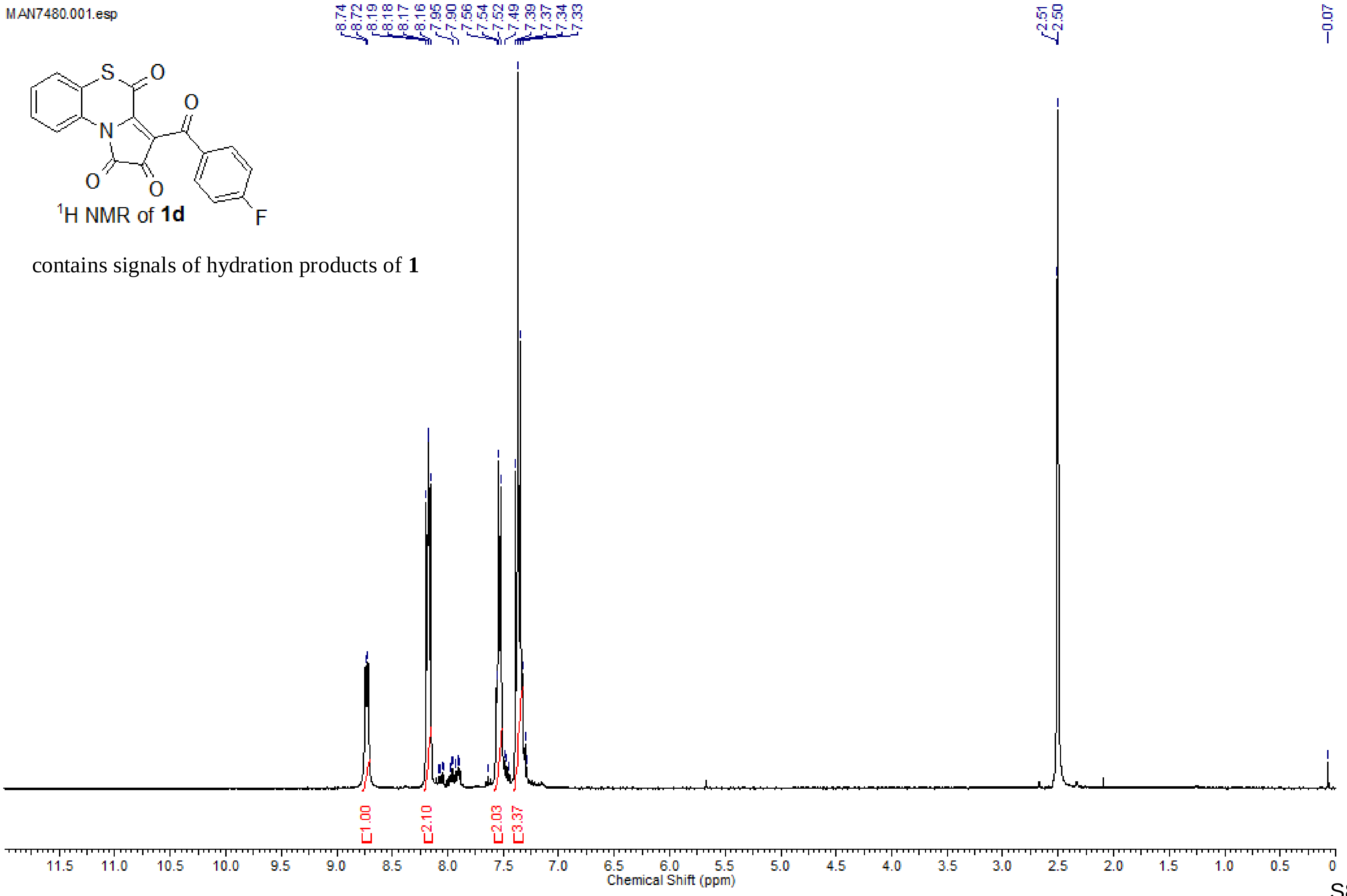

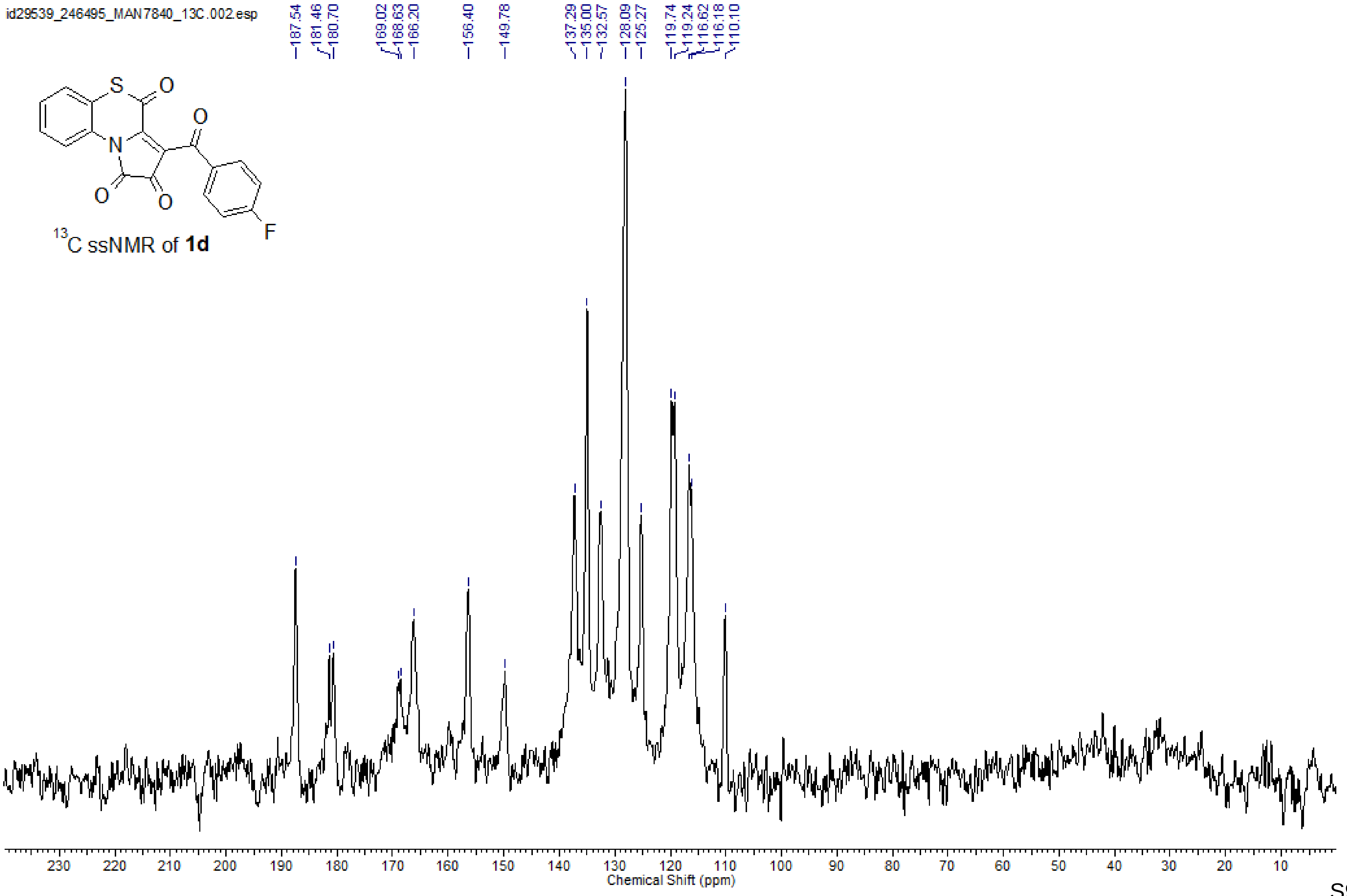
id29539_246495_MAN7840_13C.002.esp
187.54
181.46
180.70
169.02
168.63
166.20
156.40
149.78
137.29
135.00
132.57
128.09
125.27
119.74
119.24
116.62
116.18
110.10
S
O
O
N
O
O
F
13C ssNMR of 1d
230
220
210
200
190
180
170
160
150
140
130
120
110
100
90
80
70
60
50
40
30
20
10
Chemical Shift (ppm)

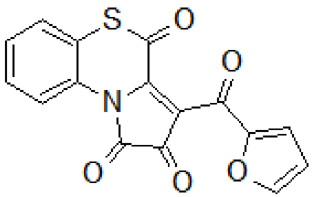

^{1}H NMR of **1g**

contains signals of hydration products of **1**

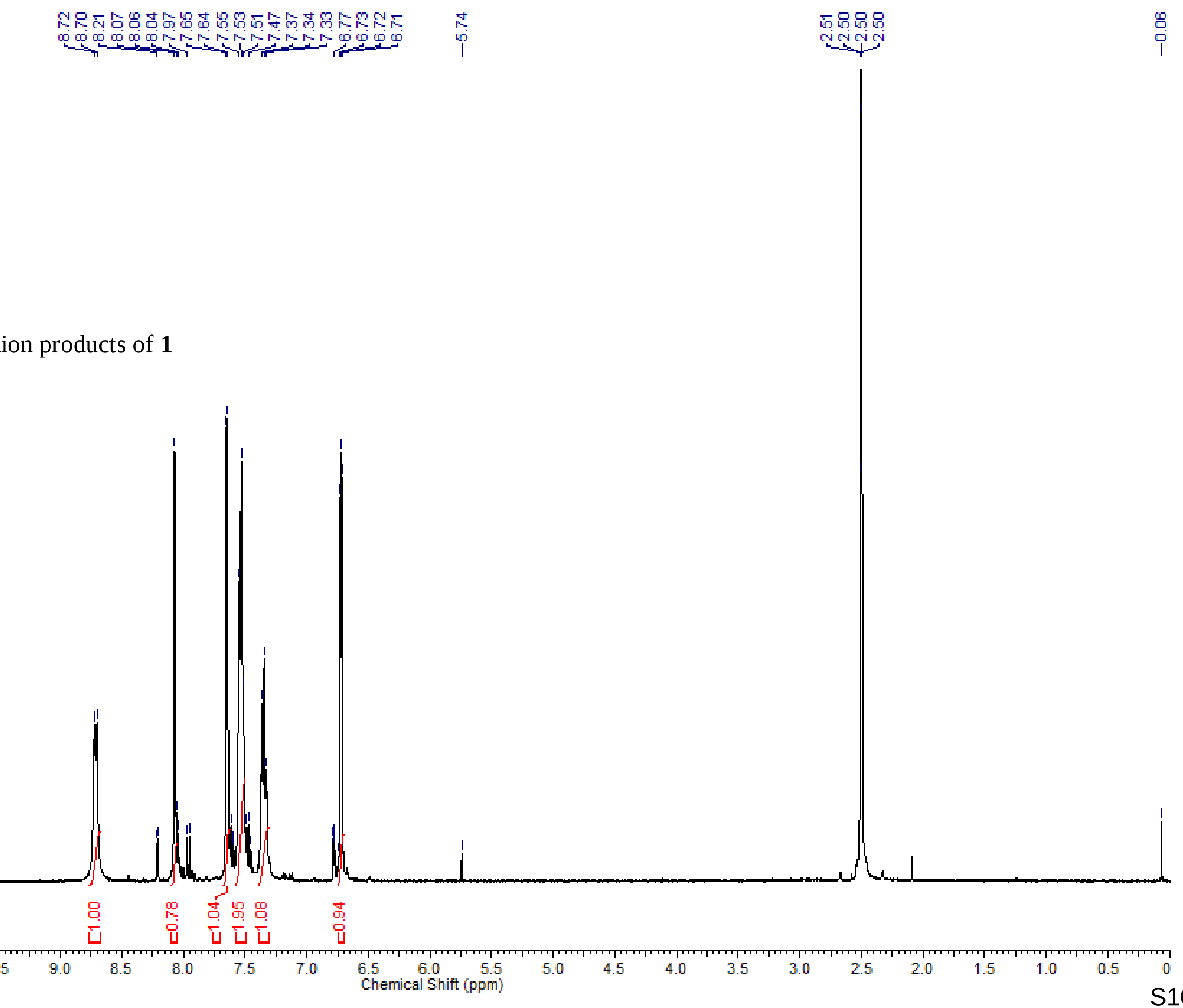

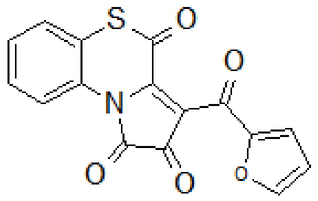

^{13}C ssNMR of **1g**

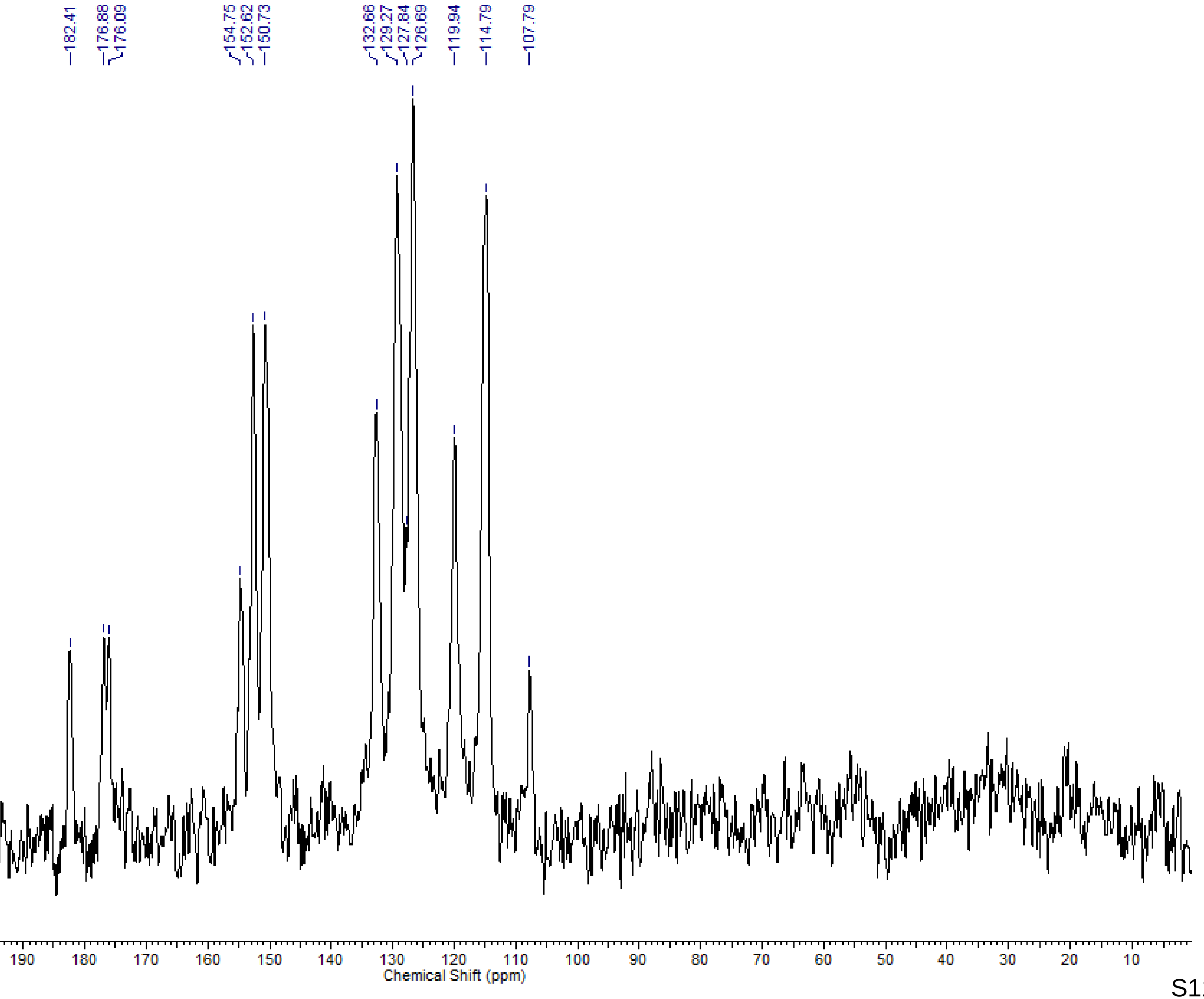

MAN7481.010.esp

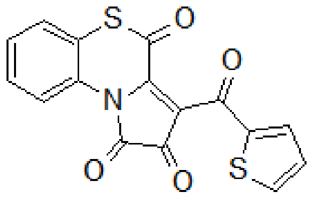

^{1}H NMR of **1h**

contains signals of hydration products of **1**

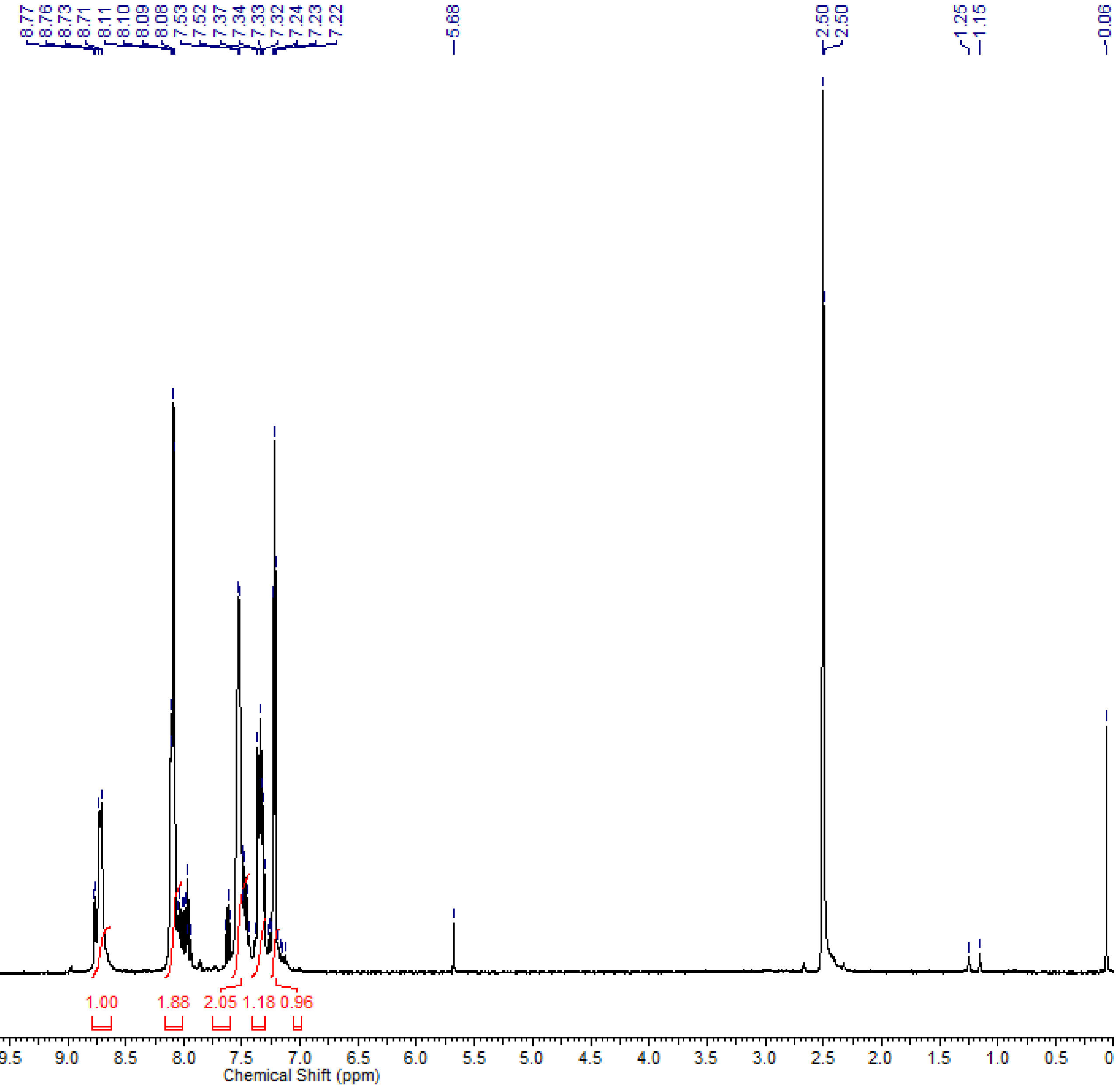

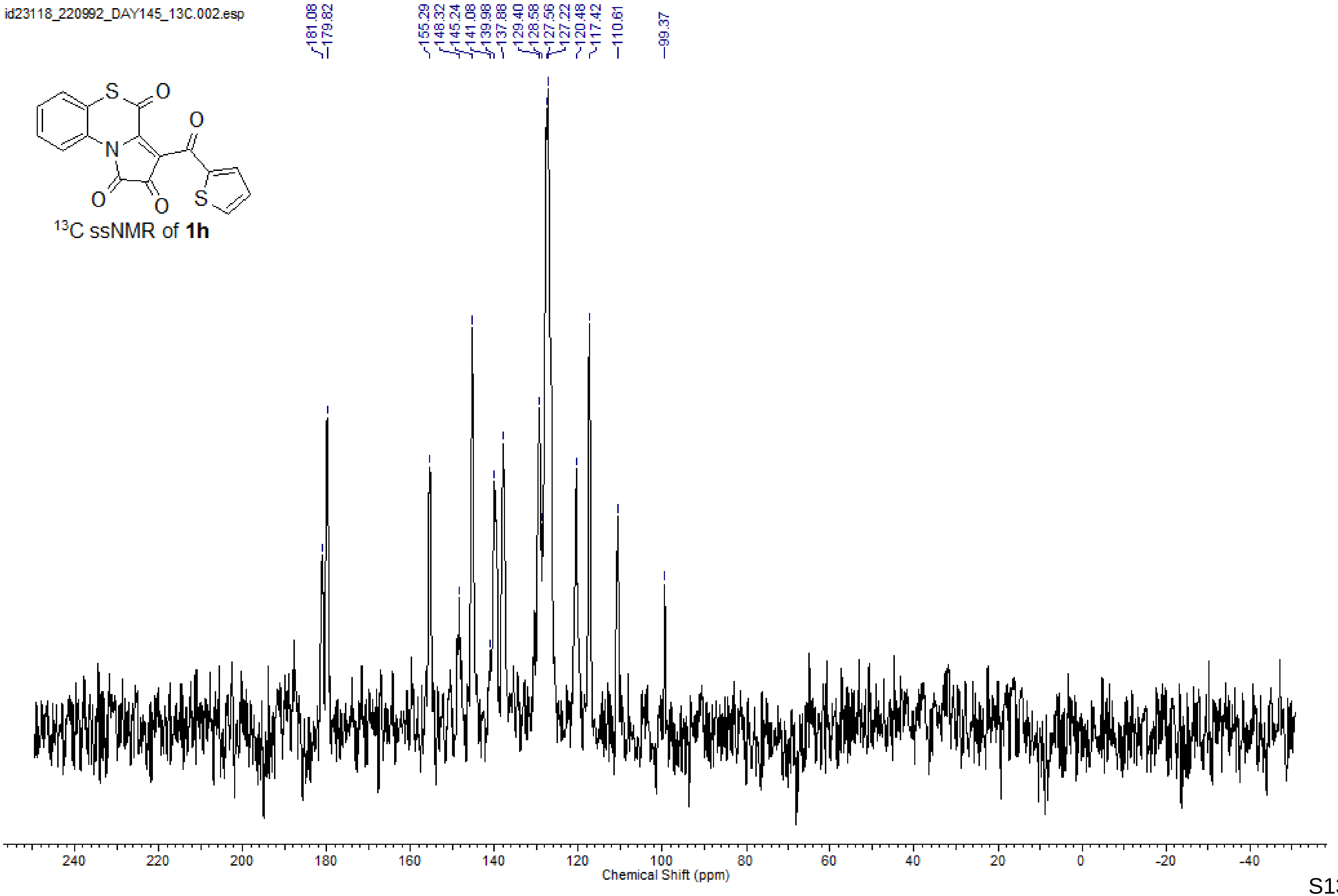

^{13}C ssNMR of **1h**

Alkyl 3-aroyl-2-hydroxy-1-oxopyrrolo[2,1-*b*][1,3]benzothiazole-3a(1*H*)-carboxylates 3. General procedure.

A mixture of APBTT **1** (0.45 mmol) and alkanol **2** (5 mL) is stirred in a closed microreaction V-vial at the b.p. of the alkanol **2** (65 °C for MeOH **2a**, 85 °C for EtOH **2c** and iPrOH **2b**) for 1-3 h (1 h for MeOH **2a**, 2.5 h EtOH **2c**, 3 h for iPrOH **2b**). Then, the resulting solution is cooled to room temperature, and the solvent is evaporated under vacuum. The evaporated reaction mass is recrystallized from toluene (the compound **3ha** is recrystallized from toluene/xylene, ~2:1); the formed precipitate of compound **3** is filtered off, and washed with toluene.

Methyl 3-benzoyl-2-hydroxy-1-oxopyrrolo[2,1-*b*][1,3]benzothiazole-3a(1*H*)-carboxylate (3aa)
Yellow solid; yield (86 mg, 52%); mp 217–219 °C (toluene).
^{1}H NMR (400 MHz, DMSO-d_6): δ = 9.78 (br.s, 1H), 7.84 (m, 2H), 7.64 (m, 1H), 7.54–7.47 (m, 3H), 7.40 (m, 1H), 7.25–7.17 (m, 2H), 3.61 (s, 3H) ppm.
^{13}C NMR (100 MHz, DMSO-d_6): δ = 188.4, 167.4, 166.5, 152.2, 136.9, 134.5, 134.2, 133.0, 128.9 (2C), 128.2 (2C), 126.6, 125.7, 122.9, 118.3, 117.7, 78.2, 53.7 ppm.
IR (mineral oil): 3284, 3083, 1740, 1704, 1669 cm^{-1}.
EA: Found: C, 62.31; H, 3.60; N, 3.90. Calc. for $C_{19}H_{13}NO_5S$: C, 62.12; H, 3.57; N, 3.81.

Methyl 2-hydroxy-3-(4-methylbenzoyl)-1-oxopyrrolo[2,1-*b*][1,3]benzothiazole-3a(1*H*)-carboxylate (3ba)
Yellow solid; yield (112 mg, 65%); mp 219–221 °C (toluene).
^{1}H NMR (400 MHz, DMSO-d_6): δ = 9.26 (br.s, 1H), 7.75 (m, 2H), 7.48 (m, 1H), 7.39 (m, 1H), 7.33 (m, 2H), 7.24–7.16 (m, 2H), 3.59 (s, 3H), 2.40 (s, 3H) ppm.
^{13}C NMR (100 MHz, DMSO-d_6): δ = 188.0, 167.4, 166.6, 151.6, 143.5, 134.6, 134.3, 134.2, 129.2 (2C), 128.8 (2C), 126.5, 125.7, 122.9, 118.5, 117.7, 78.3, 53.6, 21.1 ppm.
IR (mineral oil): 3274, 3111, 1745, 1700, 1659 cm^{-1}.
EA: Found: C, 63.17; H, 4.02; N, 3.81. Calc. for $C_{20}H_{15}NO_5S$: C, 62.98; H, 3.96; N, 3.67.

Methyl 2-hydroxy-3-(4-methoxylbenzoyl)-1-oxopyrrolo[2,1-*b*][1,3]benzothiazole-3a(1*H*)-carboxylate (3ca)
Yellow solid; yield (102 mg, 57%); mp 212–214 °C (toluene).
^{1}H NMR (400 MHz, DMSO-d_6): δ = 7.87 (m, 2H), 7.47 (m, 1H), 7.39 (m, 1H), 7.24–7.16 (m, 2H), 7.06 (m, 2H), 3.87 (s, 3H), 3.59 (s, 3H) ppm.
^{13}C NMR (100 MHz, DMSO-d_6): δ = 186.8, 167.4, 166.6, 163.4, 150.6, 134.7, 134.2, 131.6 (2C), 129.4, 126.5, 125.7, 122.9, 118.8, 117.7, 113.6 (2C), 78.3, 55.5, 53.6 ppm.

IR (mineral oil): 3184, 3089, 1755, 1732, 1666 cm^{-1}.
EA: Found: C, 61.02; H, 3.99; N, 3.61. Calc. for $C_{20}H_{15}NO_6S$: C, 60.45; H, 3.80; N, 3.52.

Methyl 3-(4-fluorobenzoyl)-2-hydroxy-1-oxopyrrolo[2,1-*b*][1,3]benzothiazole-3a(1*H*)-carboxylate (3da)
Yellow solid; yield (80 mg, 46%); mp 225–227 °C (toluene).
^{1}H NMR (400 MHz, DMSO-d_6): δ = 7.98 (m, 2H), 7.46 (m, 1H), 7.37 (m, 1H), 7.31 (m, 2H), 7.22–7.15 (m, 2H), 3.58 (s, 3H) ppm.
^{13}C NMR (100 MHz, DMSO-d_6): δ = 186.8, 167.5, 166.5, 166.1, 163.6, 152.6, 134.5, 134.2, 133.6, 133.5, 132.1, 131.9, 126.5, 125.7, 122.9, 117.8, 117.7, 115.4, 115.2, 78.2, 53.6 ppm.
IR (mineral oil): 3274, 3106, 1740, 1712, 1651 cm^{-1}.
EA: Found: C, 59.43; H, 3.25; N, 3.70. Calc. for $C_{19}H_{12}FNO_5S$: C, 59.22; H, 3.14; N, 3.63.

Methyl 3-(4-chlorobenzoyl)-2-hydroxy-1-oxopyrrolo[2,1-*b*][1,3]benzothiazole-3a(1*H*)-carboxylate (3ea)
Yellow solid; yield (85 mg, 47%); mp 229–231 °C (toluene).
^{1}H NMR (400 MHz, DMSO-d_6): δ = 8.80 (br.s, 1H), 7.87 (m, 2H), 7.59 (m, 2H), 7.48 (m, 1H), 7.39 (m, 1H), 7.24–7.16 (m, 2H), 3.60 (s, 3H) ppm.
^{13}C NMR (100 MHz, DMSO-d_6): δ = 187.0, 167.5, 166.5, 153.2, 137.7, 135.7, 134.4, 134.2, 130.8 (2C), 128.3 (2C), 126.5, 125.7, 122.9, 117.7, 117.6, 78.2, 53.6 ppm.
IR (mineral oil): 3232, 3071, 1751, 1725, 1660 cm^{-1}.
EA: Found: C, 56.94; H, 3.07; N, 3.56. Calc. for $C_{19}H_{12}ClNO_5S$: C, 56.79; H, 3.01; N, 3.49.

Methyl 3-(4-bromobenzoyl)-2-hydroxy-1-oxopyrrolo[2,1-*b*][1,3]benzothiazole-3a(1*H*)-carboxylate (3fa)
Yellow solid; yield (93 mg, 46%); mp 229–231 °C (toluene).
^{1}H NMR (400 MHz, DMSO-d_6): δ = 9.82 (br.s, 1H), 7.79 (m, 2H), 7.73 (m, 2H), 7.48 (m, 1H), 7.39 (m, 1H), 7.24–7.16 (m, 2H), 3.60 (s, 3H) ppm.
^{13}C NMR (100 MHz, DMSO-d_6): δ = 187.2, 167.5, 166.5, 153.3, 136.1, 134.4, 134.2, 131.3 (2C), 130.9 (2C), 126.8, 126.5, 125.7, 122.9, 117.7, 117.6, 78.2, 53.6 ppm.
IR (mineral oil): 3239, 3075, 1750, 1724, 1659 cm^{-1}.
EA: Found: C, 51.52; H, 2.99; N, 3.20. Calc. for $C_{19}H_{12}BrNO_5S$: C, 51.14; H, 2.71; N, 3.14.

Methyl 3-(furan-2-ylcarbonyl)-2-hydroxy-1-oxopyrrolo[2,1-*b*][1,3]benzothiazole-3a(1*H*)-carboxylate (3ga)
Yellow solid; yield (88 mg, 55%); mp 155–157 °C (toluene).
^{1}H NMR (400 MHz, DMSO-d_6): δ = 10.34 (br.s, 1H), 8.04–7.78 (m, 2H), 7.46 (m, 2H), 7.19 (m, 2H), 6.76 (m, 1H), 3.59 (s, 3H) ppm.

^{13}C NMR (100 MHz, DMSO-d_6): δ = 173.2, 167.6, 166.7, 152.6, 151.1, 148.1, 134.6, 134.0, 126.6, 125.6, 122.8, 120.5, 118.1, 117.7, 112.4, 78.8, 53.7 ppm.
IR (mineral oil): 3179, 3129, 1730, 1705, 1665 cm^{-1}.
EA: Found: C, 57.25; H, 3.32; N, 3.99. Calc. for $C_{17}H_{11}NO_6S$: C, 57.14; H, 3.10; N, 3.92.

Methyl 2-hydroxy-1-oxo-3-(thiophen-2-ylcarbonyl)pyrrolo[2,1-*b*][1,3]benzothiazole-3a(1*H*)-carboxylate (3ha)
Yellow solid; yield (71 mg, 42%); mp 208–210 °C (toluene : xylene, 2 : 1).
^{1}H NMR (400 MHz, DMSO-d_6): δ = 10.63 (br.s, 1H), 8.35 (m, 1H), 8.04 (m, 1H), 7.46 (m, 1H), 7.38 (m, 1H), 7.28 (m, 1H), 7.22–7.16 (m, 2H), 3.59 (s, 3H) ppm.
^{13}C NMR (100 MHz, DMSO-d_6): δ = 178.9, 167.6, 166.8, 152.4, 143.2, 135.3, 134.6, 134.4, 134.1, 128.5, 126.6, 125.6, 122.9, 118.3, 117.7, 78.9, 53.7 ppm.
IR (mineral oil): 3269, 3085, 1739, 1707, 1663 cm^{-1}.
EA: Found: C, 54.94; H, 3.00; N, 3.51. Calc. for $C_{17}H_{11}NO_5S_2$: C, 54.68; H, 2.97; N, 3.75.

Isopropyl 3-benzoyl-2-hydroxy-1-oxopyrrolo[2,1-*b*][1,3]benzothiazole-3a(1*H*)-carboxylate (3ab)
Yellow solid; yield (80 mg, 45%); mp 193–195 °C (toluene).
^{1}H NMR (400 MHz, DMSO-d_6): δ = 7.85 (m, 2H), 7.65 (m, 1H), 7.54 (m, 2H), 7.48 (m, 1H), 7.40 (m, 1H), 7.25–7.16 (m, 2H), 4.80 (m, 1H), 0.99 (d, J = 8 Hz, 3H), 0.94 (d, J = 8 Hz, 3H) ppm.
^{13}C NMR (100 MHz, DMSO-d_6): δ = 188.6, 166.3, 166.0, 151.3, 136.8, 134.9, 134.3, 133.1, 128.9 (2C), 128.2 (2C), 126.4, 125.7, 122.9, 118.1, 117.6, 78.1, 70.3, 20.8 (2C) ppm.
IR (mineral oil): 3255, 3065, 1733, 1698, 1650 cm^{-1}.
EA: Found: C, 63.96; H, 4.43; N, 3.62. Calc. for $C_{21}H_{17}NO_5S$: C, 63.79; H, 4.33; N, 3.54.

Isopropyl 2-hydroxy-3-(4-methylbenzoyl)-1-oxopyrrolo[2,1-*b*][1,3]benzothiazole-3a(1*H*)-carboxylate (3bb)
Yellow solid; yield (63 mg, 34%); mp 192–194 °C (toluene).
^{1}H NMR (400 MHz, DMSO-d_6): δ = 7.76 (m, 2H), 7.47 (m, 1H), 7.39 (m, 1H), 7.34 (m, 2H), 7.24–7.16 (m, 2H), 4.79 (m, 1H), 2.40 (s, 3H), 0.98 (d, J = 8 Hz, 3H), 0.93 (d, J = 8 Hz, 3H) ppm.
^{13}C NMR (100 MHz, DMSO-d_6): δ = 188.8, 166.8, 166.8, 151.2, 144.3, 135.1, 134.9, 134.8, 129.7 (2C), 129.4 (2C), 127.0, 126.3, 123.5, 118.9, 118.2, 78.7, 70.9, 21.7, 21.4 (2C) ppm.
IR (mineral oil): 3258, 3070, 1733, 1700, 1651 cm^{-1}.
EA: Found: C, 64.86; H, 4.72 N, 3.48. Calc. for $C_{22}H_{19}NO_5S$: C, 64.53; H, 4.68; N, 3.42.

Isopropyl 2-hydroxy-3-(4-methoxylbenzoyl)-1-oxopyrrolo[2,1-*b*][1,3]benzothiazole-3a(1*H*)-carboxylate (3cb)
Yellow solid; yield (69 mg, 36%); mp 193–195 °C (toluene).
^{1}H NMR (400 MHz, DMSO-d_6): δ = 7.87 (m, 2H), 7.47 (m, 1H), 7.39 (m, 1H), 7.24–7.16 (m, 2H), 7.07 (m, 2H), 4.78 (1H), 3.87 (s, 3H), 0.98 (d, *J* = 4 Hz, 3H), 0.91 (d, *J* = 8 Hz, 3H) ppm.
^{13}C NMR (100 MHz, DMSO-d_6): δ = 187.0, 166.4, 166.0, 163.4, 149.7, 135.0, 134.4, 131.6 (2C), 129.4, 126.4, 125.7, 122.9, 118.5, 117.6, 113.7 (2C), 78.1, 70.3, 55.5, 20.8 (2C) ppm.
IR (mineral oil): 3227, 3075, 1742, 1694, 1644 cm^{-1}.
EA: Found: C, 62.86; H, 4.72 N, 3.48. Calc. for $C_{22}H_{19}NO_6S$: C, 62.11; H, 4.50; N, 3.29.

Isopropyl 3-(4-chlorobenzoyl)-2-hydroxy-1-oxopyrrolo[2,1-*b*][1,3]benzothiazole-3a(1*H*)-carboxylate (3eb)
Yellow solid; yield (58 mg, 30%); mp 202–204°C (toluene).
^{1}H NMR (400 MHz, DMSO-d_6): δ = 9.37 (br.s, 1H), 7.87 (m, 2H), 7.60 (m, 2H), 7.47 (m, 1H), 7.39 (m, 1H), 7.24–7.16 (m, 2H), 4.83–4.75 (m, 1H), 0.97 (m, 6H) ppm.
^{13}C NMR (100 MHz, DMSO-d_6): δ = 187.3, 166.3, 166.0, 152.2, 137.8, 135.6, 134.8, 134.3, 130.8 (2C), 128.4 (2C), 126.4, 125.7, 122.9, 117.6, 117.5, 78.0, 70.3, 20.8 (2C) ppm.
IR (mineral oil): 3248, 3074, 1735, 1703, 1657 cm^{-1}.
EA: Found: C, 58.83; H, 3.79; N, 3.30. Calc. for $C_{21}H_{16}ClNO_5S$: C, 58.68; H, 3.75; N, 3.26.

Isopropyl 3-(4-bromobenzoyl)-2-hydroxy-1-oxopyrrolo[2,1-*b*][1,3]benzothiazole-3a(1*H*)-carboxylate (3fb)
Yellow solid; yield (178 mg, 87%); mp 186–188 °C (toluene).
^{1}H NMR (400 MHz, DMSO-d_6): δ = 8.20 (br.s, 1H), 7.80–7.73 (m, 4H), 7.47 (m, 1H), 7.39 (m, 1H), 7.24–7.16 (m, 2H), 4.80 (m, 1H), 1.00 (d, *J* = 4 Hz, 3H), 0.96 (d, *J* = 4 Hz, 3H) ppm.
^{13}C NMR (100 MHz, DMSO-d_6): δ = (ppm) 187.5, 166.3, 166.1, 152.4, 136.0, 134.8, 134.3, 131.3 (2C), 130.9 (2C), 126.9, 126.4, 125.6, 122.9, 117.6, 117.4, 78.1, 70.3, 20.8 (2C) ppm.
IR (mineral oil): 3202, 3063, 1737, 1712, 1659 cm^{-1}.
EA: Found: C, 53.41; H, 3.68; N, 3.07. Calc. for $C_{21}H_{16}BrNO_5S$: C, 53.18; H, 3.40; N, 2.95.

Isopropyl 2-hydroxy-1-oxo-3-(thiophen-2-ylcarbonyl)pyrrolo[2,1-*b*][1,3]benzothiazole-3a(1*H*)-carboxylate (3hb)
Yellow solid; yield (116 mg, 64%); mp 170–172°C (toluene, xylene).
^{1}H NMR (400 MHz, DMSO-d_6): δ = 9.96 (br.s, 1H), 8.23 (m, 1H), 8.06 (m, 1H), 7.46 (m, 1H), 7.38 (m, 1H), 7.28 (m, 1H), 7.23–7.15 (m, 2H), 4.77 (m, 1H), 0.99 (d, *J* = 4 Hz, 3H), 0.93 (d, *J* = 4 Hz, 3H) ppm.

^{13}C NMR (100 MHz, DMSO-d_6): δ = 179.5, 166.5, 166.0, 151.0, 143.2, 135.5, 134.9, 134.7, 134.3, 128.5, 126.4, 125.6, 122.8, 118.0, 117.6, 78.5, 70.3, 20.8, 20.7 ppm.
IR (mineral oil): 3245, 3098, 1733, 1699, 1672 cm^{-1}.
EA: Found: C, 57.12; H, 3.89; N, 3.61. Calc. for $C_{19}H_{15}NO_5S_2$: C, 56.85; H, 3.77; N, 3.49.

Ethyl 3-benzoyl-2-hydroxy-1-oxopyrrolo[2,1-*b*][1,3]benzothiazole-3a(1*H*)-carboxylate (3ac)
Yellow solid; yield (70 mg, 41%); mp 207–209 °C (toluene).
^{1}H NMR (400 MHz, DMSO-d_6): δ = 7.84 (m, 2H), 7.64 (m, 1H), 7.53 (m, 2H), 7.47 (m, 1H), 7.40 (m, 1H), 7.24–7.16 (m, 2H), 4.05 (m, 2H), 0.96 (t, J = 8 Hz, 3H) ppm.
^{13}C NMR (100 MHz, DMSO-d_6): δ = 188.7, 166.8, 166.5, 151.8, 136.9, 134.8, 134.3, 133.2, 129.1 (2C), 128.3 (2C), 126.6, 125.8, 123.1, 118.2, 117.8, 78.2, 62.6, 13.5 ppm.
IR (mineral oil): 3249, 3068, 1738, 1704, 1661 cm^{-1}.
EA: Found: C, 63.31; H, 4.18; N, 3.83. Calc. for $C_{20}H_{15}NO_5S$: C, 62.98; H, 3.96; N, 3.67.

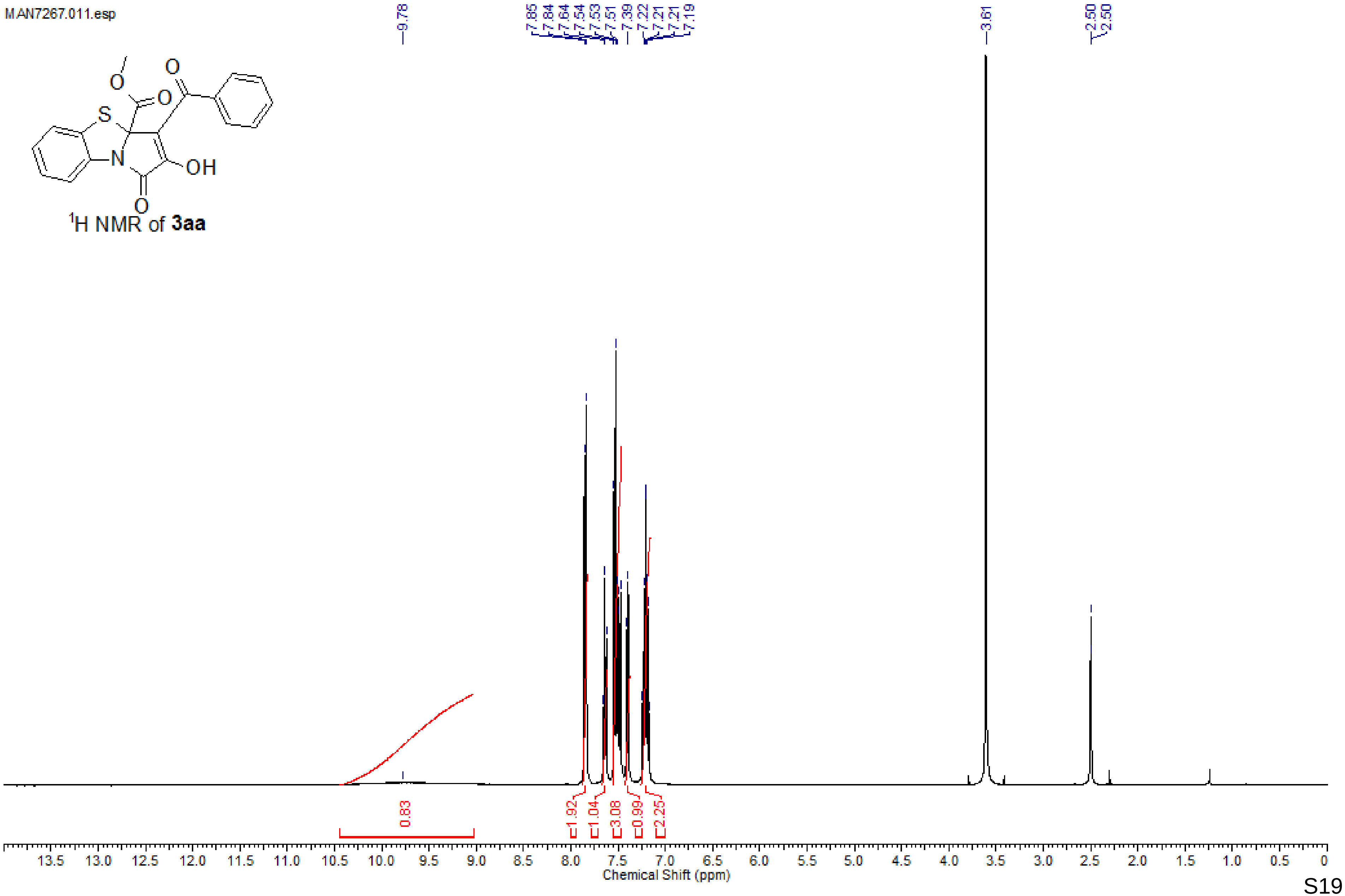
MAN7267.011.esp
S
N
O
O
O
O
OH
O
9.78
7.85
7.84
7.64
7.54
7.53
7.51
7.39
7.22
7.21
7.21
7.19
3.61
2.50
2.50
0.83
1.92
1.04
3.08
0.99
2.25
13.5
13.0
12.5
12.0
11.5
11.0
10.5
10.0
9.5
9.0
8.5
8.0
7.5
7.0
6.5
6.0
5.5
5.0
4.5
4.0
3.5
3.0
2.5
2.0
1.5
1.0
0.5
0
Chemical Shift (ppm)

^1H NMR of **3aa**

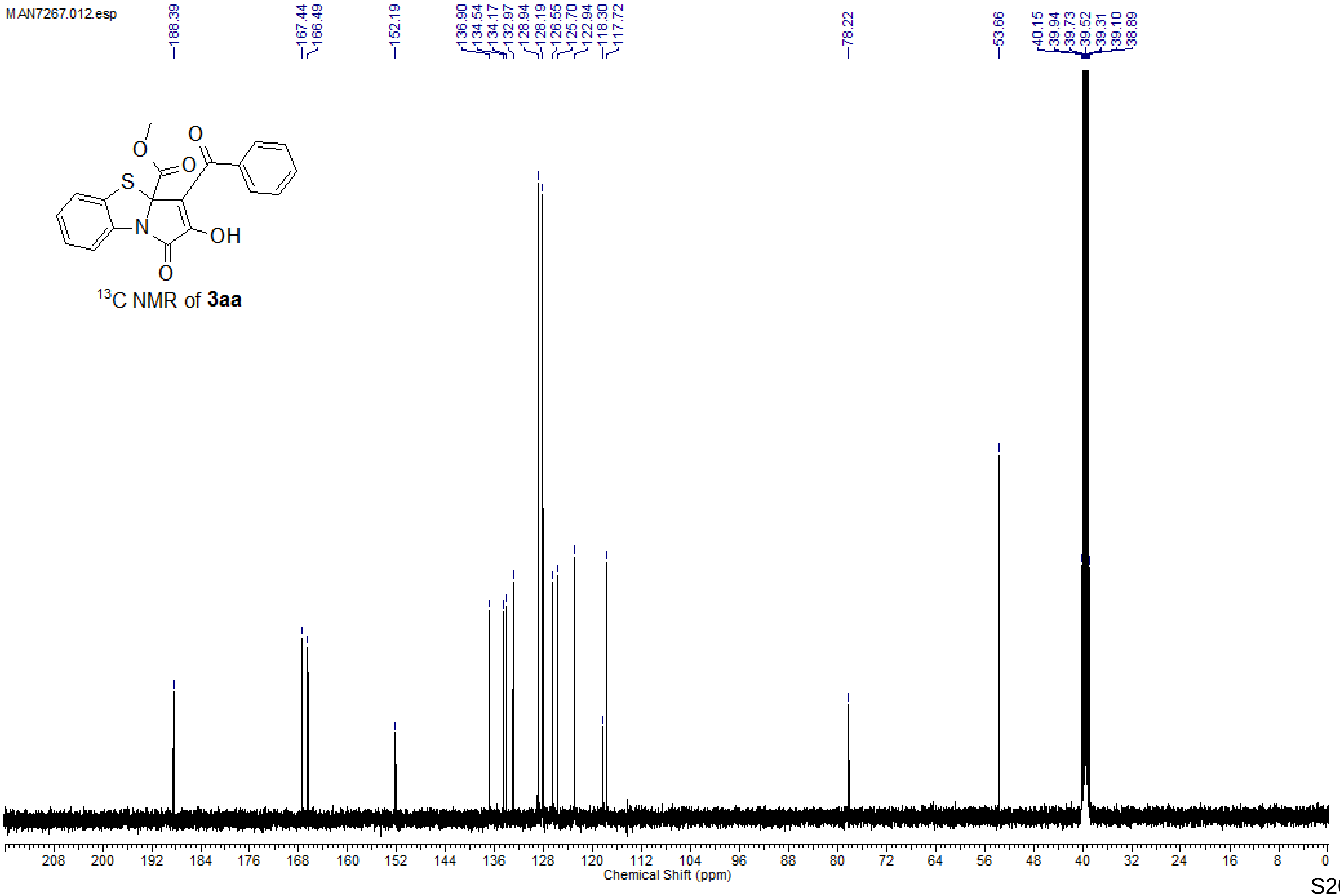

^{13}C NMR of **3aa**

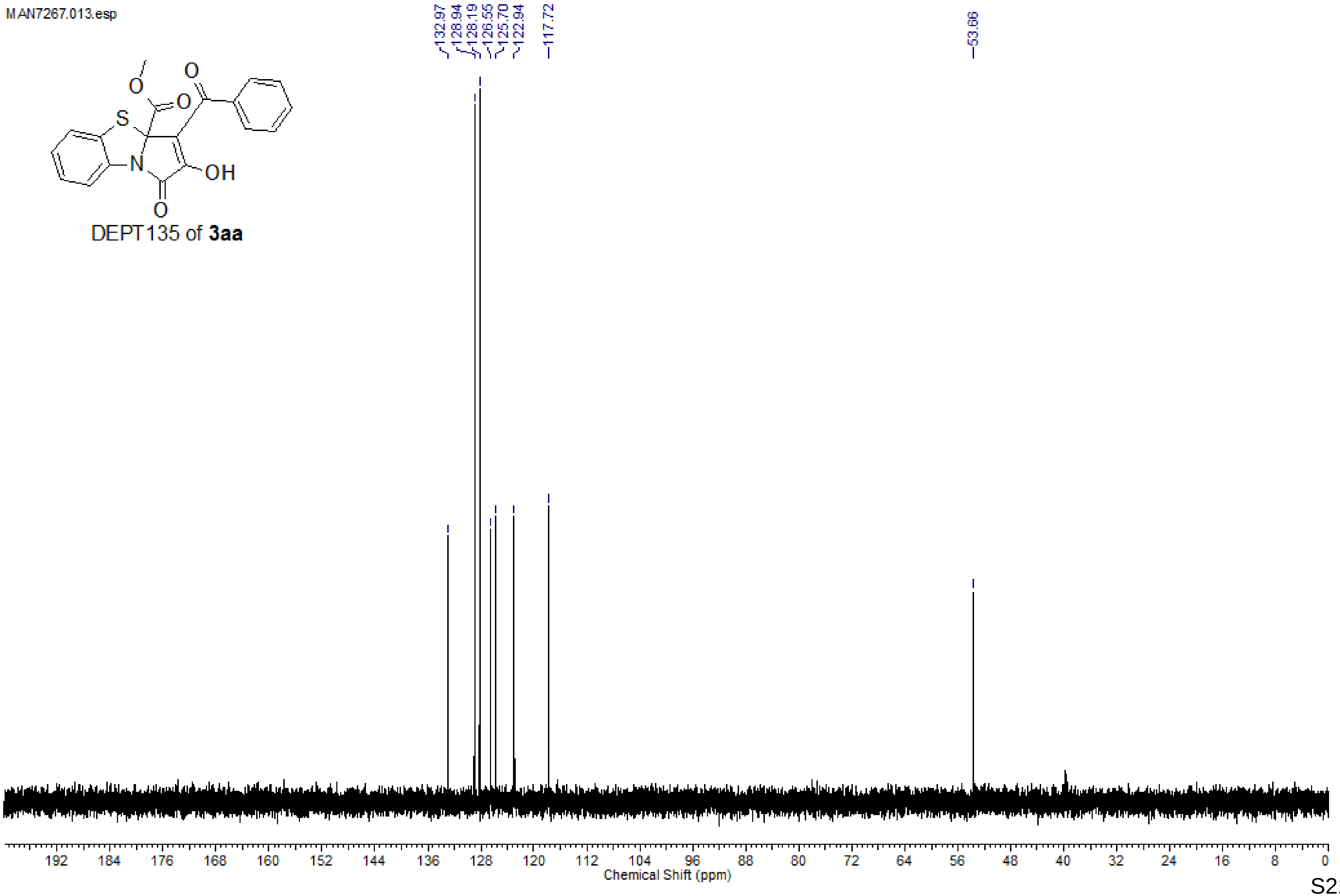

DEPT135 of **3aa**

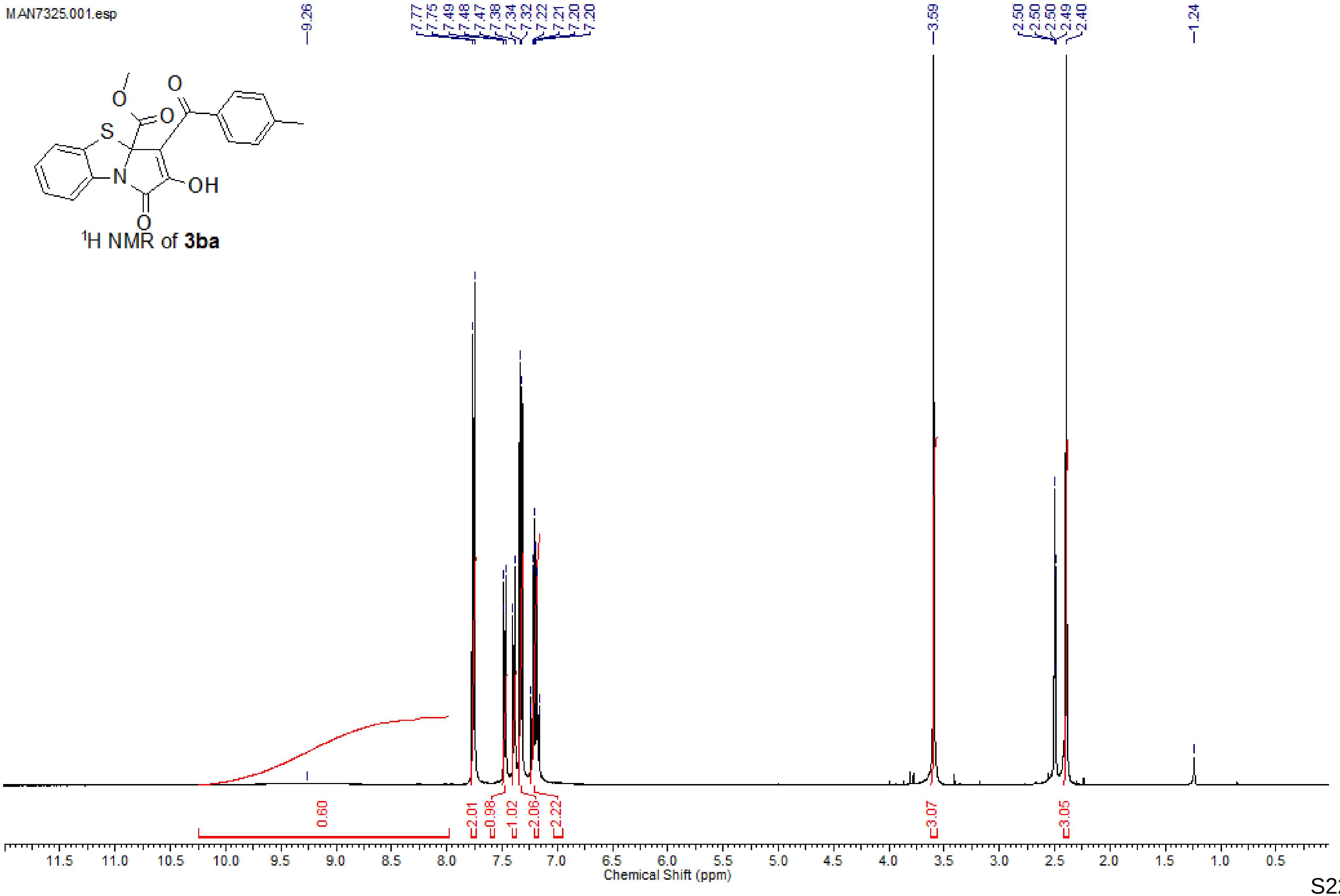
MAN7325.001.esp
9.26
7.77
7.75
7.49
7.48
7.47
7.38
7.34
7.32
7.22
7.21
7.20
7.20
3.59
2.50
2.50
2.50
2.49
2.40
1.24
O
O
O
S
N
OH
O
¹H NMR of **3ba**
0.60
2.01
0.98
1.02
2.06
2.22
3.07
3.05
11.5 11.0 10.5 10.0 9.5 9.0 8.5 8.0 7.5 7.0 6.5 6.0 5.5 5.0 4.5 4.0 3.5 3.0 2.5 2.0 1.5 1.0 0.5
Chemical Shift (ppm)

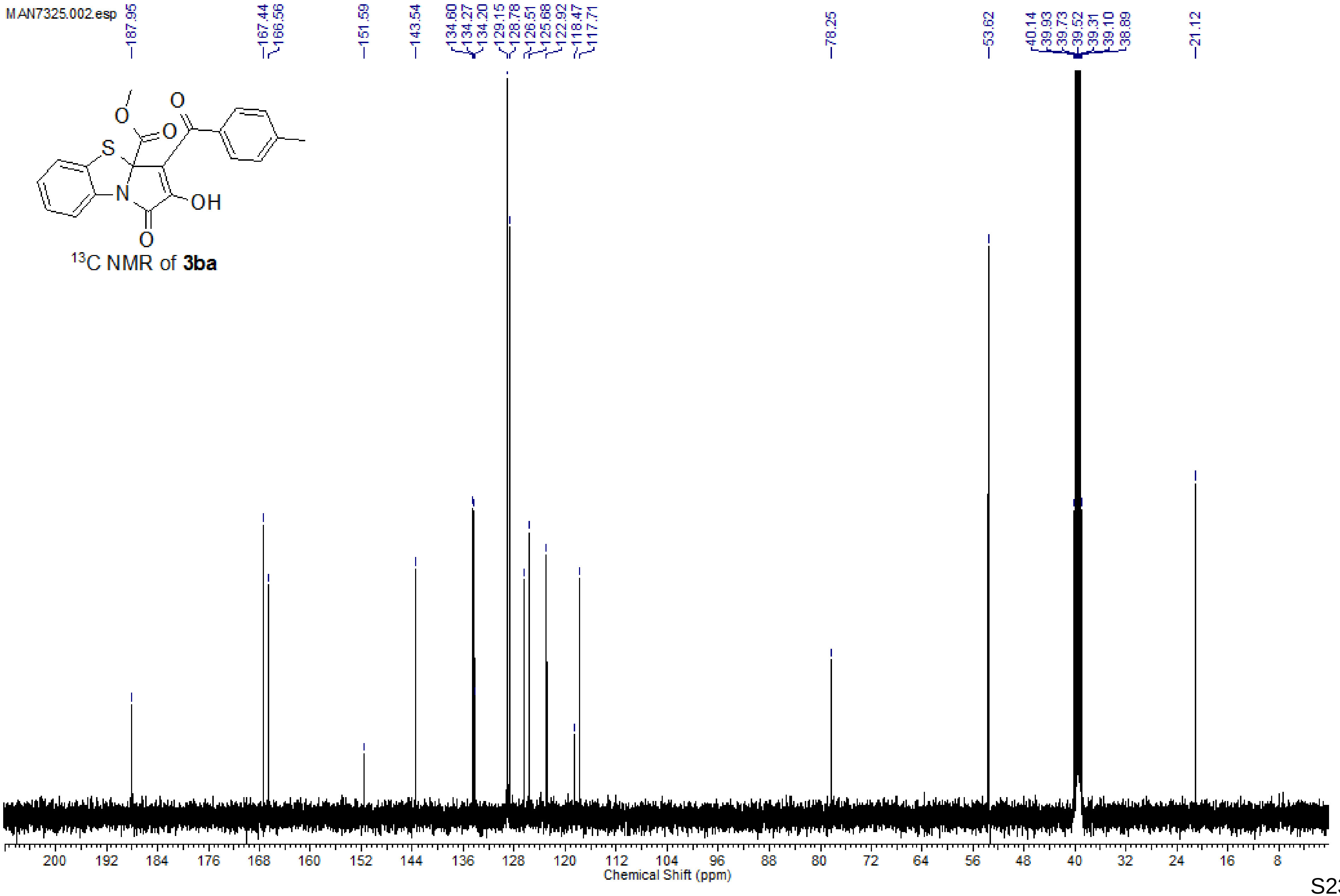

^{13}C NMR of **3ba**

MAN7325.003.esp

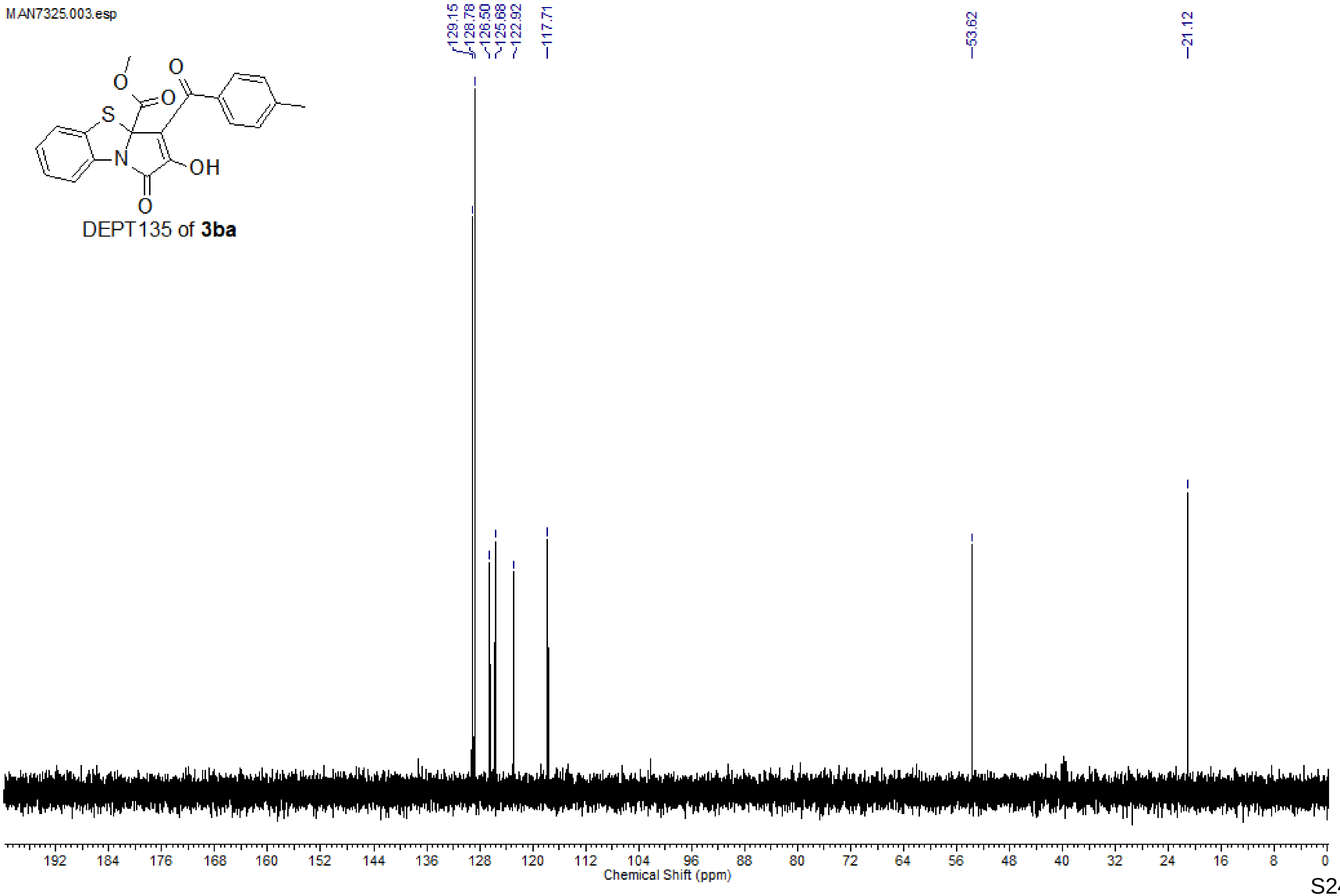

DEPT135 of **3ba**

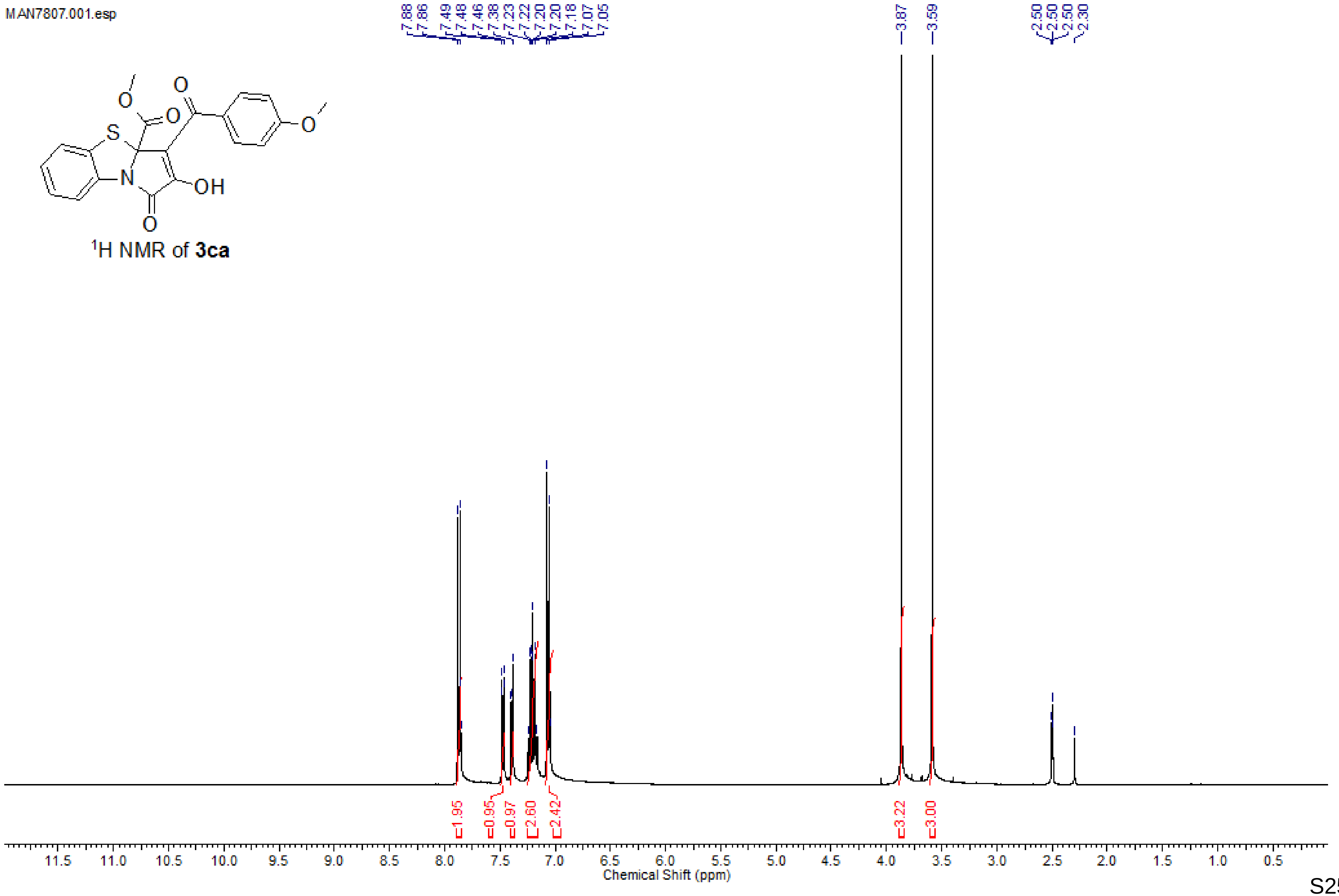
MAN7807.001.esp
7.88 7.86 7.49 7.48 7.46 7.38 7.23 7.22 7.20 7.20 7.18 7.07 7.05
3.87
3.59
2.50 2.50 2.50 2.30
O
O
O
S
N
OH
O
O
1.95
0.95
0.97
2.60
2.42
3.22
3.00
11.5 11.0 10.5 10.0 9.5 9.0 8.5 8.0 7.5 7.0 6.5 6.0 5.5 5.0 4.5 4.0 3.5 3.0 2.5 2.0 1.5 1.0 0.5
Chemical Shift (ppm)

^{1}H NMR of **3ca**

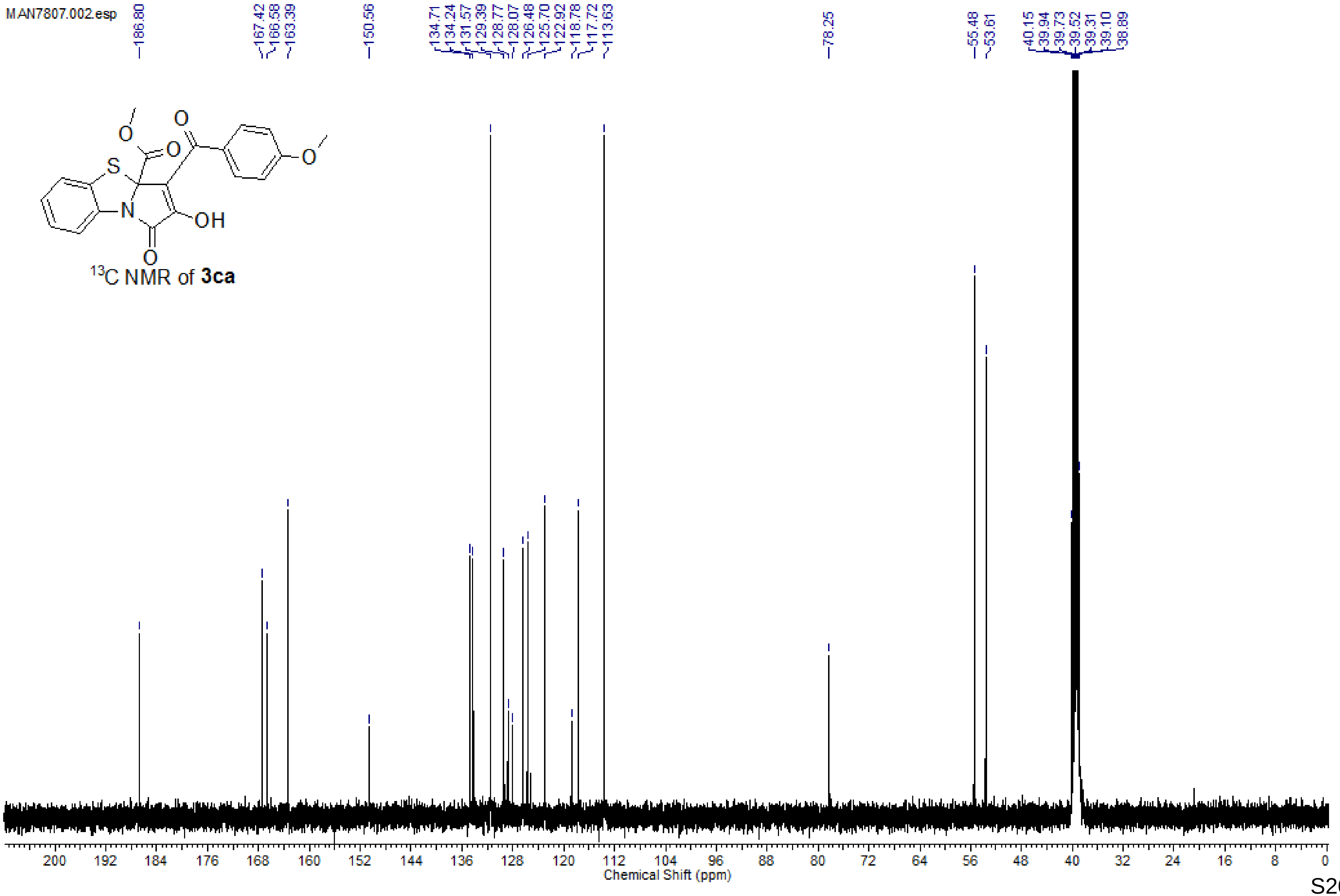

MAN7807.002.esp
186.80
167.42
166.58
163.39
150.56
134.71
134.24
131.57
129.39
128.77
128.07
126.48
125.70
122.92
118.78
117.72
113.63
78.25
55.48
53.61
40.15
39.94
39.73
39.52
39.31
39.10
38.89
O
O
O
S
N
OH
O
O
^{13}C NMR of **3ca**
200
192
184
176
168
160
152
144
136
128
120
112
104
96
88
80
72
64
56
48
40
32
24
16
8
0
Chemical Shift (ppm)

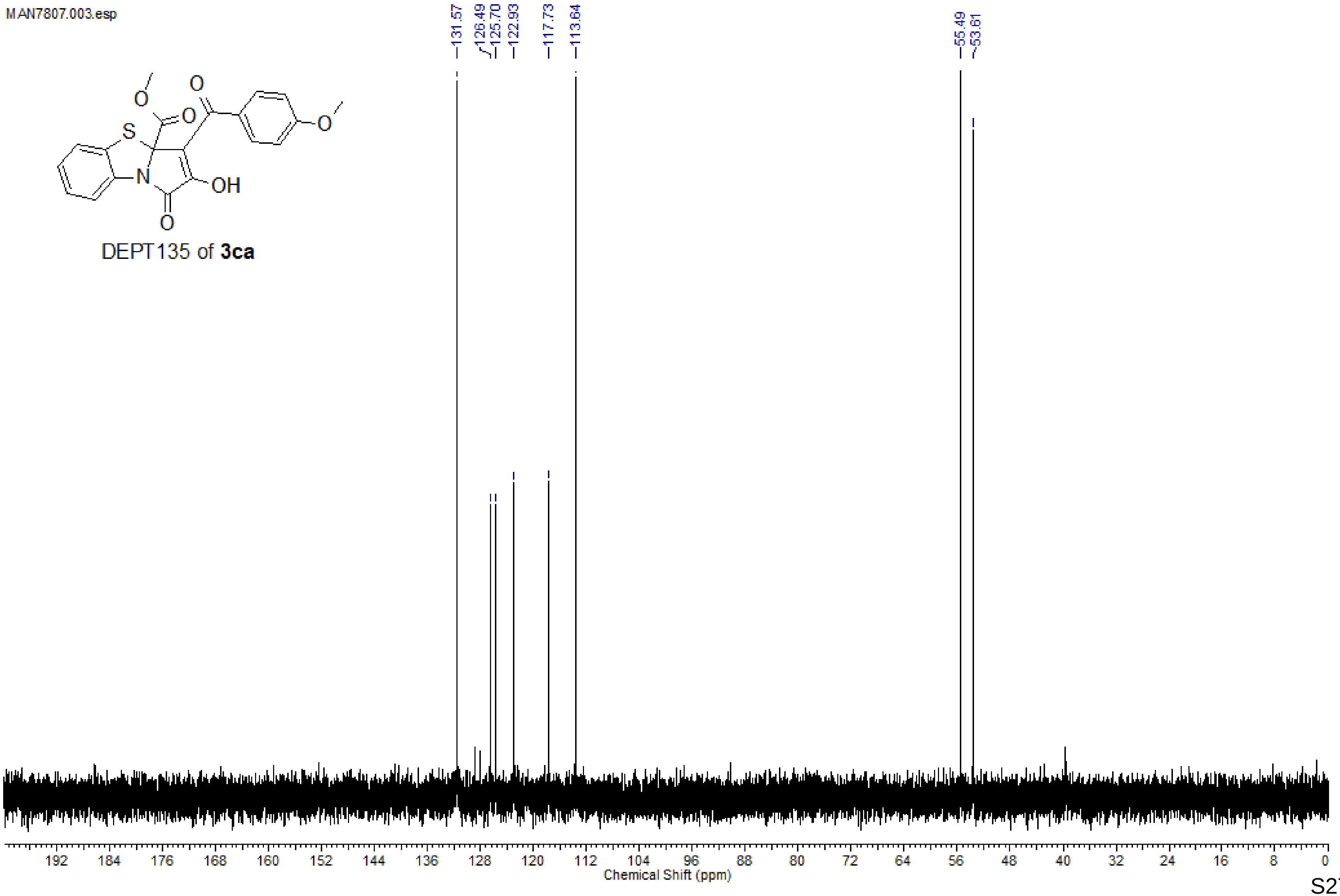

MAN7807.003.esp
DEPT135 of 3ca
131.57
126.49
125.70
122.93
117.73
113.64
55.49
53.61
Chemical Shift (ppm)

MAN7902.004.esp

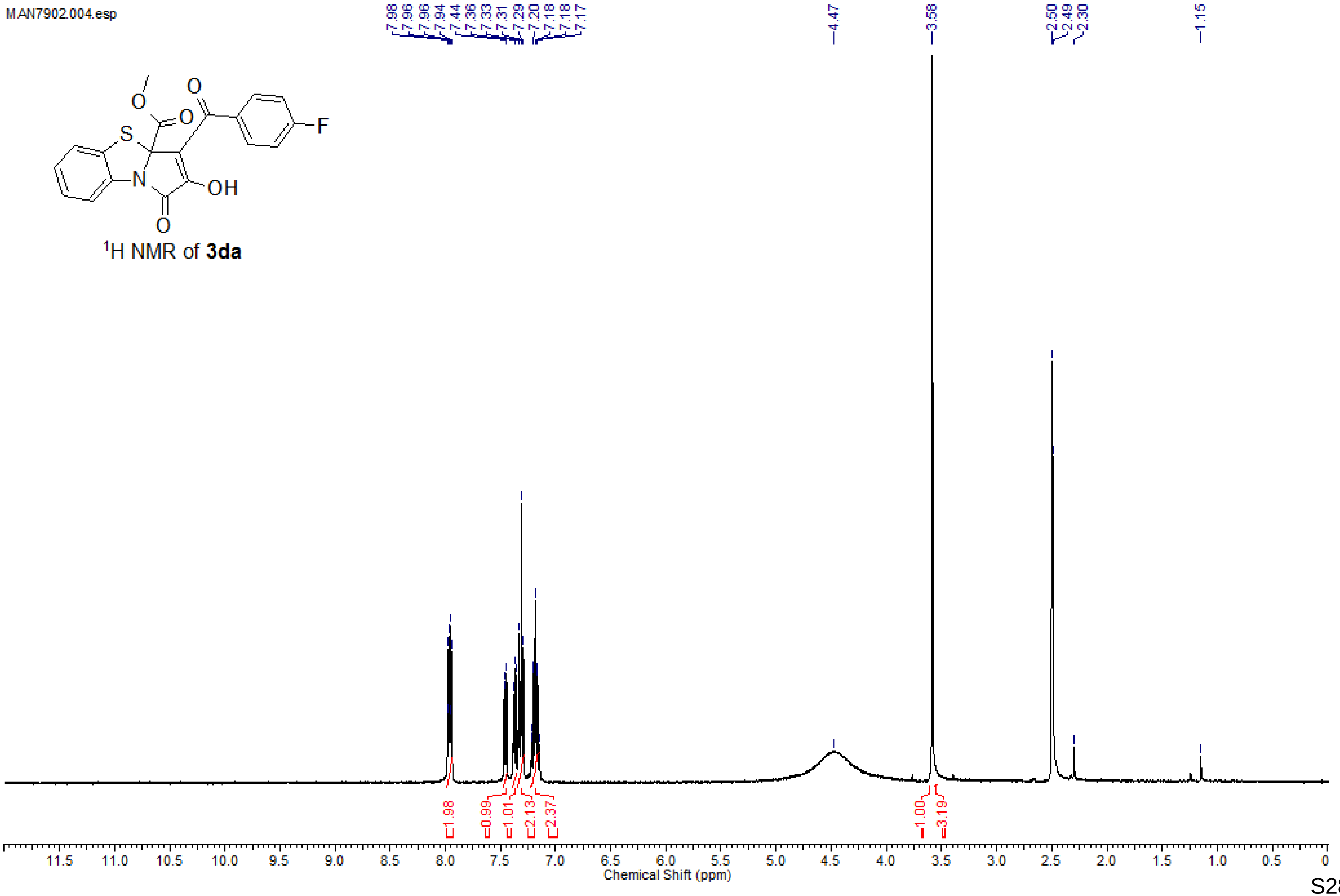

^{1}H NMR of **3da**

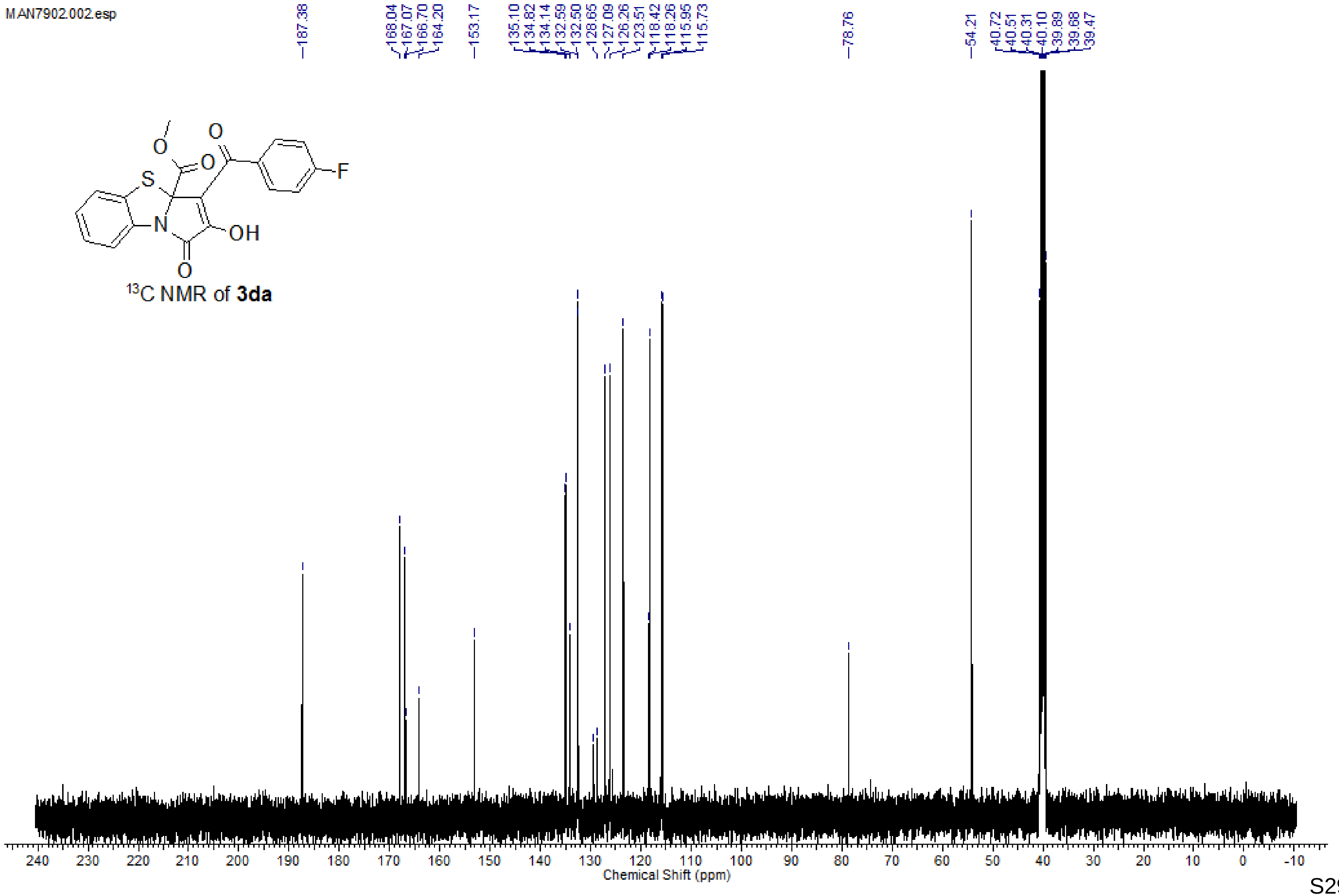

^{13}C NMR of **3da**

MAN7902.003.esp

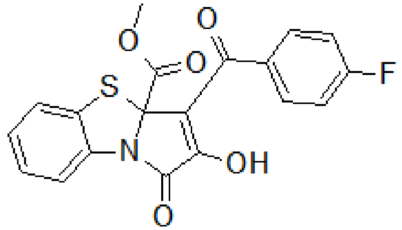

DEPT135 of **3da**

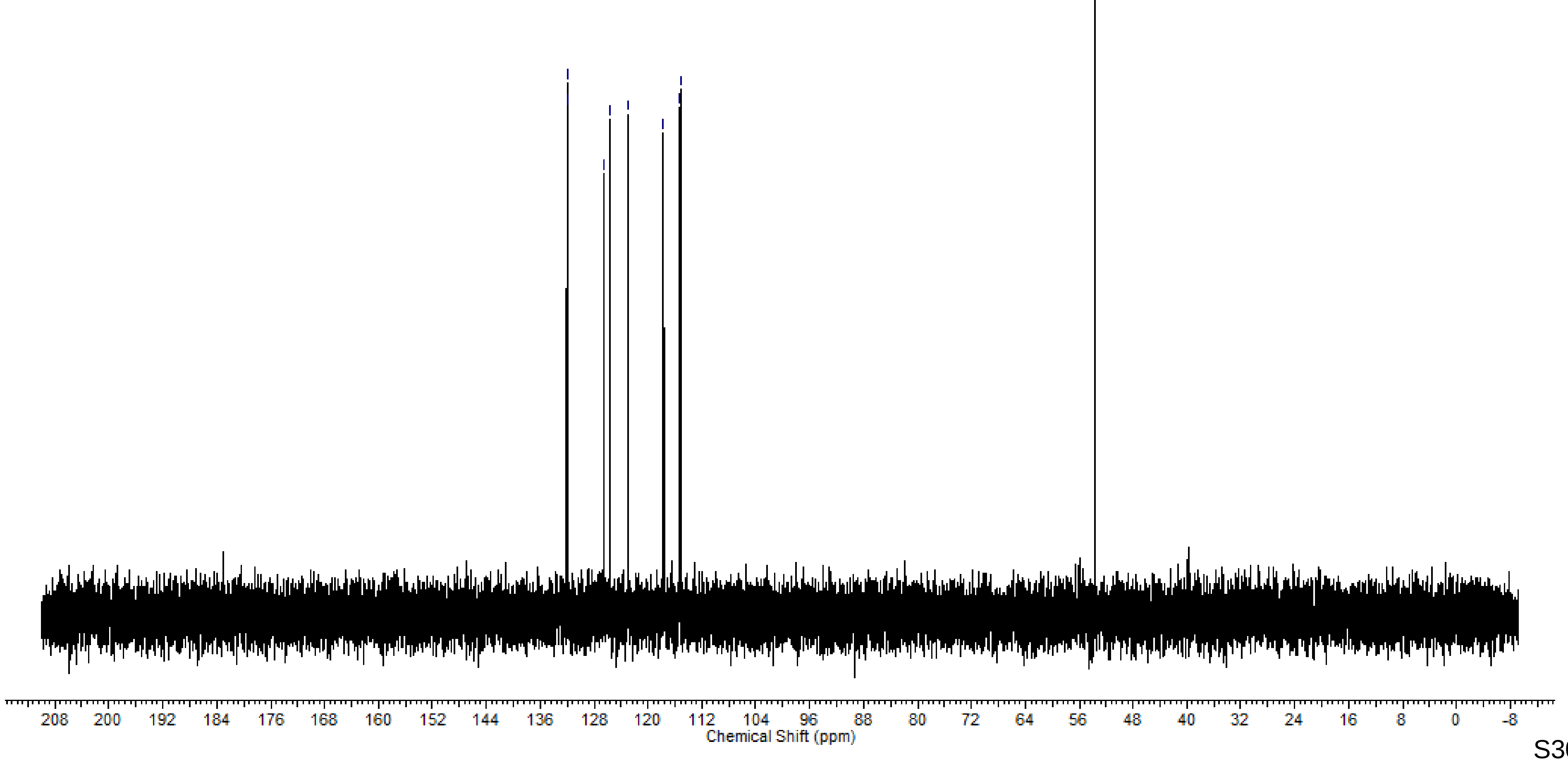

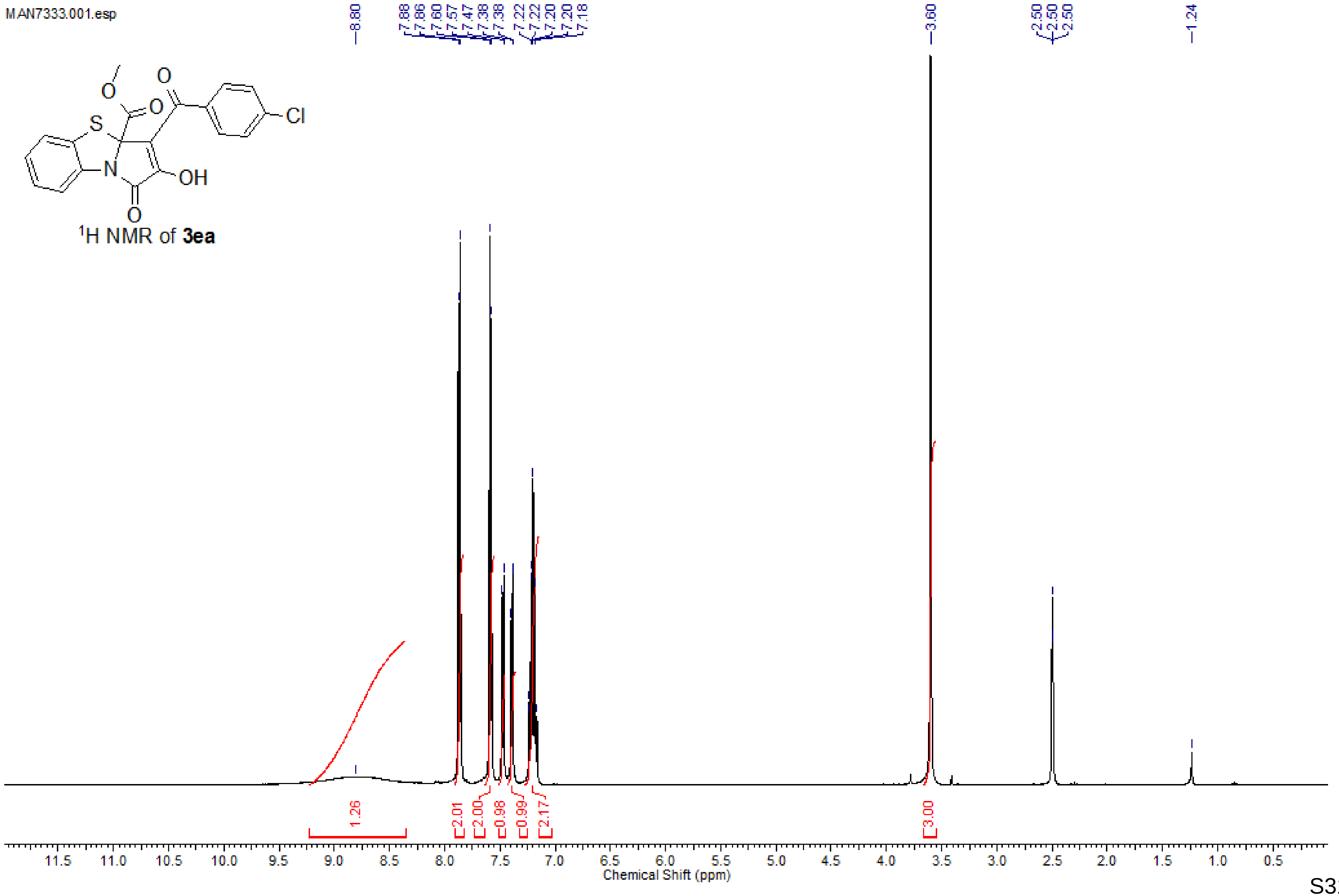
MAN7333.001.esp
O
O
O
S
N
OH
O
Cl
8.80
7.88
7.86
7.60
7.57
7.47
7.38
7.38
7.22
7.22
7.20
7.20
7.18
3.60
2.50
2.50
2.50
1.24
1.26
2.01
2.00
0.98
0.99
2.17
3.00
11.5
11.0
10.5
10.0
9.5
9.0
8.5
8.0
7.5
7.0
6.5
6.0
5.5
5.0
4.5
4.0
3.5
3.0
2.5
2.0
1.5
1.0
0.5
Chemical Shift (ppm)

^{1}H NMR of **3ea**

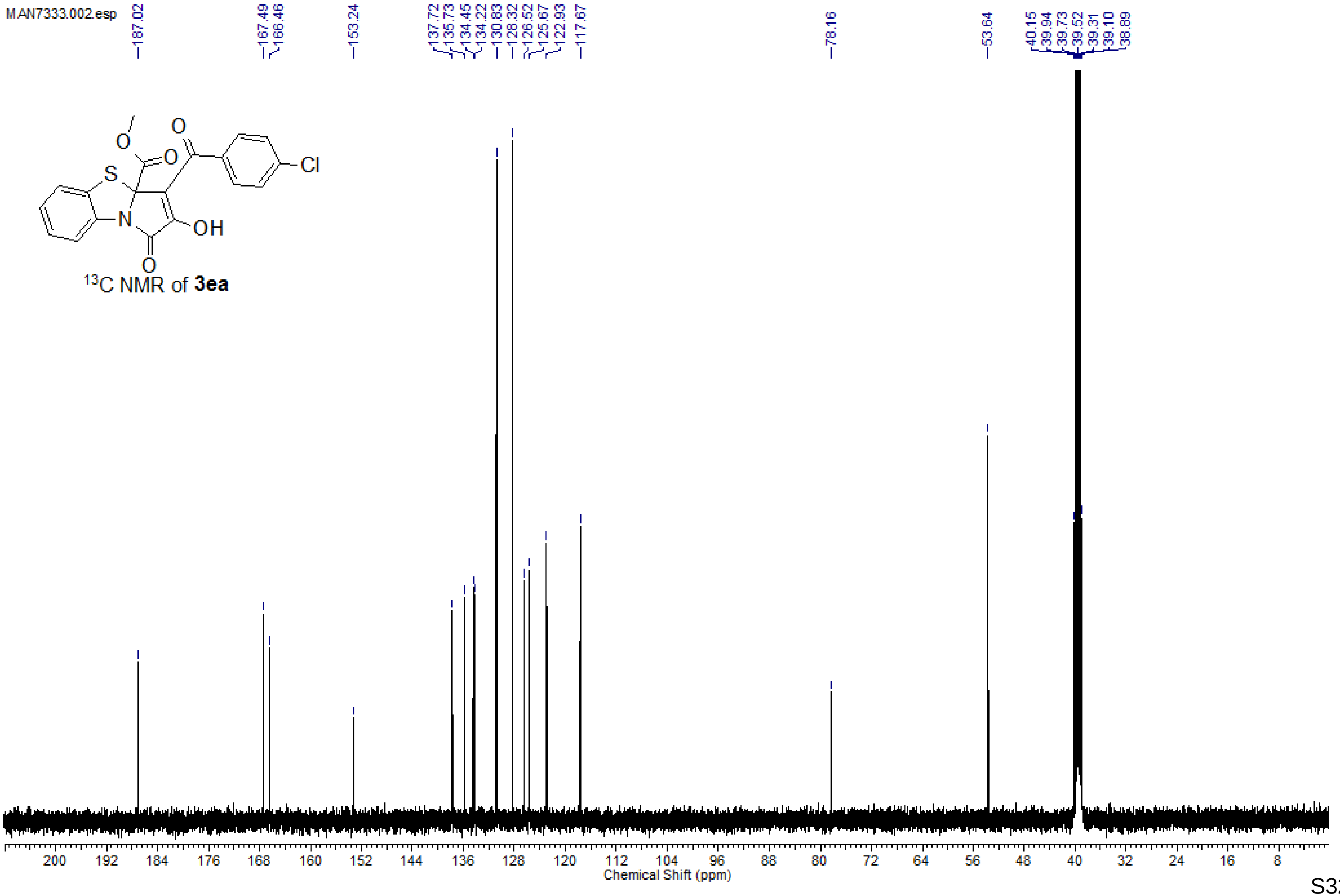

MAN7333.002.esp
187.02
167.49
166.46
153.24
137.72
135.73
134.45
134.22
130.83
128.32
126.52
125.67
122.93
117.67
78.16
53.64
40.15
39.94
39.73
39.52
39.31
39.10
38.89
O
O
O
S
N
OH
O
Cl
^{13}C NMR of **3ea**
200
192
184
176
168
160
152
144
136
128
120
112
104
96
88
80
72
64
56
48
40
32
24
16
8
Chemical Shift (ppm)

MAN7333.003.esp

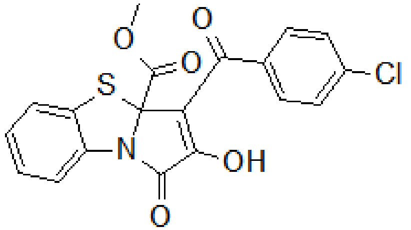

DEPT135 of **3ea**

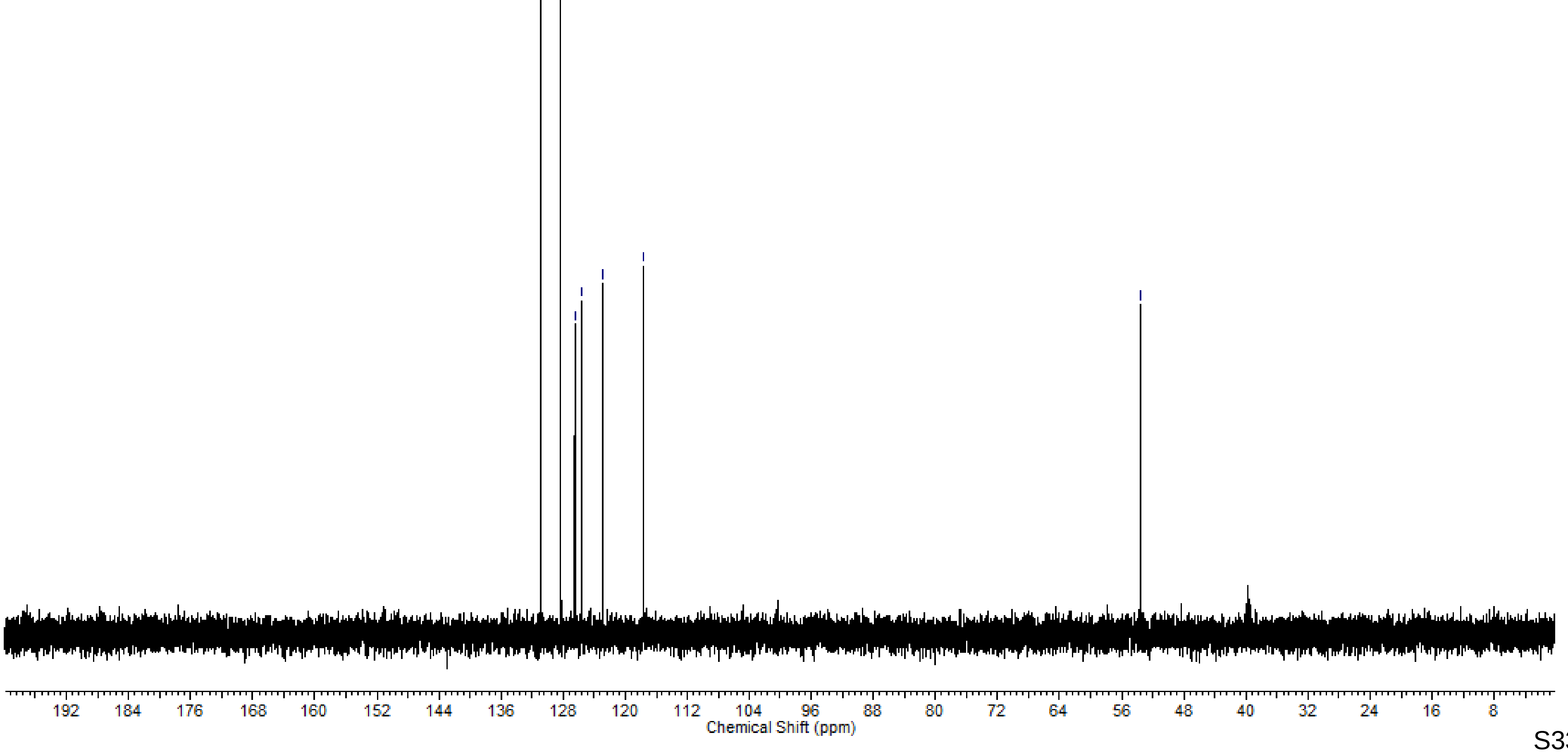

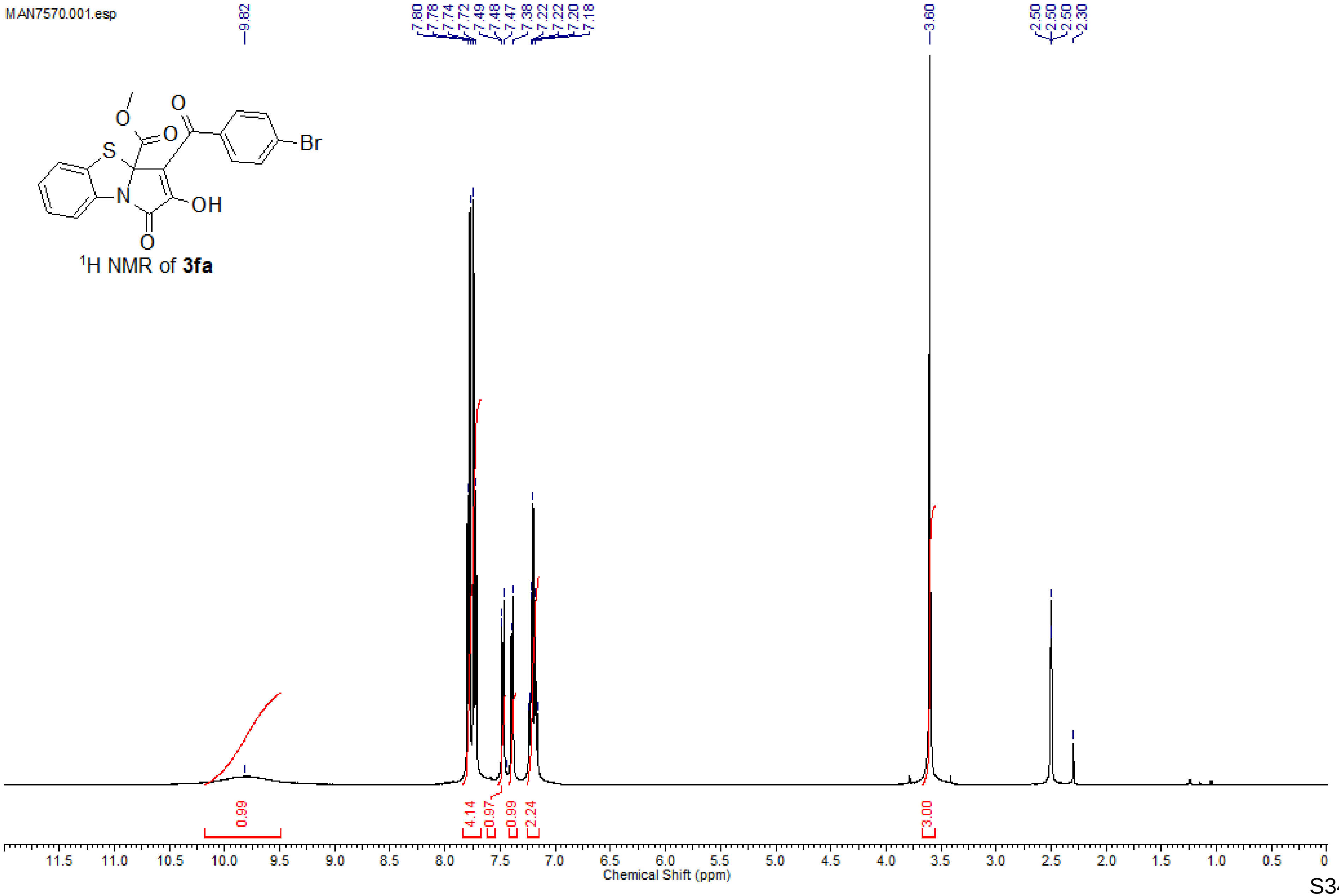
MAN7570.001.esp
9.82
7.80
7.78
7.74
7.72
7.49
7.48
7.47
7.38
7.22
7.22
7.20
7.18
3.60
2.50
2.50
2.50
2.30
S
N
O
O
O
O
OH
O
Br
¹H NMR of 3fa
0.99
4.14
0.97
0.99
2.24
3.00
Chemical Shift (ppm)

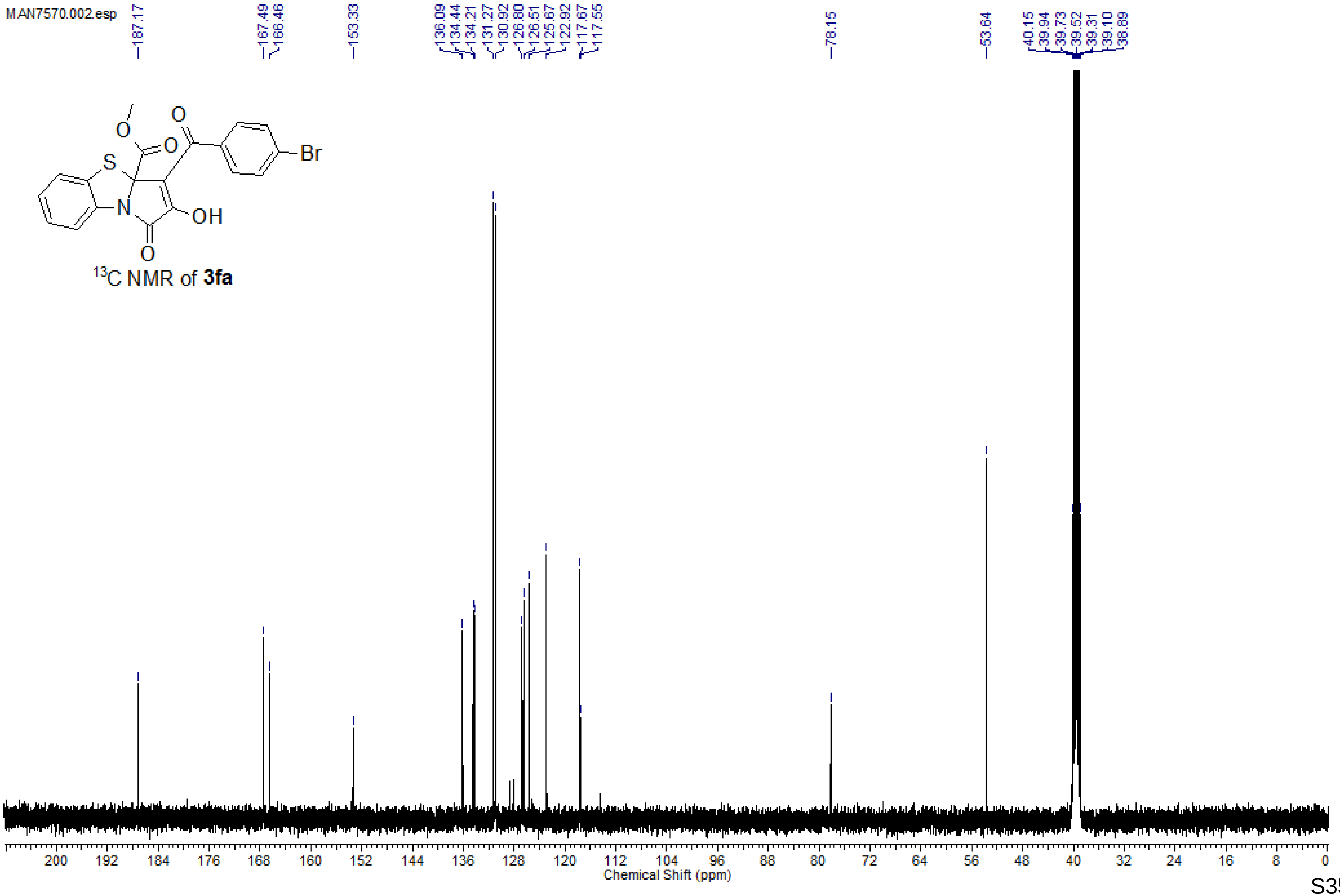

^{13}C NMR of **3fa**

MAN7570.003.esp

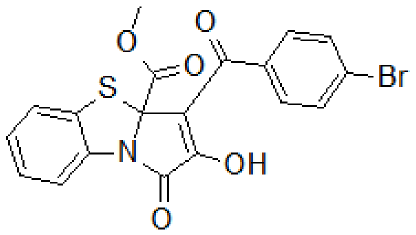

DEPT135 of **3fa**

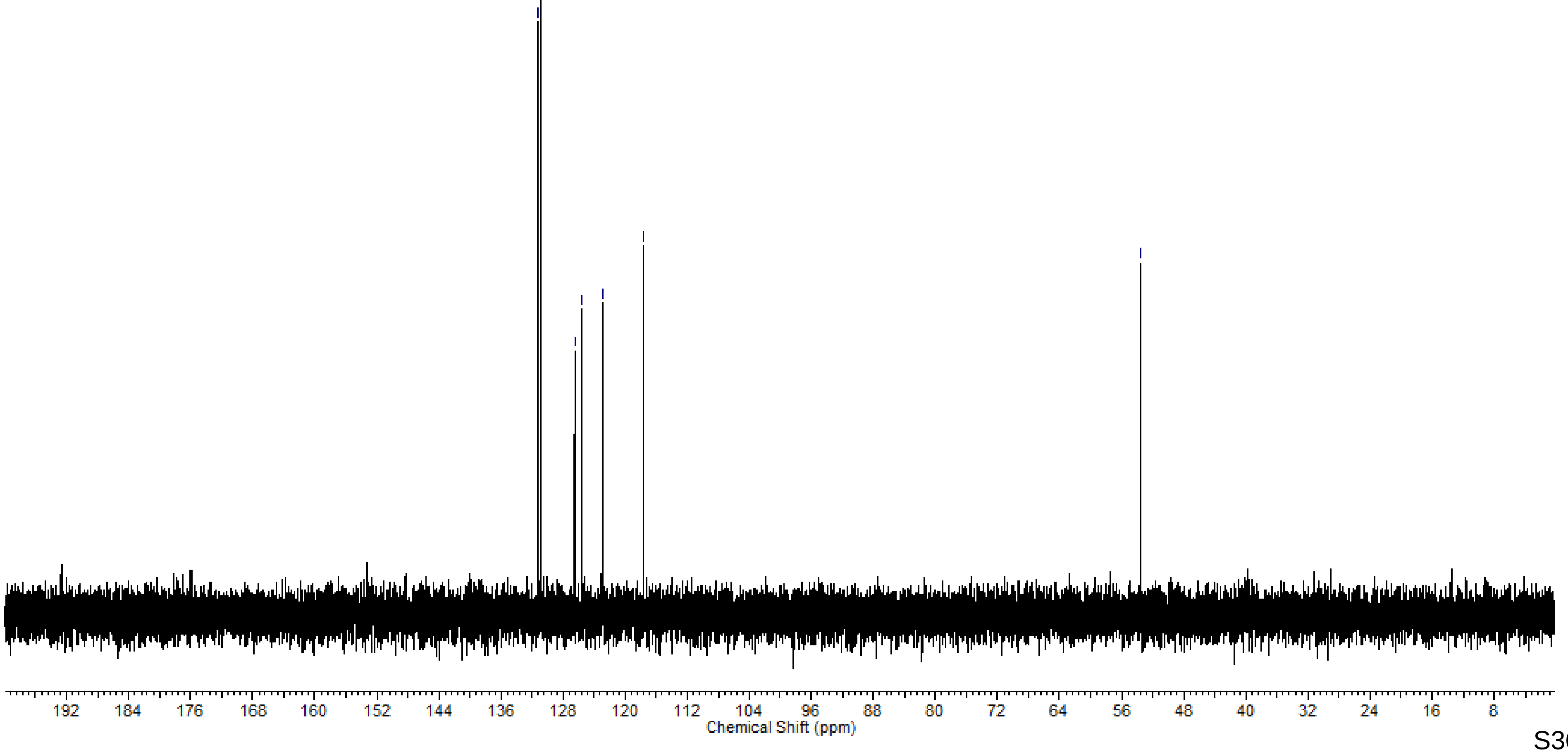

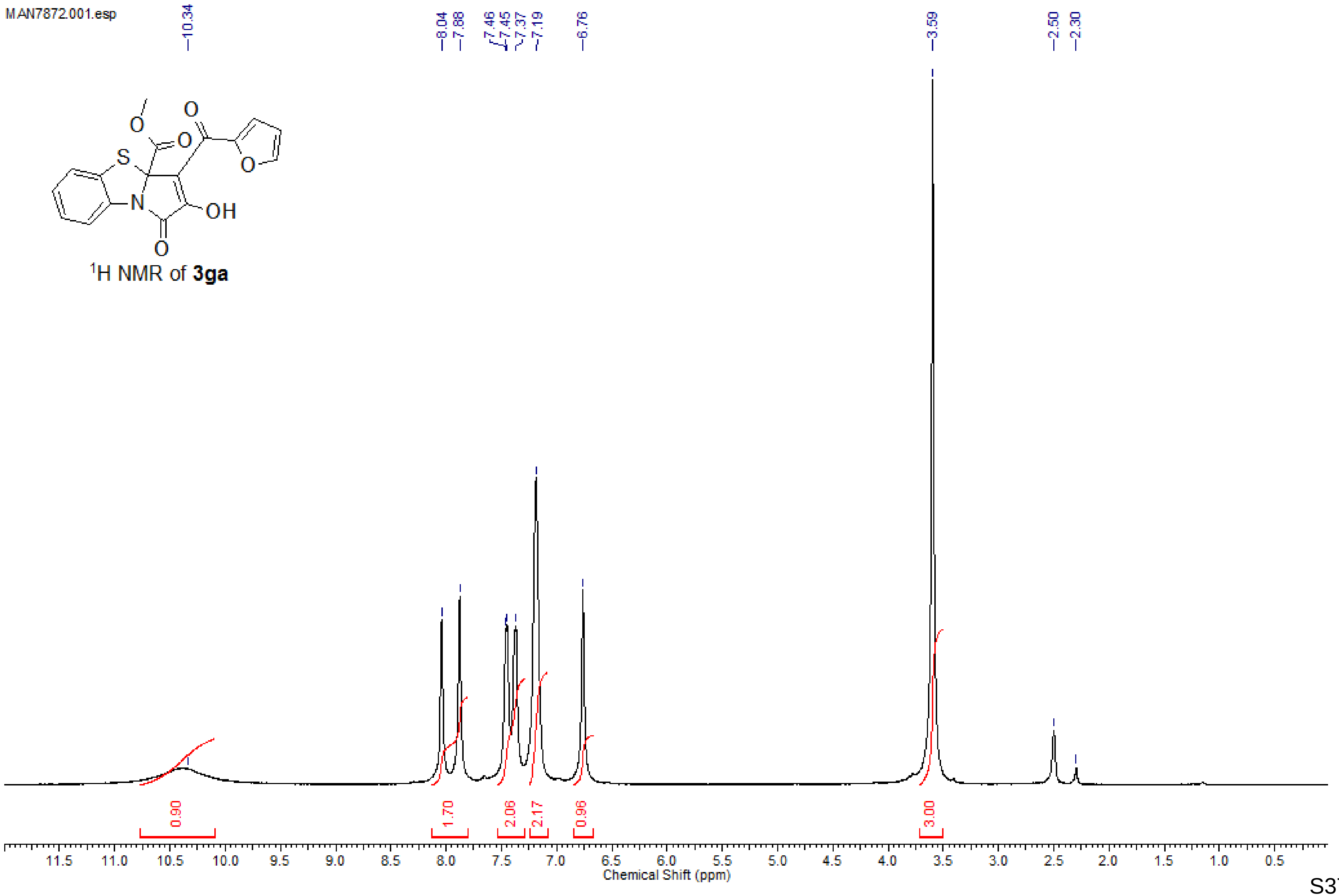
MAN7872.001.esp
10.34
8.04
7.88
7.46
7.45
7.37
7.19
6.76
3.59
2.50
2.30
O
O
O
S
O
N
OH
O
O
¹H NMR of 3ga
0.90
1.70
2.06
2.17
0.96
3.00
11.5
11.0
10.5
10.0
9.5
9.0
8.5
8.0
7.5
7.0
6.5
6.0
5.5
5.0
4.5
4.0
3.5
3.0
2.5
2.0
1.5
1.0
0.5
Chemical Shift (ppm)

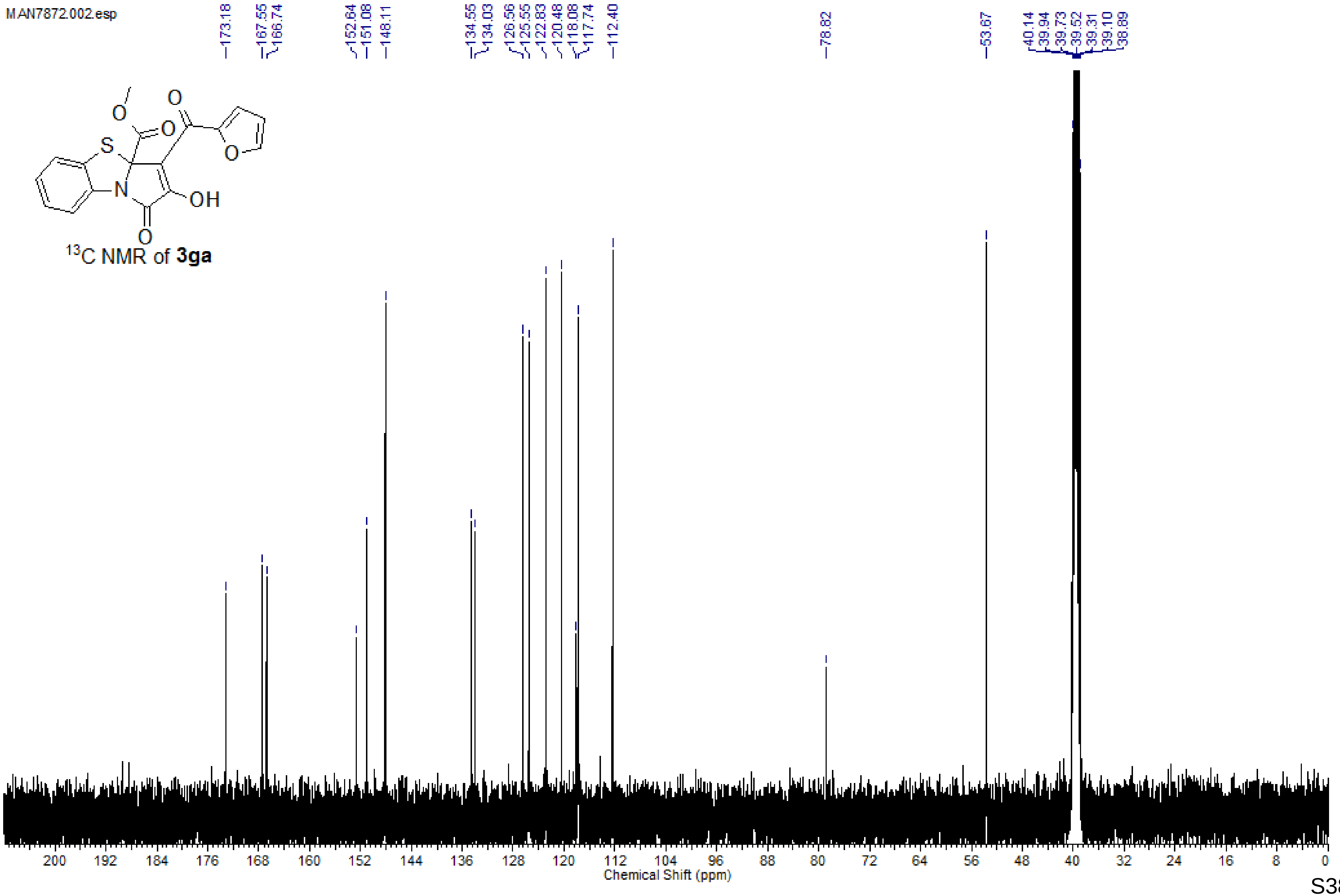
MAN7872.002.esp
173.18
167.55
166.74
152.64
151.08
148.11
134.55
134.03
126.56
125.55
122.83
120.48
118.08
117.74
112.40
78.82
53.67
40.14
39.94
39.73
39.52
39.31
39.10
38.89
O
O
S
O
O
N
OH
O
O
^{13}C NMR of **3ga**
200 192 184 176 168 160 152 144 136 128 120 112 104 96 88 80 72 64 56 48 40 32 24 16 8 0
Chemical Shift (ppm)

MAN7872.003.esp

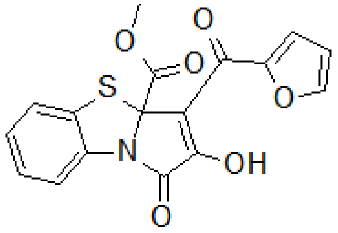

DEPT135 of **3ga**

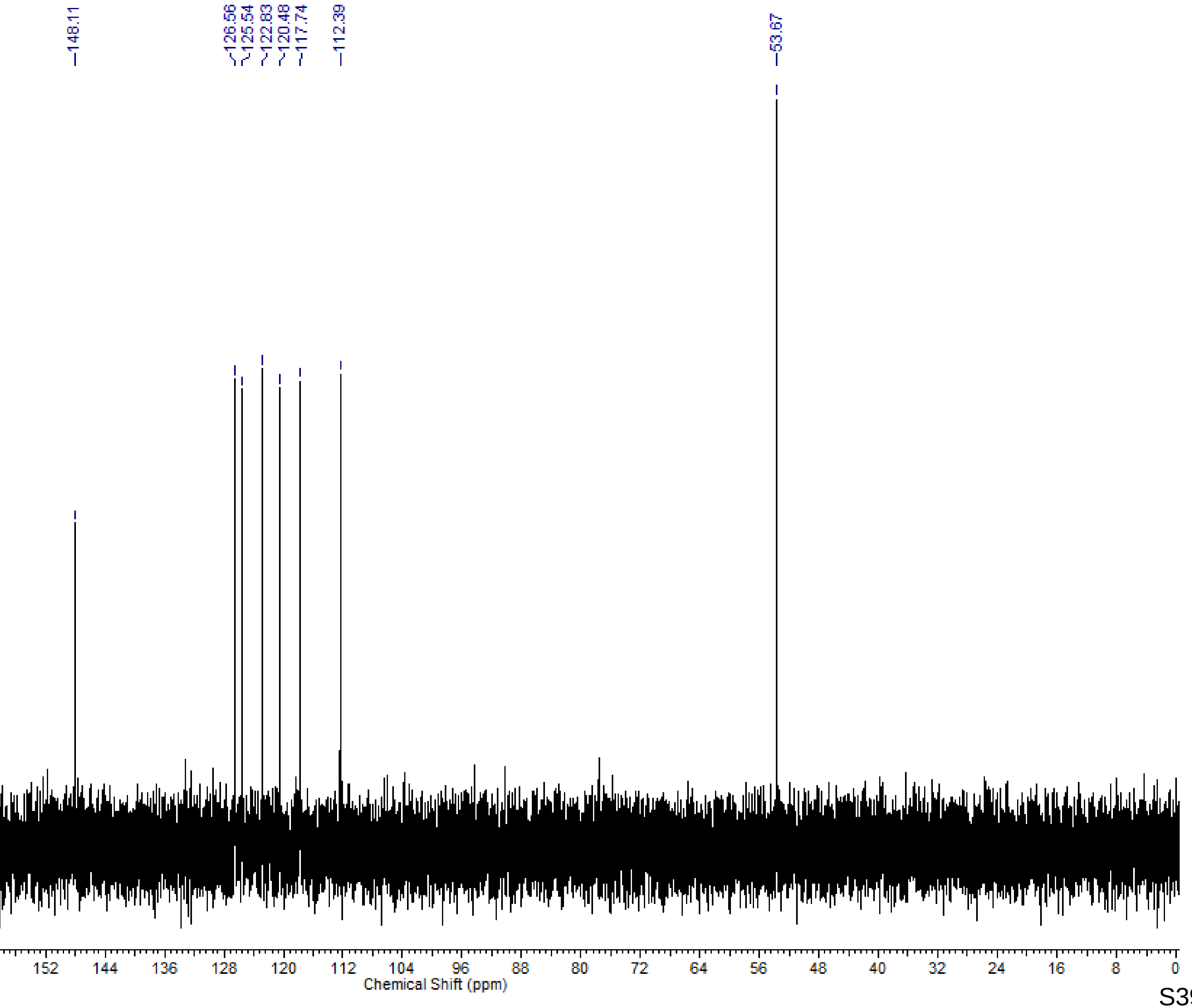

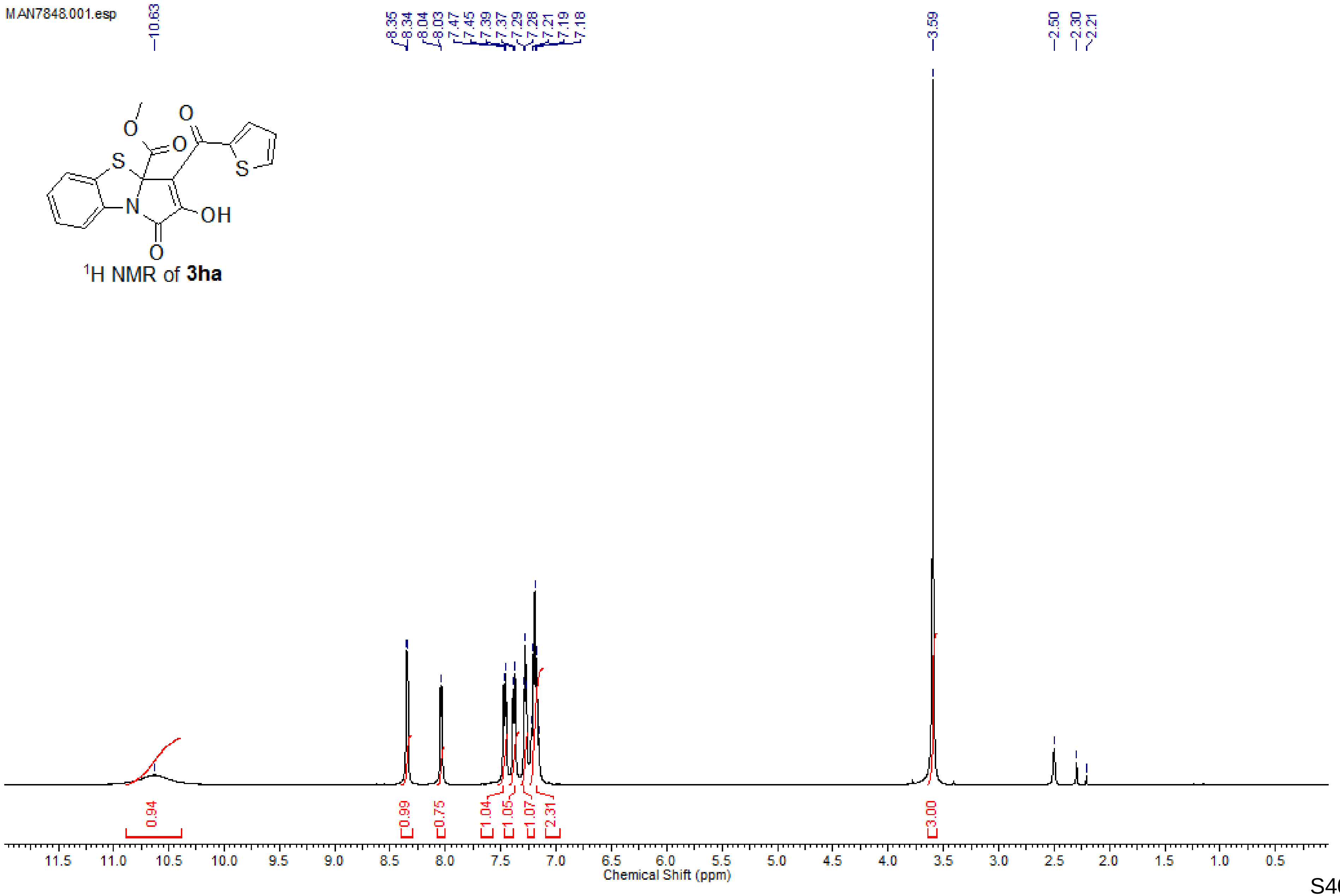
MAN7848.001.esp
10.63
8.35
8.34
8.04
8.03
7.47
7.45
7.39
7.37
7.29
7.28
7.21
7.19
7.18
3.59
2.50
2.30
2.21
O
O
O
S
S
N
OH
O
0.94
0.99
0.75
1.04
1.05
1.07
2.31
3.00
11.5
11.0
10.5
10.0
9.5
9.0
8.5
8.0
7.5
7.0
6.5
6.0
5.5
5.0
4.5
4.0
3.5
3.0
2.5
2.0
1.5
1.0
0.5
Chemical Shift (ppm)

^{1}H NMR of **3ha**

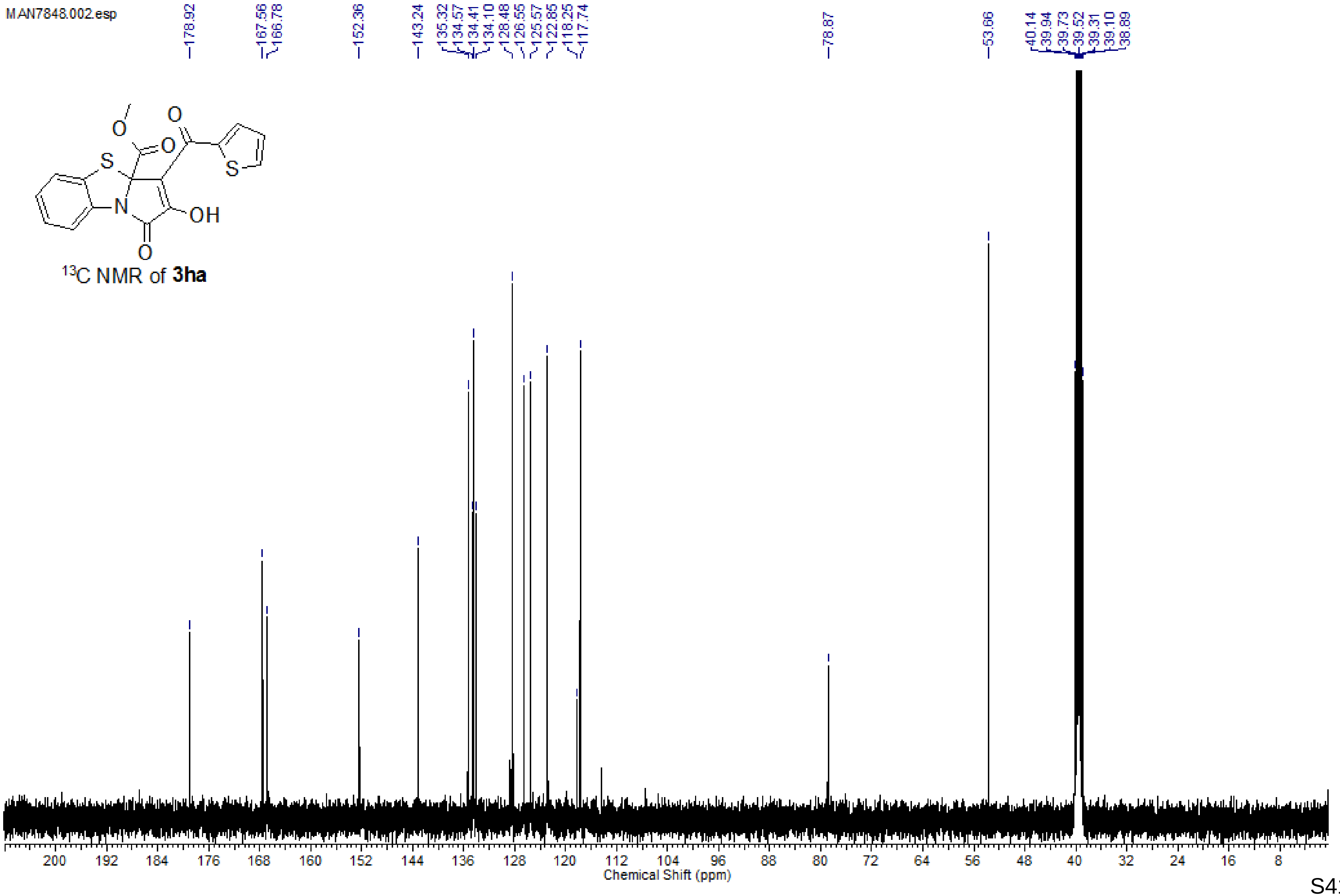

^{13}C NMR of **3ha**

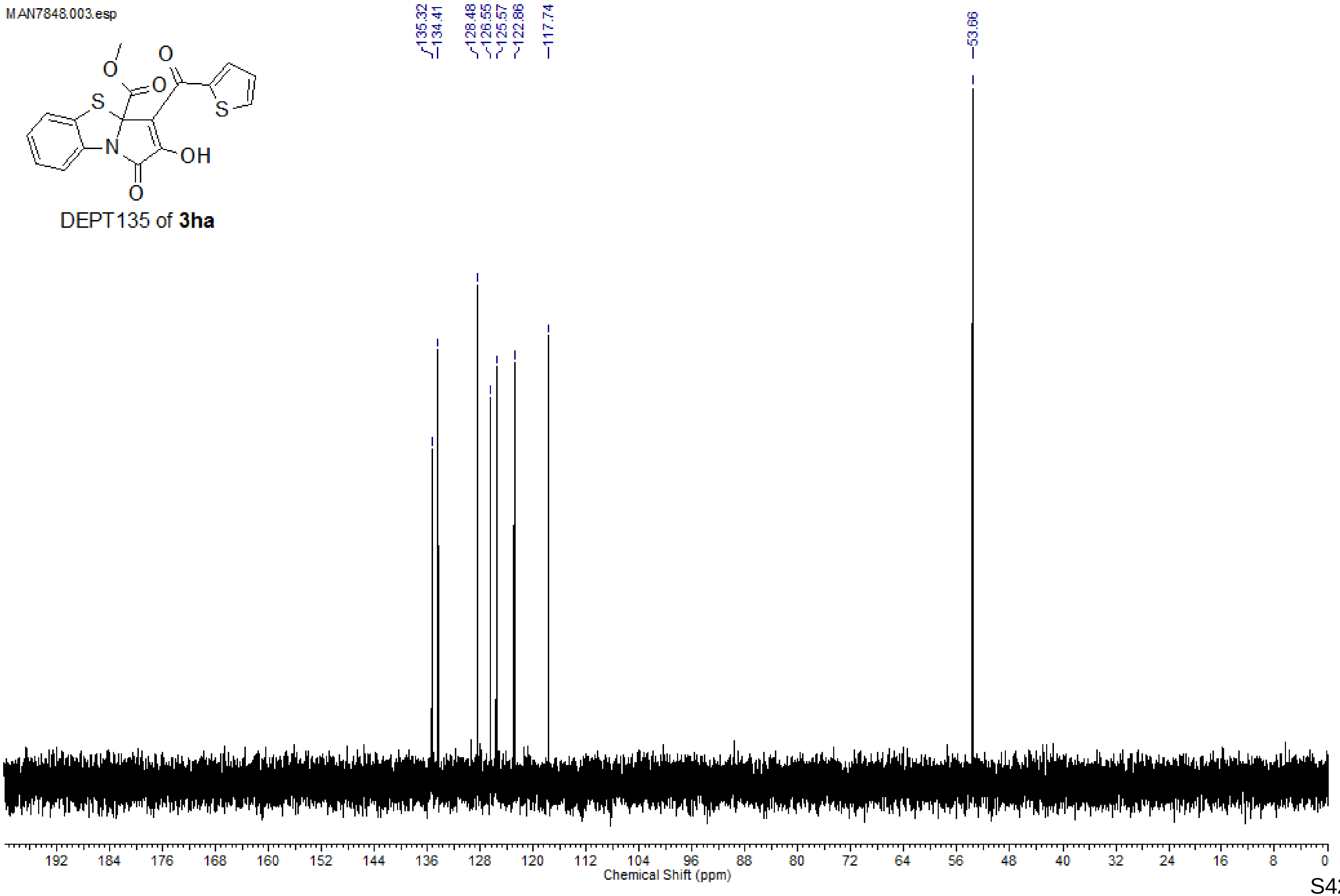

MAN7848.003.esp
O
O
O
S
S
N
OH
O
DEPT135 of **3ha**
135.32
134.41
128.48
126.55
125.57
122.86
117.74
53.66
192
184
176
168
160
152
144
136
128
120
112
104
96
88
80
72
64
56
48
40
32
24
16
8
0
Chemical Shift (ppm)

MAN7326.001.esp

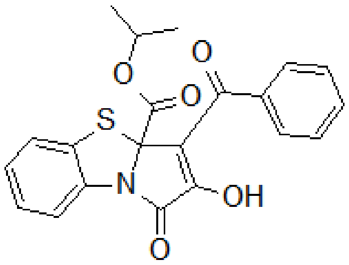

^{1}H NMR of **3ab**

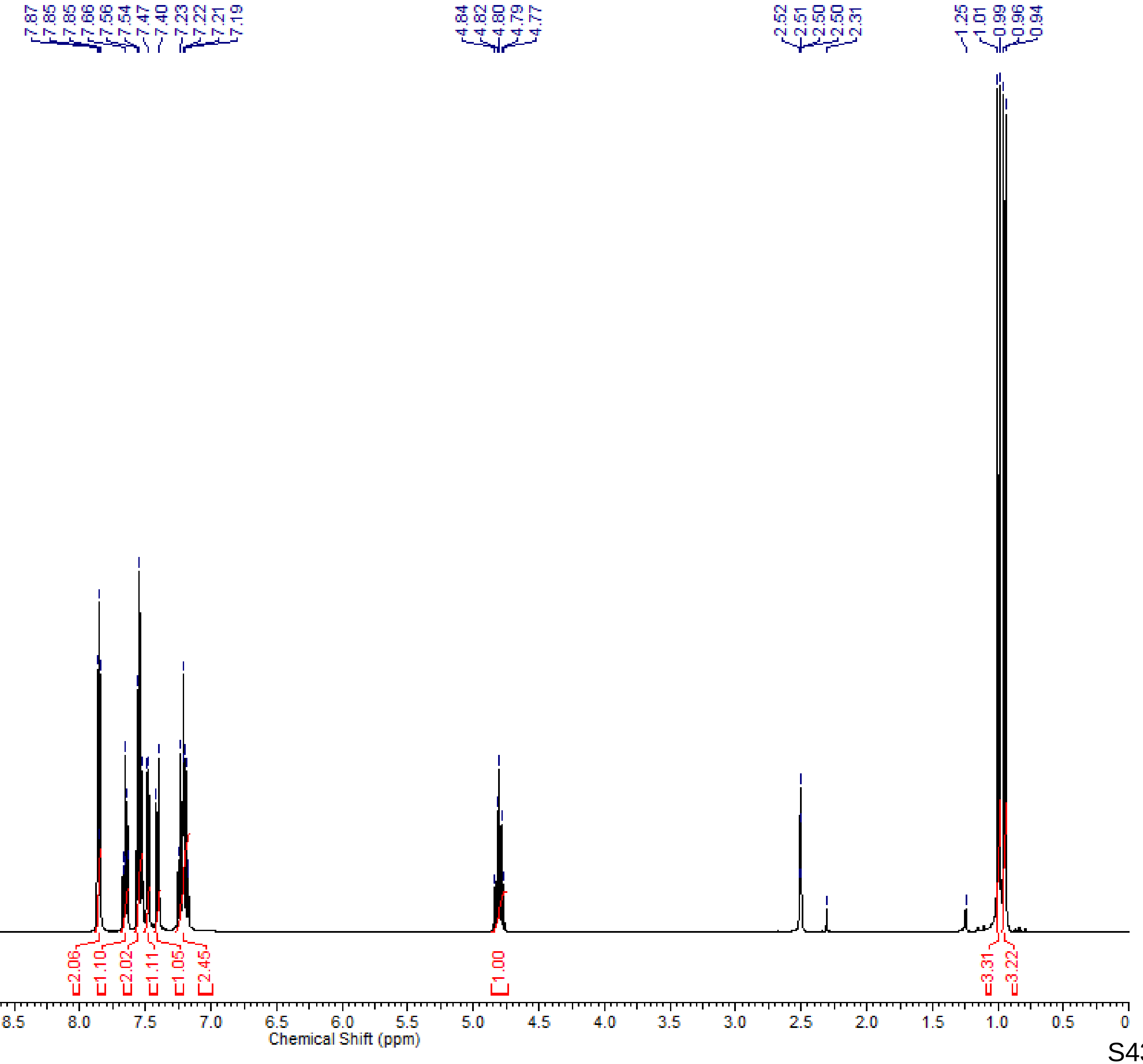

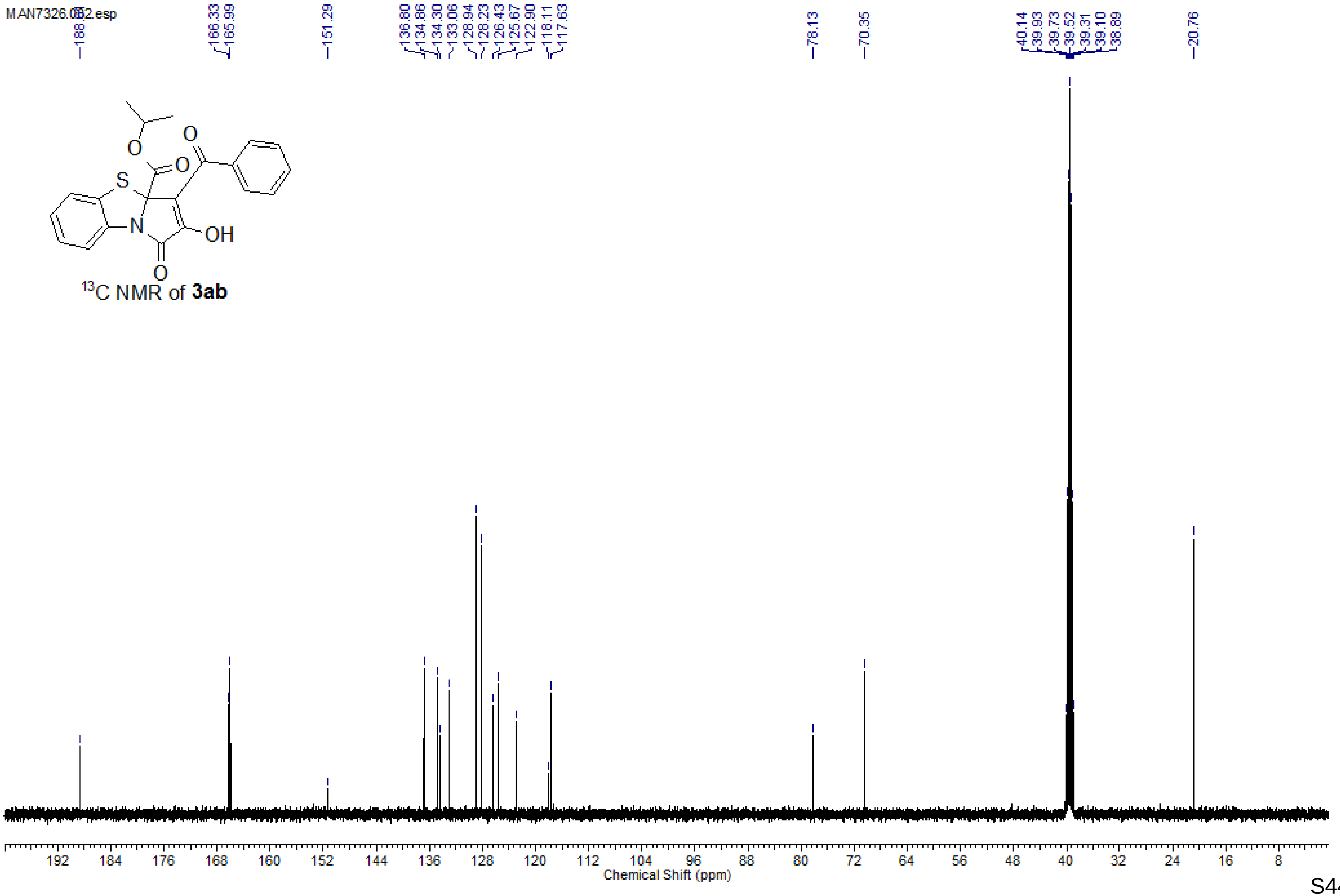
166.33
165.99
151.29
136.80
134.86
134.30
133.06
128.94
128.23
126.43
125.67
122.90
118.11
117.63
78.13
70.35
40.14
39.93
39.73
39.52
39.31
39.10
38.89
20.76
O
O
O
S
N
OH
O
^{13}C NMR of **3ab**
192 184 176 168 160 152 144 136 128 120 112 104 96 88 80 72 64 56 48 40 32 24 16 8
Chemical Shift (ppm)

MAN7326.003.esp

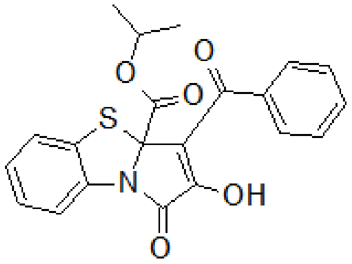

DEPT135 of **3ab**

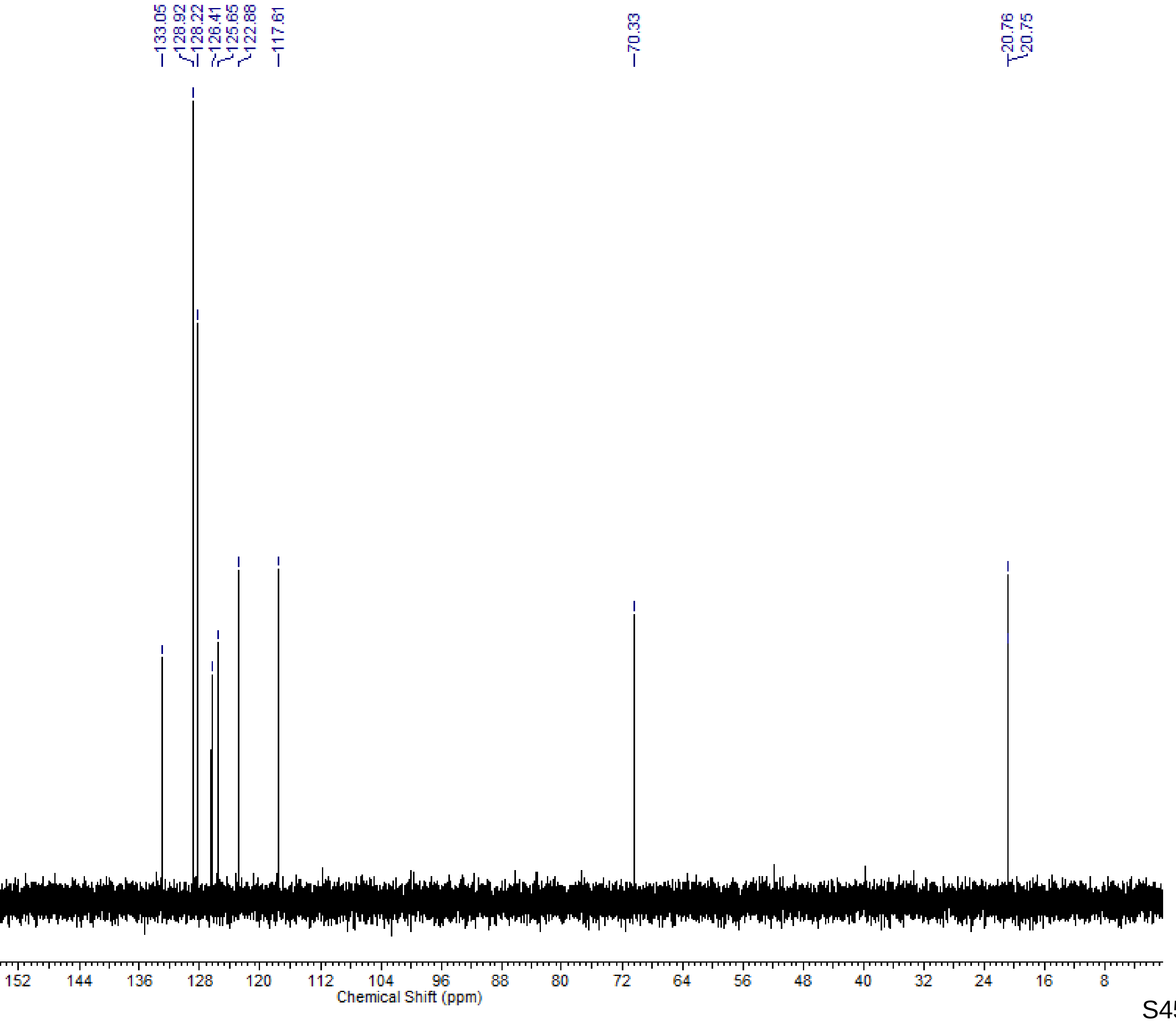

MAN7327.001.esp

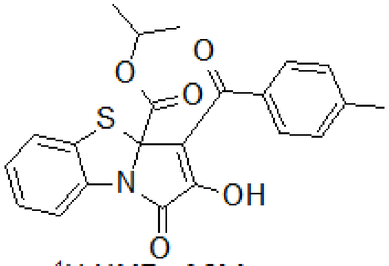

^1H NMR of **3bb**

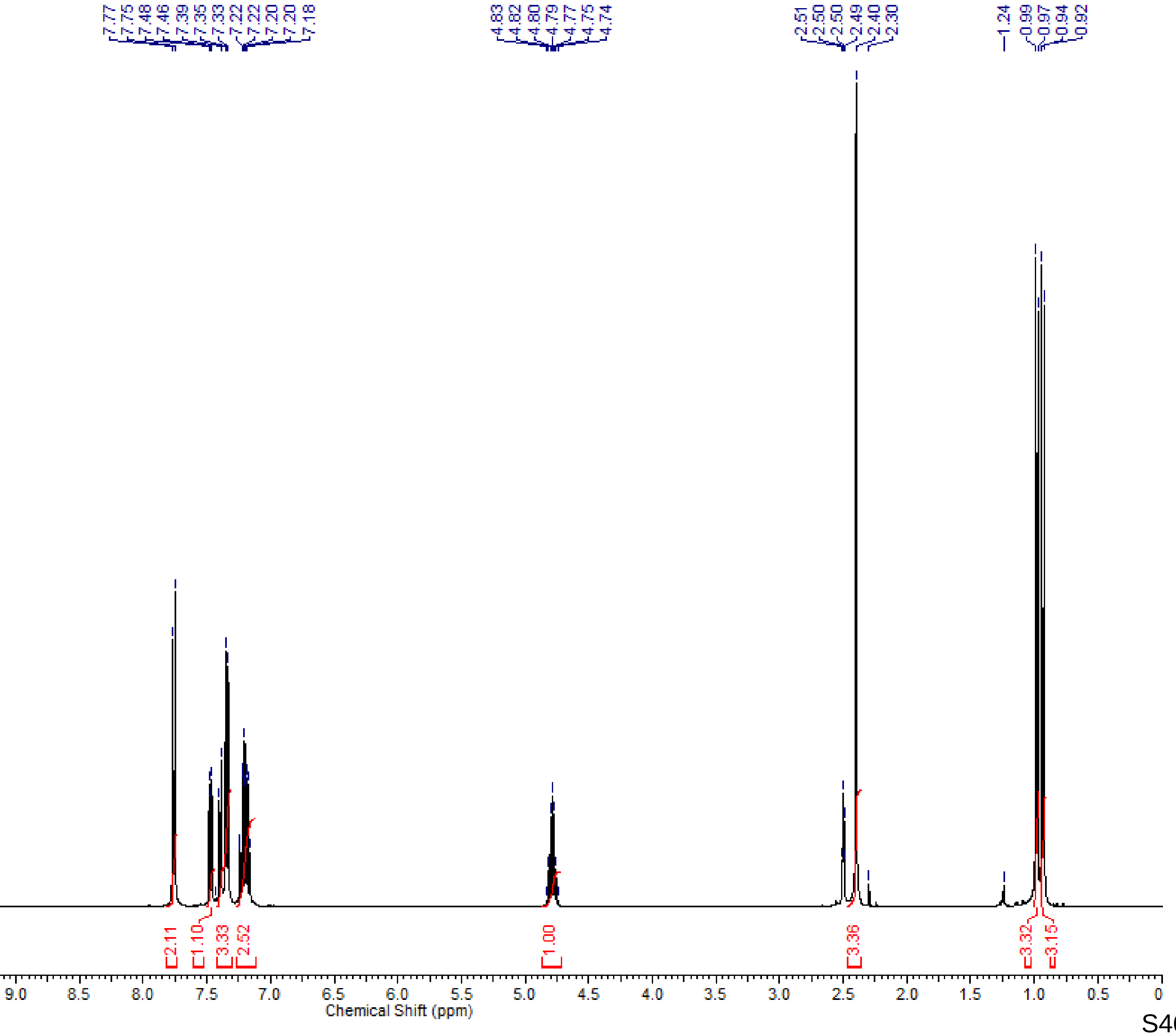

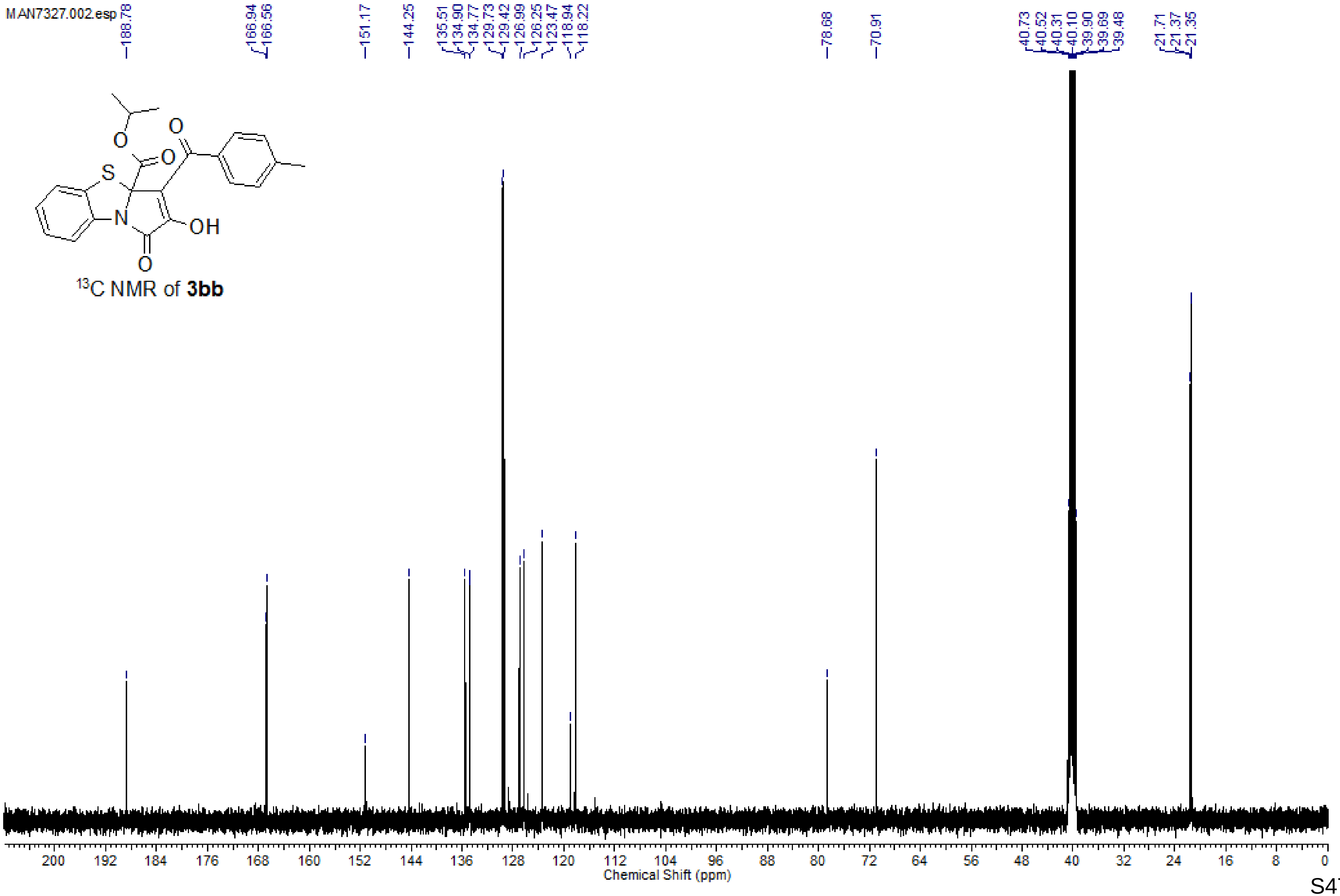

^{13}C NMR of **3bb**

MAN7327.003.esp

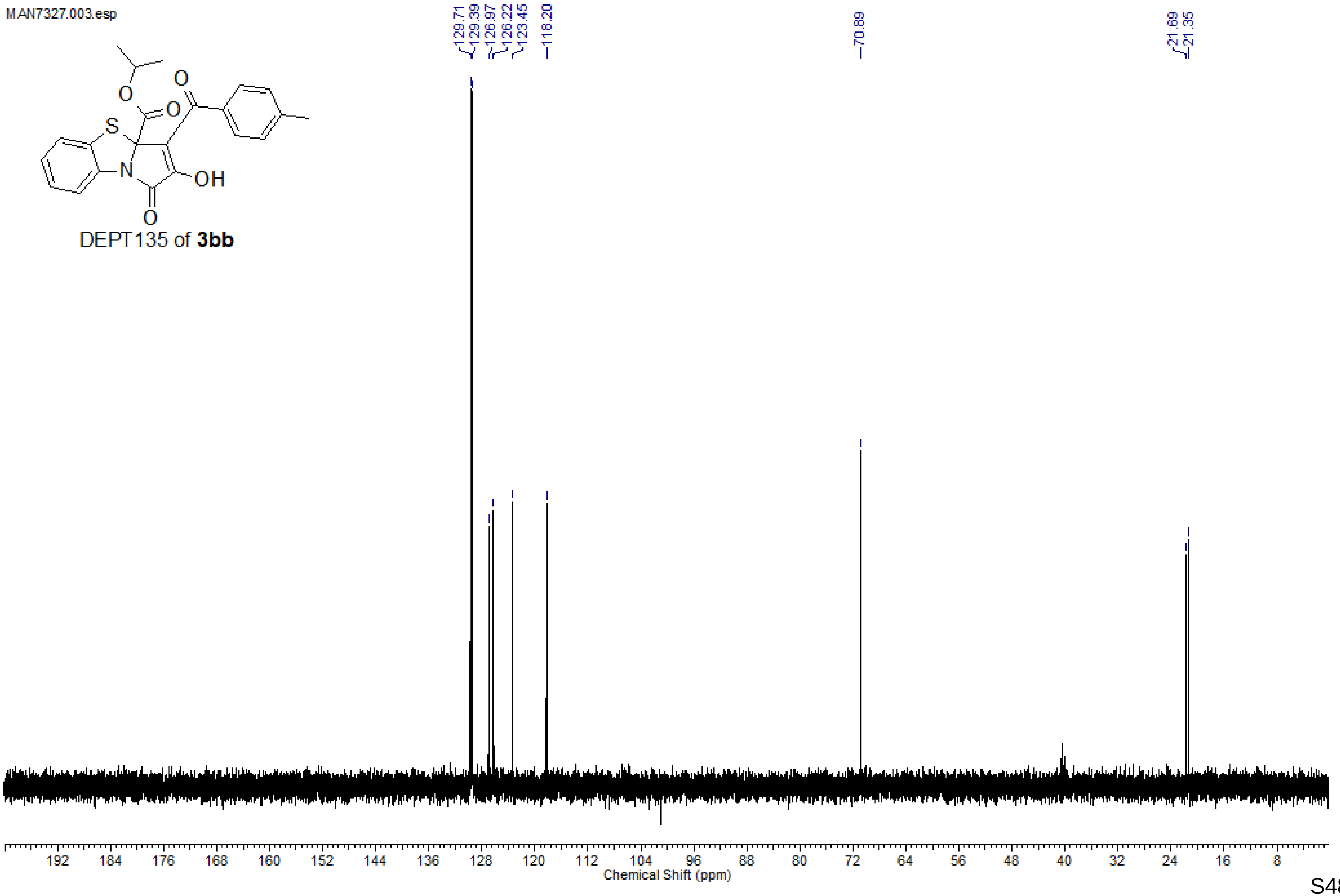

DEPT135 of **3bb**

MAN7809.001.esp

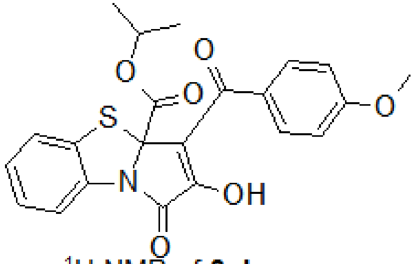

^{1}H NMR of **3cb**

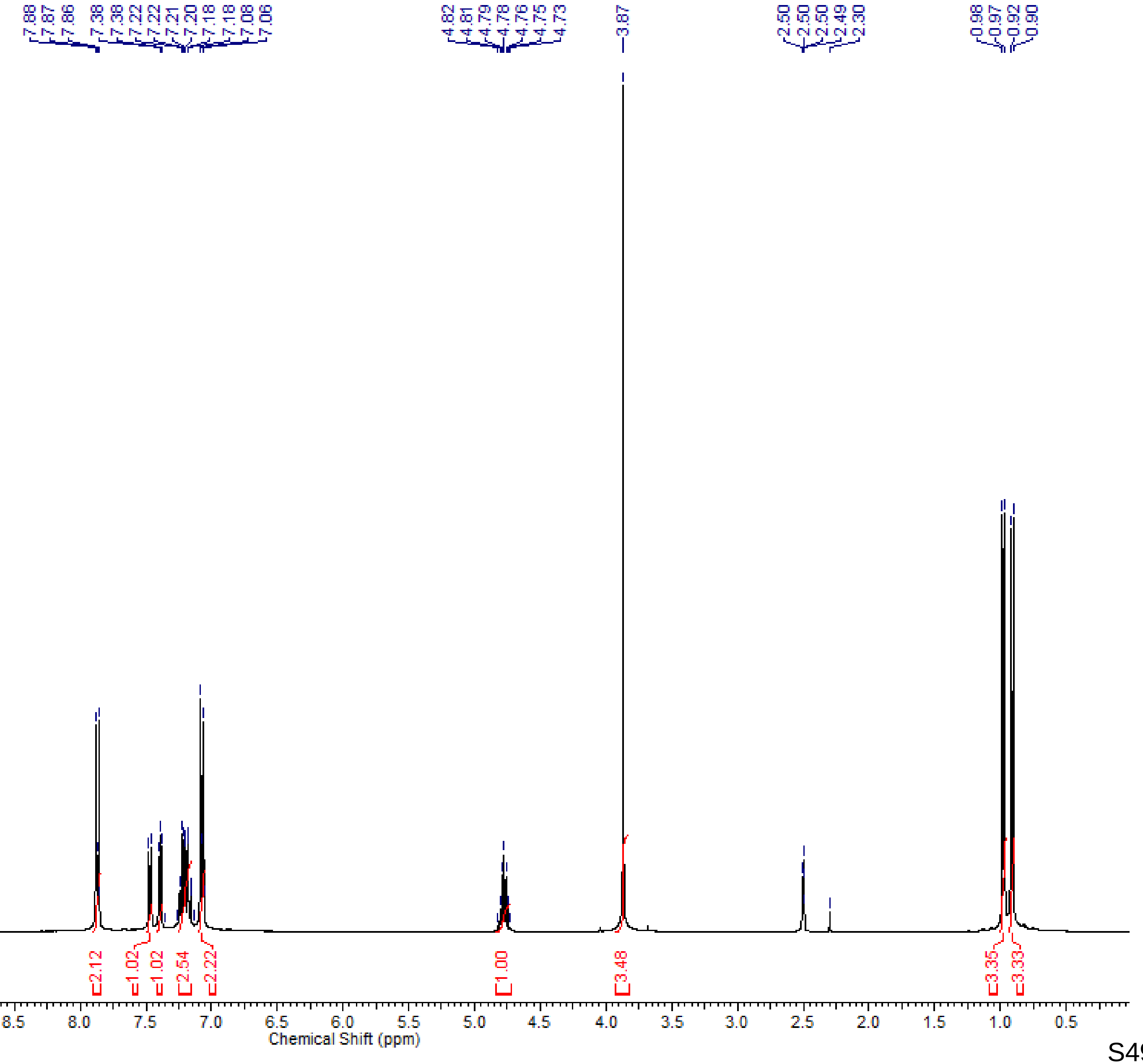

MAN7809.002.esp

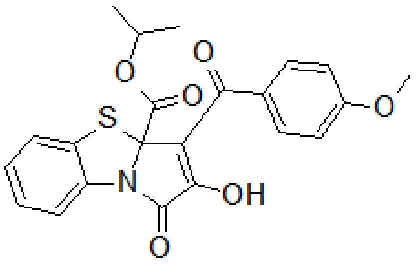

^{13}C NMR of **3cb**

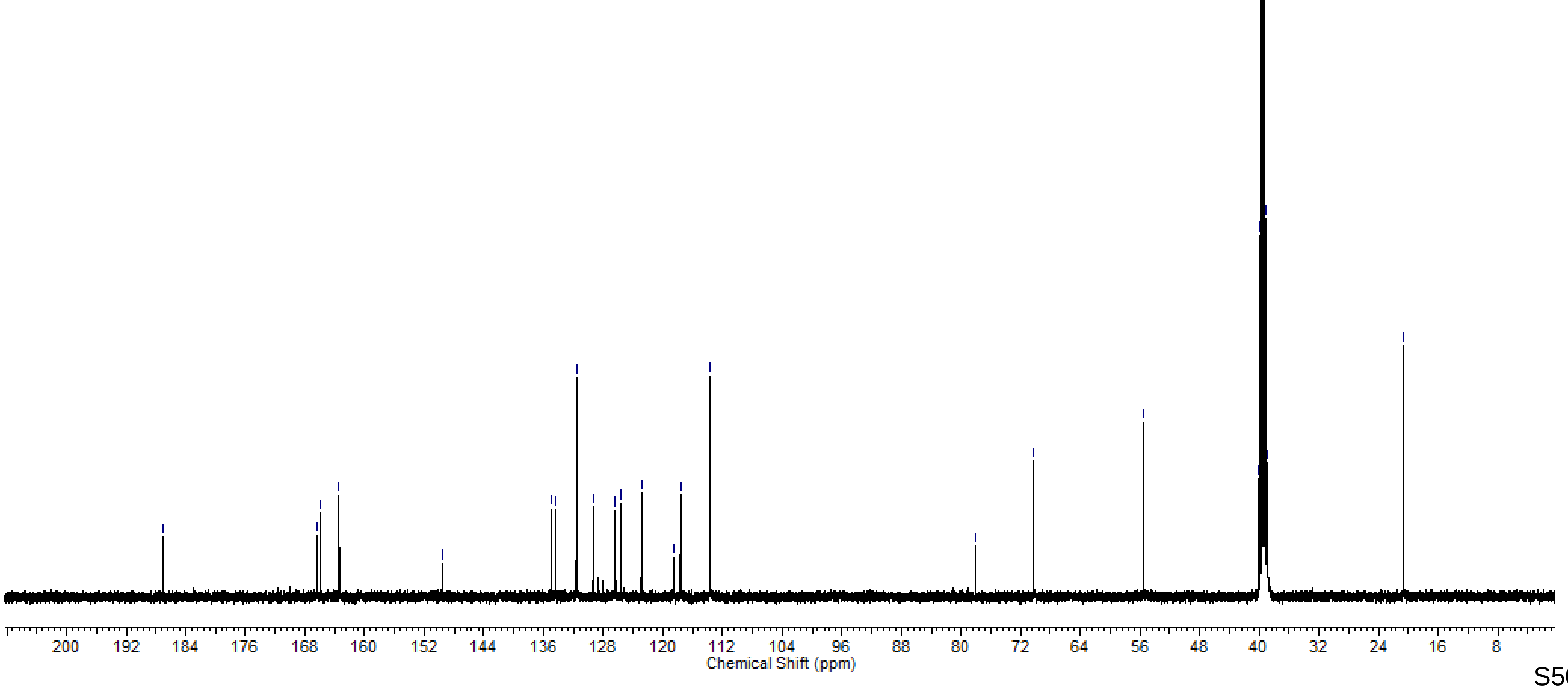

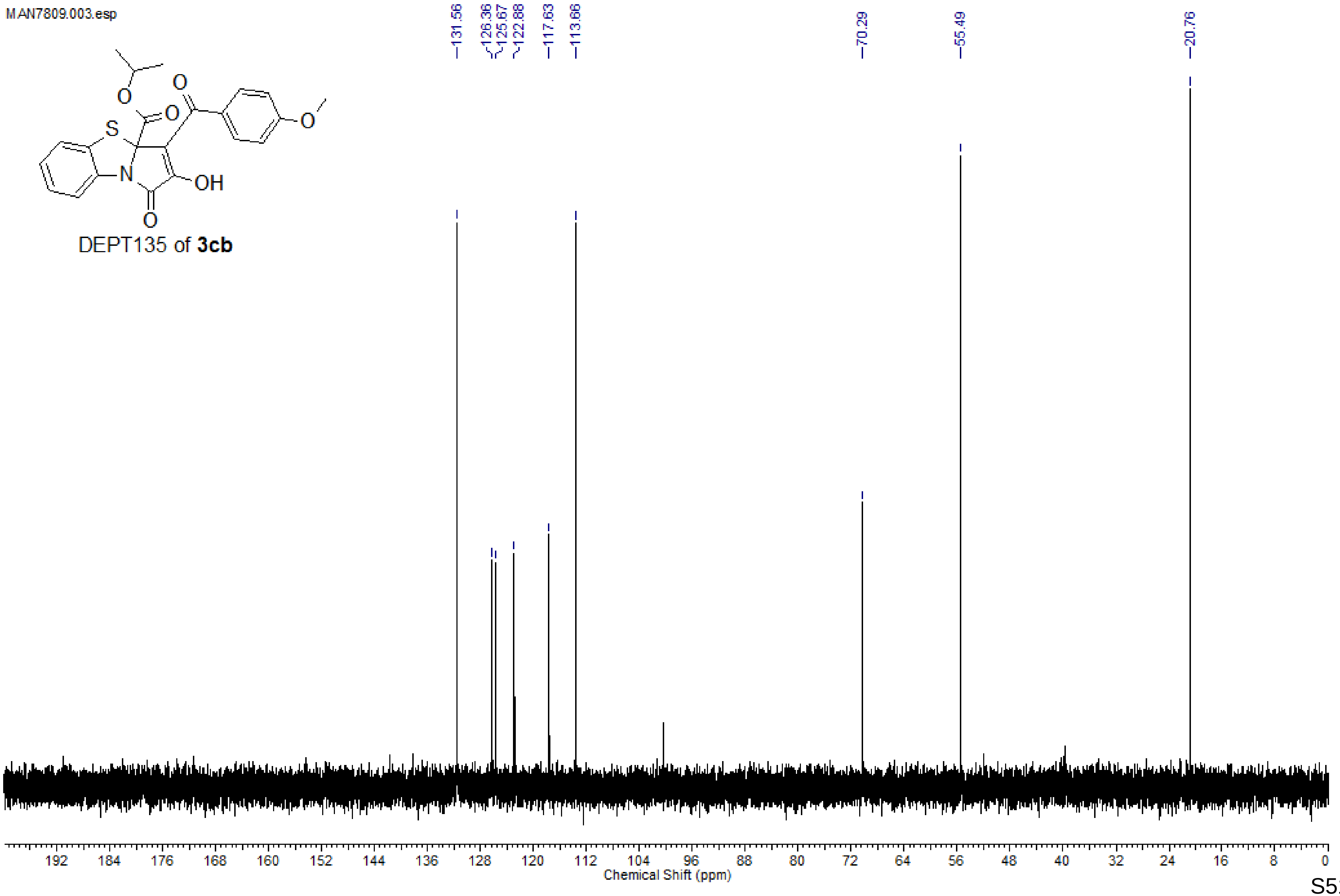

DEPT135 of **3cb**

MAN7381.001.esp

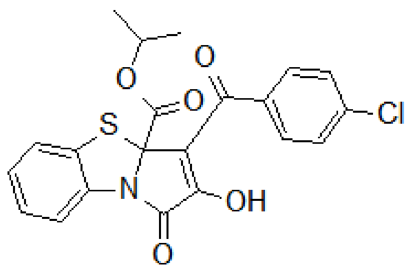

^{1}H NMR of **3eb**

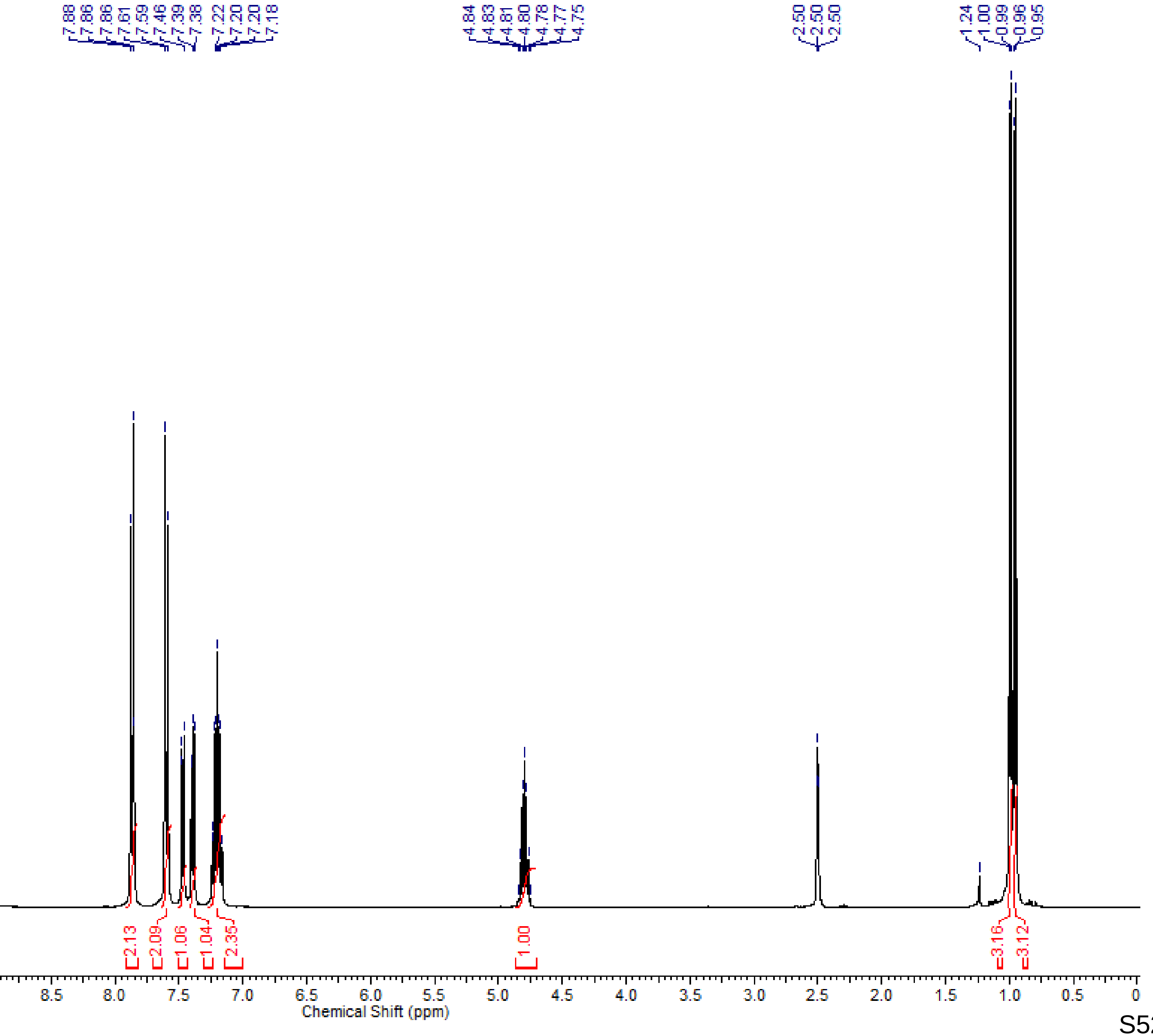

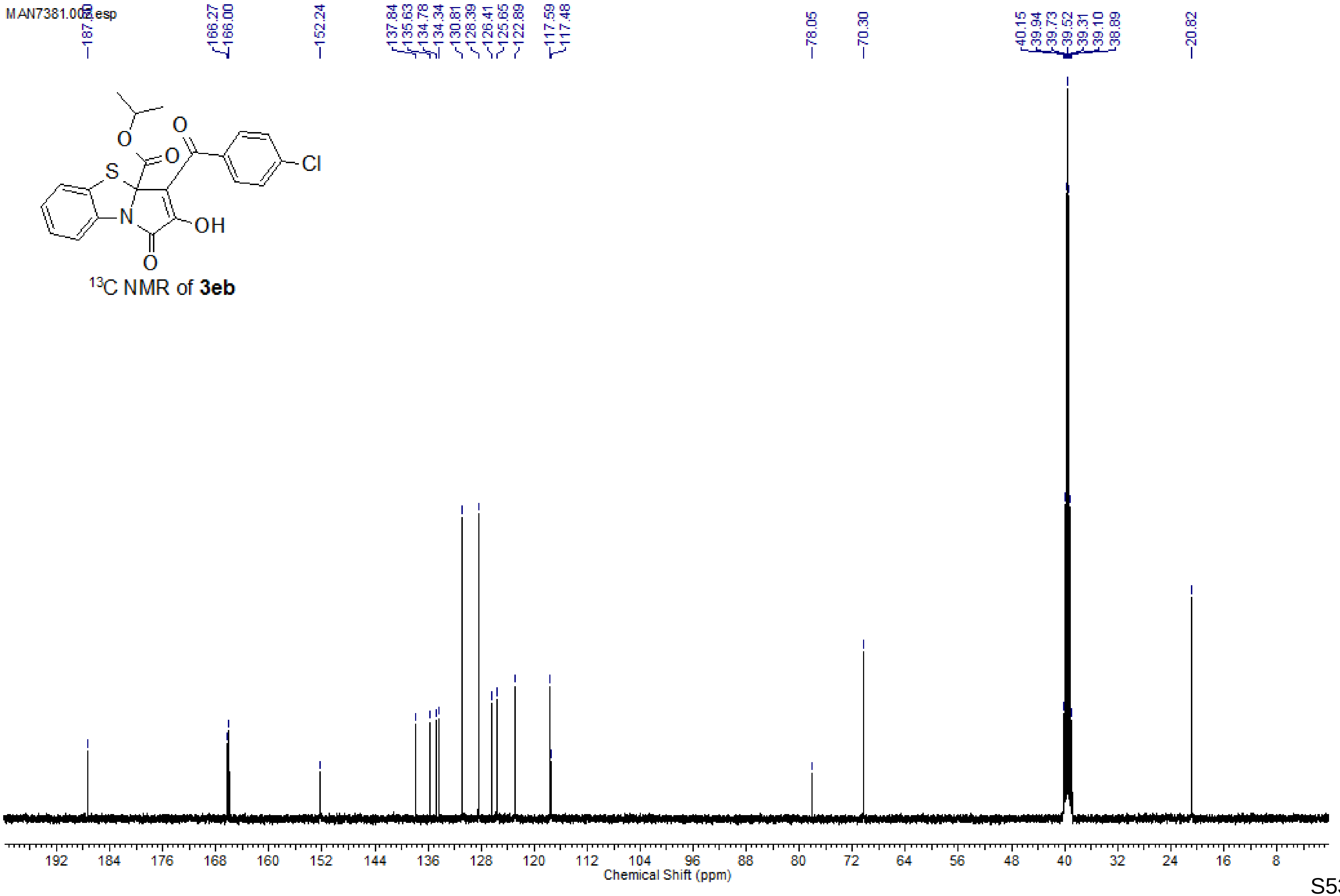

^{13}C NMR of **3eb**

MAN7381.003.esp

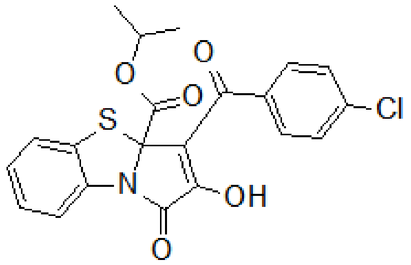

DEPT135 of **3eb**

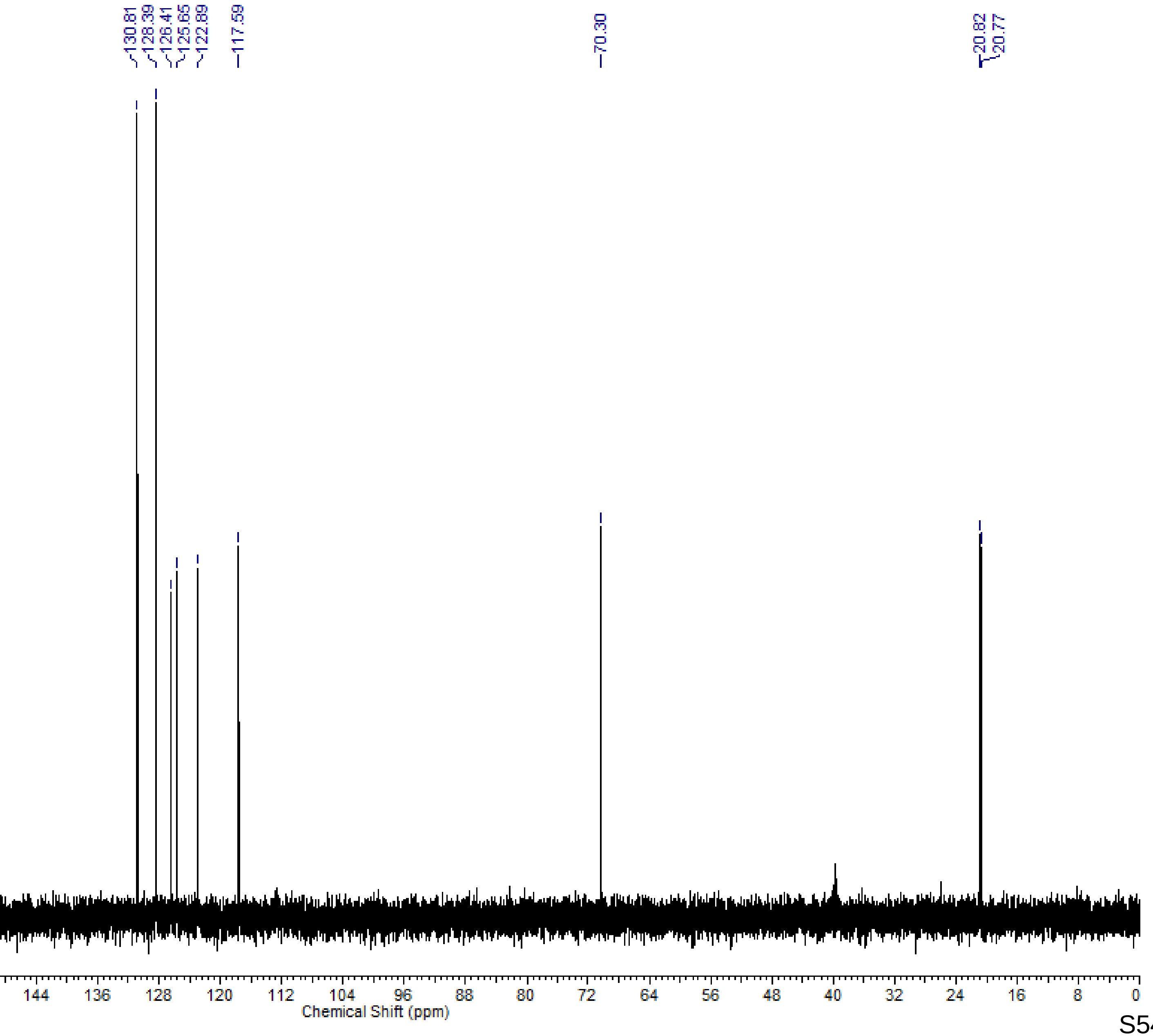

MAN7612.001.esp

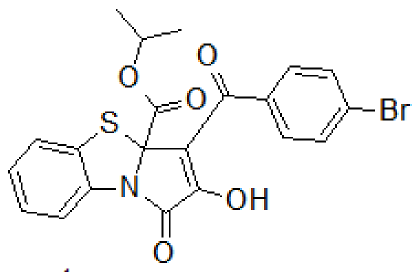

^{1}H NMR of **3fb**

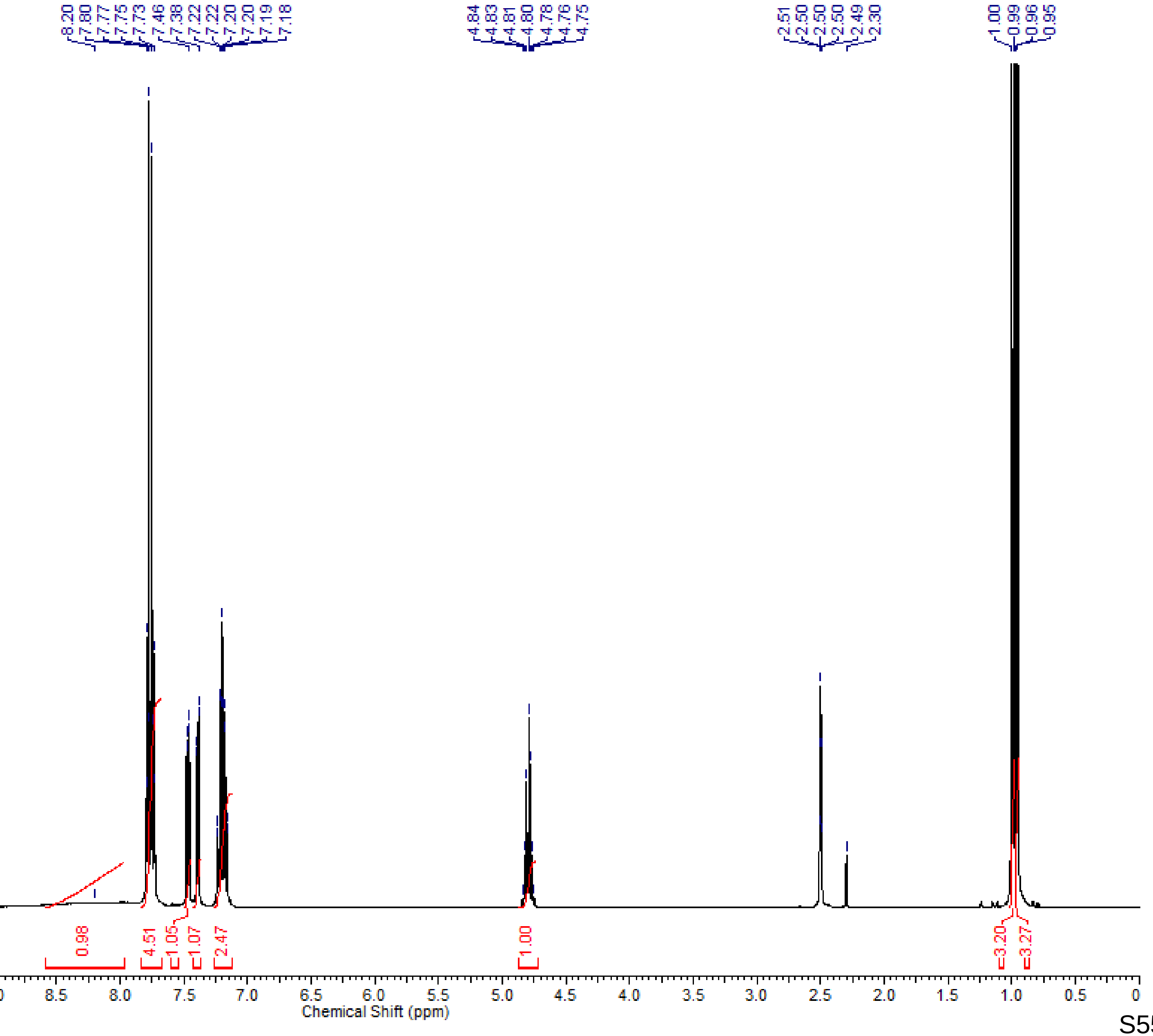

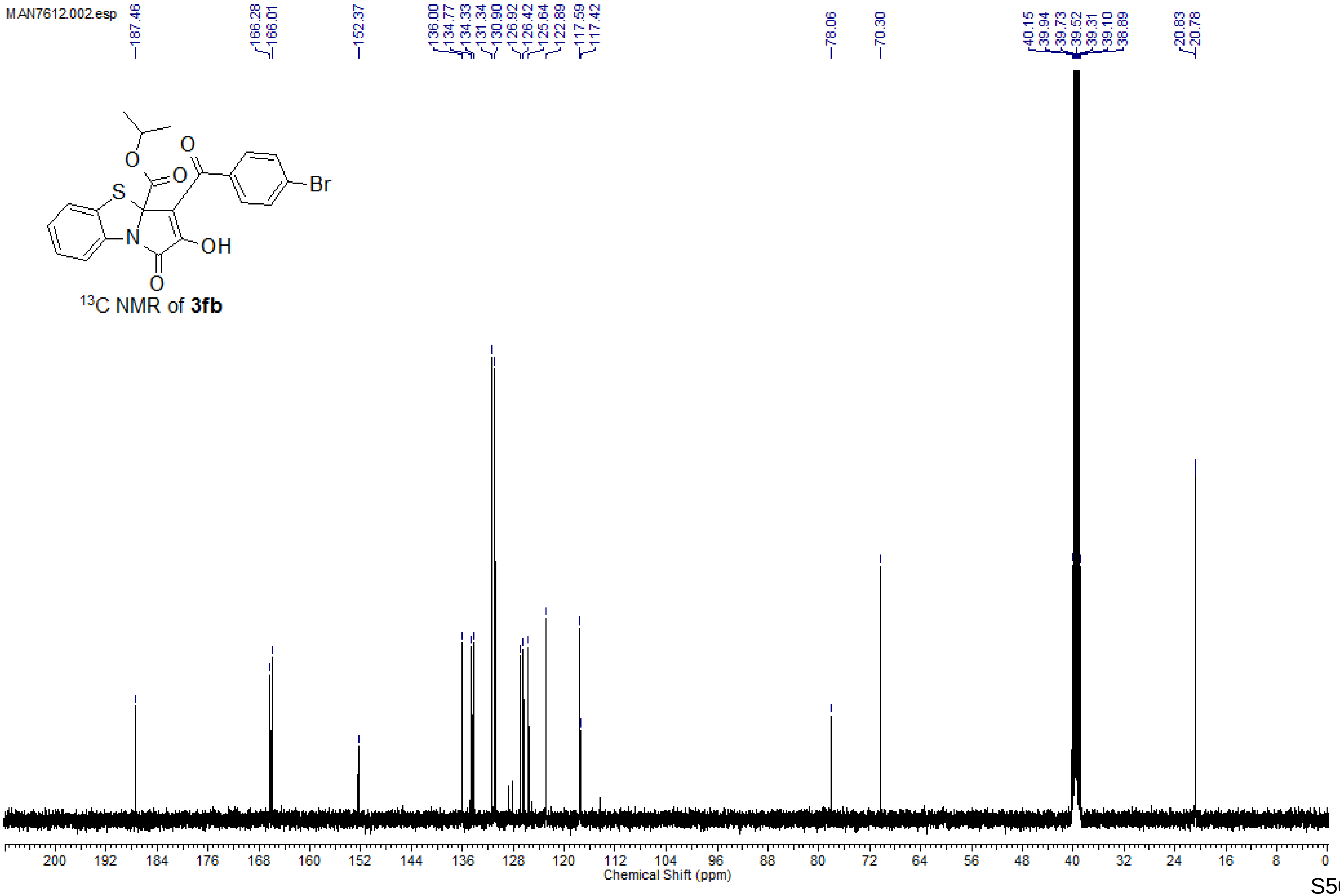

^{13}C NMR of **3fb**

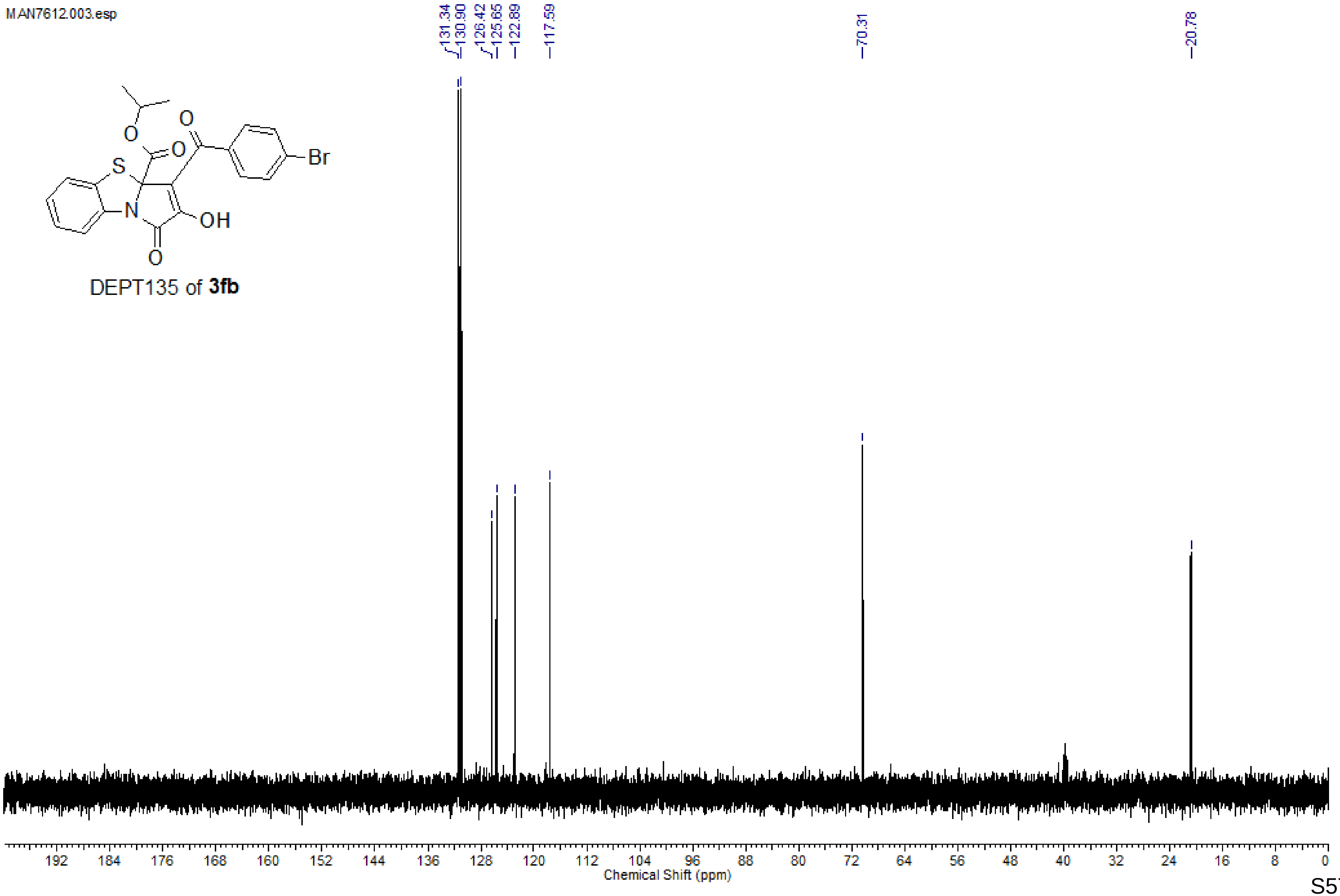

DEPT135 of **3fb**

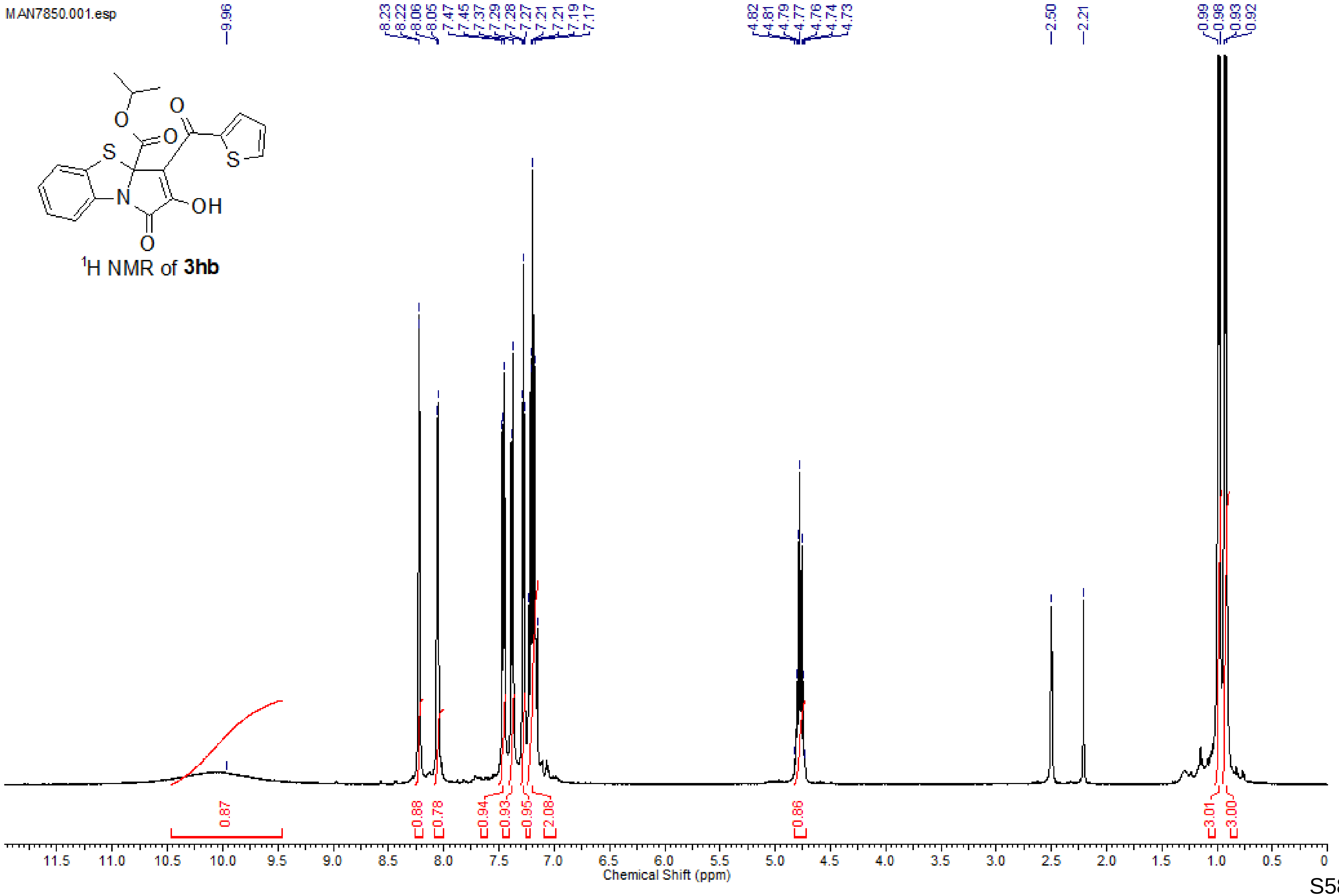
MAN7850.001.esp
9.96
8.23
8.22
8.06
8.05
7.47
7.45
7.37
7.29
7.28
7.27
7.21
7.21
7.19
7.17
4.82
4.81
4.79
4.77
4.76
4.74
4.73
2.50
2.21
0.99
0.98
0.93
0.92
OH
0.87
0.88
0.78
0.94
0.93
0.95
2.08
0.86
3.01
3.00
Chemical Shift (ppm)

^{1}H NMR of **3hb**

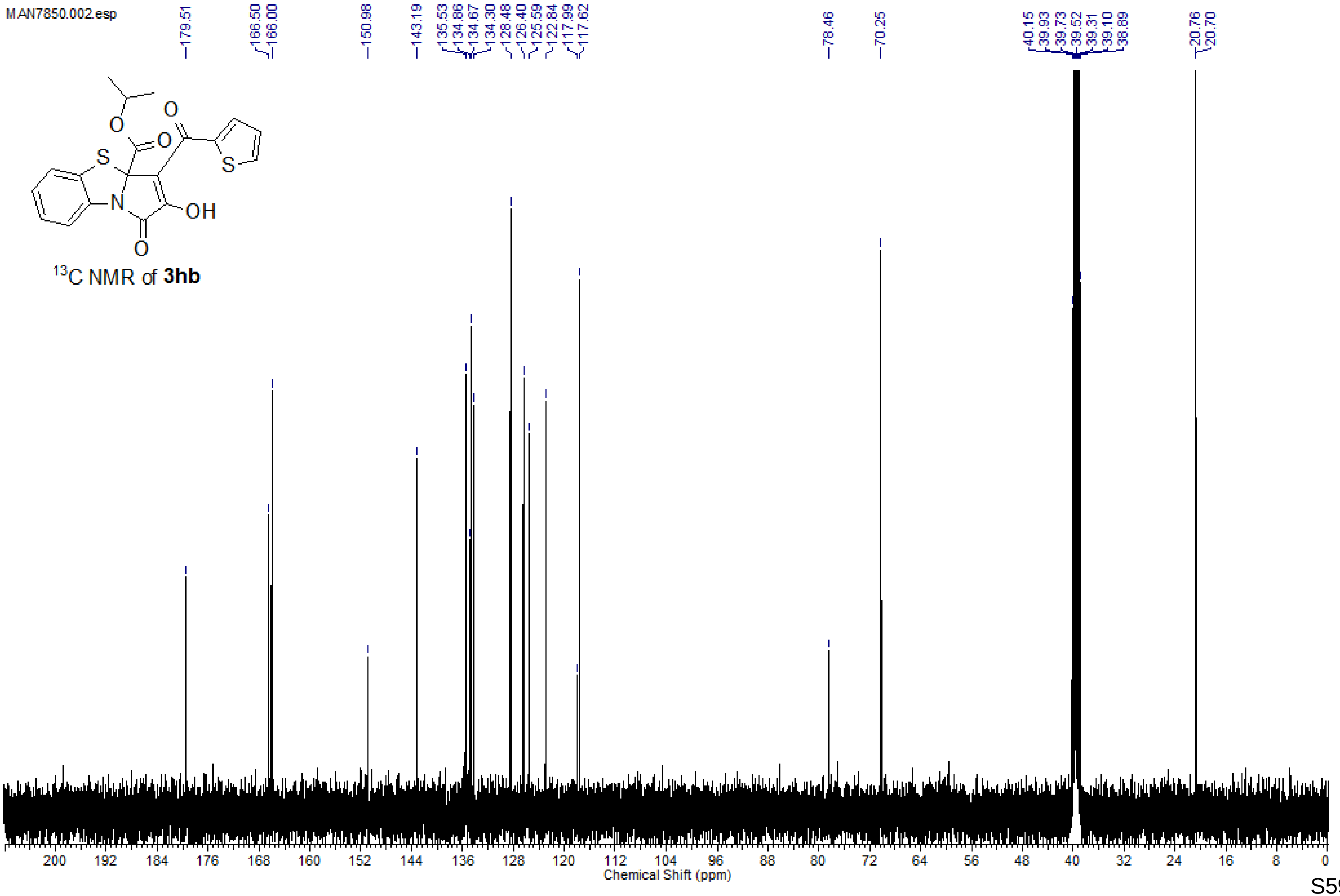

^{13}C NMR of **3hb**

MAN7850.003.esp

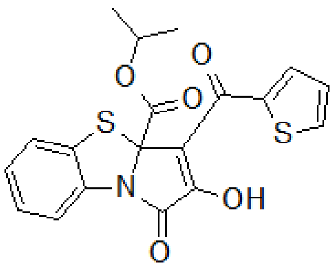

DEPT135 of **3hb**

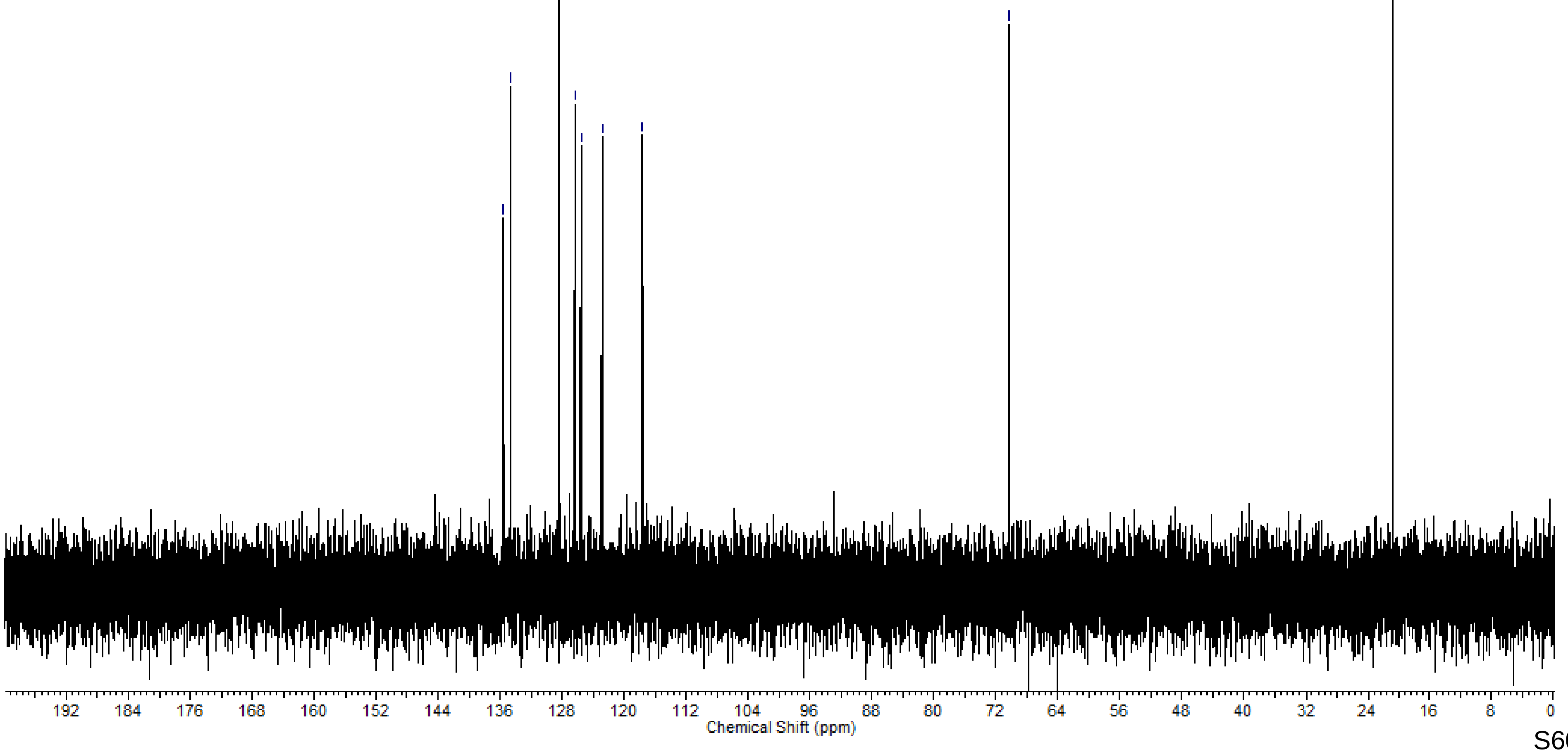

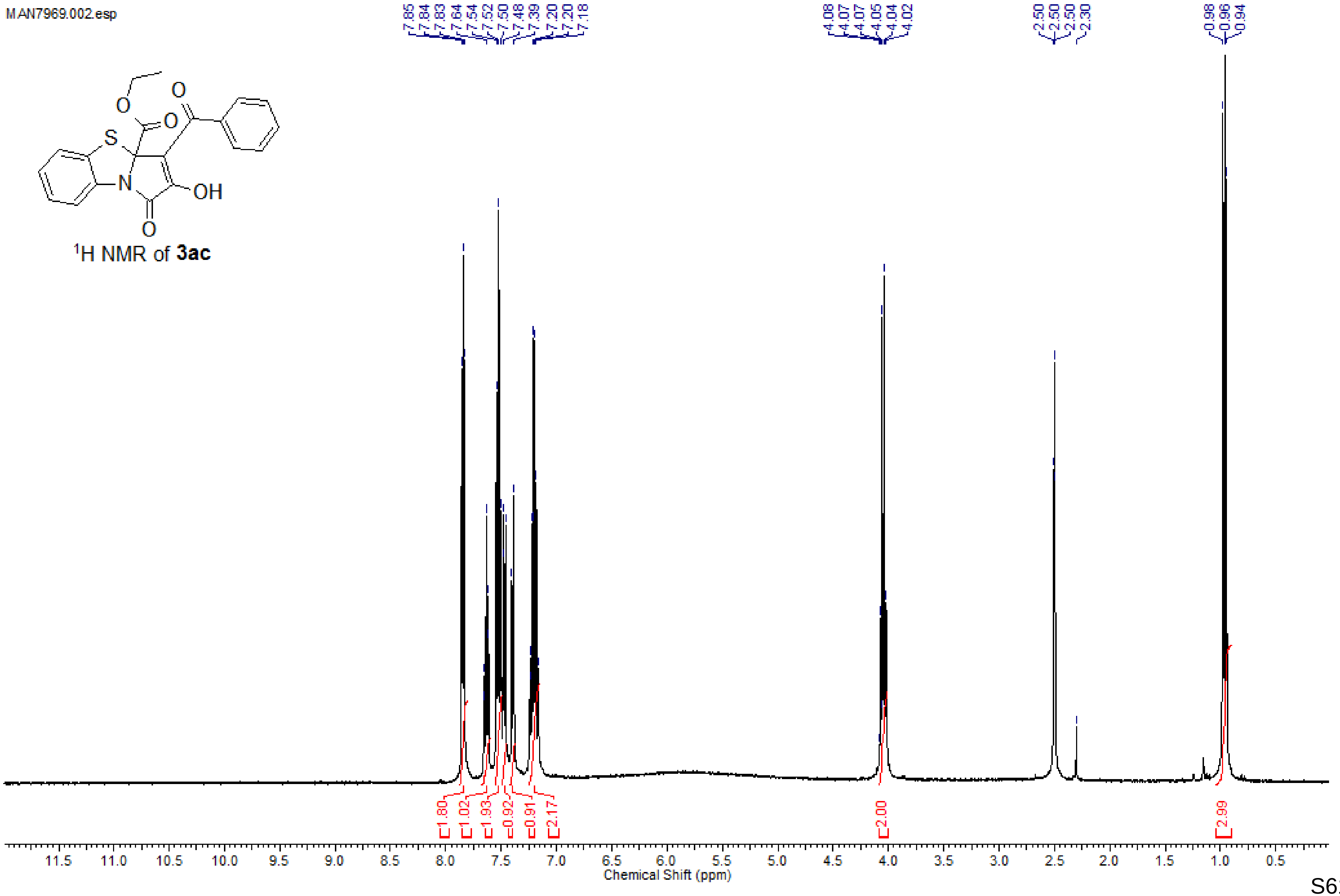

MAN7969.002.esp
7.85
7.84
7.83
7.64
7.54
7.52
7.50
7.48
7.39
7.20
7.20
7.18
4.08
4.07
4.07
4.05
4.04
4.02
2.50
2.50
2.50
2.30
0.98
0.96
0.94
1.80
1.02
1.93
0.92
0.91
2.17
2.00
2.99
Chemical Shift (ppm)

^{1}H NMR of **3ac**

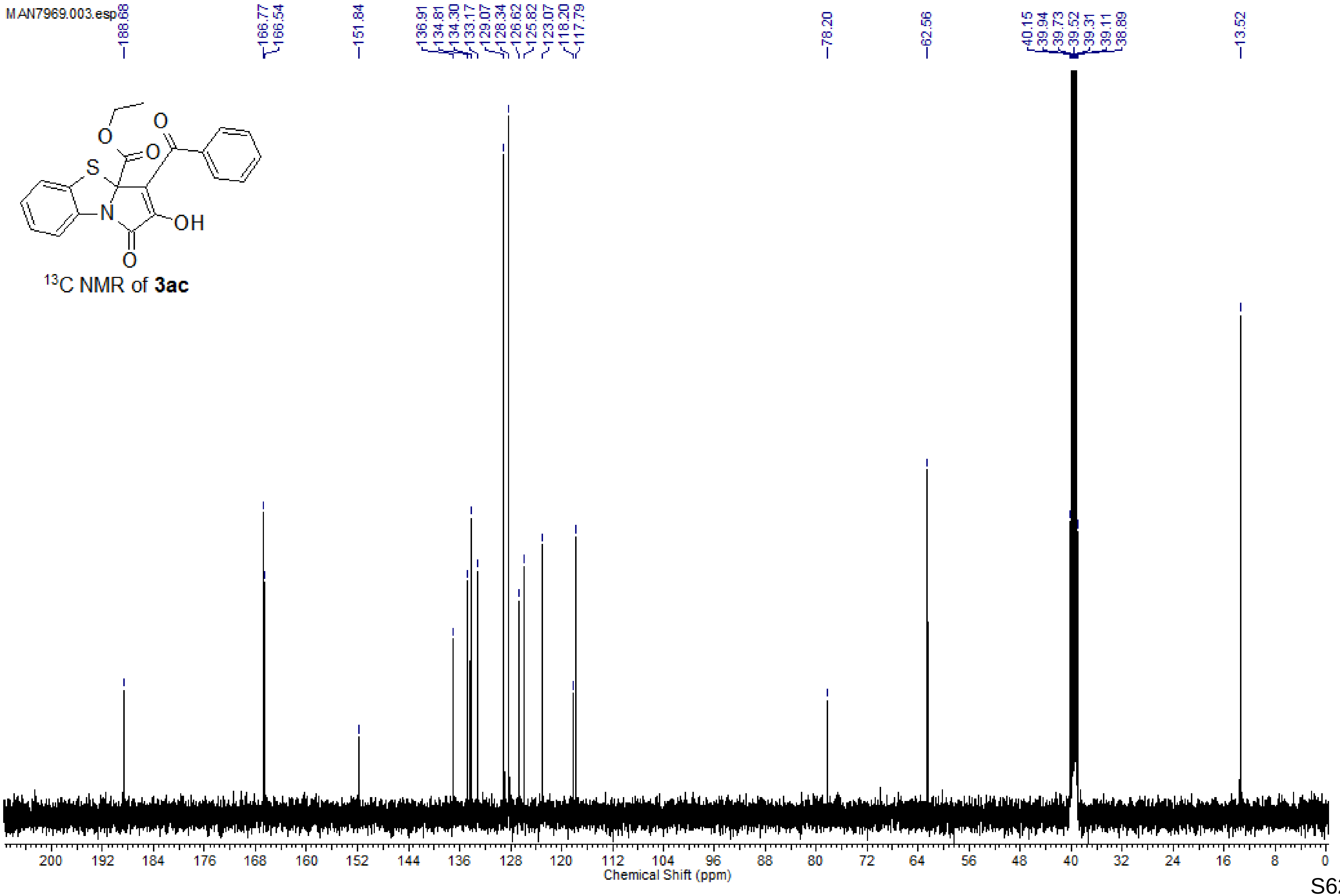

^{13}C NMR of **3ac**

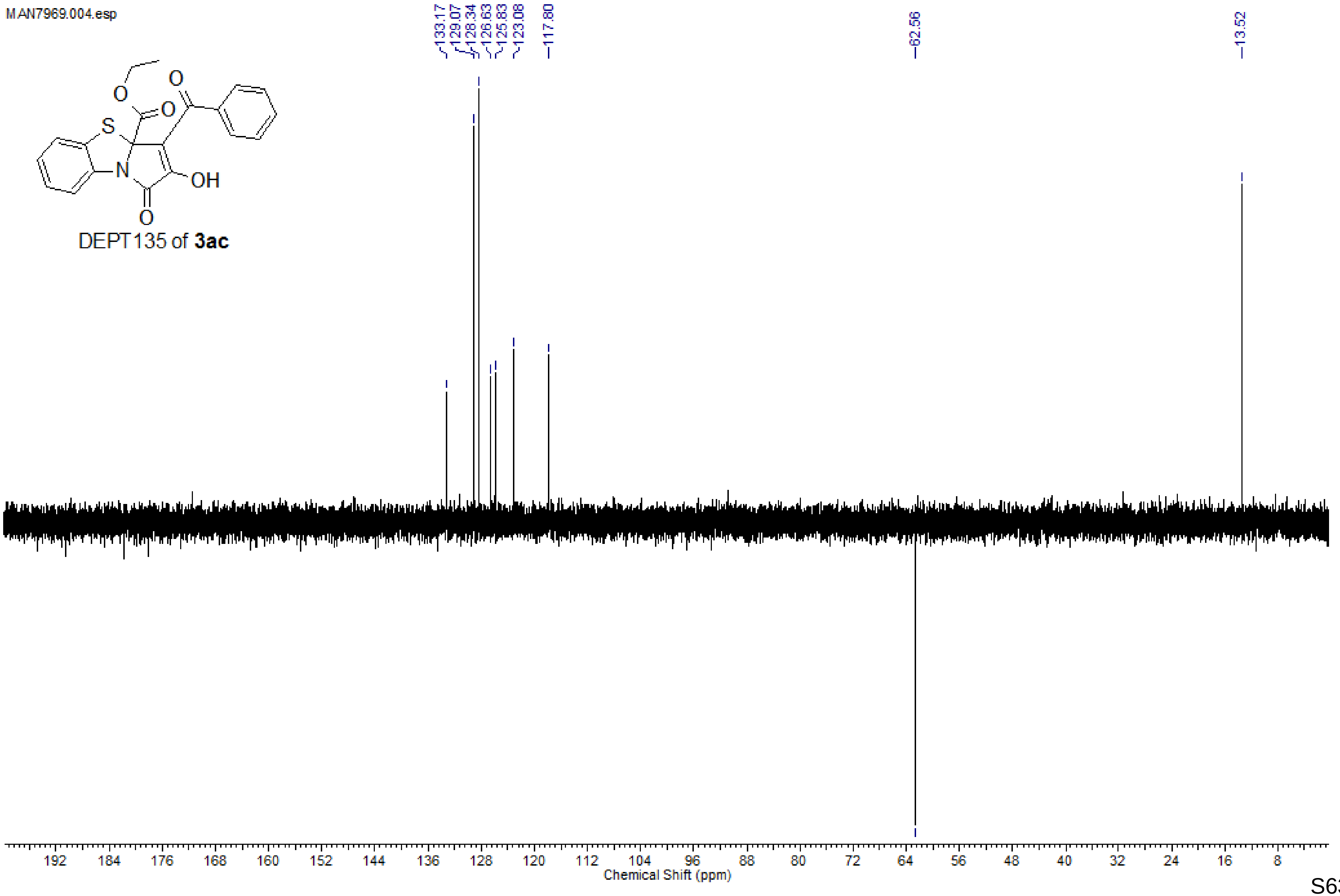

MAN7969.004.esp
133.17
129.07
128.34
126.63
125.83
123.08
117.80
62.56
13.52
O
O
O
S
N
OH
O
DEPT135 of 3ac
192
184
176
168
160
152
144
136
128
120
112
104
96
88
80
72
64
56
48
40
32
24
16
8
Chemical Shift (ppm)

3-Aroyl-2-hydroxy-3a-methoxy-1*H*-pyrrolo[2,1-*c*][1,4]benzothiazine-1,4(3a*H*)-diones 5. General procedure.

A mixture of APBTT **1** (0.45 mmol), methanol (20 µL, 0.49 mmol), and anhydrous toluene (300 µL) is stirred in a closed microreaction V-vial at room temperature for 24 h. Then, the formed precipitate of compound **5** is filtered off, and washed with anhydrous toluene. Compounds **5** should not be dried in an oven or under vacuum, since they readily eliminate methanol.

3-Benzoyl-2-hydroxy-3a-methoxy-1*H*-pyrrolo[2,1-*c*][1,4]benzothiazine-1,4(3a*H*)-dione (5a)
Beige solid; yield (83 mg, 50%); mp 126–128°C (toluene).
^{1}H NMR (400 MHz, DMSO-d_6): δ = 8.02 (m, 1H), 7.85 (m, 2H), 7.62 (m, 1H), 7.54–7.48 (m, 4H), 7.39 (m, 1H), 3.11 (s, 3H) ppm.
^{13}C NMR (100 MHz, DMSO-d_6): δ = 189.4, 188.2, 162.5, 152.3, 137.5, 132.7, 129.2 (2C), 128.1 (2C), 128.0, 127.8, 126.6, 126.5, 123.0, 122.6, 108.7, 90.7, 50.1 ppm.
IR (mineral oil): 3180, 1747, 1700, 1642 cm^{-1}.
EA: Found: C, 62.36; H, 3.71; N, 3.86. Calc. for $C_{19}H_{13}NO_5S$: C, 62.12; H, 3.57; N, 3.81.

2-Hydroxy-3a-methoxy-3-(4-methylbenzoyl)-1*H*-pyrrolo[2,1-*c*][1,4]benzothiazine-1,4(3a*H*)-dione (5b)
Beige solid; yield (129 mg, 75%); mp 107–109°C (toluene).
^{1}H NMR (400 MHz, DMSO-d_6): δ = 8.02 (m, 1H), 7.75 (m, 2H), 7.53 (m, 2H), 7.39 (m, 1H), 7.31 (m, 2H), 3.10 (s, 3H), 2.40 (s, 3H) ppm.
^{13}C NMR (100 MHz, DMSO-d_6): δ = 189.0, 188.3, 162.6, 151.7, 143.2, 135.0, 129.4 (2C), 128.7 (2C), 128.0, 127.8, 126.6, 126.5, 123.0, 122.6, 109.1, 90.7, 50.1, 21.1 ppm.
IR (mineral oil): 3182, 1744, 1699, 1633 cm^{-1}.
EA: Found: C, 65.15; H, 4.36; N, 3.44. Calc. for $3C_{20}H_{15}NO_5S \cdot C_7H_8$: C, 65.09; H, 4.32; N, 3.40.

3-(4-Chlorobenzoyl)-2-Hydroxy-3a-methoxy-1*H*-pyrrolo[2,1-*c*][1,4]benzothiazine-1,4(3a*H*)-dione (5e)
White solid; yield (136 mg, 75%); mp 113–115°C (toluene).
^{1}H NMR (400 MHz, DMSO-d_6): δ = 8.01 (m, 1H), 7.87 (m, 2H), 7.58–7.49 (m, 4H), 7.39 (m, 1H), 3.09 (s, 3H) ppm.
^{13}C NMR (100 MHz, DMSO-d_6): δ = 188.3, 188.2, 162.5, 152.9, 137.7, 136.3, 131.0 (2C), 128.3 (2C), 128.0, 127.7, 126.6, 126.5, 122.99, 122.6, 108.2, 90.6, 50.1 ppm.
IR (mineral oil): 3193, 1744, 1701, 1633 cm^{-1}.
EA: Found: C, 59.31; H, 3.46; N, 3.32. Calc. for $3C_{19}H_{12}ClNO_5S \cdot C_7H_8$: C, 59.24; H, 3.42; N, 3.24.

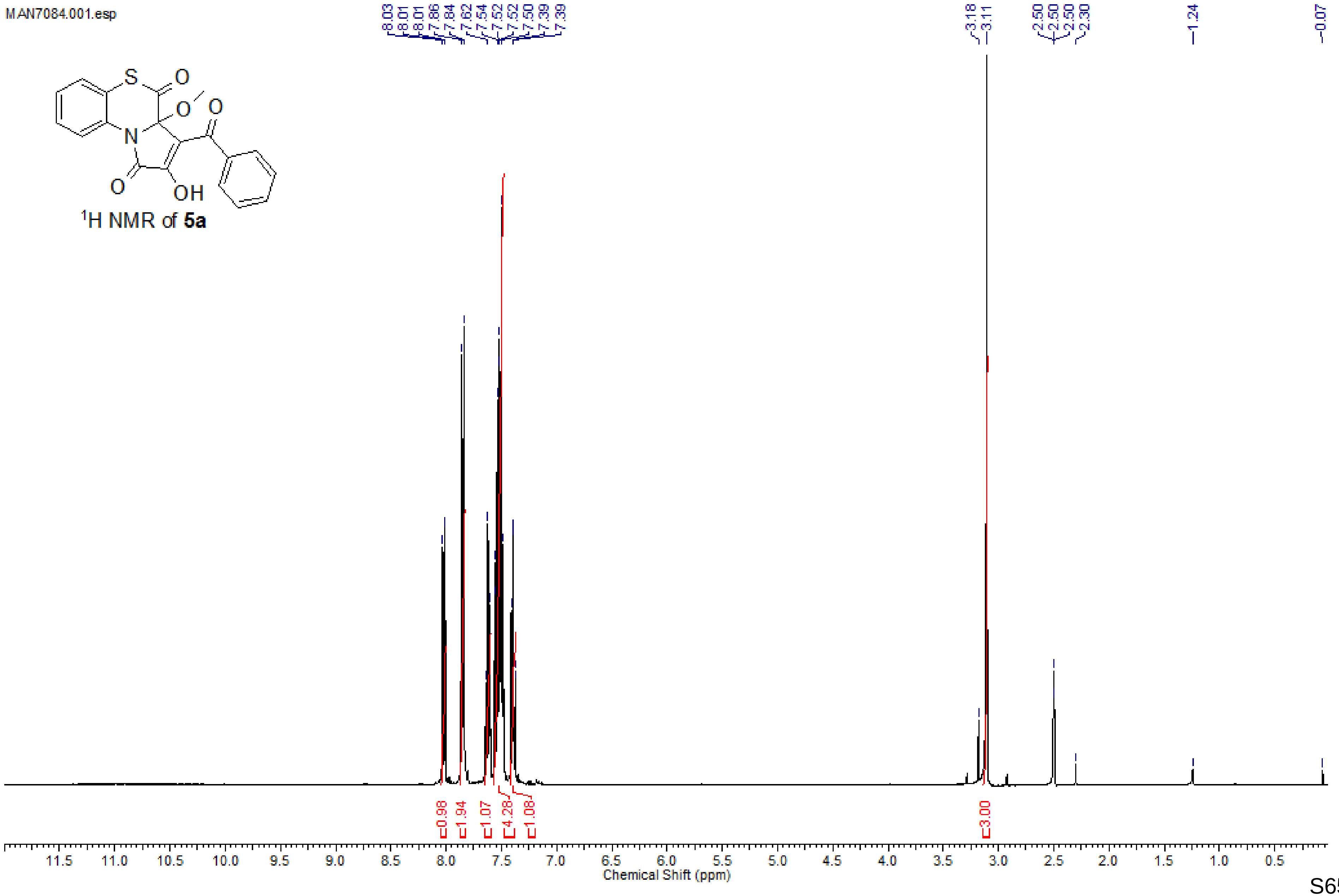
MAN7084.001.esp
S
O
O
O
N
O
OH
8.03
8.01
8.01
7.86
7.84
7.62
7.54
7.52
7.52
7.50
7.39
7.39
3.18
3.11
2.50
2.50
2.50
2.30
1.24
-0.07
0.98
1.94
1.07
4.28
1.08
3.00
11.5
11.0
10.5
10.0
9.5
9.0
8.5
8.0
7.5
7.0
6.5
6.0
5.5
5.0
4.5
4.0
3.5
3.0
2.5
2.0
1.5
1.0
0.5
Chemical Shift (ppm)

^{1}H NMR of **5a**

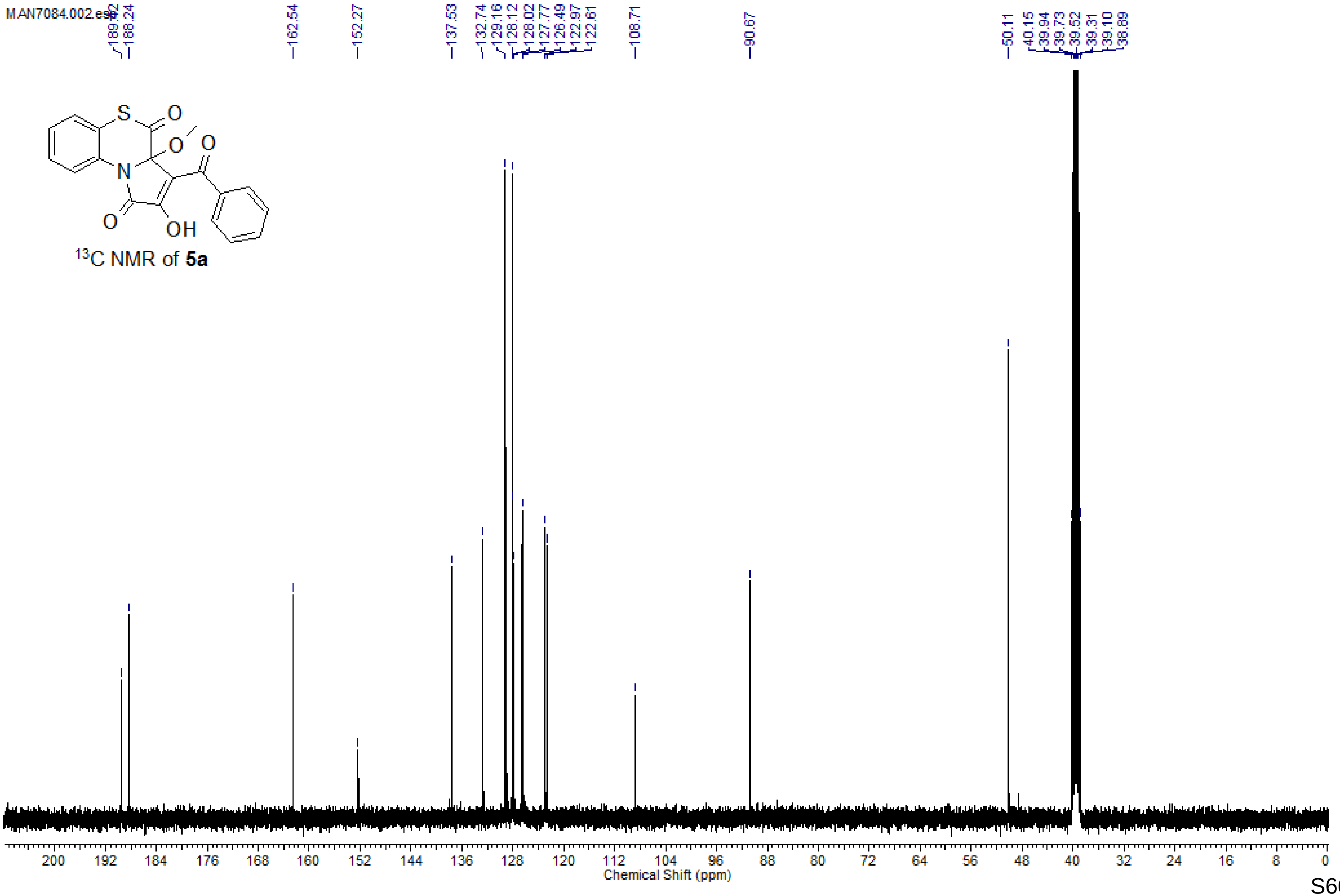
MAN7084.002.esp
188.24
162.54
152.27
137.53
132.74
129.16
128.12
128.02
127.77
126.49
122.97
122.61
108.71
90.67
50.11
40.15
39.94
39.73
39.52
39.31
39.10
38.89
S
O
O
O
N
O
OH
Chemical Shift (ppm)
200 192 184 176 168 160 152 144 136 128 120 112 104 96 88 80 72 64 56 48 40 32 24 16 8 0

^{13}C NMR of **5a**

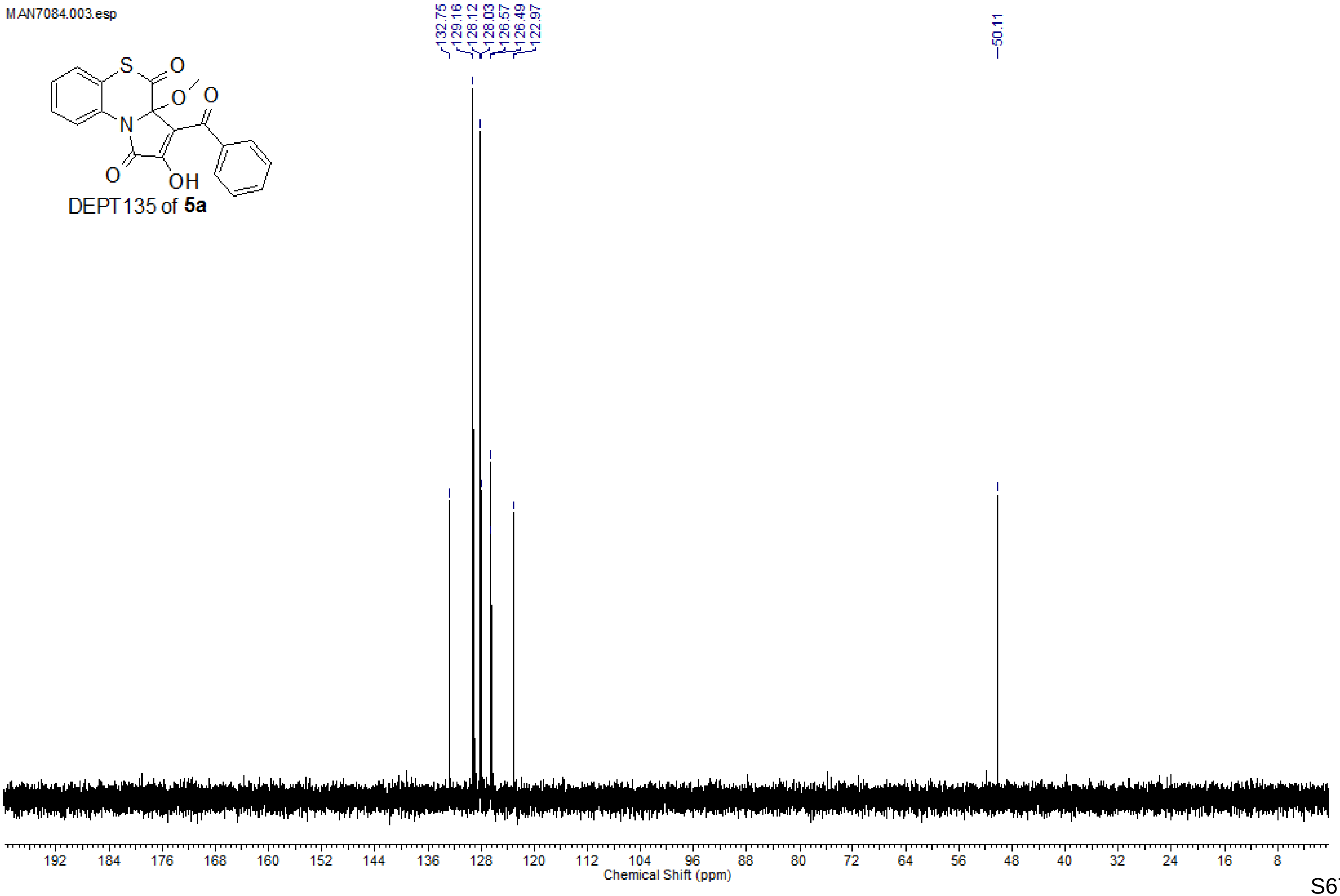
MAN7084.003.esp
DEPT135 of 5a
132.75
129.16
128.12
128.03
126.57
126.49
122.97
50.11
192 184 176 168 160 152 144 136 128 120 112 104 96 88 80 72 64 56 48 40 32 24 16 8
Chemical Shift (ppm)

MAN7143.001.esp

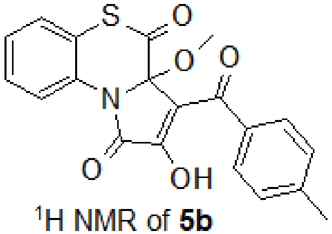

^{1}H NMR of **5b**

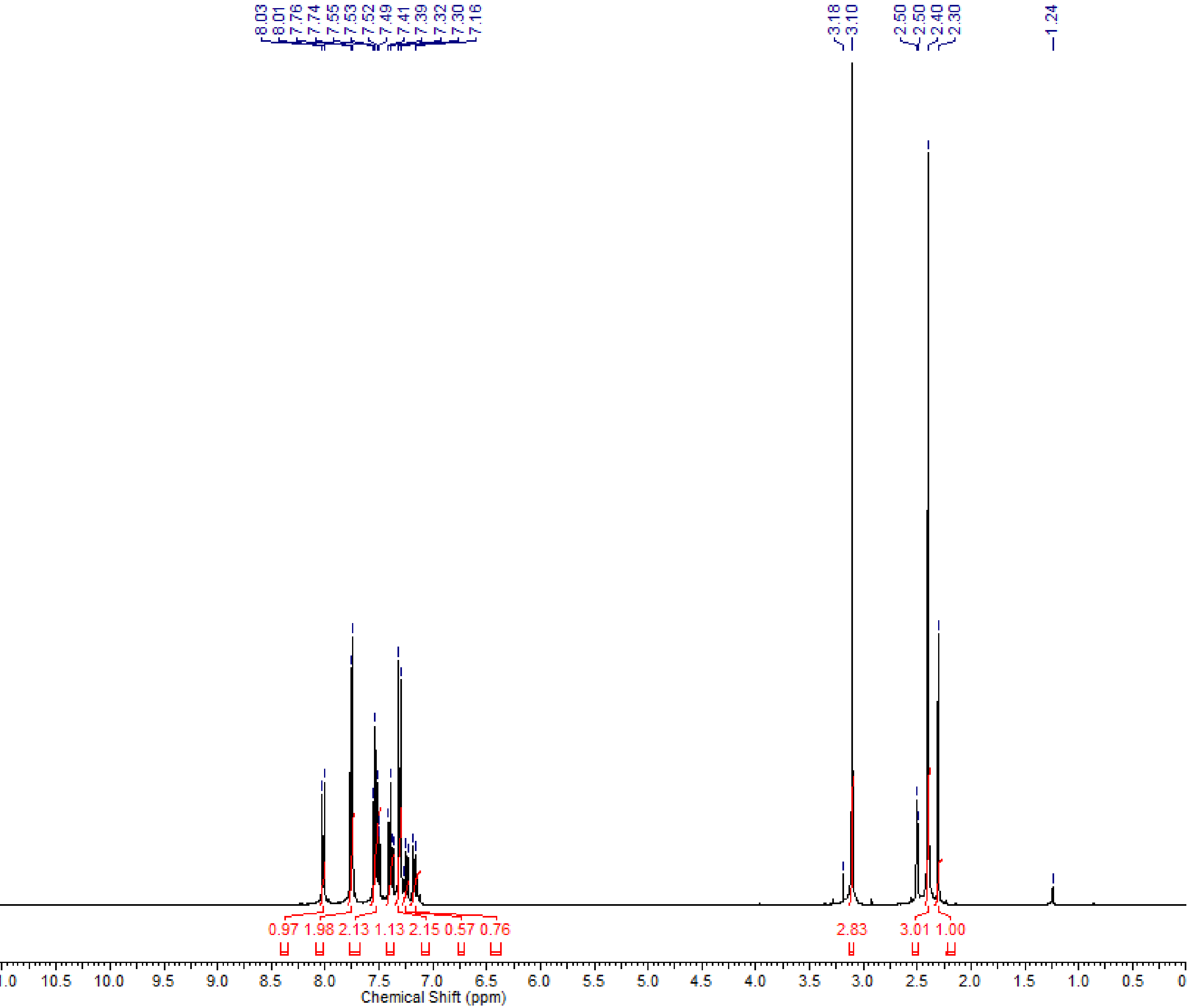

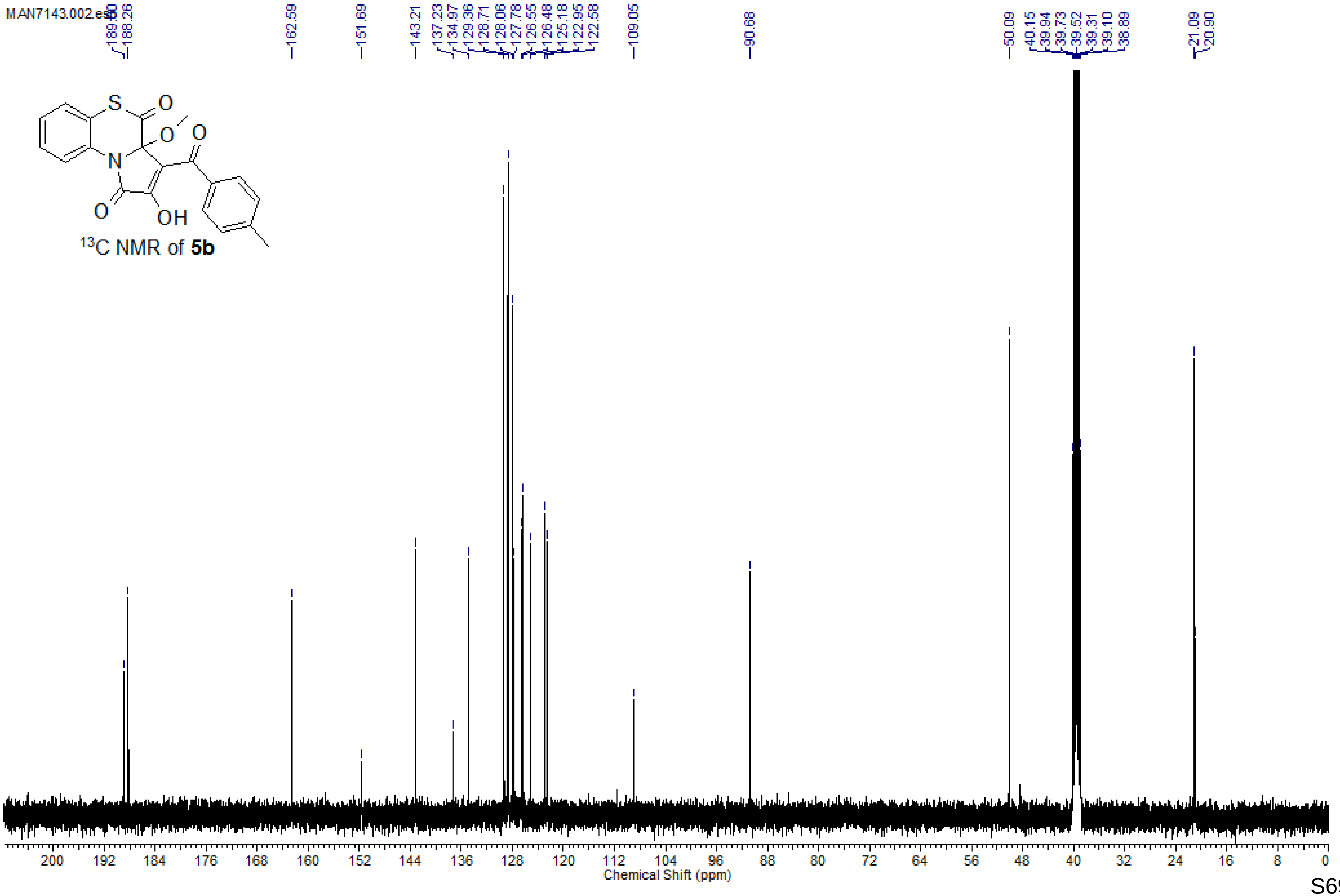
MAN7143.002.esp
[illegible]
188.26
162.59
151.69
143.21
137.23
134.97
129.36
128.71
128.06
127.78
126.55
126.48
125.18
122.95
122.58
109.05
90.68
50.09
40.15
39.94
39.73
39.52
39.31
39.10
38.89
21.09
20.90
S
O
O
O
N
O
OH
13C NMR of 5b
200
192
184
176
168
160
152
144
136
128
120
112
104
96
88
80
72
64
56
48
40
32
24
16
8
0
Chemical Shift (ppm)

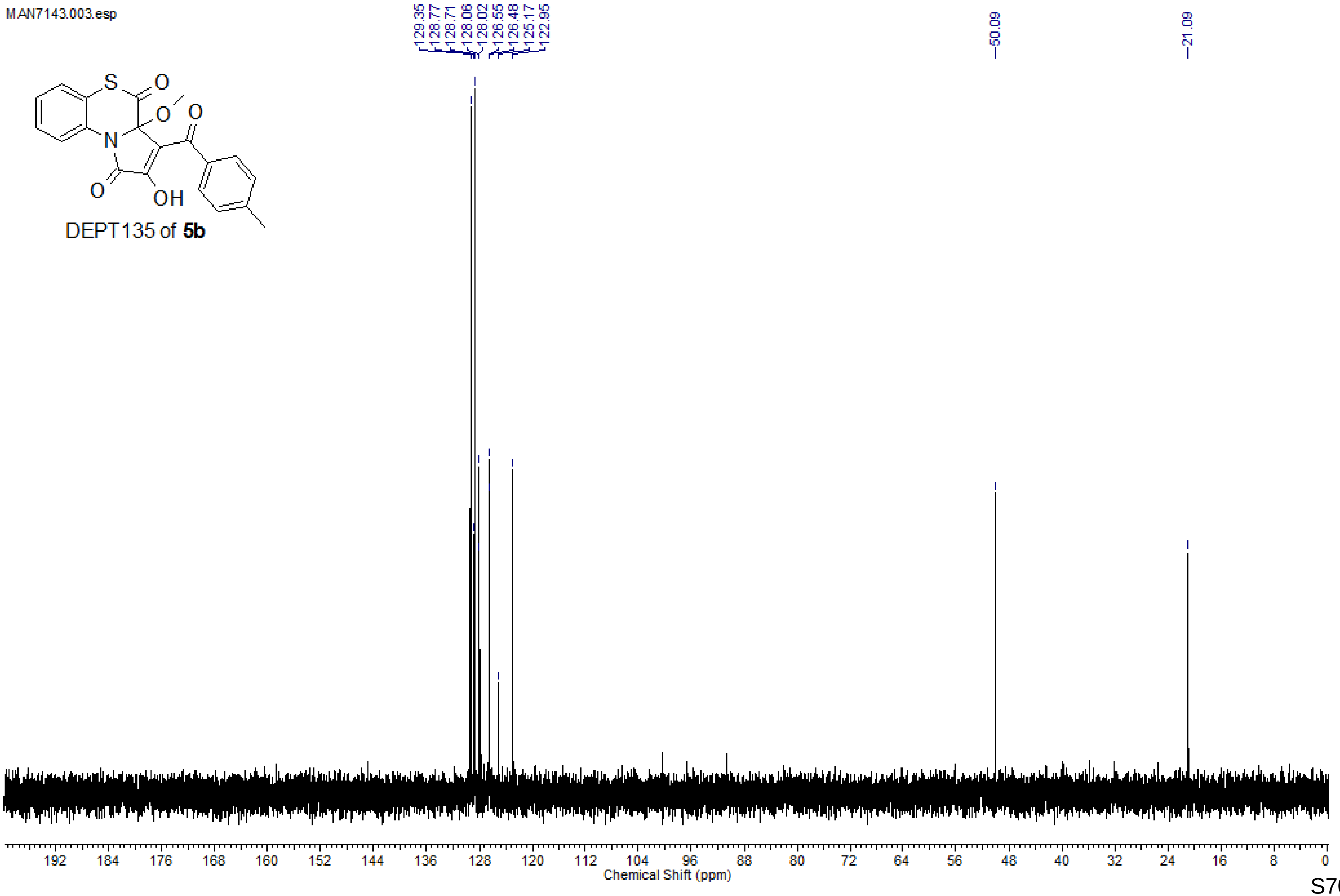
MAN7143.003.esp
129.35
128.77
128.71
128.06
128.02
126.55
126.48
125.17
122.95
50.09
21.09
S
O
O
O
N
O
OH
DEPT135 of 5b
192 184 176 168 160 152 144 136 128 120 112 104 96 88 80 72 64 56 48 40 32 24 16 8 0
Chemical Shift (ppm)

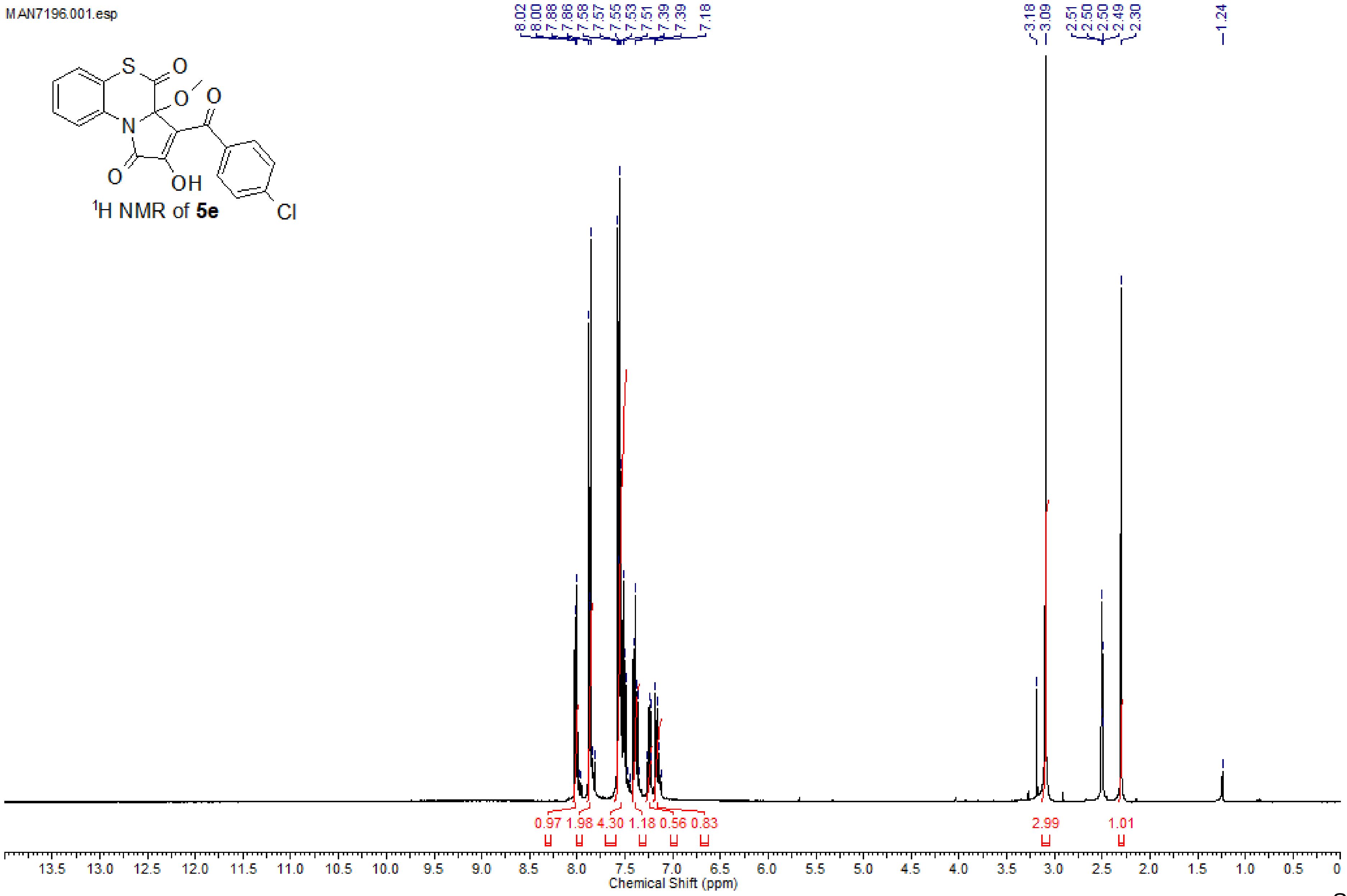
MAN7196.001.esp
S
O
O
O
N
O
OH
Cl
1H NMR of 5e
8.02
8.00
7.88
7.86
7.58
7.57
7.55
7.53
7.51
7.39
7.39
7.18
3.18
3.09
2.51
2.50
2.50
2.49
2.30
1.24
0.97
1.98
4.30
1.18
0.56
0.83
2.99
1.01
13.5 13.0 12.5 12.0 11.5 11.0 10.5 10.0 9.5 9.0 8.5 8.0 7.5 7.0 6.5 6.0 5.5 5.0 4.5 4.0 3.5 3.0 2.5 2.0 1.5 1.0 0.5 0
Chemical Shift (ppm)

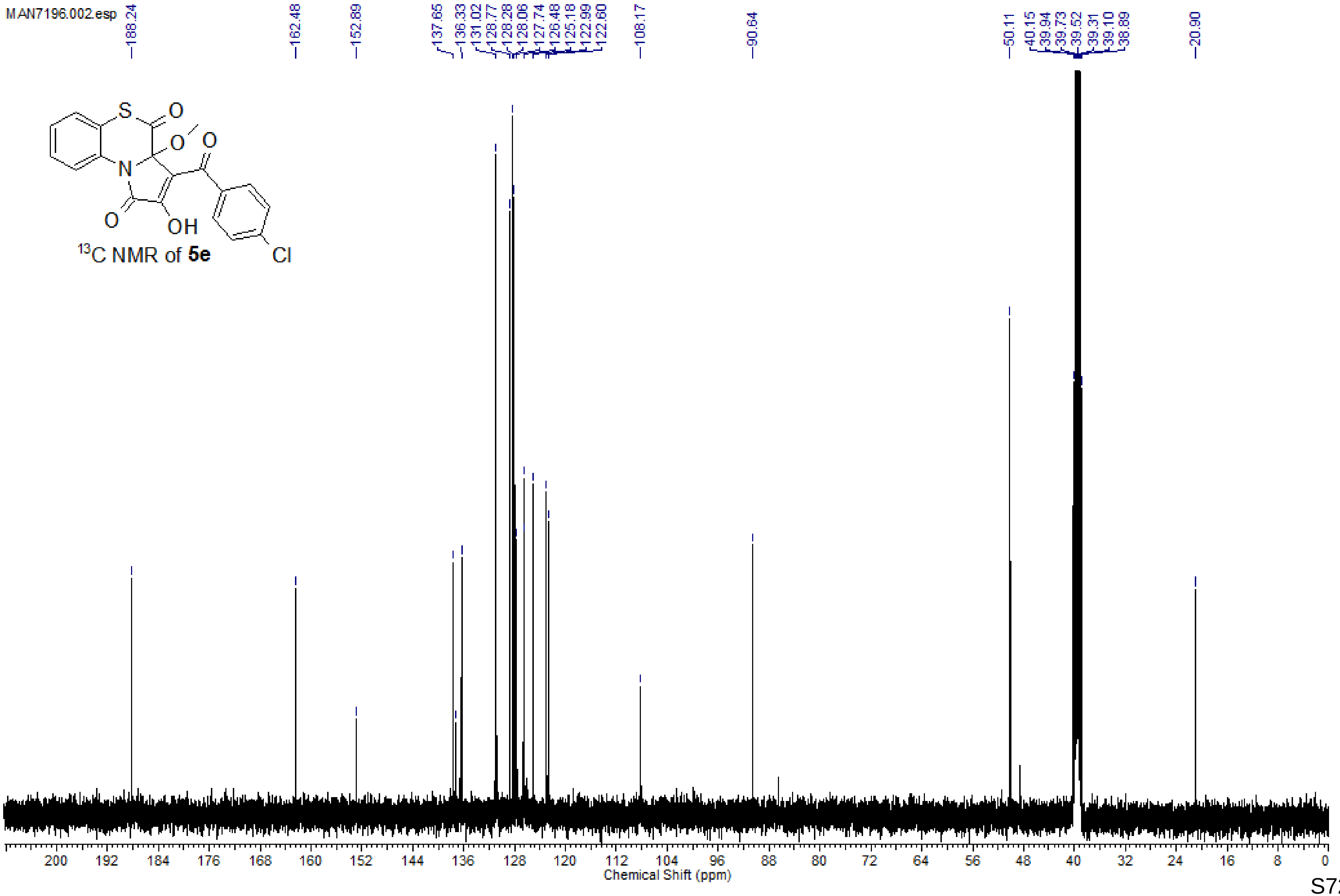
MAN7196.002.esp
188.24
162.48
152.89
137.65
136.33
131.02
128.77
128.28
128.06
127.74
126.48
125.18
122.99
122.60
108.17
90.64
50.11
40.15
39.94
39.73
39.52
39.31
39.10
38.89
20.90
^{13}C NMR of **5e**
Chemical Shift (ppm)

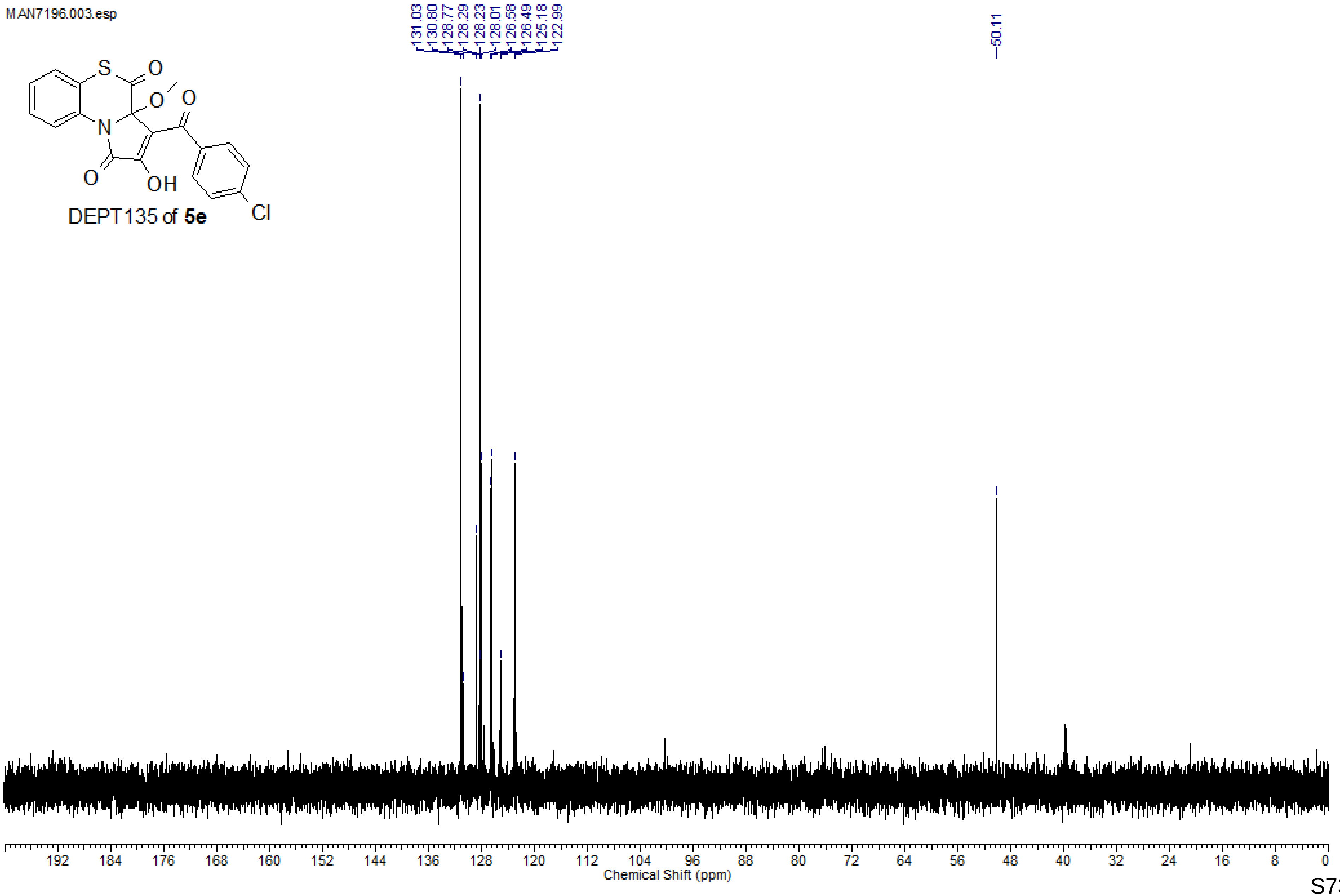
MAN7196.003.esp
DEPT135 of **5e**
131.03
130.80
128.77
128.29
128.23
128.01
126.58
126.49
125.18
122.99
50.11
Chemical Shift (ppm)

3-Aroyl-2,3a-dihydroxy-1*H*-pyrrolo[2,1-*c*][1,4]benzothiazine-1,4(3a*H*)-diones 6. General procedure.

A mixture of APBTT **1** (0.45 mmol) and water (8.8 µL, 0.49 mmol) in anhydrous toluene (3 mL) is stirred in a closed microreaction V-vial at 113 °C for 2 h. Then, the resulting solution is cooled to room temperature. Then, the formed precipitate of compound **6** is filtered off, and washed with toluene. Compounds **6** should not be dried in an oven or under vacuum, since they readily eliminate water.

3-Benzoyl-2,3a-dihydroxy-1*H*-pyrrolo[2,1-*c*][1,4]benzothiazine-1,4(3a*H*)-dione (6a)
Beige solid; yield (127 mg, 80%); mp 148–150°C (toluene).
^{1}H NMR (400 MHz, DMSO-d_6): δ = 7.98 (m, 1H), 7.82 (m, 2H), 7.62 (m, 1H), 7.55–7.46 (m, 4H), 7.36 (m, 1H) ppm.
^{13}C NMR (100 MHz, DMSO-d_6): δ = 189.8, 189.5, 162.6, 151.0, 137.9, 132.6, 129.1 (2C), 129.0, 128.1 (2C), 127.6, 126.3, 126.1, 122.8, 122.6, 113.2, 86.6 ppm.
IR (mineral oil): 3233, 1727, 1666 cm^{-1}.
EA: Found: C, 61.51; H, 3.31; N, 4.22. Calc. for $C_{18}H_{11}NO_5S$: C, 61.19; H, 3.14; N, 3.96.

2,3a-Dihydroxy-3-(4-methylbenzoyl)-1*H*-pyrrolo[2,1-*c*][1,4]benzothiazine-1,4(3a*H*)-dione (6b)
Beige solid; yield (136 mg, 82%); mp 147–149°C (toluene).
^{1}H NMR (400 MHz, DMSO-d_6): δ = 7.97 (m, 1H), 7.72 (m, 2H), 7.53–7.45 (m, 2H), 7.37–7.24 (m, 3H), 2.40 (s, 3H) ppm.
^{13}C NMR (100 MHz, DMSO-d_6): δ = 189.5, 189.5, 162.7, 150.5, 143.0, 135.3, 129.2 (2C), 128.7, 128.7 (2C), 127.6, 126.3, 126.1, 122.8, 122.6, 113.4, 86.6, 21.0 ppm.
IR (mineral oil): 3382, 3085, 1727, 1702, 1661 cm^{-1}.
EA: Found: C, 62.35; H, 3.73; N, 3.94. Calc. for $C_{19}H_{13}NO_5S$: C, 62.12; H, 3.57; N, 3.81.

3-(4-Chlorobenzoyl)-2,3a-dihydroxy-1*H*-pyrrolo[2,1-*c*][1,4]benzothiazine-1,4(3a*H*)-dione (6e)
Beige solid; yield (152 mg, 87%); mp 161–163°C (toluene).
^{1}H NMR (400 MHz, DMSO-d_6): δ = 7.97 (m, 1H), 7.82 (m, 2H), 7.56 (m, 2H), 7.53 (m, 1H), 7.48 (m, 1H), 7.35 (m, 1H) ppm.
^{13}C NMR (100 MHz, DMSO-d_6): δ = 189.5, 188.6, 162.5, 151.5, 137.4, 136.7, 130.8 (2C), 128.7, 128.3 (2C), 127.6, 126.3, 126.2, 122.8, 122.6, 112.7, 86.5 ppm.
IR (mineral oil): 3188, 3061, 1759, 1708 cm^{-1}.
EA: Found: C, 55.93; H, 2.74; N, 3.86. Calc. for $C_{18}H_{10}ClNO_5S$: C, 55.75; H, 2.60; N, 3.61.

MAN7220.001.esp

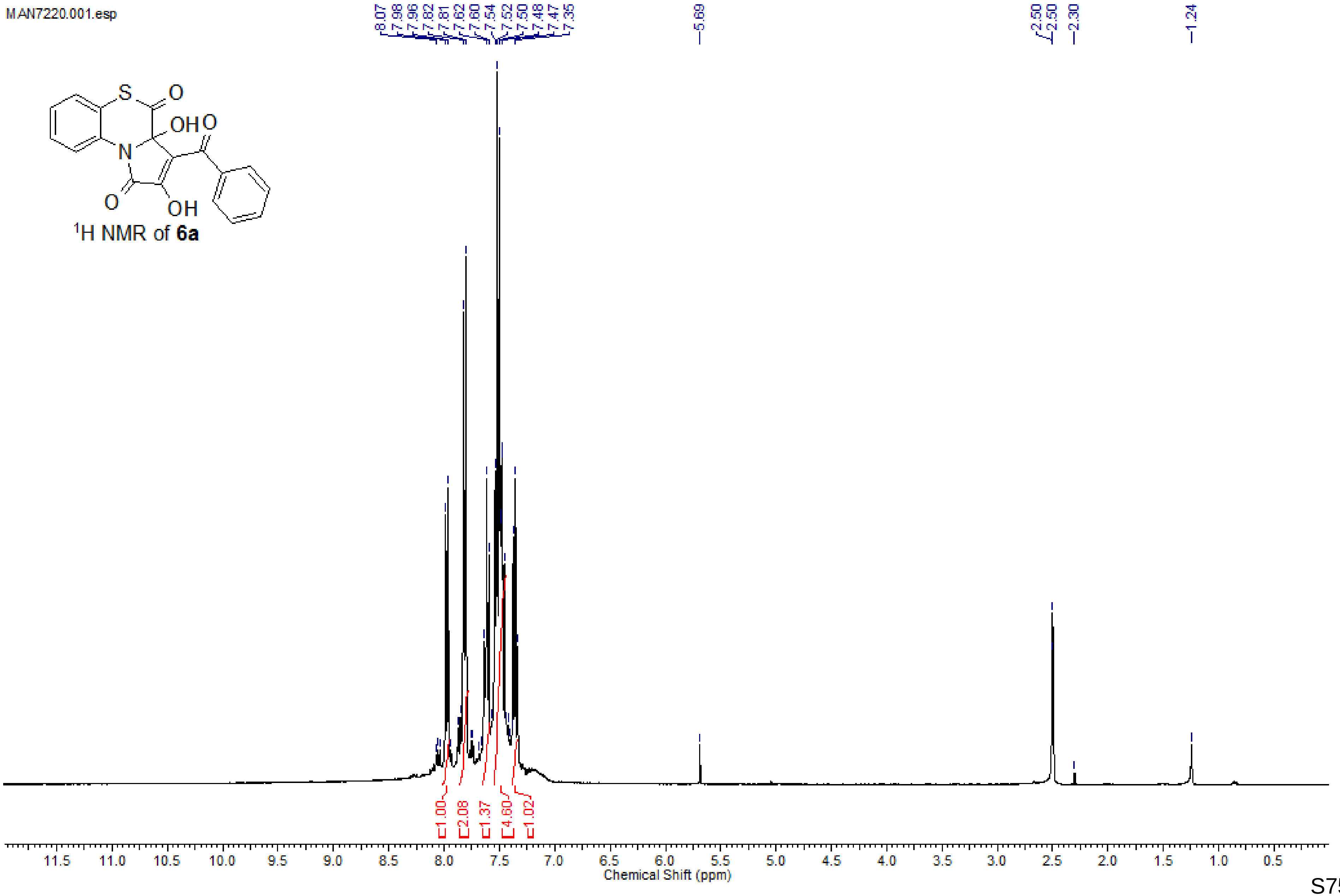

^{1}H NMR of **6a**

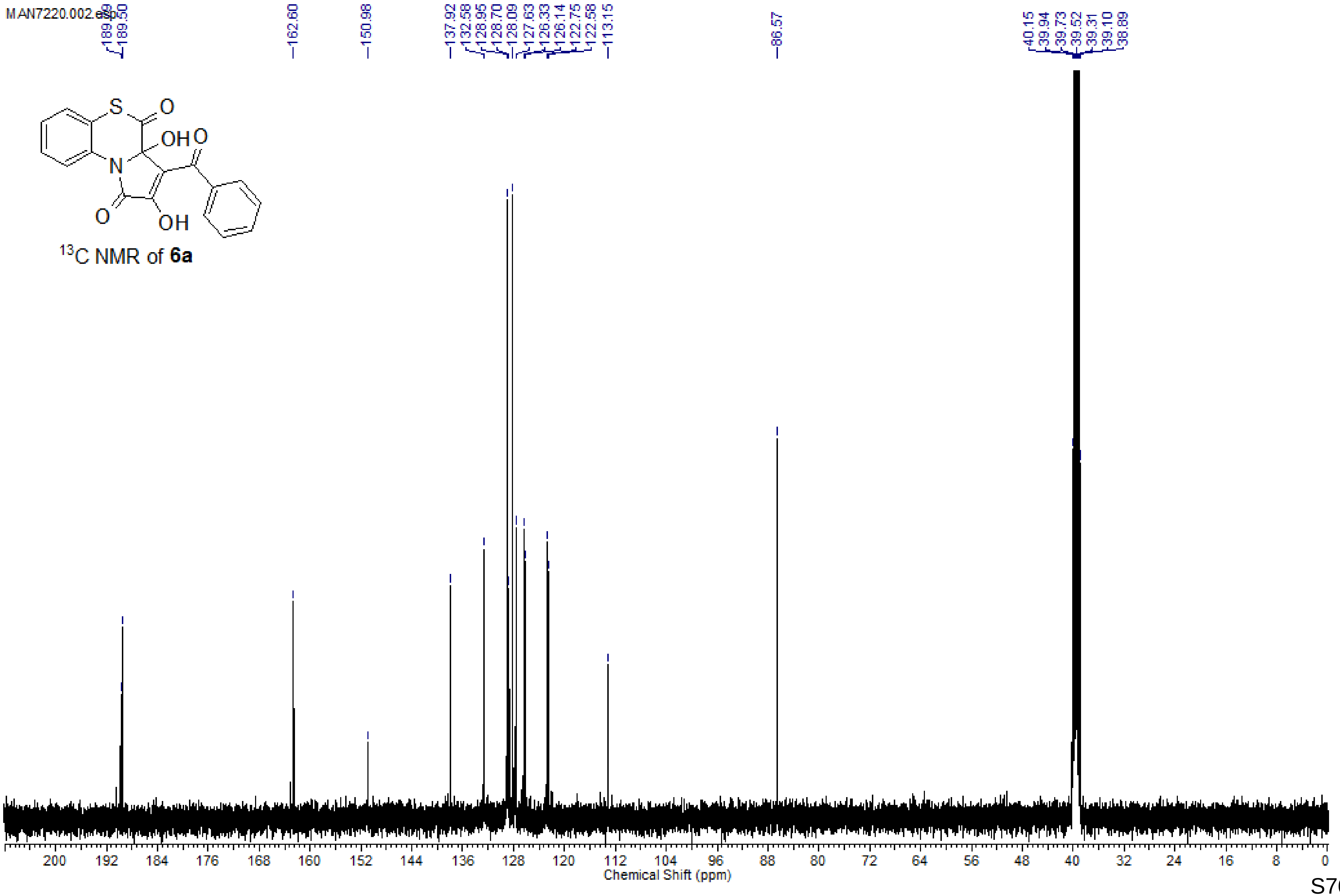
MAN7220.002.esp
189.50
162.60
150.98
137.92
132.58
128.95
128.70
128.09
127.63
126.33
126.14
122.75
122.58
113.15
86.57
40.15
39.94
39.73
39.52
39.31
39.10
38.89
S
O
OH
O
N
O
OH
200 192 184 176 168 160 152 144 136 128 120 112 104 96 88 80 72 64 56 48 40 32 24 16 8 0
Chemical Shift (ppm)

^{13}C NMR of **6a**

MAN7220.003.esp

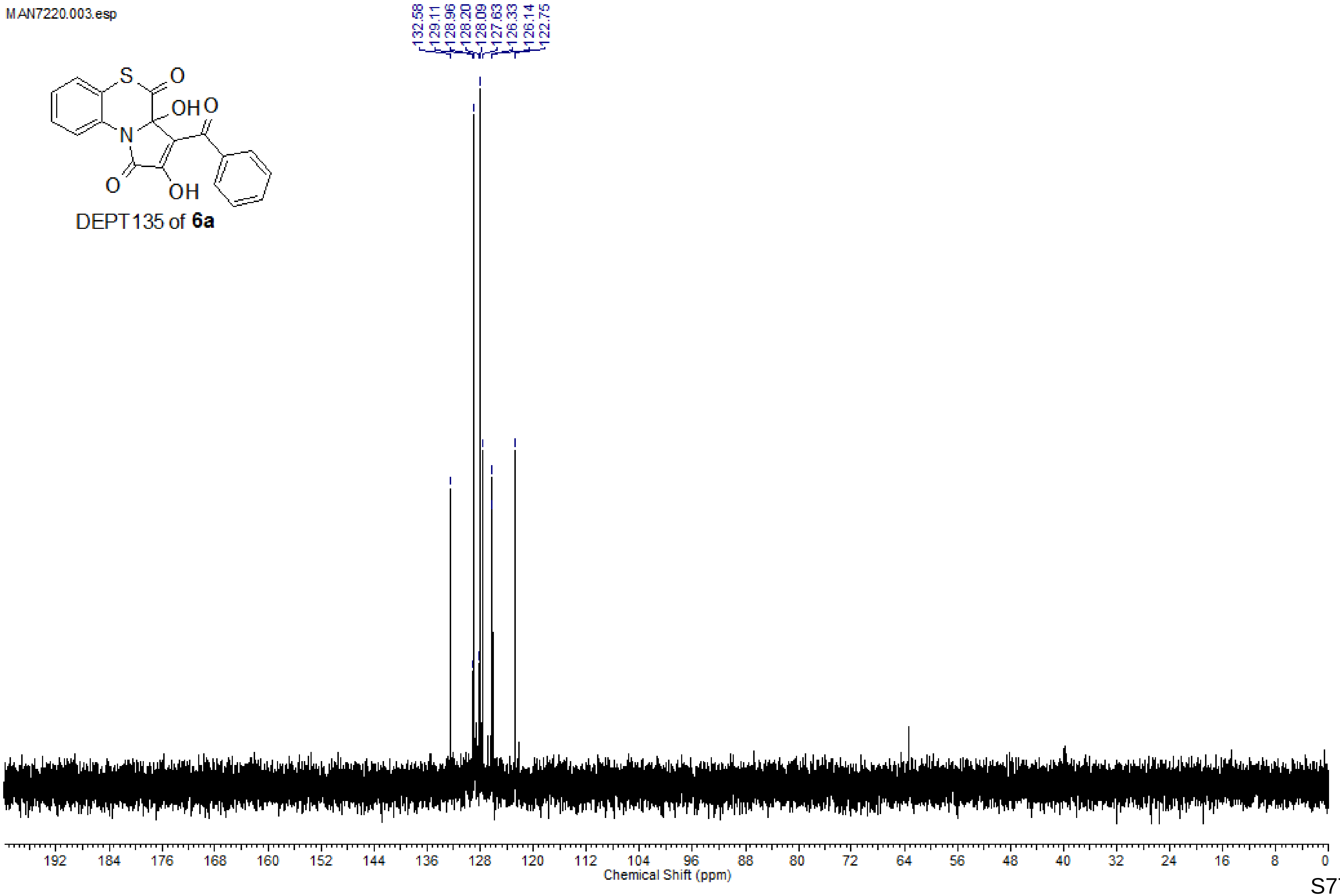

DEPT135 of **6a**

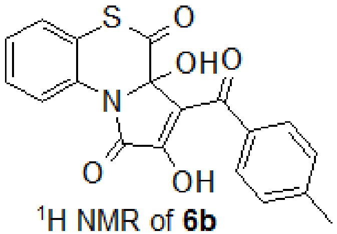

^{1}H NMR of **6b**

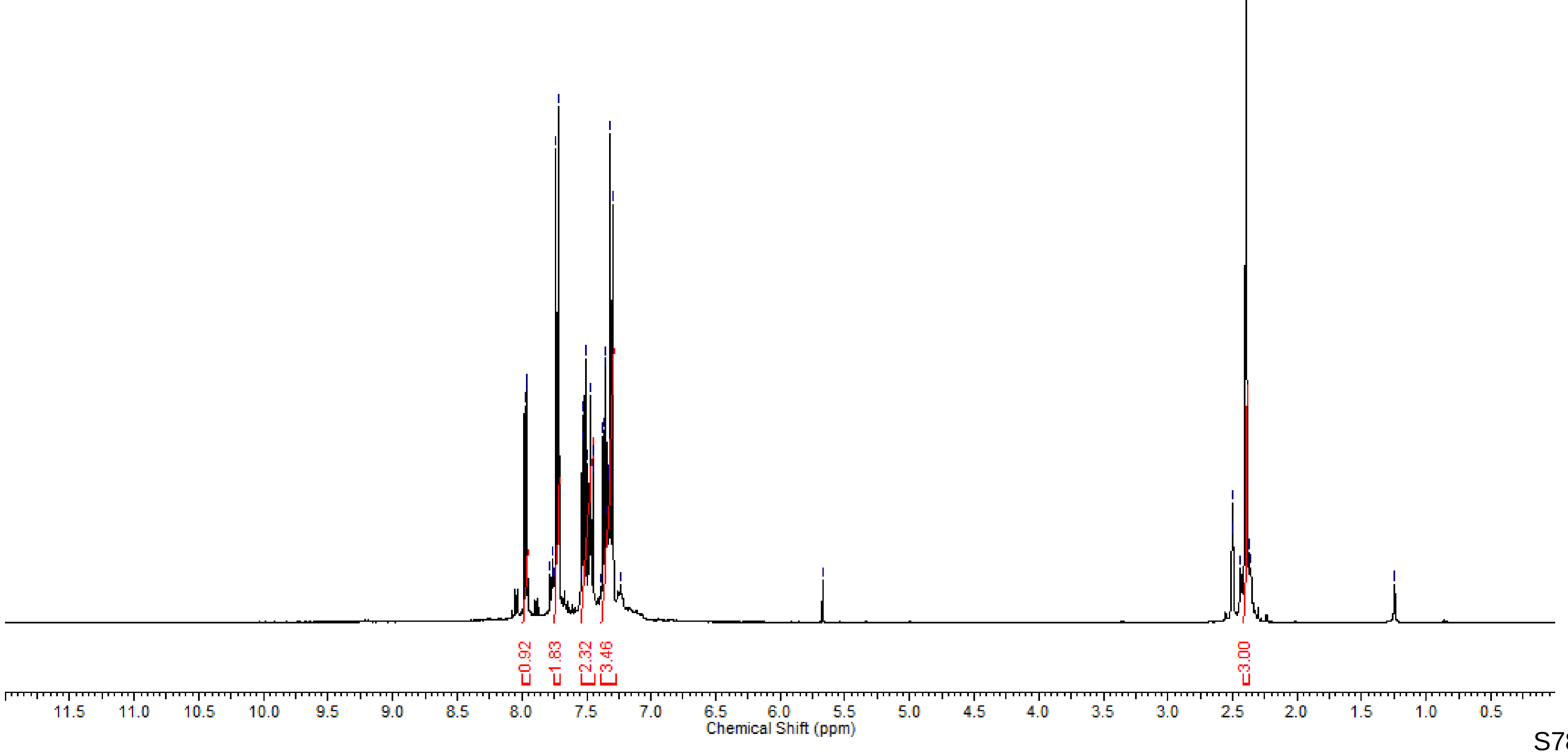

MAN7222.001.esp
7.98 7.96 7.96 7.73 7.71 7.53 7.51 7.47 7.35 7.35 7.32 7.30
5.67
2.50 2.50 2.50 2.44 2.40 2.37 2.36
1.24
0.92
1.83
2.32
3.46
3.00
Chemical Shift (ppm)

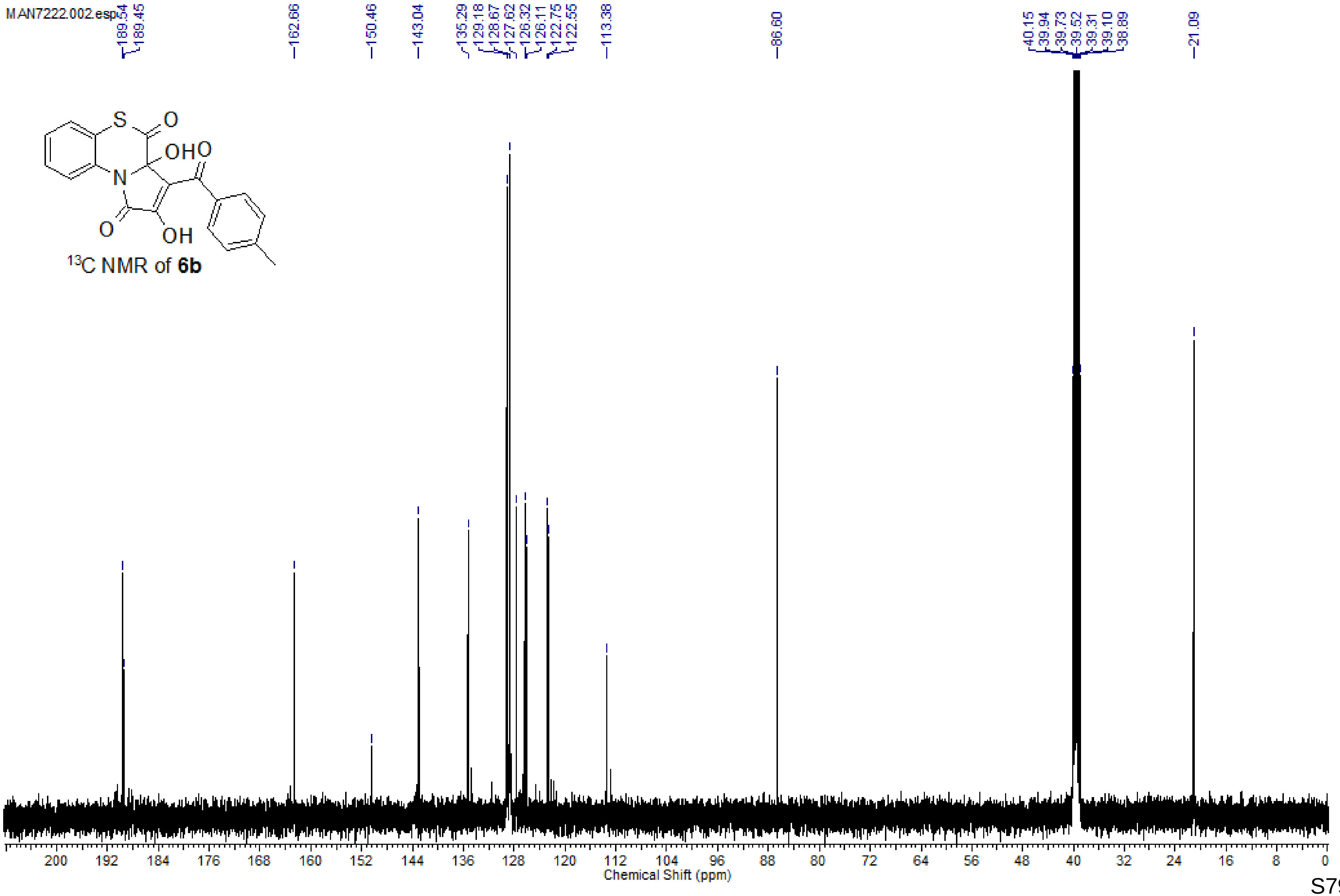

MAN7222.002.esp
189.54
189.45
162.66
150.46
143.04
135.29
129.18
128.67
127.62
126.32
126.11
122.75
122.55
113.38
86.60
40.15
39.94
39.73
39.52
39.31
39.10
38.89
21.09
S
O
OH
O
N
O
OH
^{13}C NMR of **6b**
Chemical Shift (ppm)

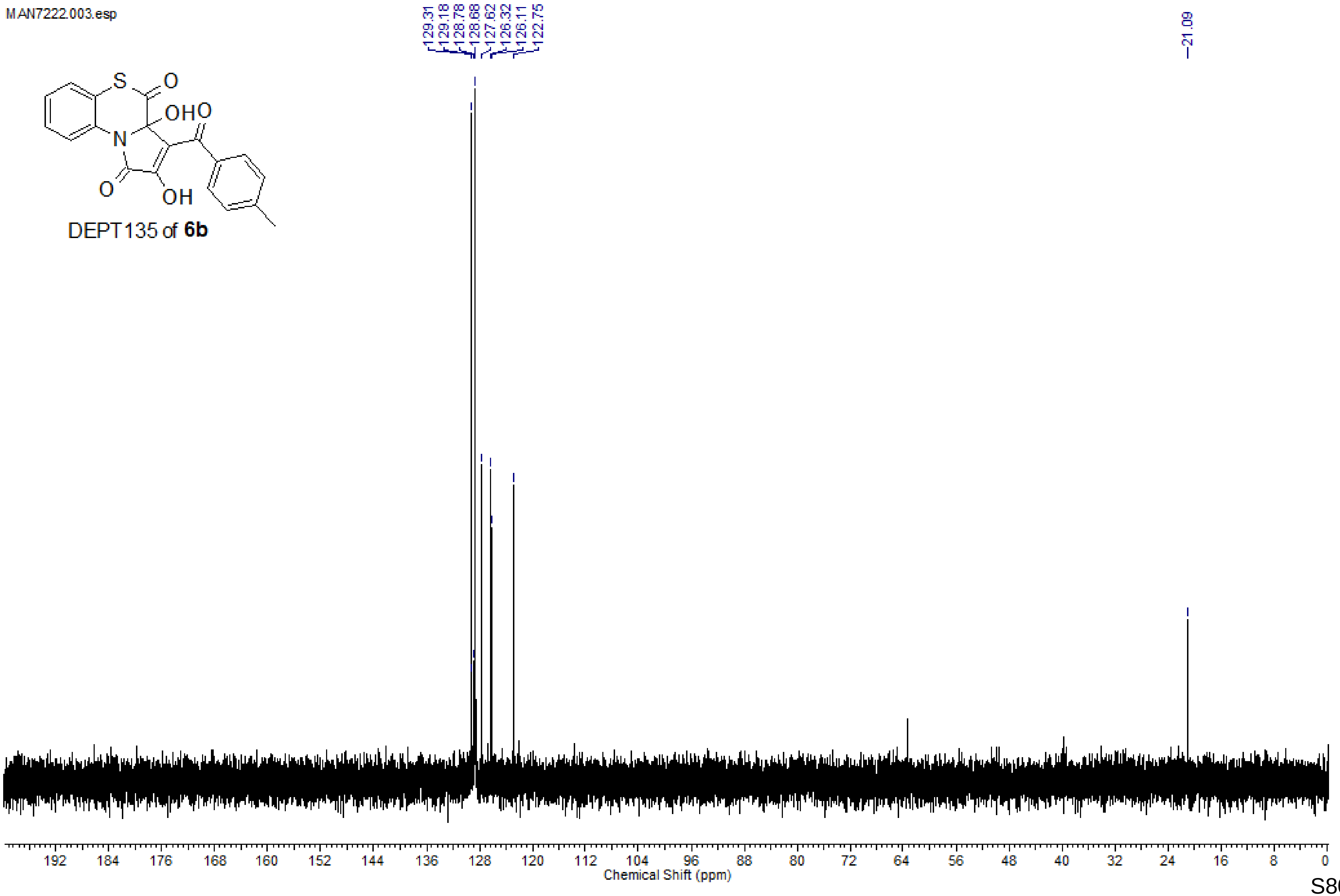

DEPT135 of **6b**

MAN7221.001.esp

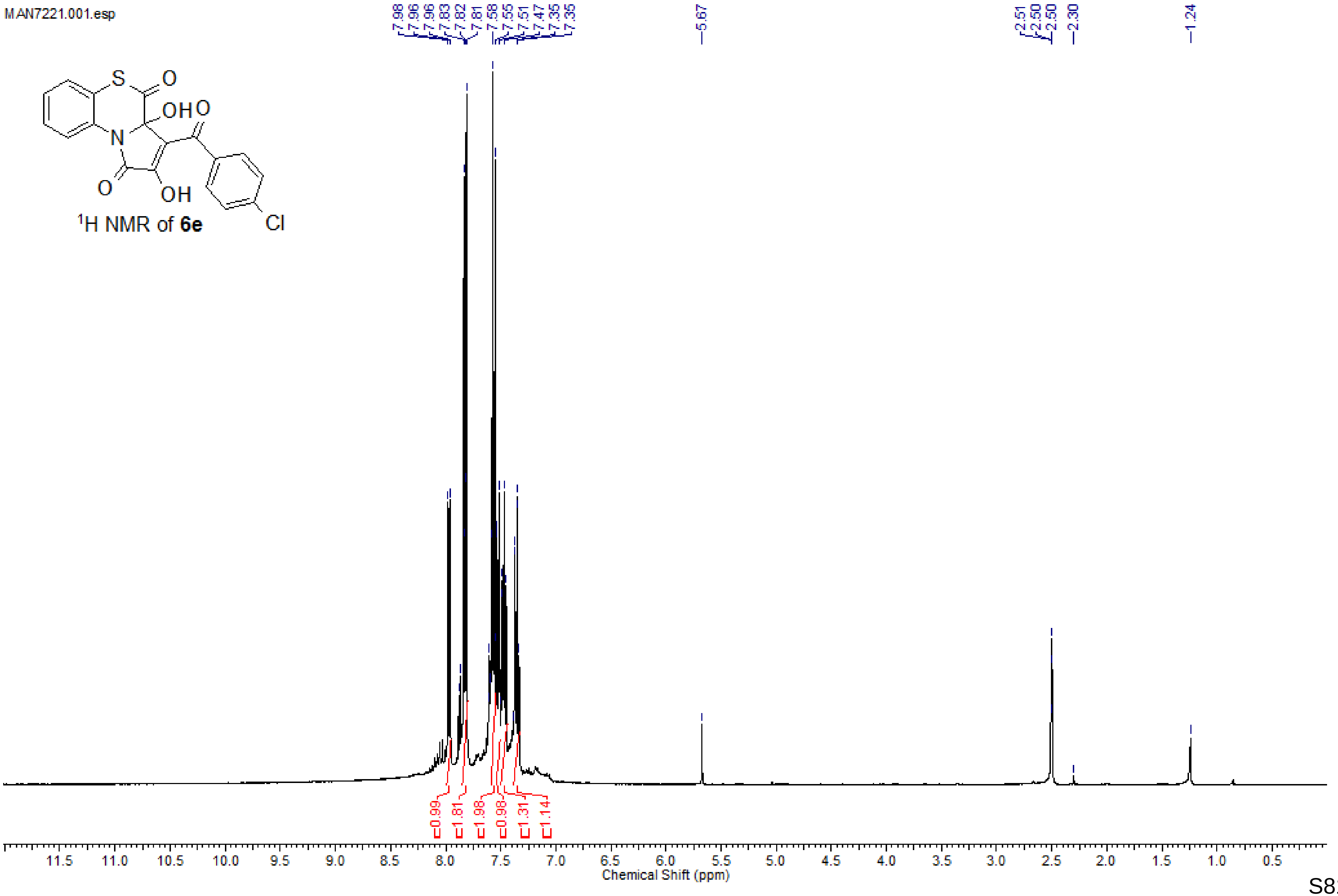

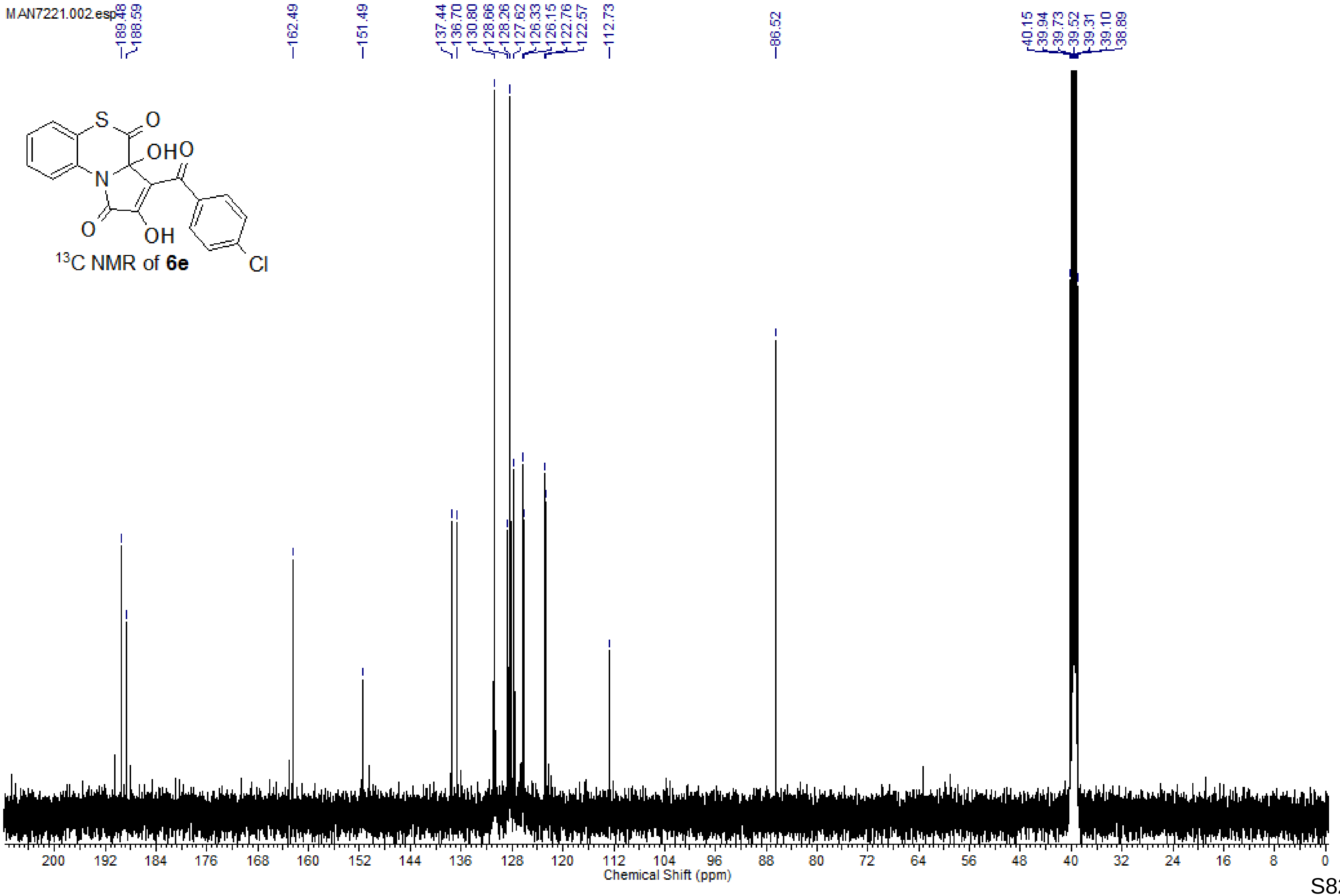

MAN7221.002.esp
189.48
188.59
162.49
151.49
137.44
136.70
130.80
128.66
128.26
127.62
126.33
126.15
122.76
122.57
112.73
86.52
40.15
39.94
39.73
39.52
39.31
39.10
38.89
S
O
OH
O
N
O
OH
Cl
^{13}C NMR of **6e**
200 192 184 176 168 160 152 144 136 128 120 112 104 96 88 80 72 64 56 48 40 32 24 16 8 0
Chemical Shift (ppm)

MAN7221.003.esp

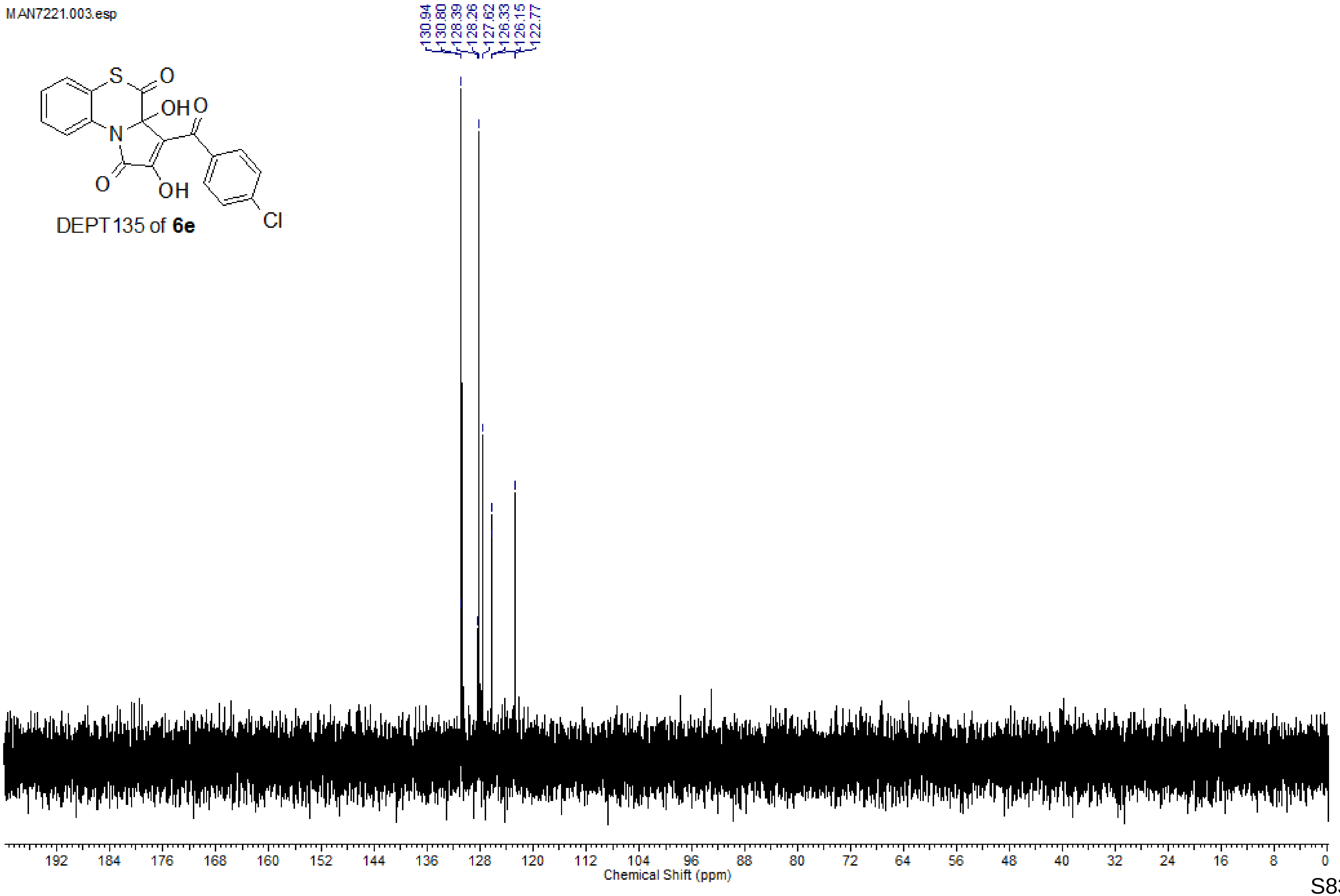

3-Aroyl-*N*-benzyl-2-hydroxy-1-oxopyrrolo[2,1-*b*][1,3]benzothiazole-3a(1*H*)-carboxamides 7. General procedure.

A mixture of APBTT **1** (0.45 mmol), benzylamine (0.49 mmol, 54 µL), and anhydrous toluene (3 mL) is stirred in a closed microreaction V-vial at 113 °C for 3 h. Then, the resulting solution is cooled to room temperature. After 24 h, the formed precipitate of compound **7** is filtered off and recrystallized from toluene.

3-Benzoyl-*N*-benzyl-2-hydroxy-1-oxopyrrolo[2,1-*b*][1,3]benzothiazole-3a(1*H*)-carboxamide (7a)
Yellow solid; yield (88 mg, 44%); mp 203–205 °C (toluene).
^{1}H NMR (400 MHz, DMSO-d_6): δ = 8.90 (m, 1H), 8.31 (br.s, 1H), 7.77 (m, 2H), 7.63 (m, 1H), 7.50 (m, 3H), 7.40 (m, 1H), 7.26–7.18 (m, 2H), 7.11 (m, 3H), 7.00 (m, 2H), 4.23 (m, 2H) ppm.
^{13}C NMR (100 MHz, DMSO-d_6): δ = 187.9, 166.8, 166.4, 154.0, 138.7, 137.3, 134.5, 133.8, 132.6, 128.8 (2C), 128.0 (2C), 127.9 (2C), 126.5, 126.5, 126.5, 126.4 (2C), 125.5, 123.0, 118.0, 80.5, 42.9 ppm.
IR (mineral oil): 3338, 3085, 1789, 1742, 1644 cm^{-1}.
EA: Found: C, 68.03; H, 4.15; N, 6.31. Calc. for $C_{25}H_{18}N_2O_4S$: C, 67.86; H, 4.10; N, 6.33.

***N*-Benzyl-2-hydroxy-3-(4-methylbenzoyl)-1-oxopyrrolo[2,1-*b*][1,3]benzothiazole-3a(1*H*)-carboxamide (7b)**
Yellow solid; yield (144 mg, 70%); mp 199–201 °C (toluene).
^{1}H NMR (400 MHz, DMSO-d_6): δ = 8.88 (m, 1H), 7.70 (m, 2H), 7.50 (m, 1H), 7.39 (m, 1H), 7.32 (m, 2H), 7.25-7.18 (m, 2H), 7.10 (m, 3H), 6.99 (m, 2H), 4.22 (m, 2H), 2.41 (s, 3H) ppm.
^{13}C NMR (100 MHz, DMSO-d_6): δ = 187.7, 166.6, 166.4, 152.4, 143.4, 138.7, 134.7, 134.5, 133.7, 129.0 (2C), 128.7 (2C), 127.9 (2C), 126.6, 126.5, 126.4 (2C), 125.5, 123.0, 119.1, 118.1, 80.6, 42.9, 21.1 ppm.
IR (mineral oil): 3307, 3090, 1779, 1741, 1662 cm^{-1}.
EA: Found: C, 68.51; H, 4.48; N, 6.21. Calc. for $C_{26}H_{20}N_2O_4S$: C, 68.41; H, 4.42; N, 6.14.

***N*-Benzyl-2-hydroxy-3-(4-methoxybenzoyl)-1-oxopyrrolo[2,1-*b*][1,3]benzothiazole-3a(1*H*)-carboxamide (7c)**
Yellow solid; yield (83 mg, 39%); mp 202–204 °C (toluene).
^{1}H NMR (400 MHz, DMSO-d_6): δ = 8.86 (s, 1H), 7.81 (m, 2H), 7.50 (m, 1H), 7.39 (m, 1H), 7.22 (m, 2H), 7.05 (m, 8H), 4.21 (m, 2H), 3.87 (s, 3H) ppm.
^{13}C NMR (100 MHz, DMSO-d_6): δ = 186.6, 166.6 (2C), 163.3, 151.2, 138.7, 135.0, 133.7, 131.5 (2C), 129.6, 127.9 (2C), 126.5, 126.5, 126.4 (2C), 125.5, 123.0, 119.4, 118.1, 113.5 (2C), 80.7, 55.5, 42.8 ppm.
IR (mineral oil): 3327, 3185, 1742, 1693, 1648 cm^{-1}.
EA: Found: C, 66.31; H, 4.34; N, 6.00. Calc. for $C_{26}H_{20}N_2O_5S$: C, 66.09; H, 4.27; N, 5.93.

_N_-Benzyl-3-(4-fluorobenzoyl)-2-hydroxy-1-oxopyrrolo[2,1-_b_][1,3]benzothiazole-3a(1_H_)-carboxamide (7d)
Yellow solid; yield (102 mg, 49%); mp 215–217 °C (toluene).
^{1}H NMR (400 MHz, DMSO-d_6): δ = 8.87 (m, 1H), 7.85 (m, 2H), 7.49 (m, 1H), 7.38 (m, 1H), 7.31 (m, 2H), 7.21 (m, 2H), 7.12 (m, 3H), 7.00 (m, 2H), 4.21 (m, 2H) ppm.
^{13}C NMR (100 MHz, DMSO-d_6): δ = 186.5, 166.6, 166.3, 166.0, 163.5, 153.8, 138.7, 134.6, 133.8, 133.8, 133.7, 131.8, 131.7, 127.9 (2C), 126.6, 126.5, 126.4 (2C), 125.5, 123.1, 118.3, 118.0, 115.3, 115.0, 80.6, 42.9 ppm.
IR (mineral oil): 3326, 3172, 1741, 1658, 1647 cm^{-1}.
EA: Found: C, 65.44; H, 3.87; N, 6.15. Calc. for $C_{25}H_{17}FN_2O_4S$: C, 65.21; H, 3.72; N, 6.08.

_N_-Benzyl-3-(4-chlorobenzoyl)-2-hydroxy-1-oxopyrrolo[2,1-_b_][1,3]benzothiazole-3a(1_H_)-carboxamide (7e)
Yellow solid; yield (93 mg, 43%); mp 217–219 °C (toluene).
^{1}H NMR (400 MHz, DMSO-d_6): δ = 8.89 (m, 1H), 7.78 (m, 2H), 7.56 (m, 2H), 7.49 (m, 1H), 7.39 (m, 1H), 7.25–7.12 (m, 5H), 7.00 (m, 2H), 4.22 (m, 2H) ppm.
^{13}C NMR (100 MHz, DMSO-d_6): δ = 186.6, 166.8, 166.3, 155.0, 138.7, 137.3, 136.1, 134.4, 133.8, 130.6 (2C), 128.2 (2C), 127.9 (2C), 126.5, 126.5, 126.4 (2C), 125.5, 123.1, 117.9, 117.2, 80.5, 42.9 ppm.
IR (mineral oil): 3310, 3088, 1784, 1739, 1659 cm^{-1}.
EA: Found: C, 63.14; H, 3.64 N, 5.92. Calc. for $C_{25}H_{17}ClN_2O_4S$: C, 62.96; H, 3.59; N, 5.87.

_N_-Benzyl-3-(4-bromobenzoyl)-2-hydroxy-1-oxopyrrolo[2,1-_b_][1,3]benzothiazole-3a(1_H_)-carboxamide (7f)
Yellow solid; yield (153 mg, 65%); mp 210–212 °C (toluene).
^{1}H NMR (400 MHz, DMSO-d_6): δ = 8.87 (m, 1H), 7.69 (m, 4H), 7.50 (m, 1H), 7.38 (m, 1H), 7.21 (m, 2H), 7.13 (3H), 7.02 (m, 2H), 6.63 (br.s, 1H), 4.21 (m, 2H) ppm.
^{13}C NMR (100 MHz, DMSO-d_6): δ = 186.7, 166.7, 166.2, 155.0, 138.7, 136.4, 134.4, 133.8, 131.1 (2C), 130.7 (2C), 127.9 (2C), 126.5, 126.5, 126.5, 126.4 (2C), 125.5, 123.1, 117.9, 117.7, 80.5, 42.9 ppm.
IR (mineral oil): 3311, 3105, 1751, 1709, 1670 cm^{-1}.
EA: Found: C, 57.73; H, 3.42 N, 5.40. Calc. for $C_{25}H_{17}BrN_2O_4S$: C, 57.59; H, 3.29; N, 5.37.

_N_-Benzyl-3-(furan-2-ylcarbonyl)-2-hydroxy-1-oxopyrrolo[2,1-_b_][1,3]benzothiazole-3a(1_H_)-carboxamide (7g)
Yellow solid; yield (35 mg, 18%); mp 152–154 °C (toluene).
^{1}H NMR (400 MHz, DMSO-d_6): δ = 8.86 (m, 1H), 8.61 (br.s, 1H), 8.04 (m, 1H), 7.79 (m, 1H), 7.49 (m, 1H), 7.38 (m, 1H), 7.20 (m, 2H), 7.13 (m, 3H), 6.99 (m, 2H), 6.76 (m, 1H), 4.21 (m, 2H) ppm.

^{13}C NMR (100 MHz, DMSO-d_6): δ = 173.0, 166.7, 166.2, 153.5, 151.2, 148.0, 138.7, 134.4, 133.8, 127.9 (2C), 126.5, 126.5, 126.3 (2C), 125.5, 123.0, 120.2, 118.1, 117.8, 112.3, 80.5, 42.9 ppm.
IR (mineral oil): 3318, 3110, 1775, 1720, 1660 cm^{-1}.
EA: Found: C, 64.11; H, 3.91 N, 6.63. Calc. for $C_{23}H_{16}N_2O_5S$: C, 63.88; H, 3.73; N, 6.48.

***N*-Benzyl-2-hydroxy-1-oxo-3-(thiophen-2-ylcarbonyl)pyrrolo[2,1-*b*][1,3]benzothiazole-3a(1*H*)-carboxamide (7h)**
Yellow solid; yield (40 mg, 20%); mp 187–186 °C (toluene).
^{1}H NMR (400 MHz, DMSO-d_6): δ = 9.30 (br.s, 1H), 8.86 (m, 1H), 8.30 (m, 1H), 8.03 (m, 1H), 7.50 (m, 1H), 7.38 (m, 1H), 7.27 (m, 1H), 7.24–7.17 (m, 2H), 7.11 (m, 3H), 6.98 (m, 2H), 4.21 (m, 2H) ppm.
^{13}C NMR (100 MHz, DMSO-d_6): δ = 178.6, 166.8, 166.3, 153.5, 143.6, 138.7, 135.2, 134.5, 134.0, 133.8, 128.4, 127.9 (2C), 126.5, 126.4, 126.3 (2C), 125.5, 123.0, 118.2, 117.9, 80.7, 42.9 ppm.
IR (mineral oil): 3333, 3170, 1785, 1743, 1659 cm^{-1}.
EA: Found: C, 61.72; H, 3.69; N, 6.32. Calc. for $C_{23}H_{16}N_2O_4S_2$: C, 61.59; H, 3.60; N, 6.25.

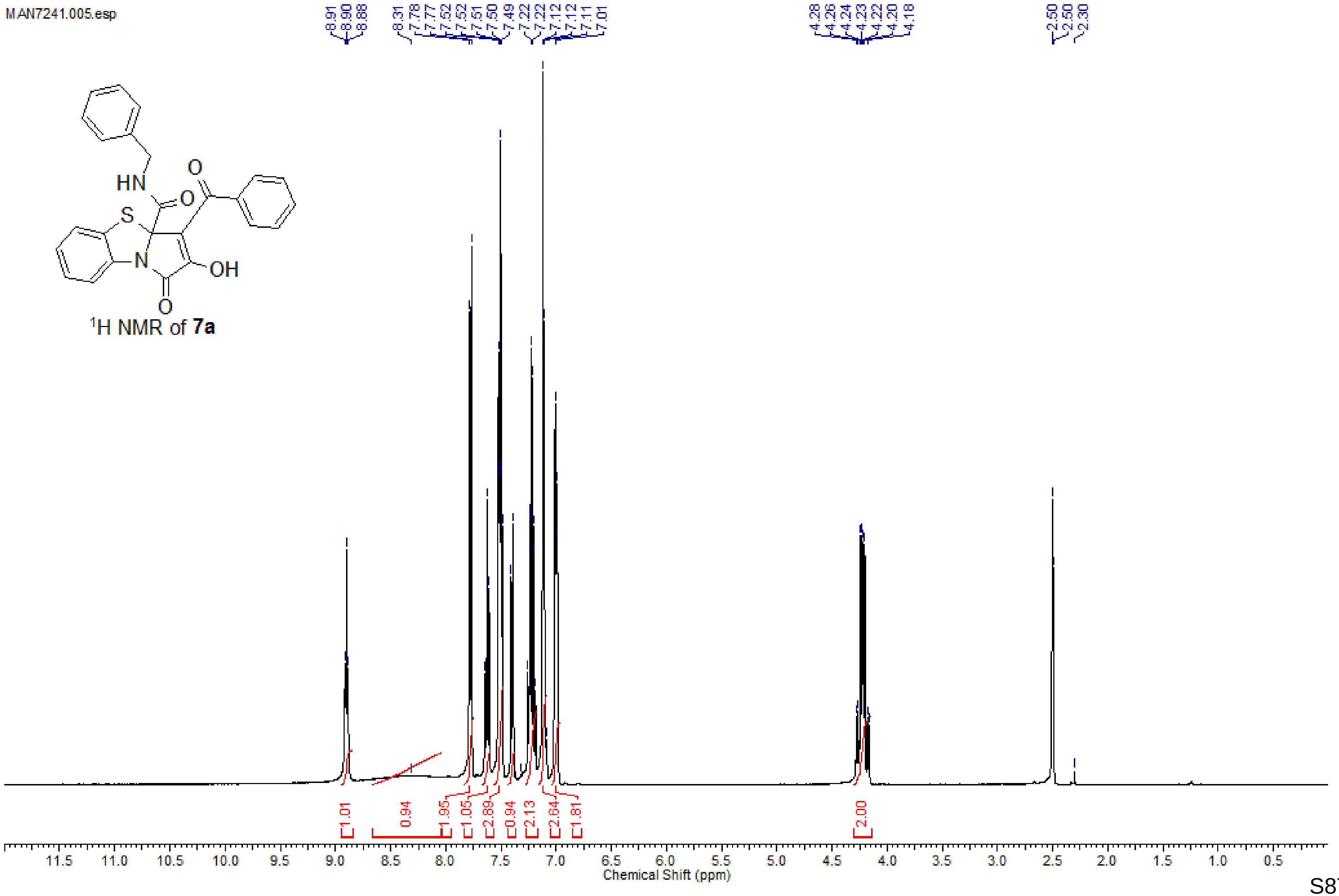
MAN7241.005.esp
HN
O
O
S
N
OH
O
8.91
8.90
8.88
8.31
7.78
7.77
7.52
7.52
7.51
7.50
7.49
7.22
7.22
7.12
7.12
7.11
7.01
4.28
4.26
4.24
4.23
4.22
4.20
4.18
2.50
2.50
2.30
1.01
0.94
1.95
1.05
2.89
0.94
2.13
2.64
1.81
2.00
11.5
11.0
10.5
10.0
9.5
9.0
8.5
8.0
7.5
7.0
6.5
6.0
5.5
5.0
4.5
4.0
3.5
3.0
2.5
2.0
1.5
1.0
0.5
Chemical Shift (ppm)

^{1}H NMR of **7a**

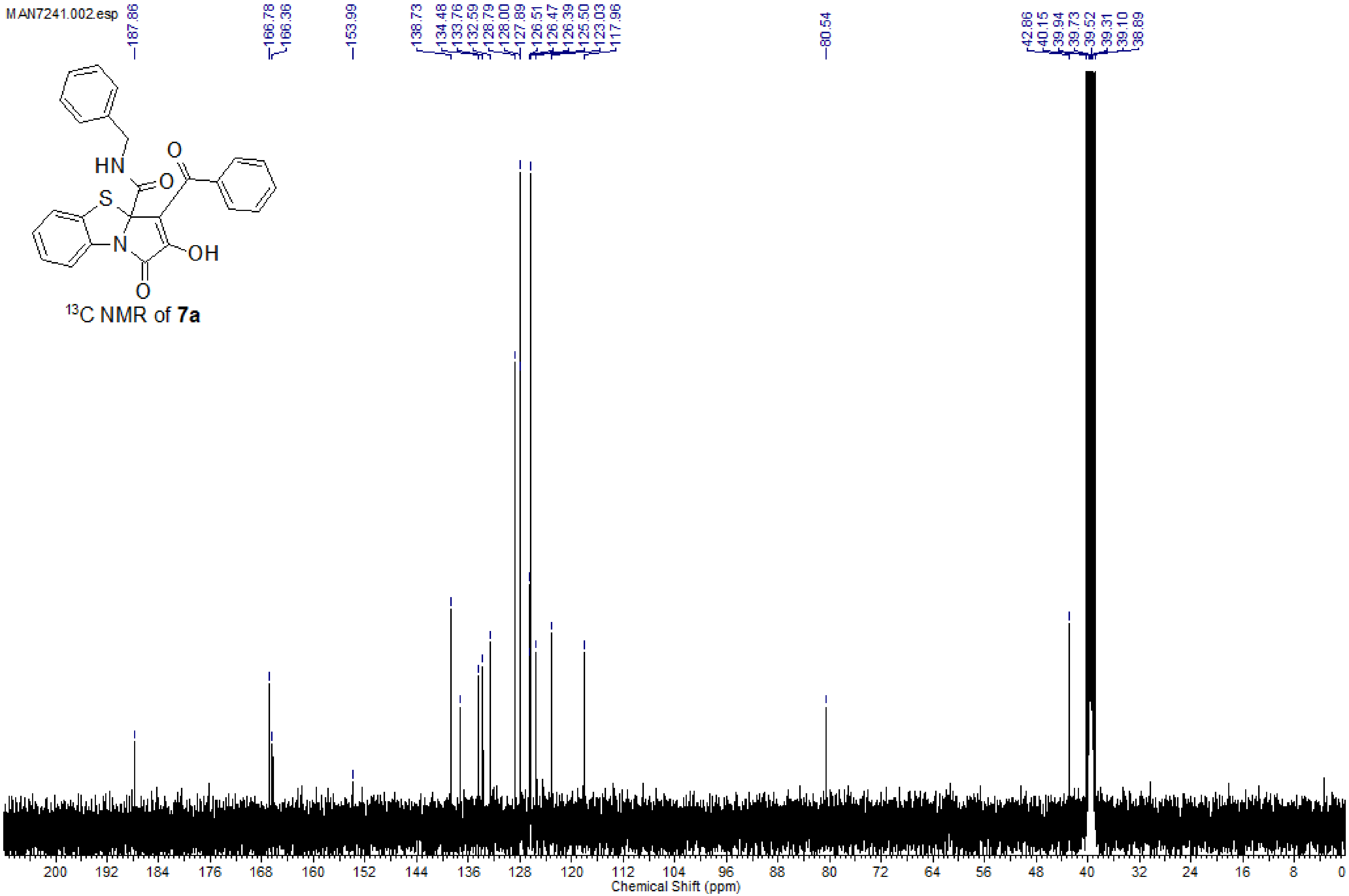
MAN7241.002.esp
187.86
166.78
166.36
153.99
138.73
134.48
133.76
132.59
128.79
128.00
127.89
126.51
126.47
126.39
125.50
123.03
117.96
80.54
42.86
40.15
39.94
39.73
39.52
39.31
39.10
38.89
HN
O
O
S
N
OH
O
200 192 184 176 168 160 152 144 136 128 120 112 104 96 88 80 72 64 56 48 40 32 24 16 8 0
Chemical Shift (ppm)
^{13}C NMR of **7a**

MAN7241.003.esp

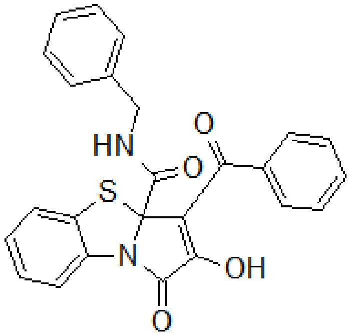

DEPT135 of **7a**

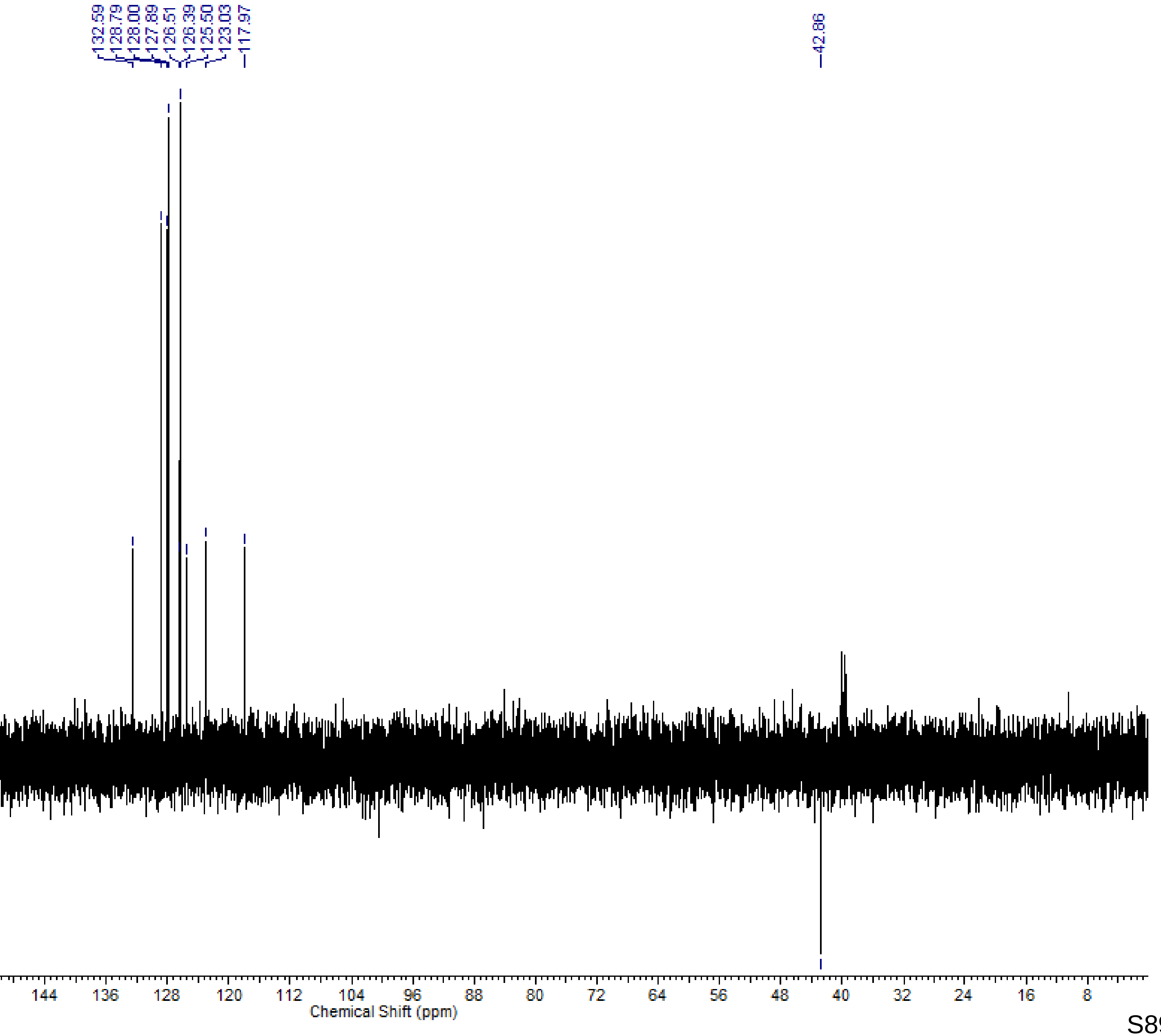

MAN7253.001.esp

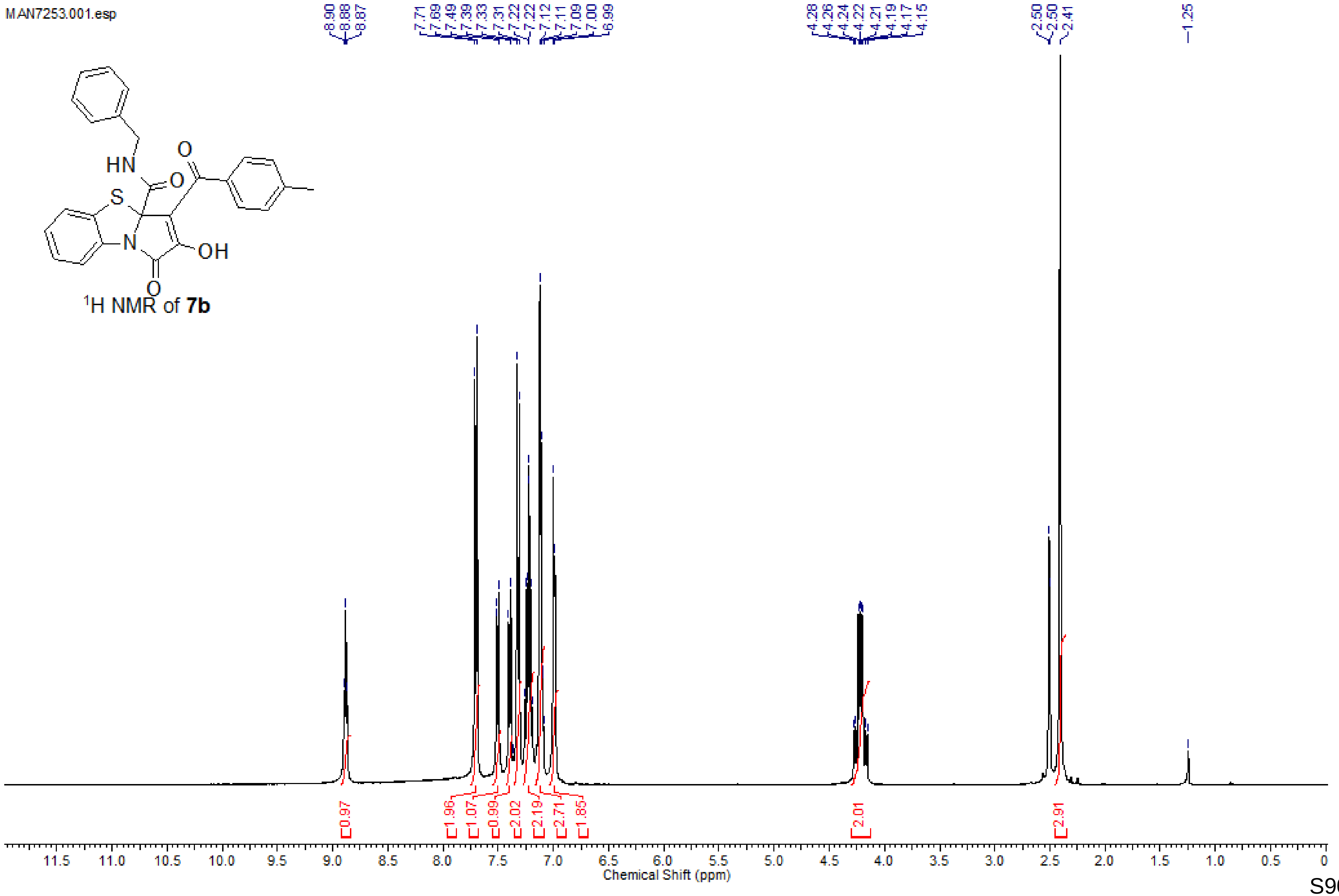

^{1}H NMR of **7b**

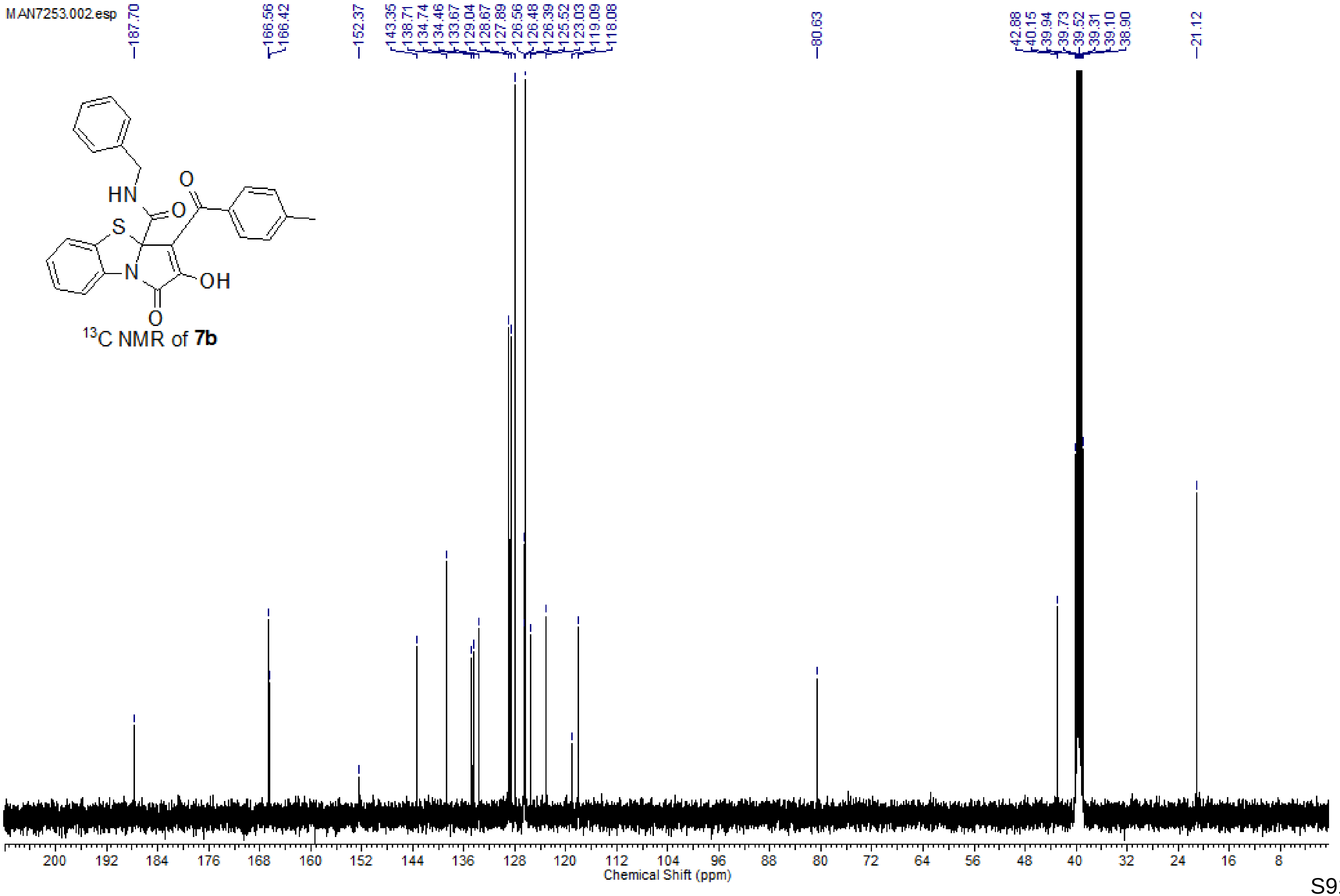

MAN7253.002.esp
187.70
166.56
166.42
152.37
143.35
138.71
134.74
134.46
133.67
129.04
128.67
127.89
126.56
126.48
126.39
125.52
123.03
119.09
118.08
80.63
42.88
40.15
39.94
39.73
39.52
39.31
39.10
38.90
21.12
HN
O
O
S
N
OH
O
Chemical Shift (ppm)

^{13}C NMR of **7b**

MAN7253.003.esp

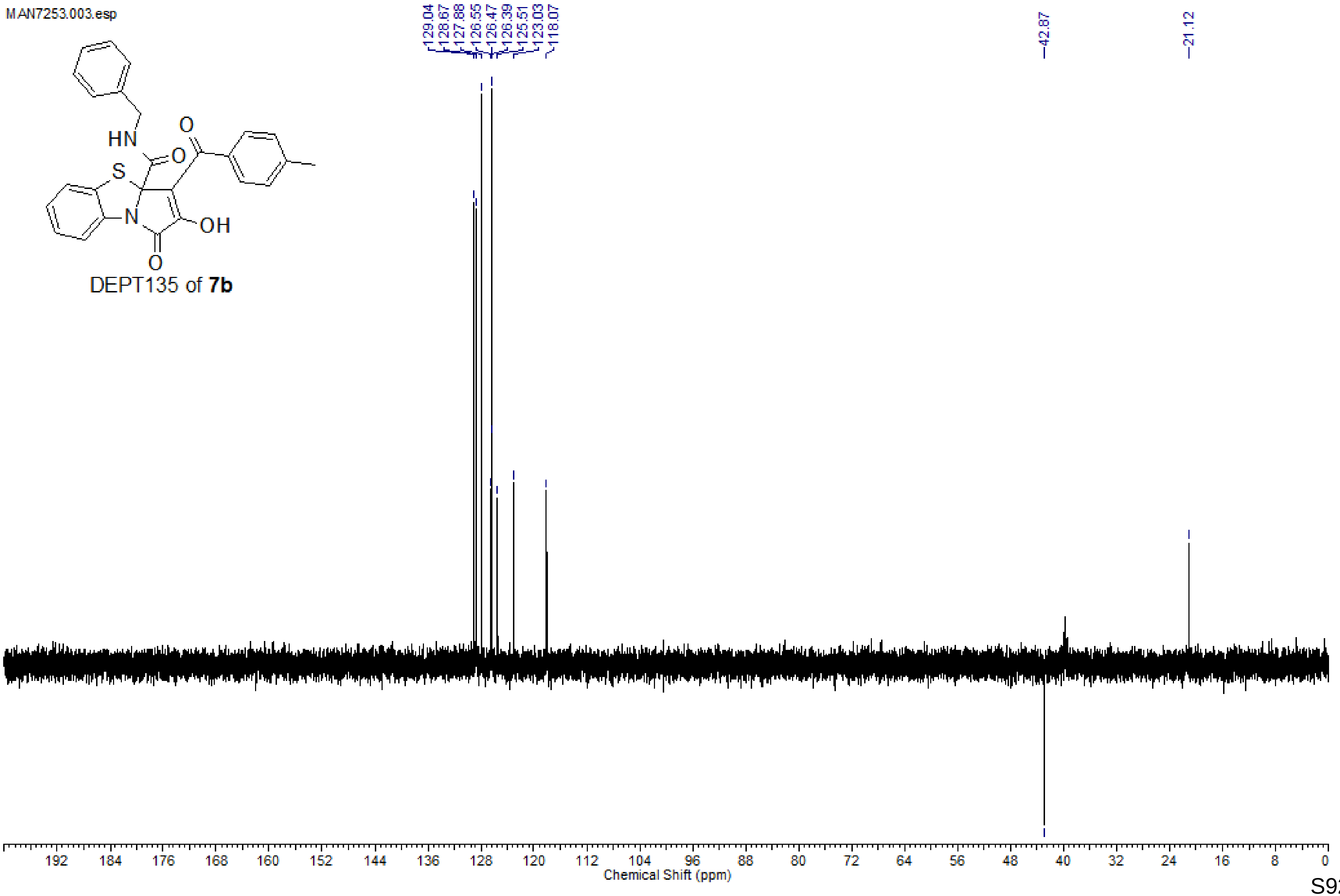

DEPT135 of **7b**

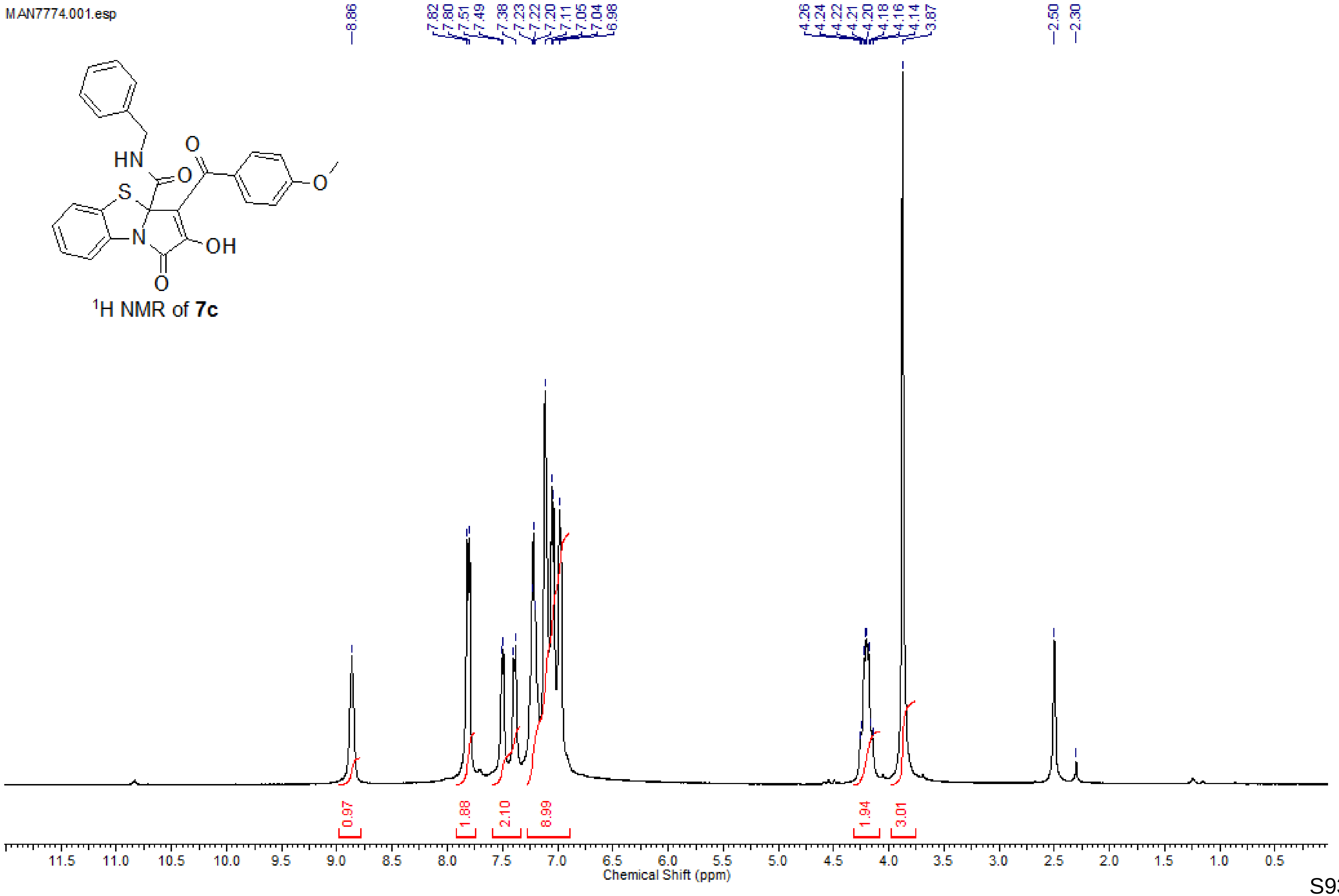

MAN7774.001.esp
HN
O
O
S
N
OH
O
O
8.86
7.82
7.80
7.51
7.49
7.38
7.23
7.22
7.20
7.11
7.05
7.04
6.98
4.26
4.24
4.22
4.21
4.20
4.18
4.16
4.14
3.87
2.50
2.30
0.97
1.88
2.10
8.99
1.94
3.01
11.5
11.0
10.5
10.0
9.5
9.0
8.5
8.0
7.5
7.0
6.5
6.0
5.5
5.0
4.5
4.0
3.5
3.0
2.5
2.0
1.5
1.0
0.5
Chemical Shift (ppm)

^{1}H NMR of **7c**

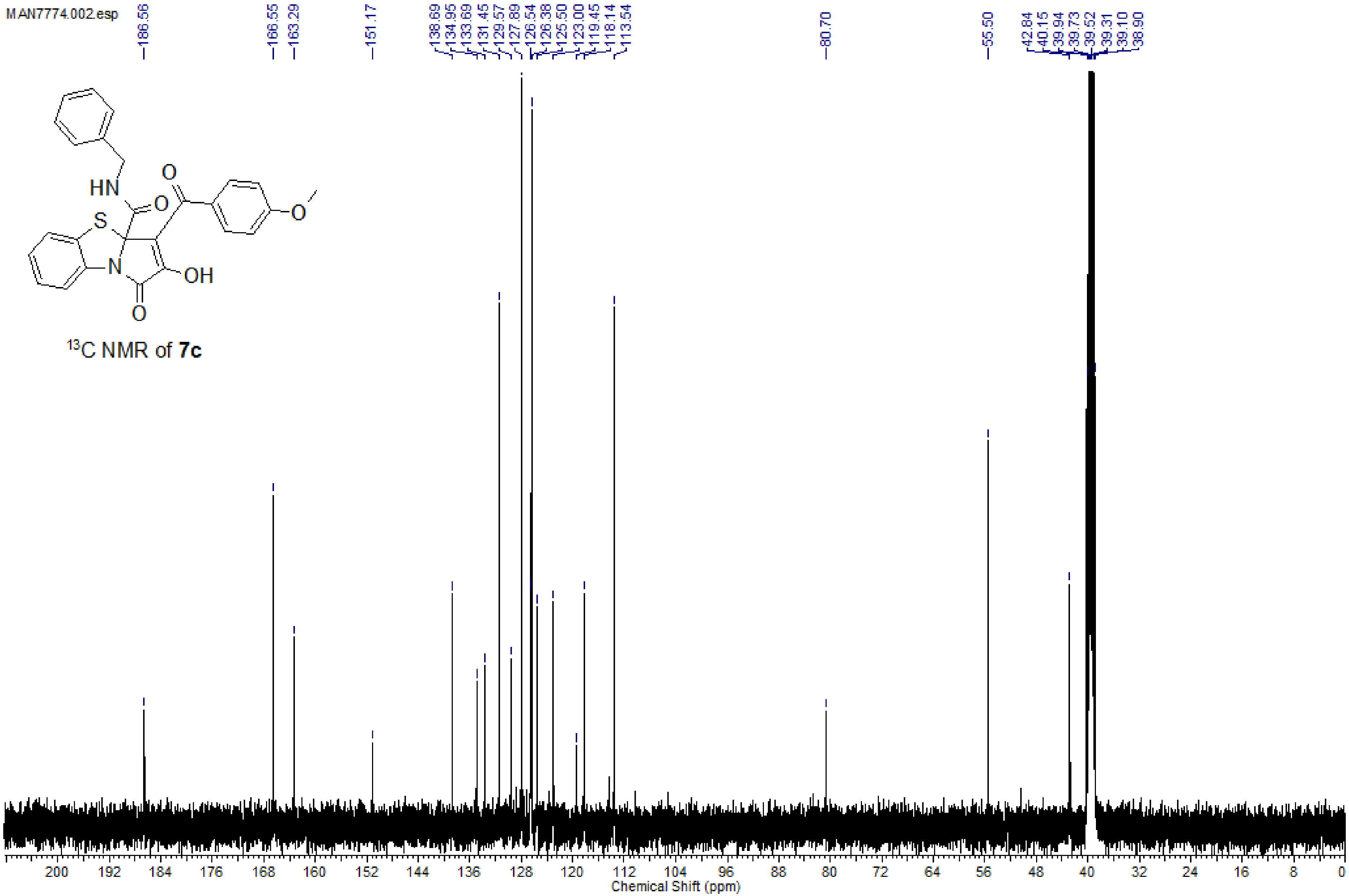
MAN7774.002.esp
186.56
166.55
163.29
151.17
138.69
134.95
133.69
131.45
129.57
127.89
126.54
126.38
125.50
123.00
119.45
118.14
113.54
80.70
55.50
42.84
40.15
39.94
39.73
39.52
39.31
39.10
38.90
HN
O
O
S
N
OH
O
O
200
192
184
176
168
160
152
144
136
128
120
112
104
96
88
80
72
64
56
48
40
32
24
16
8
0
Chemical Shift (ppm)

^{13}C NMR of **7c**

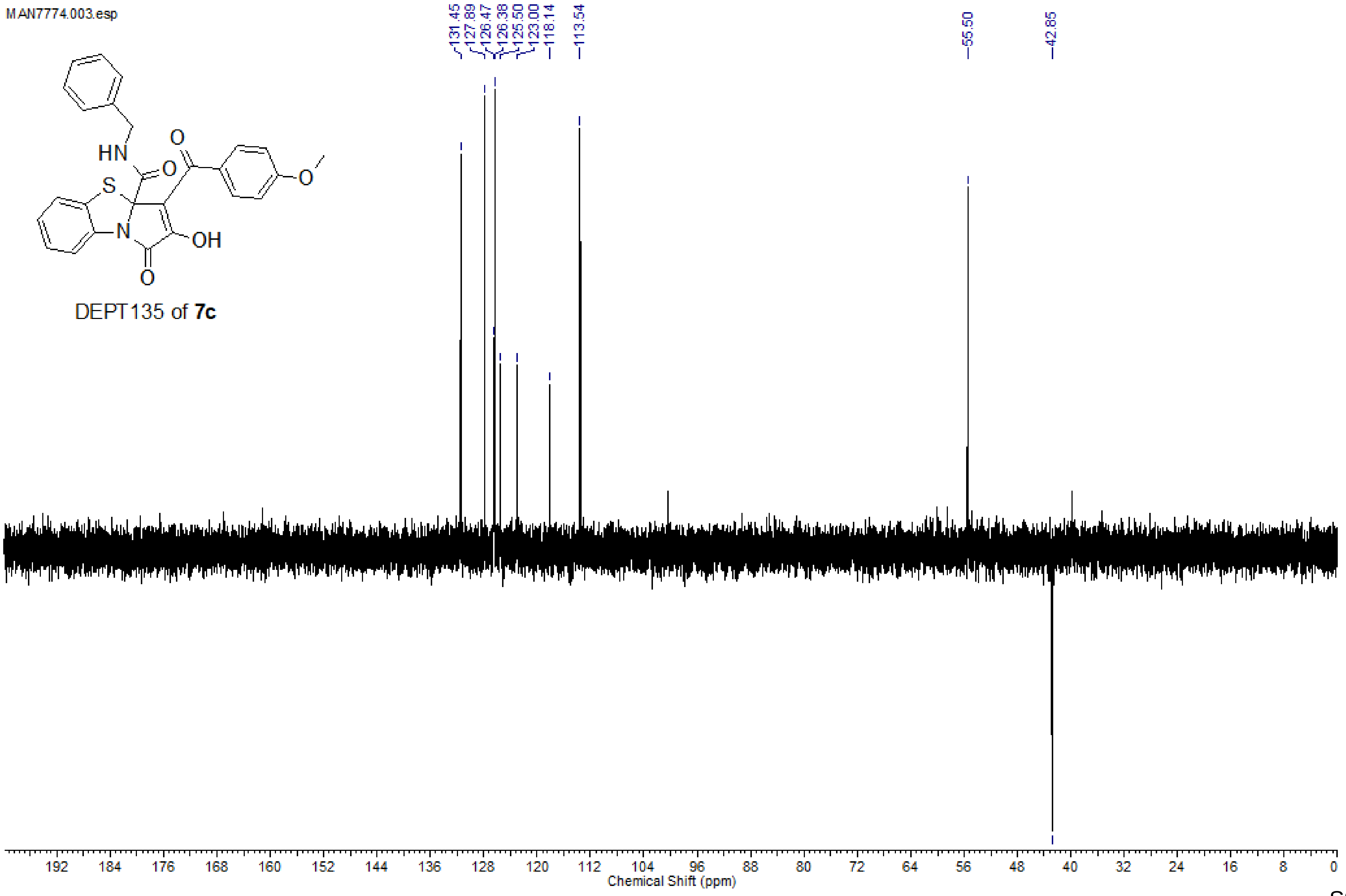
MAN7774.003.esp
DEPT135 of 7c
131.45
127.89
126.47
126.38
125.50
123.00
118.14
113.54
55.50
42.85
Chemical Shift (ppm)

MAN7897.004.esp

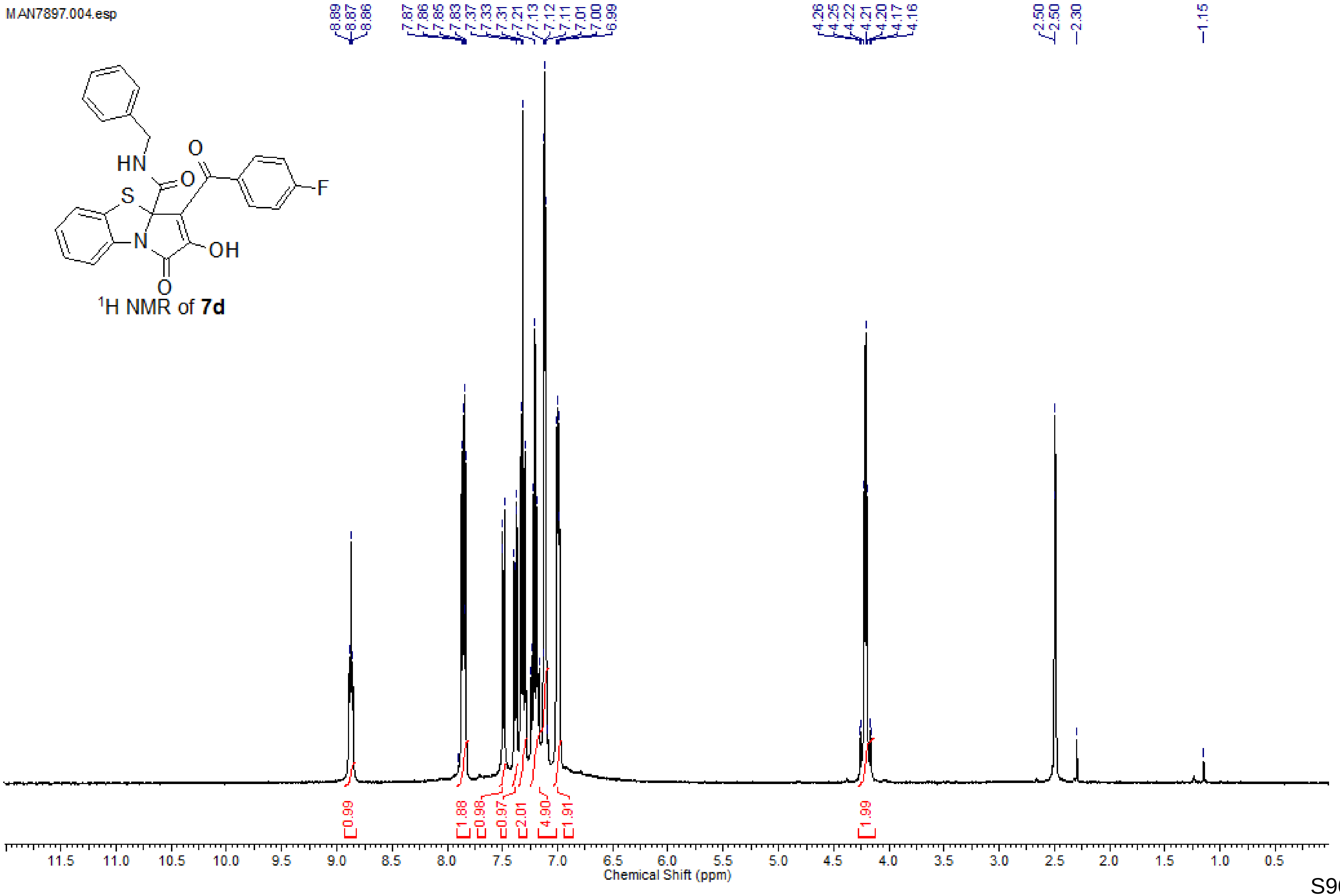

^{1}H NMR of **7d**

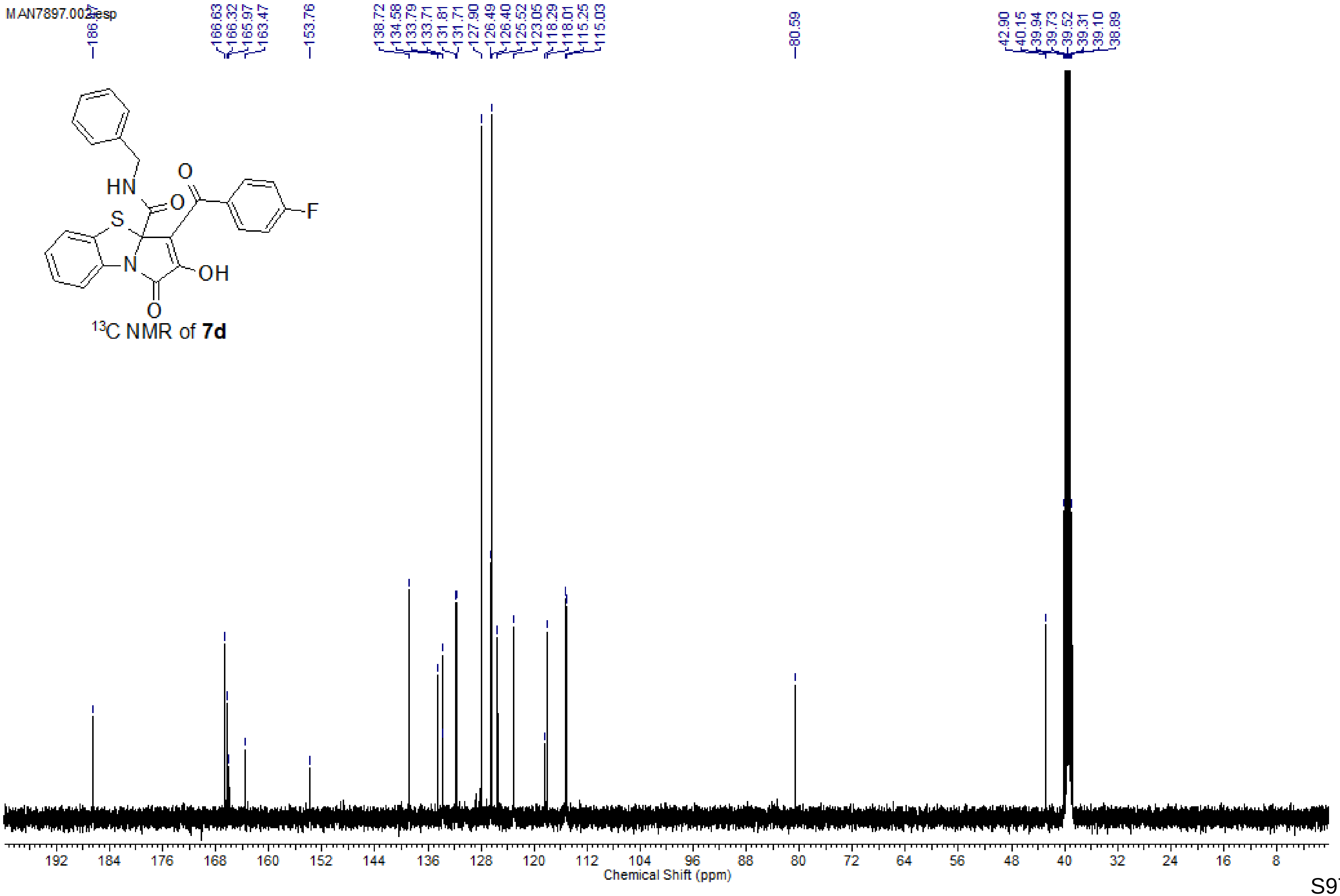

MAN7897.002.esp
166.63
166.32
165.97
163.47
153.76
138.72
134.58
133.79
133.71
131.81
131.71
127.90
126.49
126.40
125.52
123.05
118.29
118.01
115.25
115.03
80.59
42.90
40.15
39.94
39.73
39.52
39.31
39.10
38.89
HN
O
S
N
O
F
OH
192
184
176
168
160
152
144
136
128
120
112
104
96
88
80
72
64
56
48
40
32
24
16
8
Chemical Shift (ppm)

^{13}C NMR of **7d**

MAN7897.003.esp

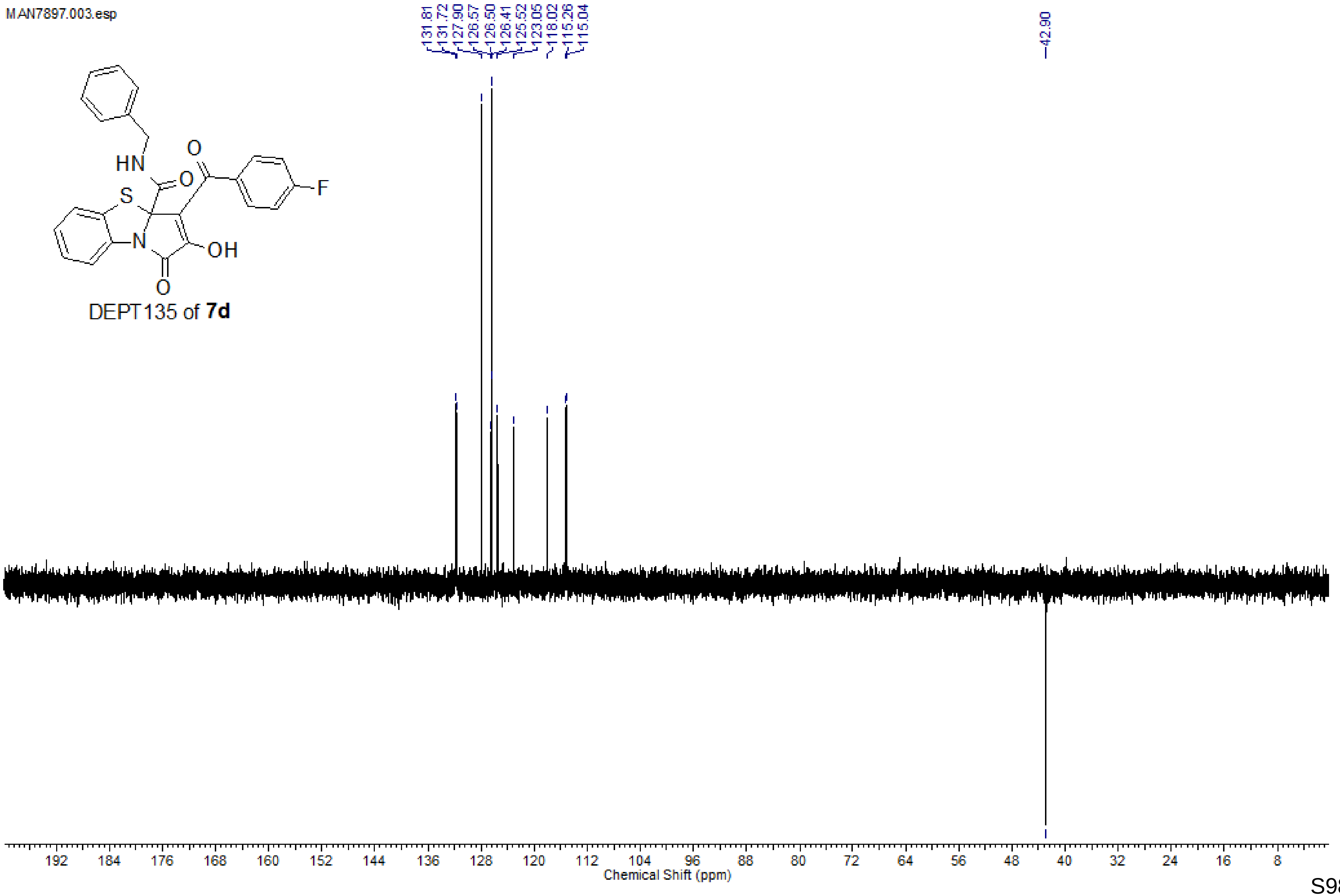

DEPT135 of **7d**

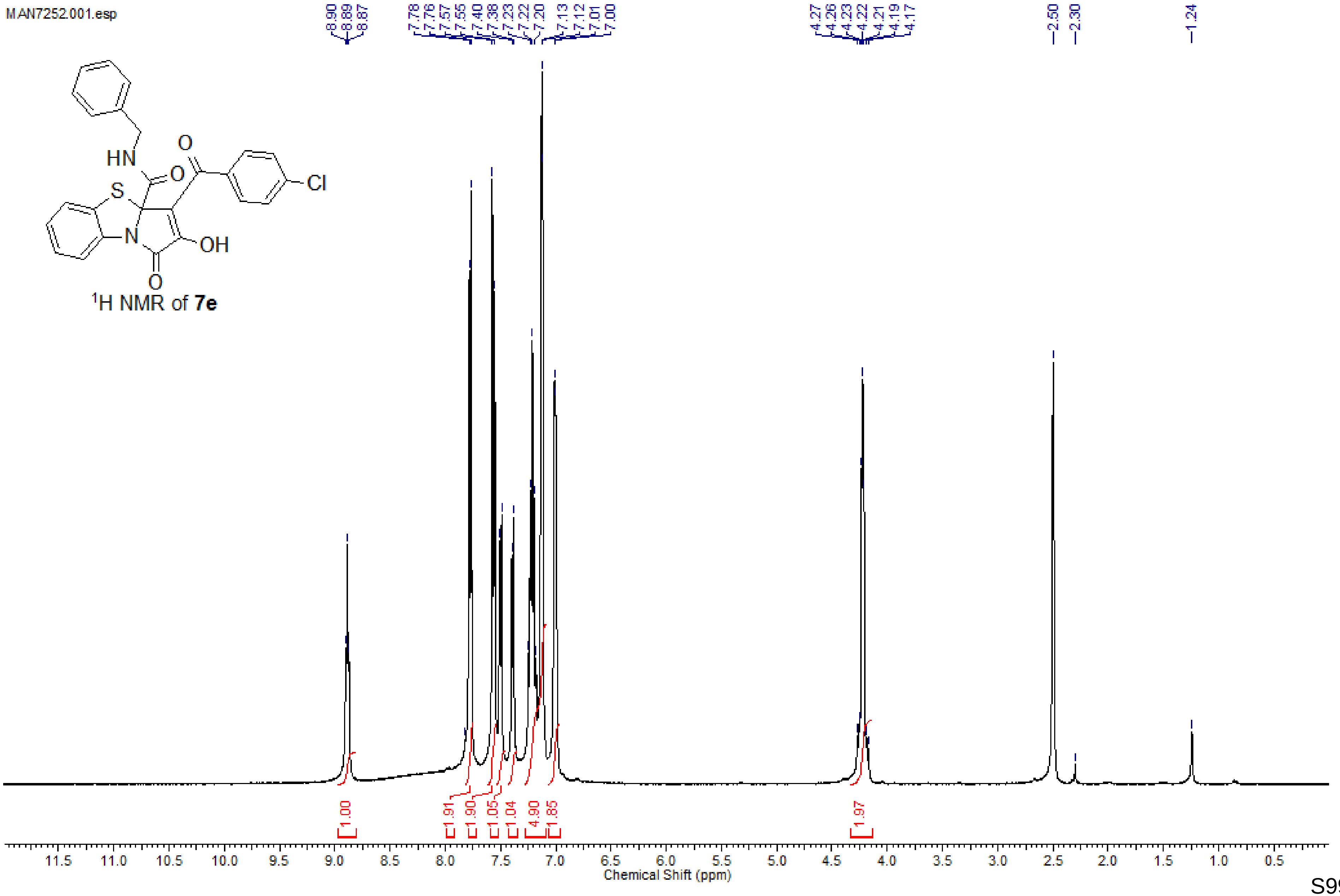
MAN7252.001.esp
8.90
8.89
8.87
7.78
7.76
7.57
7.55
7.40
7.38
7.23
7.22
7.20
7.13
7.12
7.01
7.00
4.27
4.26
4.23
4.22
4.21
4.19
4.17
2.50
2.30
1.24
HN
O
O
S
Cl
N
OH
O
1.00
1.91
1.90
1.05
1.04
4.90
1.85
1.97
11.5
11.0
10.5
10.0
9.5
9.0
8.5
8.0
7.5
7.0
6.5
6.0
5.5
5.0
4.5
4.0
3.5
3.0
2.5
2.0
1.5
1.0
0.5
Chemical Shift (ppm)

^{1}H NMR of **7e**

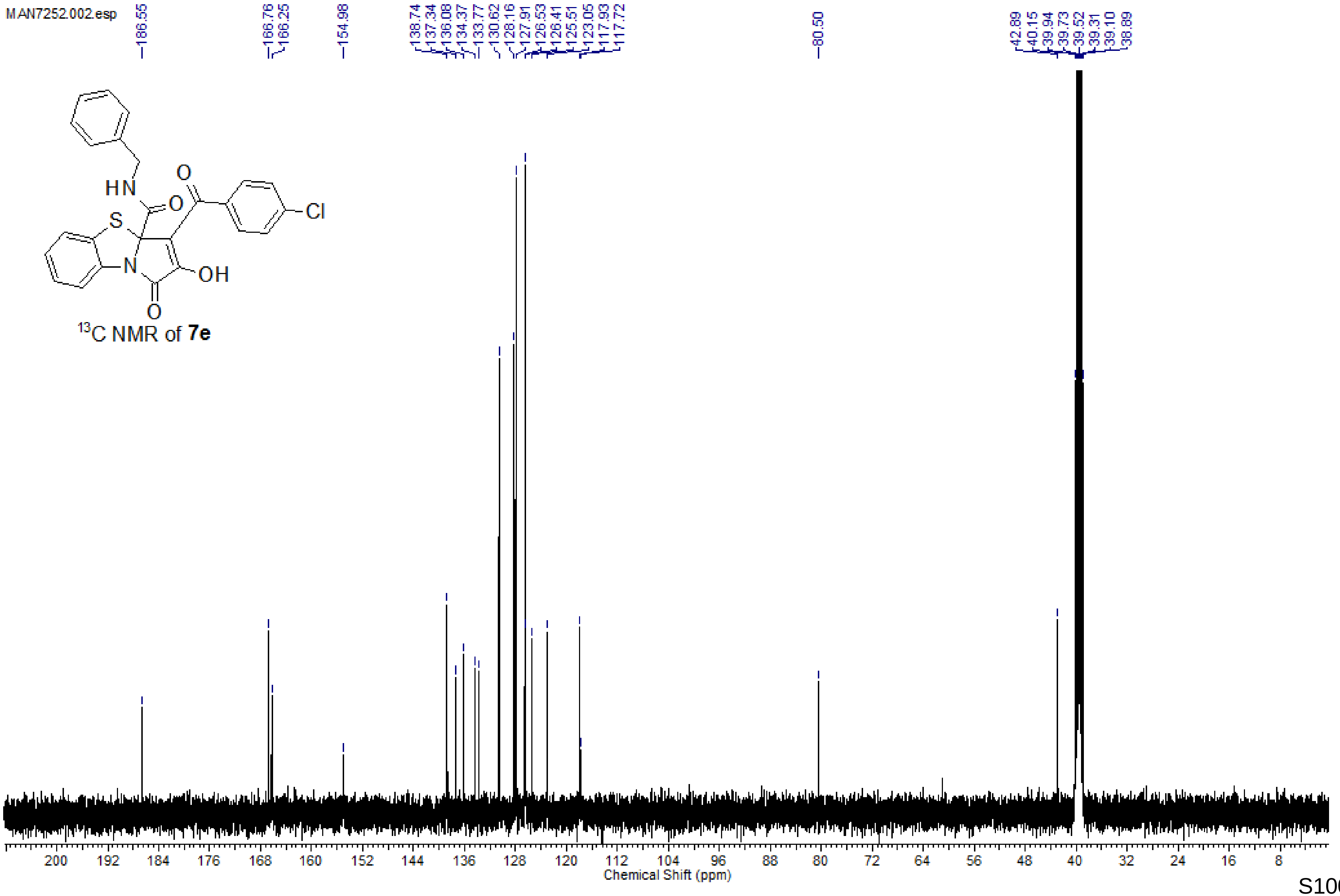

MAN7252.002.esp
186.55
166.76
166.25
154.98
138.74
137.34
136.08
134.37
133.77
130.62
128.16
127.91
126.53
126.41
125.51
123.05
117.93
117.72
80.50
42.89
40.15
39.94
39.73
39.52
39.31
39.10
38.89
HN
O
O
S
N
OH
O
Cl
200 192 184 176 168 160 152 144 136 128 120 112 104 96 88 80 72 64 56 48 40 32 24 16 8
Chemical Shift (ppm)

^{13}C NMR of **7e**

MAN7252.003.esp

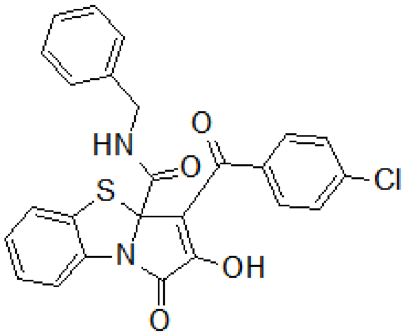

DEPT135 of **7e**

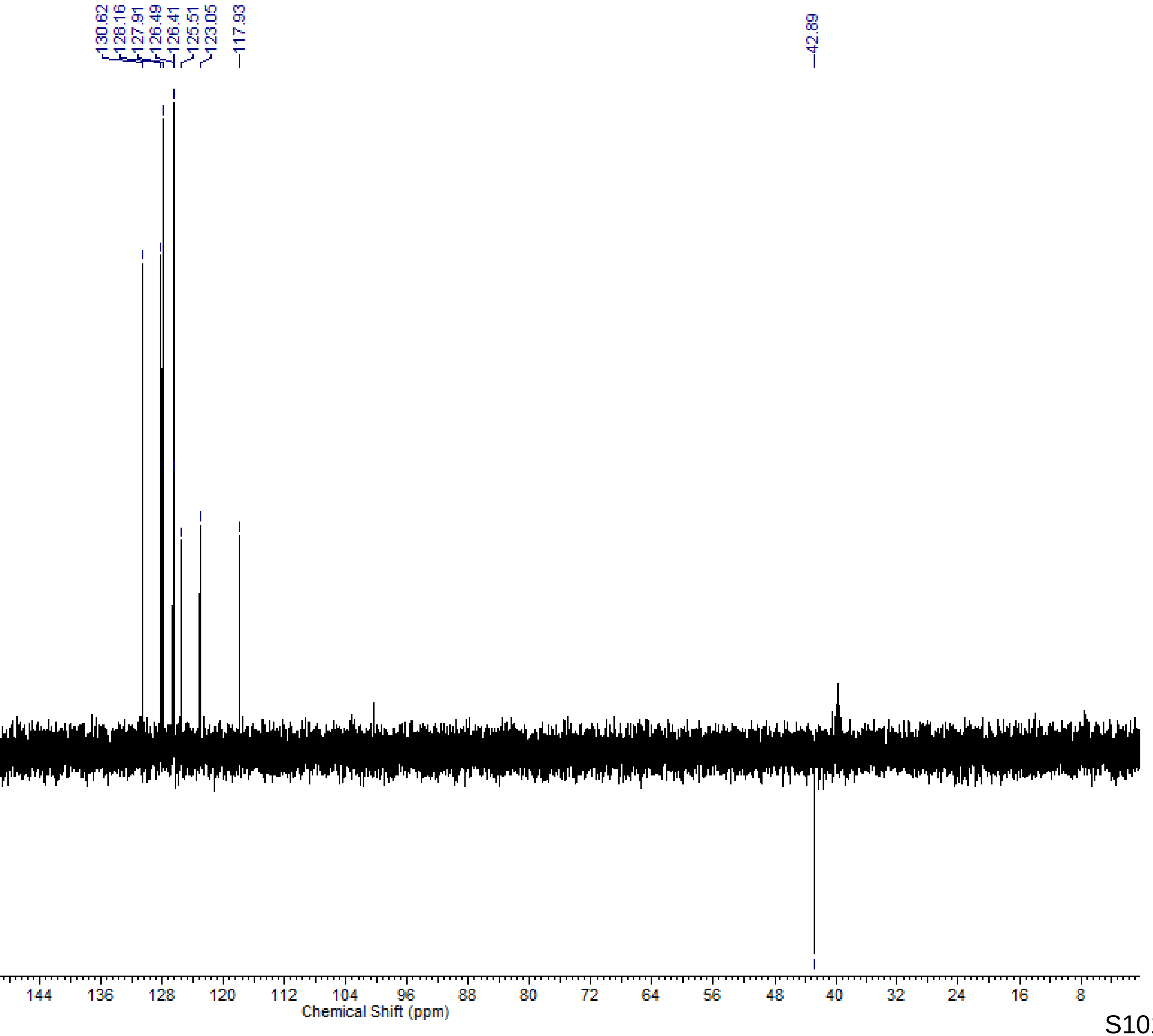

MAN7895.004.esp

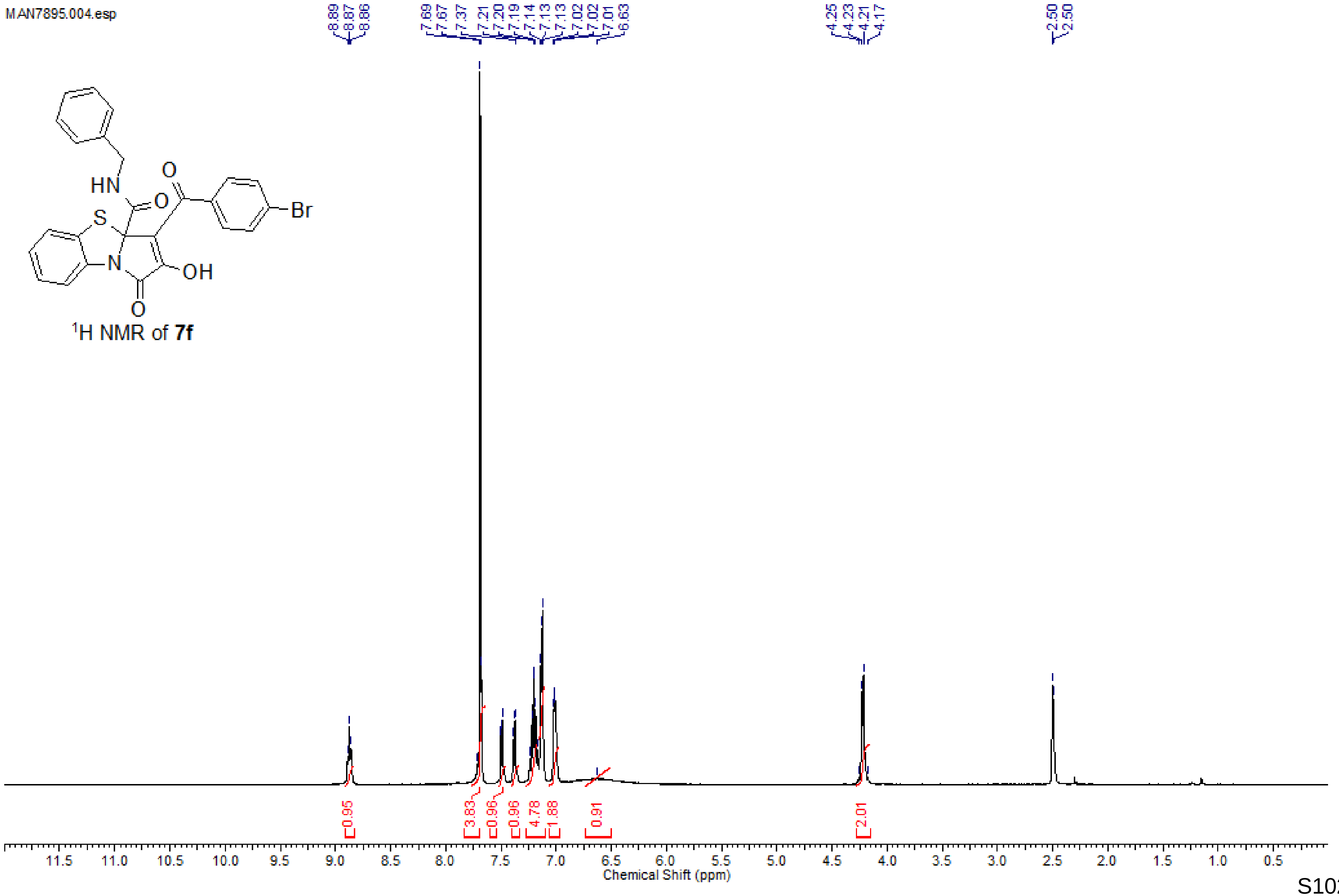

^{1}H NMR of **7f**

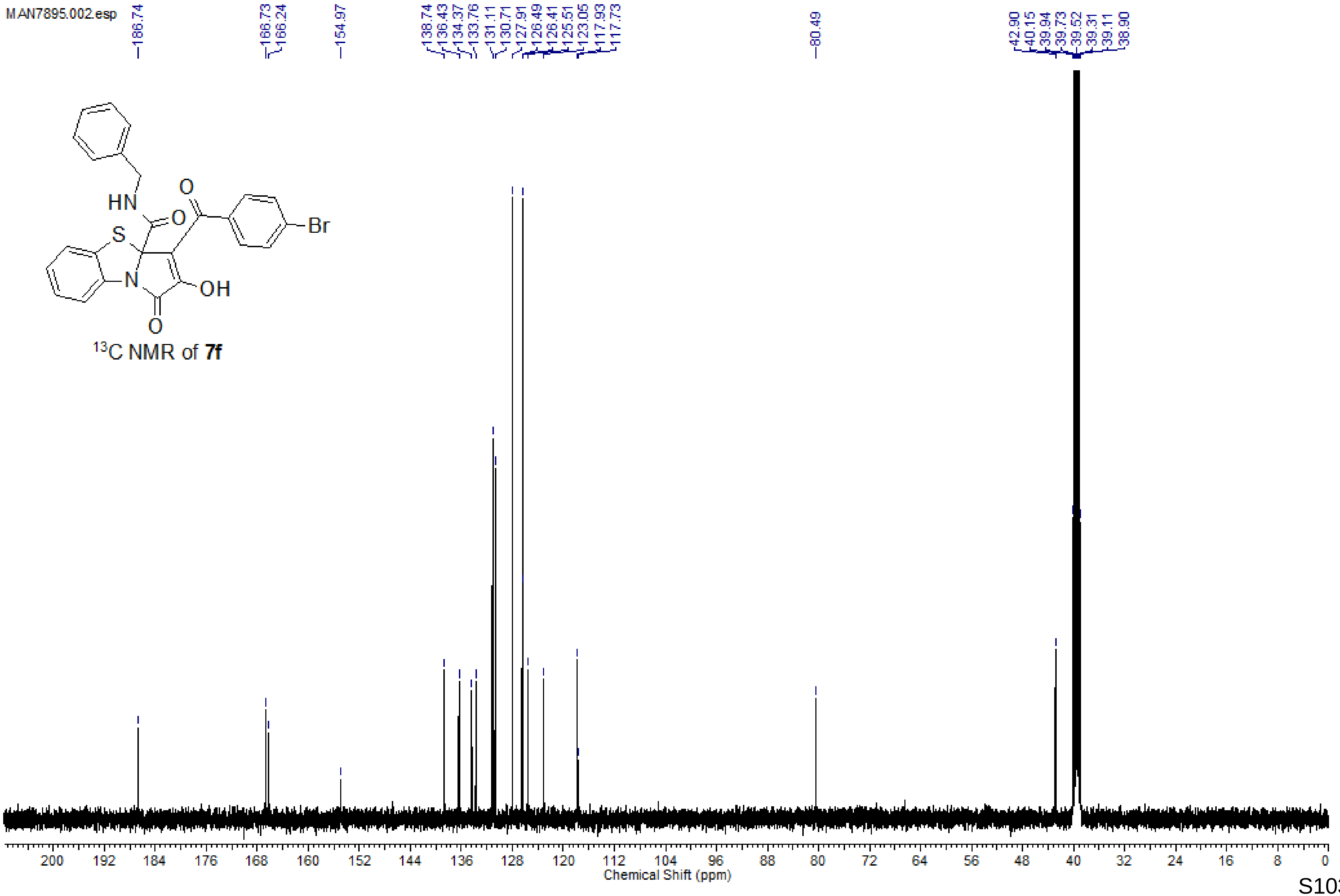

MAN7895.002.esp
186.74
166.73
166.24
154.97
138.74
136.43
134.37
133.76
131.11
130.71
127.91
126.49
126.41
125.51
123.05
117.93
117.73
80.49
42.90
40.15
39.94
39.73
39.52
39.31
39.11
38.90
HN
O
S
N
O
Br
OH
O
200 192 184 176 168 160 152 144 136 128 120 112 104 96 88 80 72 64 56 48 40 32 24 16 8 0
Chemical Shift (ppm)

^{13}C NMR of **7f**

MAN7895.003.esp

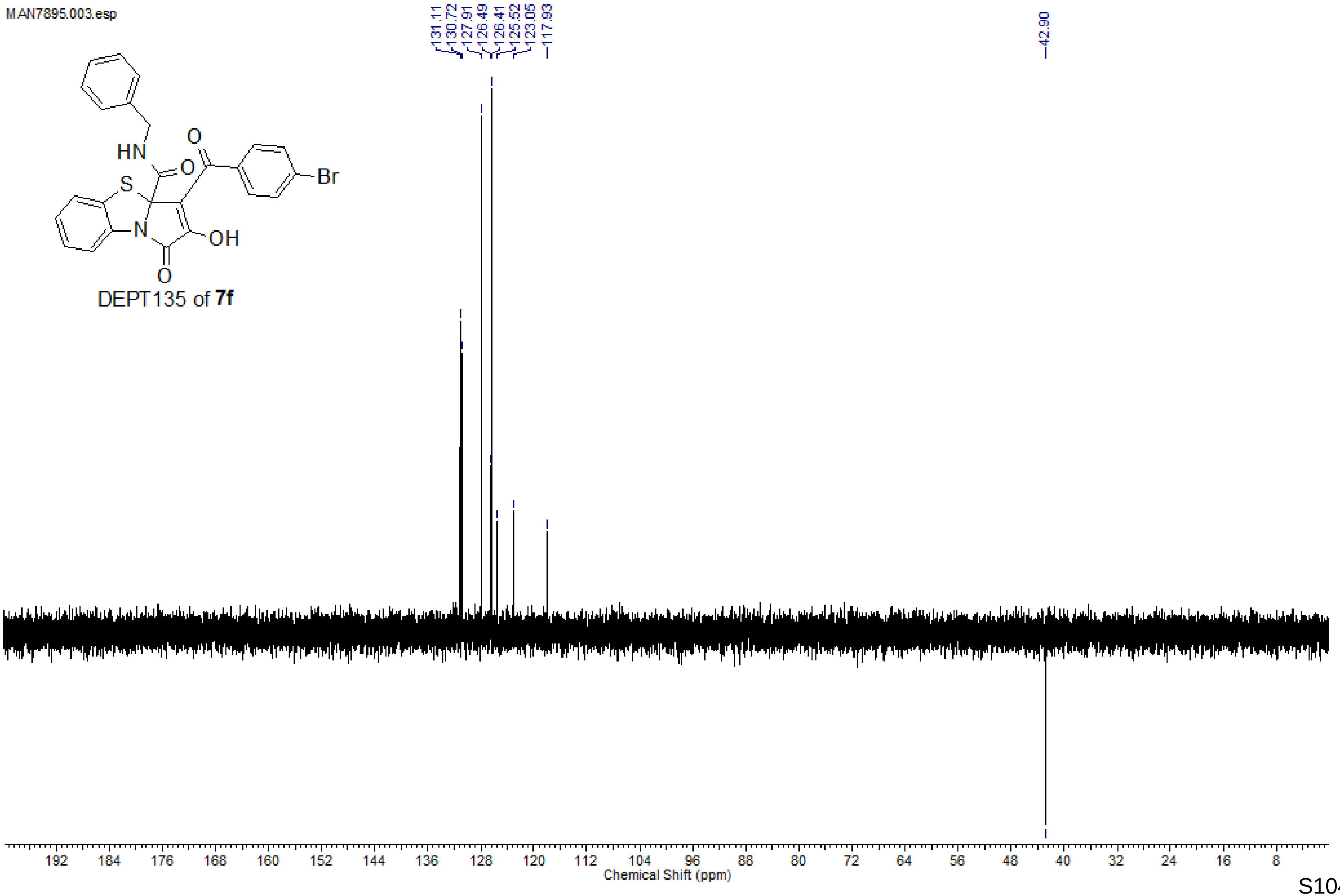

DEPT135 of **7f**

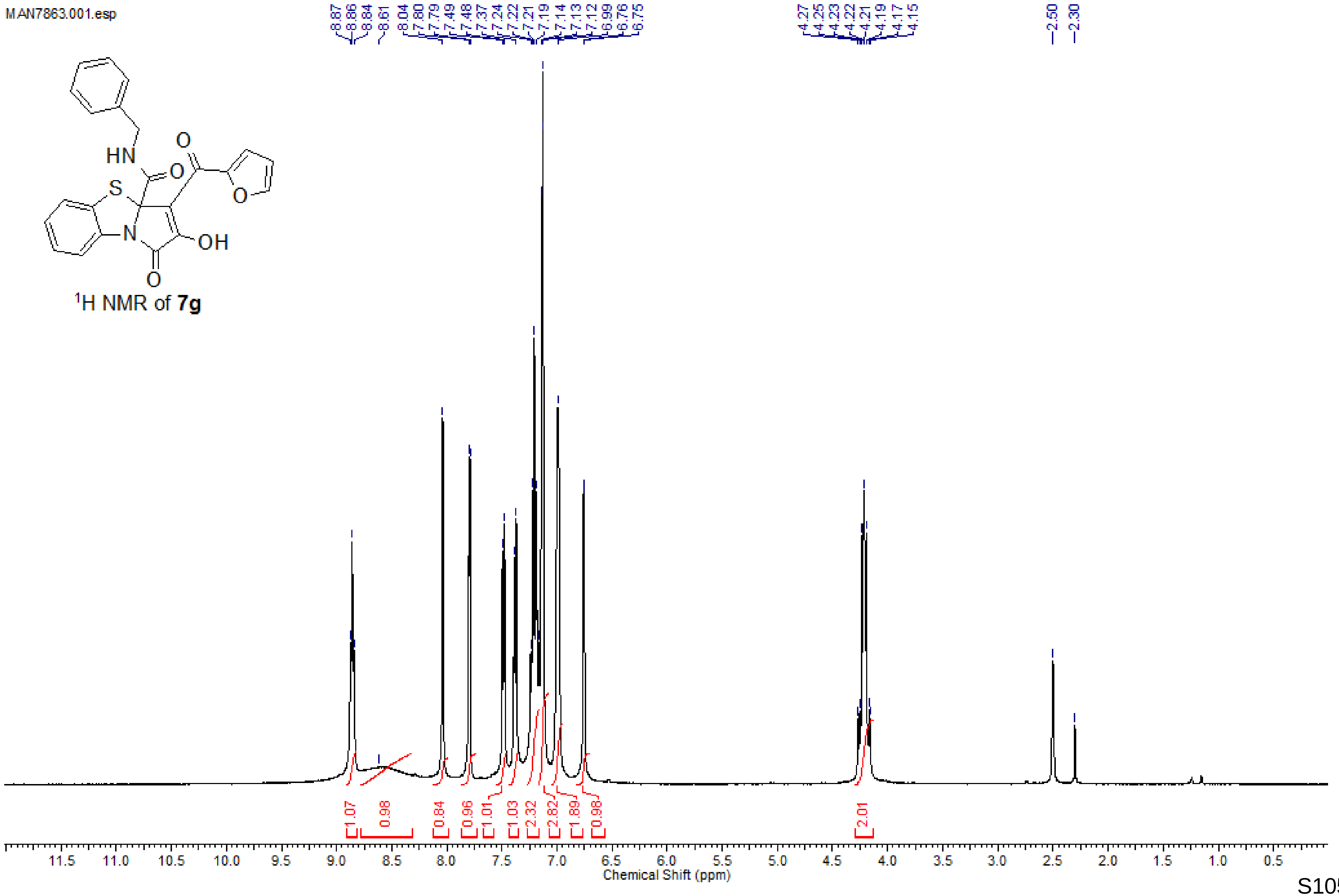
MAN7863.001.esp
HN
O
O
S
O
N
OH
O
8.87
8.86
8.84
8.61
8.04
7.80
7.79
7.49
7.48
7.37
7.24
7.22
7.21
7.19
7.14
7.13
7.12
6.99
6.76
6.75
4.27
4.25
4.23
4.22
4.21
4.19
4.17
4.15
2.50
2.30
1.07
0.98
0.84
0.96
1.01
1.03
2.32
2.82
1.89
0.98
2.01
Chemical Shift (ppm)

^{1}H NMR of **7g**

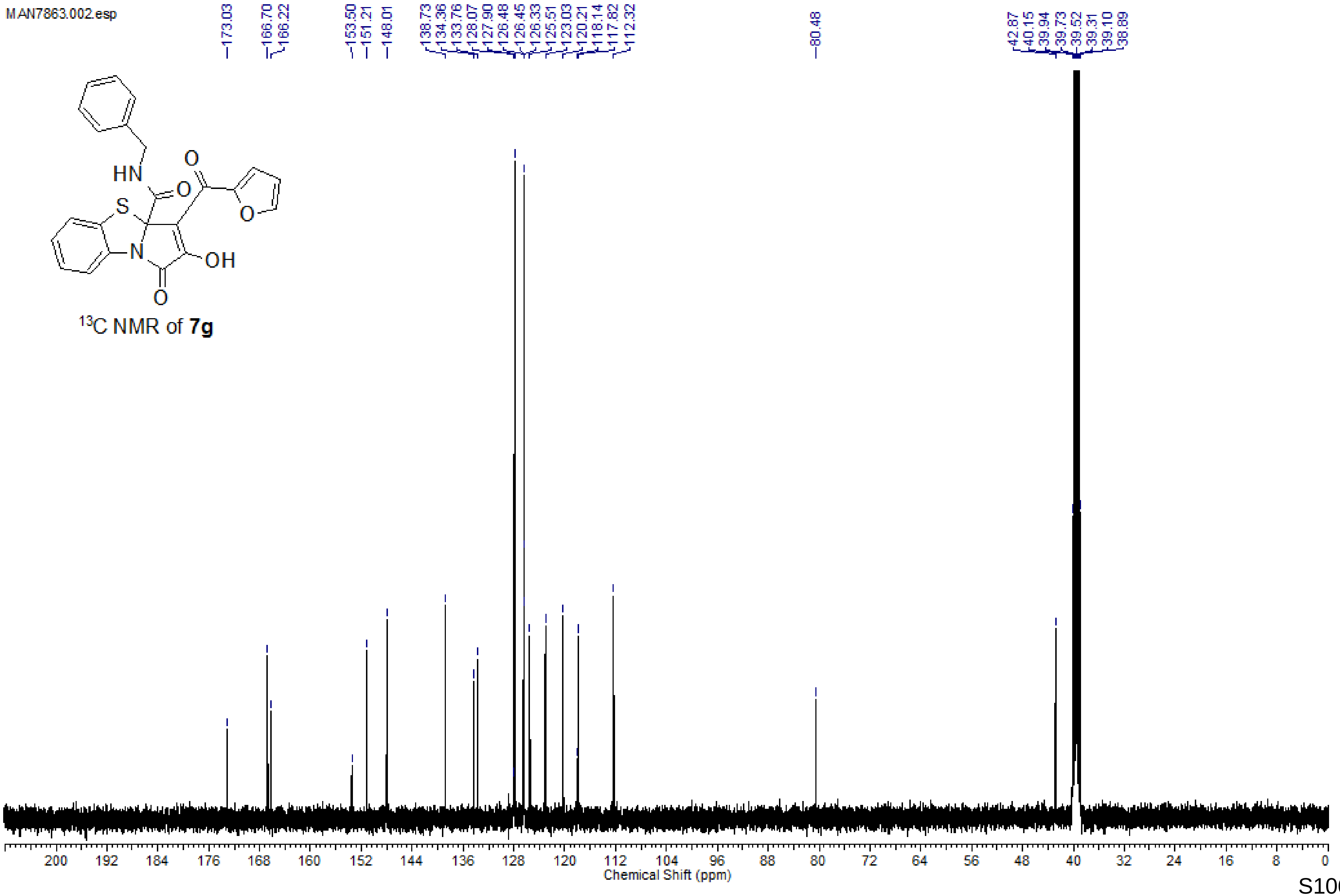

MAN7863.002.esp
173.03
166.70
166.22
153.50
151.21
148.01
138.73
134.36
133.76
128.07
127.90
126.48
126.45
126.33
125.51
123.03
120.21
118.14
117.82
112.32
80.48
42.87
40.15
39.94
39.73
39.52
39.31
39.10
38.89
HN
O
O
S
O
N
OH
O
200 192 184 176 168 160 152 144 136 128 120 112 104 96 88 80 72 64 56 48 40 32 24 16 8 0
Chemical Shift (ppm)

^{13}C NMR of **7g**

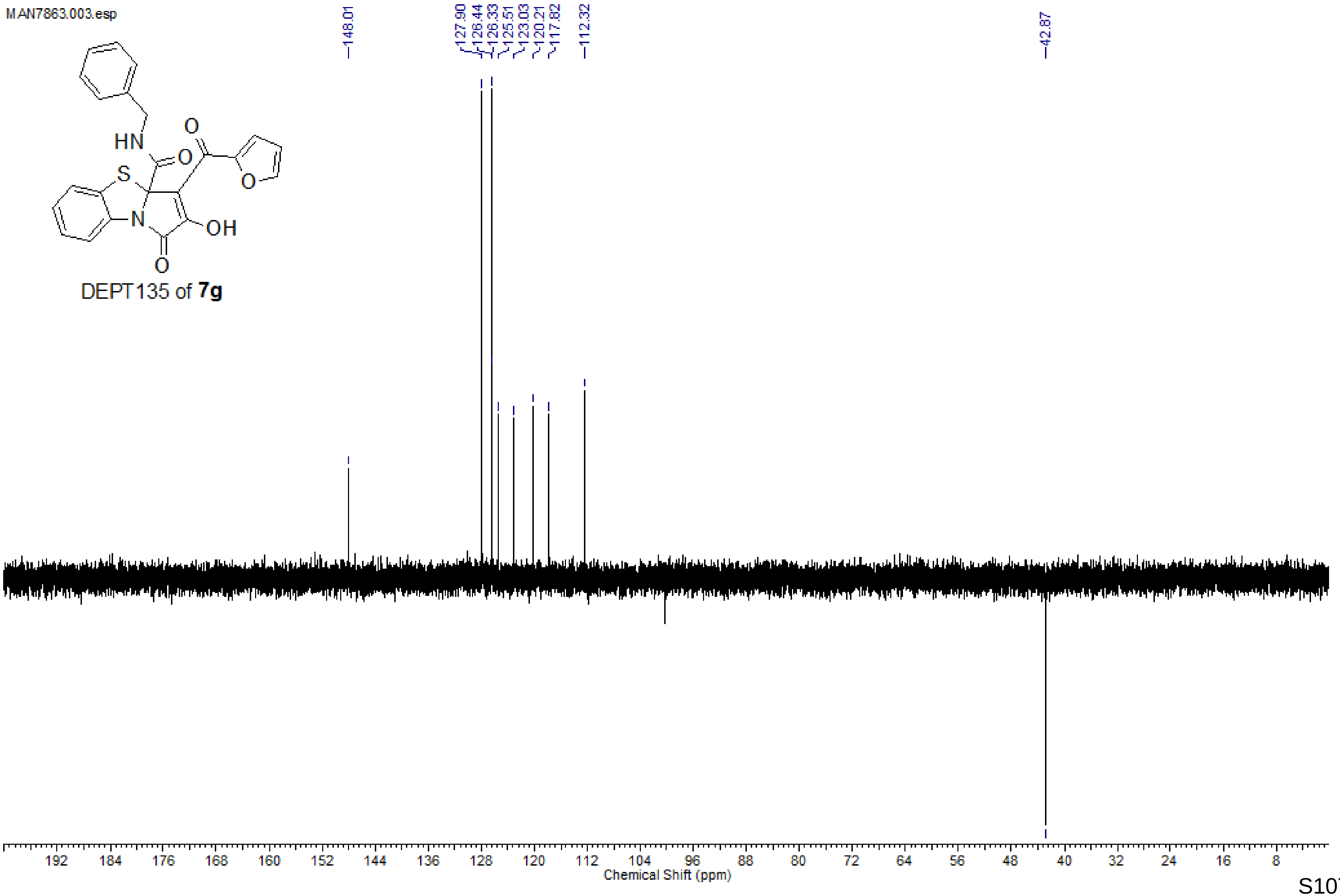

DEPT135 of **7g**

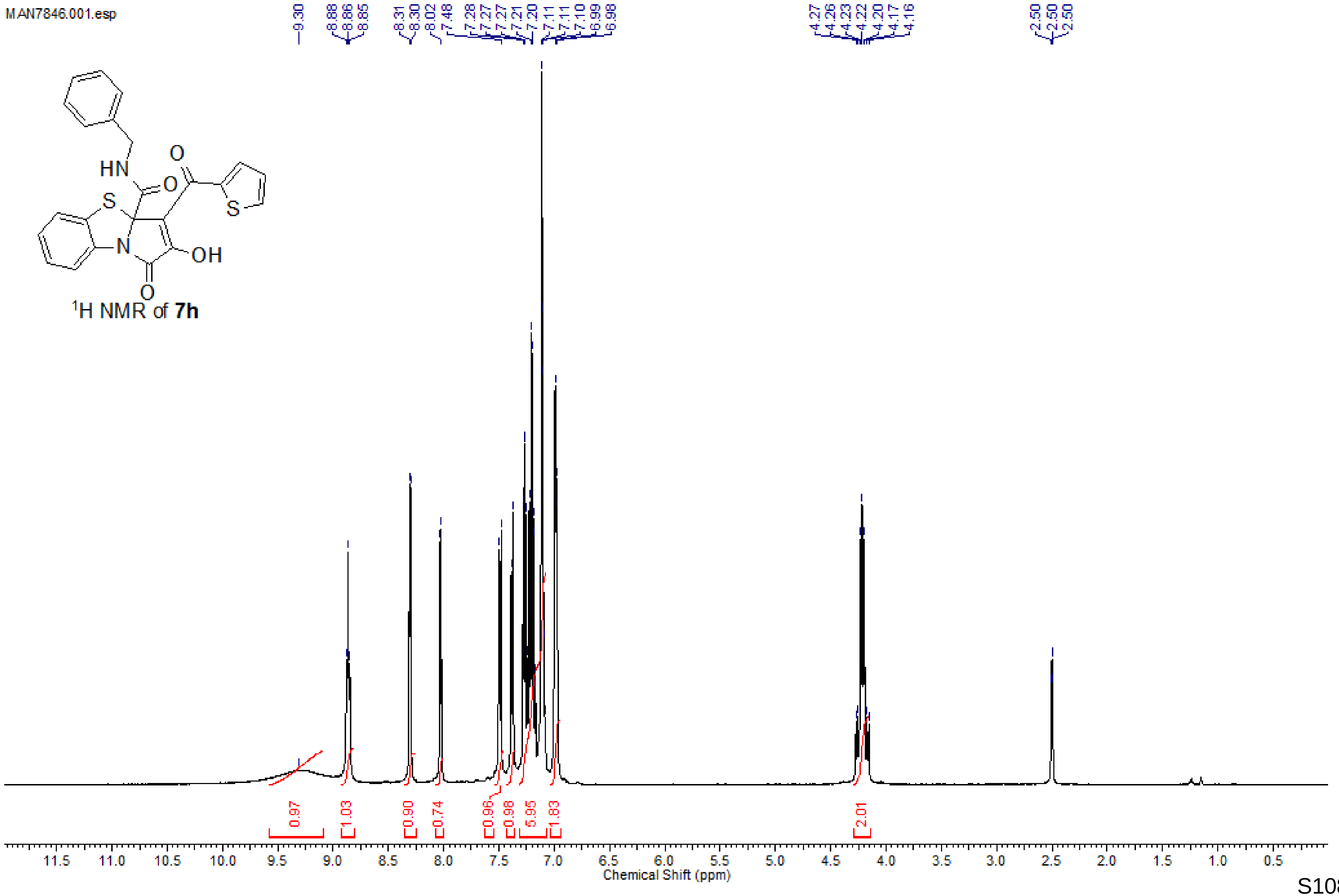
MAN7846.001.esp
9.30
8.88
8.86
8.85
8.31
8.30
8.02
7.48
7.28
7.27
7.27
7.21
7.20
7.11
7.11
7.10
6.99
6.98
4.27
4.26
4.23
4.22
4.20
4.17
4.16
2.50
2.50
2.50
O
HN
O
S
S
N
OH
O
0.97
1.03
0.90
0.74
0.96
0.98
5.95
1.83
2.01
11.5 11.0 10.5 10.0 9.5 9.0 8.5 8.0 7.5 7.0 6.5 6.0 5.5 5.0 4.5 4.0 3.5 3.0 2.5 2.0 1.5 1.0 0.5
Chemical Shift (ppm)

^{1}H NMR of **7h**

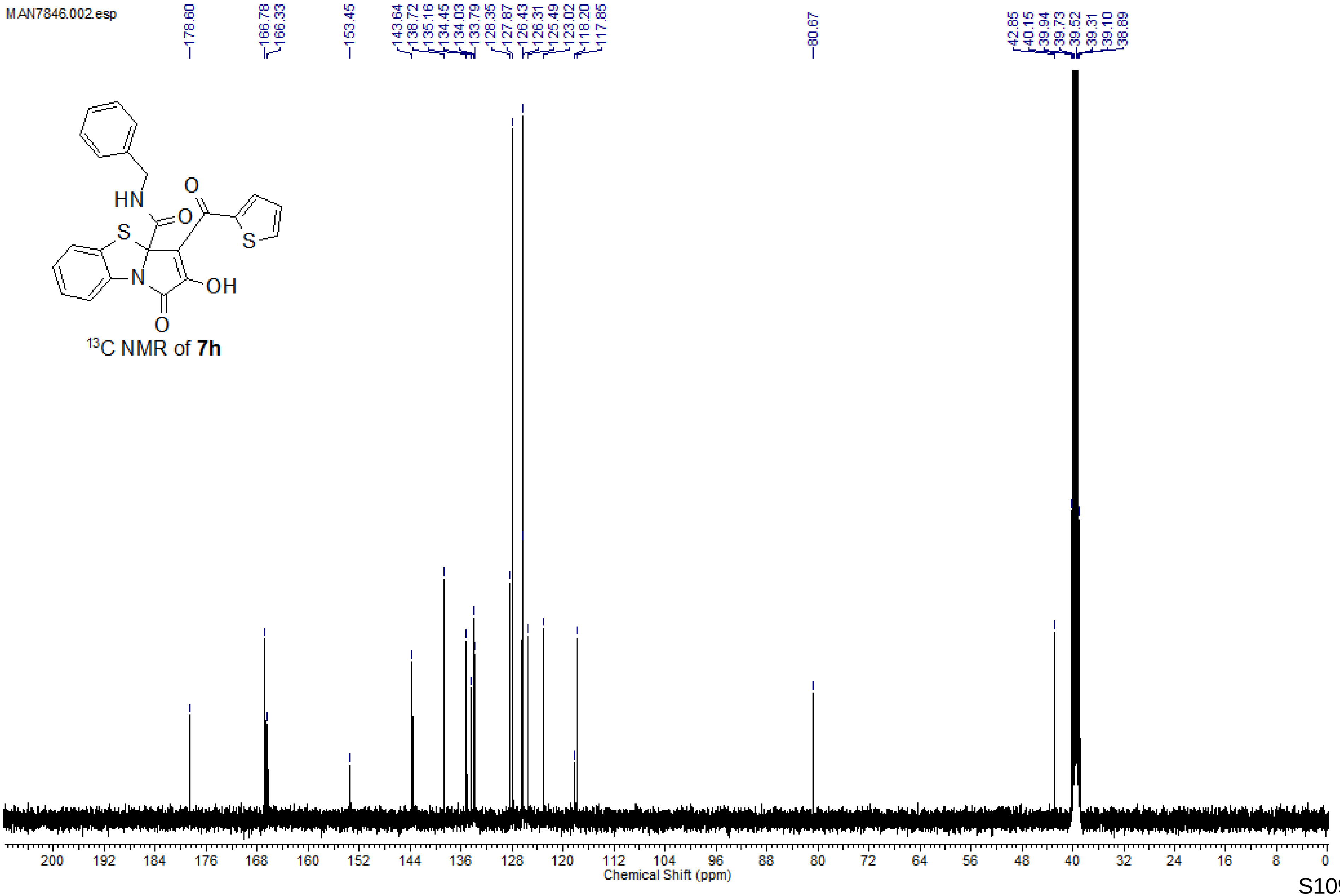

MAN7846.002.esp
178.60
166.78
166.33
153.45
143.64
138.72
135.16
134.45
134.03
133.79
128.35
127.87
126.43
126.31
125.49
123.02
118.20
117.85
80.67
42.85
40.15
39.94
39.73
39.52
39.31
39.10
38.89
Chemical Shift (ppm)

^{13}C NMR of **7h**

MAN7846.003.esp

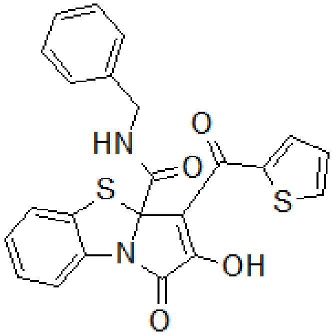

DEPT135 of **7h**

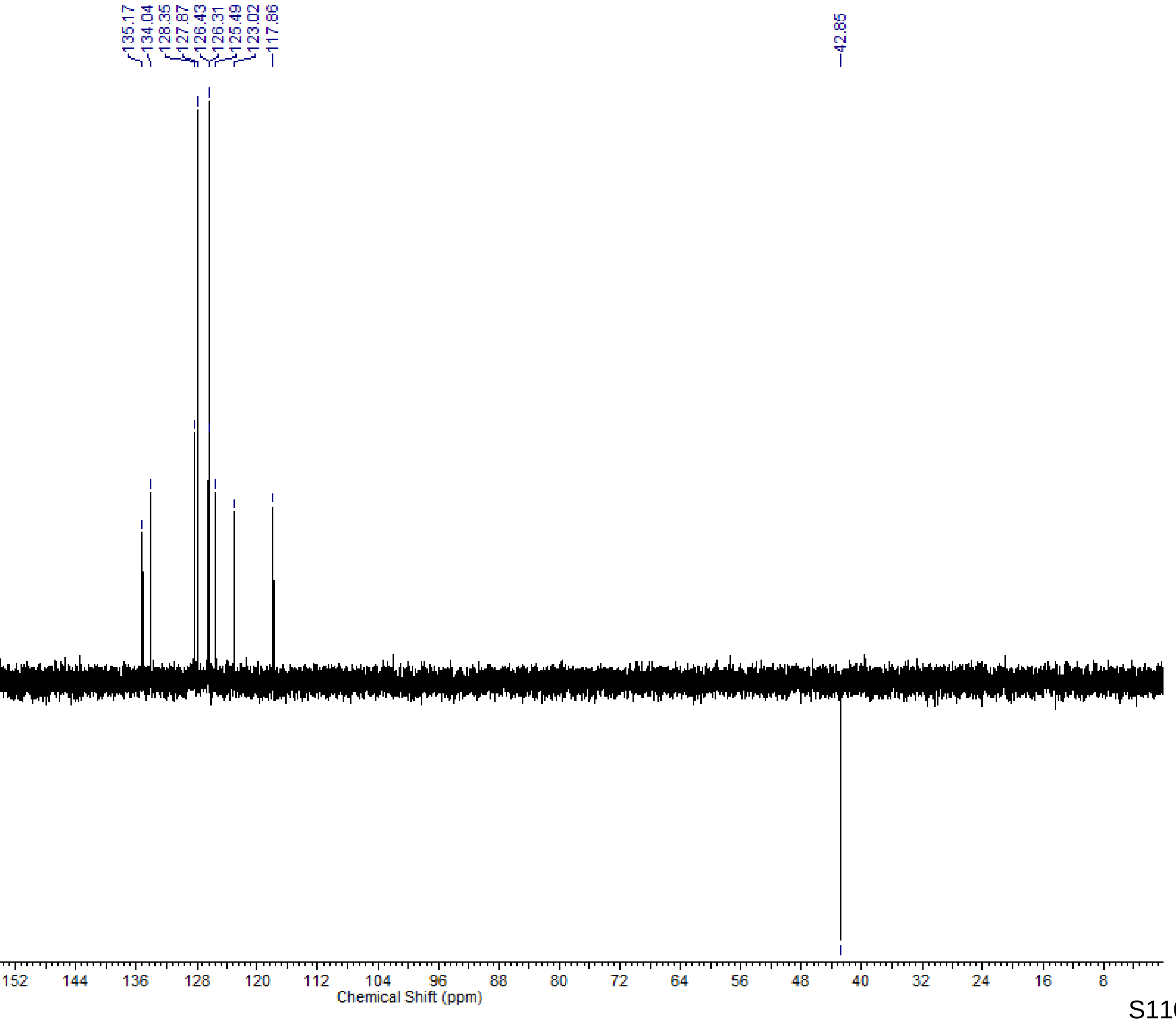

1-(Morpholin-4-yl)-3-(2-oxo-2*H*-1,4-benzothiazin-3(4*H*)-ylidene)-4-phenylbutane-1,2,4-trione (10a)

A mixture of compound **1a** (150 mg, 0.45 mmol), morpholine (43 µL, 0.49 mmol), and anhydrous toluene (3 mL) is stirred in a closed microreaction V-vial at 113 °C for 1.5 h. Then, the reaction mixture is cooled to room temperature, and hexane (5 mL) is added to it. The formed precipitate of compound **10a** is filtered off.

Orange solid; yield (127 mg, 67%); mp 151–153 °C (hexane, toluene).

^{1}H NMR (400 MHz, DMSO-d_6): δ = 14.15 (s, 1H), 7.83 (m, 2H), 7.62 (m, 1H), 7.51 (m, 3H), 7.43 (m, 2H), 7.34 (m, 1H), 3.66 (m, 2H), 3.54 (m, 2H), 3.44 (m, 2H), 3.35 (m, 2H) ppm.

^{13}C NMR (100 MHz, DMSO-d_6): δ = 193.4, 189.1, 181.2, 165.0, 144.2, 139.8, 132.5, 129.8, 129.3, 128.7 (2C), 128.7 (2C), 128.2, 125.8, 122.9, 120.8, 115.0, 66.3, 66.2, 46.5, 41.5 ppm.

IR (mineral oil): 3462, 1728, 1651, 1640 cm^{-1}.

EA: Found: C, 62.83; H, 4.42; N, 6.71. Calc. for $C_{22}H_{18}N_2O_5S$: C, 62.55; H, 4.29; N, 6.63.

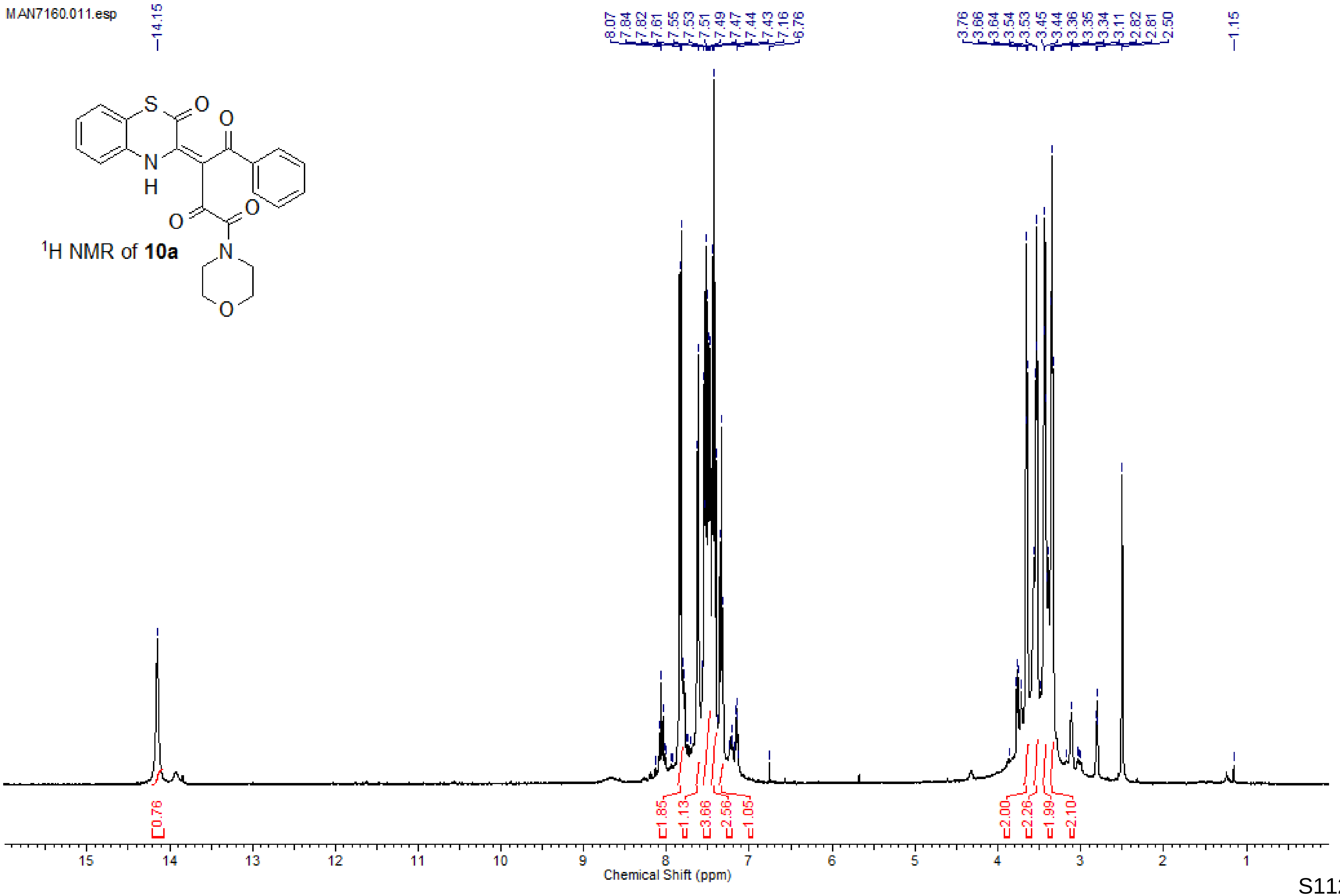

MAN7160.011.esp
14.15
8.07 7.84 7.82 7.61 7.55 7.53 7.51 7.49 7.47 7.44 7.43 7.16 6.76
3.76 3.66 3.64 3.54 3.53 3.45 3.44 3.36 3.35 3.34 3.11 2.82 2.81 2.50
1.15
S
O
N
H
^{1}H NMR of **10a**
0.76
1.85 1.13 3.66 2.56 1.05
2.00 2.26 1.99 2.10
15 14 13 12 11 10 9 8 7 6 5 4 3 2 1
Chemical Shift (ppm)

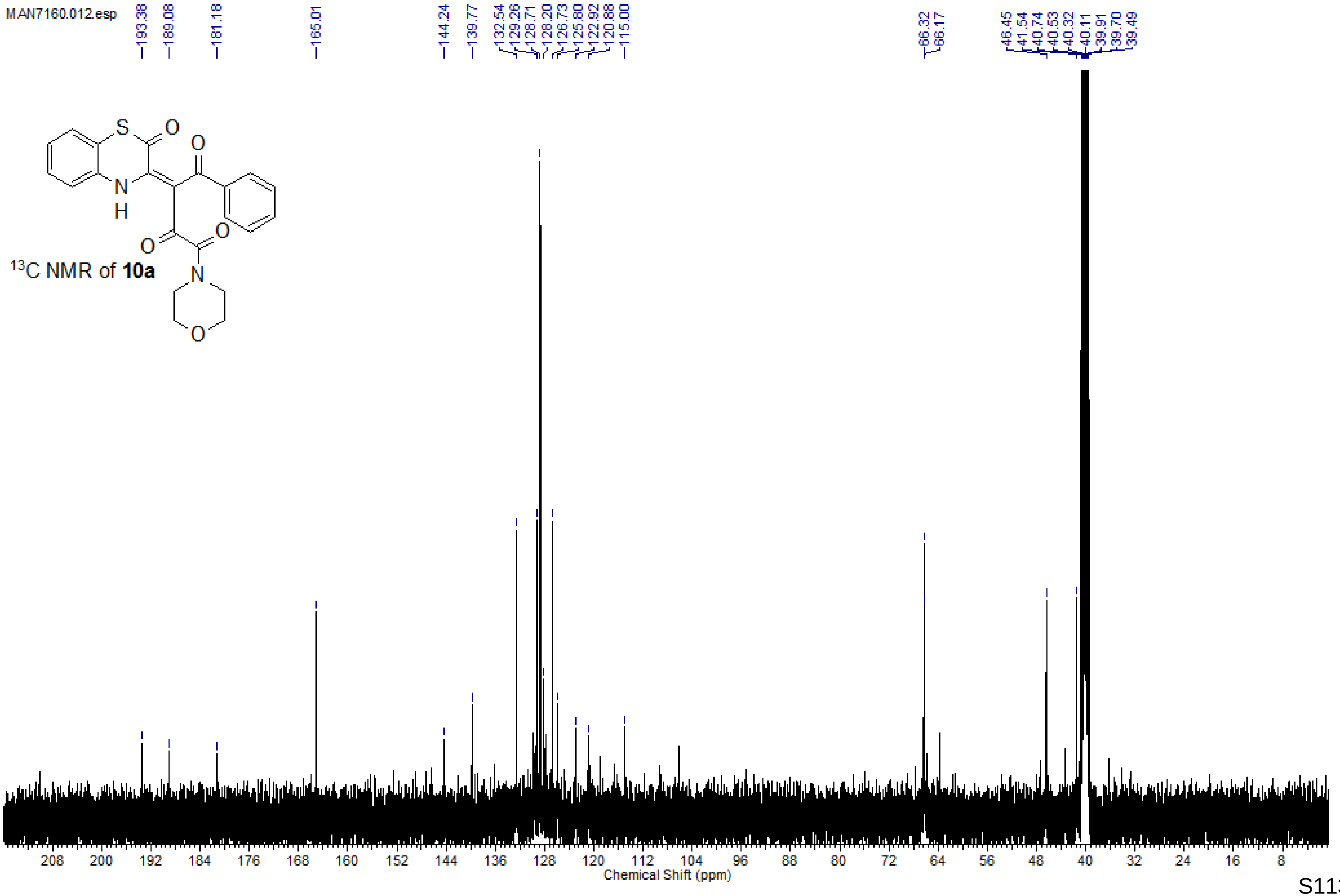

MAN7160.012.esp
193.38
189.08
181.18
165.01
144.24
139.77
132.54
129.26
128.71
128.20
126.73
125.80
122.92
120.88
115.00
66.32
66.17
46.45
41.54
40.74
40.53
40.32
40.11
39.91
39.70
39.49
^{13}C NMR of **10a**
208
200
192
184
176
168
160
152
144
136
128
120
112
104
96
88
80
72
64
56
48
40
32
24
16
8
Chemical Shift (ppm)

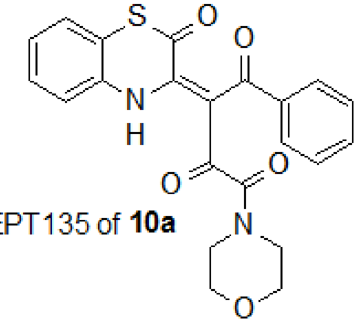

DEPT135 of **10a**

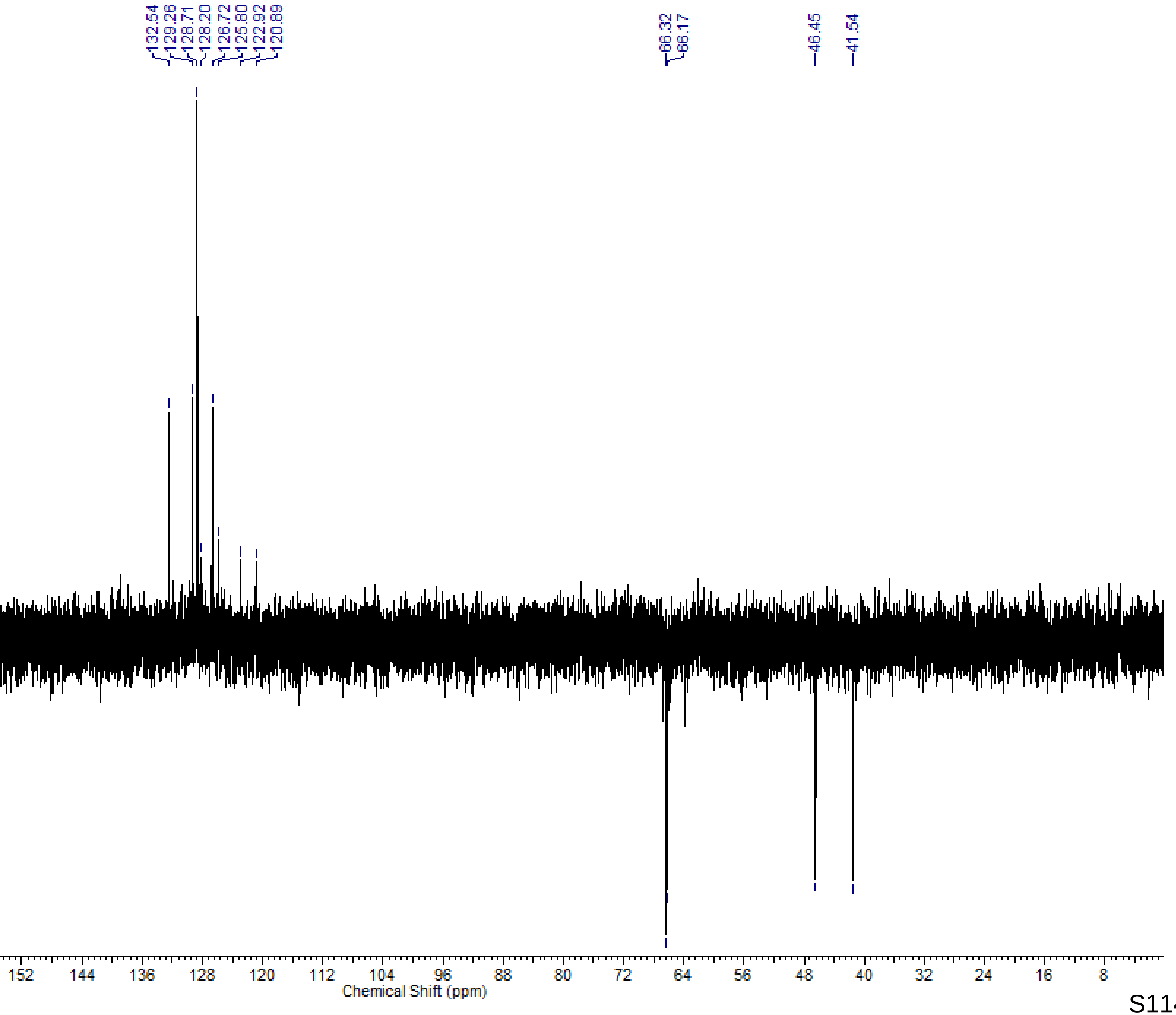

_N_-Aryl-3-aroyl-2-hydroxy-1-oxopyrrolo[2,1-_b_][1,3]benzothiazole-3a(1_H_)-carboxamides 12. General procedure.

A mixture of APBTT **1** (0.45 mmol), arylamine **11** (0.49 mmol for **11a,b** or 0.45 mmol for **11c,d**), and anhydrous toluene (3 mL) is stirred in a closed microreaction V-vial at 113 °C for 3 or 8 h (3 h for compounds **12ad,bd**). Then, the resulting solution is cooled to room temperature. In 24 h, the formed precipitate of compound **12** is filtered off and recrystallized from toluene (compound **12ca** is recrystallized from acetonitrile).

3-Benzoyl-2-hydroxy-1-oxo-_N_-phenylpyrrolo[2,1-_b_][1,3]benzothiazole-3a(1_H_)-carboxamide (12aa)
Yellow solid; yield (77 mg, 40%); mp 196–198 °C (toluene).
^{1}H NMR (400 MHz, DMSO-d_6): δ = 10.18 (s, 1H), 9.90 (br.s, 1H), 7.82 (m, 2H), 7.59 (m, 1H), 7.53 (m, 1H), 7.49 (m, 4H), 7.40 (m, 1H), 7.27 (m, 2H), 7.21 (m, 2H), 7.05 (m, 1H) ppm.
^{13}C NMR (100 MHz, DMSO-d_6): δ = 188.0, 166.8, 165.3, 155.7, 138.0, 137.5, 134.4, 133.9, 132.5, 128.8 (2C), 128.5 (2C), 128.0 (2C), 126.6, 125.5, 124.1, 123.0, 120.3 (2C), 118.2, 117.0, 81.7 ppm.
IR (mineral oil): 3200, 3080, 1727, 1678 cm^{-1}.
EA: Found: C, 67.41; H, 3.81 N, 6.60. Calc. for $C_{24}H_{16}N_2O_4S$: C, 67.28; H, 3.76; N, 6.54.

2-Hydroxy-3-(4-methoxybenzoyl)-1-oxo-_N_-phenylpyrrolo[2,1-_b_][1,3]benzothiazole-3a(1_H_)-carboxamide (12ca)
Yellow solid; yield (66 mg, 34%); mp 215–217 °C (acetonitrile).
^{1}H NMR (400 MHz, DMSO-d_6): δ = 10.15 (s, 1H), 7.87 (m, 2H), 7.53 (m, 1H), 7.46 (m, 2H), 7.39 (m, 1H), 7.27–7.17 (m, 4H), 7.04 (m, 3H), 3.84 (s, 3H) ppm.
^{13}C NMR (100 MHz, DMSO-d_6): δ = 186.8, 167.3, 165.2, 163.4, 152.9, 138.0, 135.2, 133.8, 131.6 (2C), 129.8, 128.6 (2C), 126.8, 125.6, 124.3, 123.1, 120.5 (2C), 118.5, 118.2, 113.7 (2C), 82.2, 55.6 ppm.
IR (mineral oil): 3342, 3215, 1720, 1691, 1659 cm^{-1}.
EA: Found: C, 65.59; H, 4.00 N, 6.15. Calc. for $C_{25}H_{18}N_2O_5S$: C, 65.49; H, 3.96; N, 6.11.

3-Benzoyl-2-hydroxy-_N_-mesityl-1-oxopyrrolo[2,1-_b_][1,3]benzothiazole-3a(1_H_)-carboxamide (12ab)
Yellow solid; yield (83 mg, 39%); mp 227–229 °C (toluene).
^{1}H NMR (400 MHz, DMSO-d_6): δ = 9.64 (s, 1H), 7.85 (m, 2H), 7.63 (m, 1H), 7.52 (m, 3H), 7.42 (m, 1H), 7.26–7.19 (m, 2H), 6.78 (m, 2H), 5.39 (br.s, 1H), 2.16 (s, 3H), 1.79 (s, 6H) ppm.
^{13}C NMR (100 MHz, DMSO-d_6): δ = 188.1, 166.2, 165.0, 153.9, 137.2, 135.7, 134.9, 134.8 (2C), 133.7, 132.7, 131.7, 128.8 (2C), 128.1 (2C), 128.1 (2C), 126.6, 125.5, 123.1, 118.4, 118.1, 81.3, 20.3, 17.2 (2C) ppm.
IR (mineral oil): 3325, 3181, 1731, 1666, 1645 cm^{-1}.

EA: Found: C, 69.04; H, 4.88; N, 6.00. Calc. for $C_{27}H_{22}N_2O_4S$: C, 68.92; H, 4.71; N, 5.95.

2-Hydroxy-*N*-mesityl-3-(4-methylbenzoyl)-1-oxopyrrolo[2,1-*b*][1,3]benzothiazole-3a(1*H*)-carboxamide (12bb)
Yellow solid; yield (70 mg, 32%); mp 201–203 °C (toluene).
^{1}H NMR (400 MHz, DMSO-d_6): δ = 9.61 (s, 1H), 7.77 (m, 2H), 7.53 (m, 1H), 7.42 (m, 1H), 7.33 (m, 2H), 7.26–7.19 (m, 2H), 6.77 (m, 2H), 5.92 (br.s, 1H), 2.40 (s, 3H), 2.16 (s, 3H), 1.78 (s, 6H) ppm.
^{13}C NMR (100 MHz, DMSO-d_6): δ = 187.7, 166.3, 165.0, 153.2, 143.2, 135.7, 135.0, 134.8 (2C), 134.6, 133.7, 131.7, 129.1 (2C), 128.6 (2C), 128.1 (2C), 126.6, 125.5, 123.1, 118.6, 118.1, 81.3, 21.1, 20.3, 17.2 (2C) ppm.
IR (mineral oil): 3331, 3181, 1728, 1665, 1645 cm^{-1}.
EA: Found: C, 69.51; H, 5.07; N, 5.83. Calc. for $C_{28}H_{24}N_2O_4S$: C, 69.40; H, 4.99; N, 5.78.

2-Hydroxy-*N*-mesityl-3-(4-methoxybenzoyl)-1-oxopyrrolo[2,1-*b*][1,3]benzothiazole-3a(1*H*)-carboxamide (12cb)
Yellow solid; yield (81 mg, 36%); mp 203–205 °C (toluene).
^{1}H NMR (400 MHz, DMSO-d_6): δ = 9.59 (s, 1H), 7.88 (m, 2H), 7.52 (m, 1H), 7.41 (m, 1H), 7.22 (m, 2H), 7.05 (m, 2H), 6.76 (m, 2H), 3.87 (s, 3H), 2.16 (s, 3H), 1.76 (s, 6H) ppm.
^{13}C NMR (100 MHz, DMSO-d_6): δ = 186.7, 166.6, 165.0, 163.3, 151.8, 135.8, 135.5, 134.9 (2C), 133.7, 131.8, 131.6 (2C), 129.7, 128.2 (2C), 126.7, 125.6, 123.2, 119.1, 118.3, 113.6 (2C), 81.5, 55.6, 20.4, 17.3 (2C) ppm.
IR (mineral oil): 3323, 3169, 1725, 1665, 1641 cm^{-1}.
EA: Found: C, 67.25; H, 4.93; N, 5.72. Calc. for $C_{28}H_{24}N_2O_5S$: C, 67.19; H, 4.83; N, 5.60.

3-(4-Fluorobenzoyl)-2-Hydroxy-*N*-mesityl-1-oxopyrrolo[2,1-*b*][1,3]benzothiazole-3a(1*H*)-carboxamide (12db)
Yellow solid; yield (92 mg, 42%); mp 229–231 °C (toluene).
^{1}H NMR (400 MHz, DMSO-d_6): δ = 9.63 (s, 1H), 7.95 (m, 2H), 7.52 (m, 1H), 7.42 (m, 1H), 7.35 (m, 2H), 7.26–7.19 (m, 2H), 6.78 (m, 2H), 2.16 (s, 3H), 1.79 (s, 6H) ppm.
^{13}C NMR (100 MHz, DMSO-d_6): δ = 186.5, 166.2, 165.9, 163.5, 165.1, 154.4, 135.7, 134.9, 134.8 (2C), 133.9, 133.7, 133.9, 131.8, 131.7 (2C), 128.1 (2C), 126.6, 125.5, 123.1, 118.1, 118.0, 115.2, 115.0, 81.3, 20.3, 17.2 (2C) ppm.
IR (mineral oil): 3332, 3195, 1728, 1666, 1647 cm^{-1}.
EA: Found: C, 66.47; H, 4.39; N, 5.78. Calc. for $C_{27}H_{21}FN_2O_4S$: C, 66.38; H, 4.33; N, 5.73.

3-(4-Chlorobenzoyl)-2-Hydroxy-*N*-mesityl-1-oxopyrrolo[2,1-*b*][1,3]benzothiazole-3a(1*H*)-carboxamide (12eb)
Yellow solid; yield (99 mg, 44%); mp 184–186 °C (toluene).

^{1}H NMR (400 MHz, DMSO-d_6): δ = 9.65 (s, 1H), 7.85 (m, 2H), 7.58 (m, 2H), 7.52 (m, 1H), 7.41 (m, 1H), 7.22 (m, 2H), 6.78 (m, 2H), 5.90 (br.s, 1H), 2.16 (s, 3H), 1.80 (s, 6H) ppm.
^{13}C NMR (100 MHz, DMSO-d_6): δ = 186.5, 166.2, 165.3, 156.0, 137.2, 136.3, 135.7, 134.8 (2C), 134.6, 133.8, 131.7, 130.6 (2C), 128.1 (2C), 128.1 (2C), 126.6, 125.5, 123.0, 117.9, 81.2, 20.3, 17.2 (2C) ppm.
IR (mineral oil): 3255, 3065, 1744, 1683, 1646 cm^{-1}.
EA: Found: C, 64.35; H, 4.21; N, 5.60. Calc. for $C_{27}H_{21}ClN_2O_4S$: C, 64.22; H, 4.19; N, 5.55.

3-(4-Bromobenzoyl)-2-Hydroxy-*N*-mesityl-1-oxopyrrolo[2,1-*b*][1,3]benzothiazole-3a(1*H*)-carboxamide (12fb)
Yellow solid; yield (101 mg, 41%); mp 156–158 °C (toluene).
^{1}H NMR (400 MHz, DMSO-d_6): δ = 9.67 (s, 1H), 7.76 (m, 2H), 7.70 (m, 2H), 7.51 (m, 1H), 7.40 (m, 1H), 7.24–7.17 (m, 2H), 6.78 (m, 2H), 5.80 (br.s, 1H), 2.17 (s, 3H), 1.82 (s, 6H) ppm.
^{13}C NMR (100 MHz, DMSO-d_6): δ = 187.0, 166.3, 165.2, 155.6, 136.6, 135.8, 134.9 (2C), 133.8, 131.8, 131.2 (2C), 130.9 (2C), 128.2 (2C), 126.8, 126.6, 125.6, 123.2, 118.1, 117.5, 81.3, 20.4, 17.3 (2C) ppm.
IR (mineral oil): 3251, 3083, 1744, 1683, 1645 cm^{-1}.
EA: Found: C, 59.15; H, 3.70; N, 5.14. Calc. for $C_{27}H_{21}BrN_2O_4S$: C, 59.02; H, 3.85; N, 5.10.

3-(4-Furan-2-carbonyl)-2-Hydroxy-*N*-mesityl-1-oxopyrrolo[2,1-*b*][1,3]benzothiazole-3a(1*H*)-carboxamide (12gb)
Greenish yellow solid; yield (66 mg, 32%); mp 184–186 °C (toluene).
^{1}H NMR (400 MHz, DMSO-d_6): δ = 9.66 (s, 1H), 8.01 (m, 1H), 7.95 (m, 1H), 7.50 (m, 1H), 7.39 (m, 1H), 7.24–7.16 (m, 2H), 6.97 (br.s, 1H), 6.77 (m, 2H), 6.75 (m, 1H), 2.16 (s, 3H), 1.80 (s, 6H) ppm.
^{13}C NMR (100 MHz, DMSO-d_6): δ = 173.1, 166.3, 165.3, 154.2, 151.3, 148.1, 135.7, 134.9 (2C), 134.7, 133.9, 131.8, 128.2 (2C), 126.6, 125.6, 123.2, 120.3, 118.0, 117.8, 112.4, 81.2, 20.4, 17.2 (2C) ppm.
IR (mineral oil): 3326, 3148, 1744, 1731, 1661 cm^{-1}.
EA: Found: C, 65.33; H, 4.47; N, 6.16. Calc. for $C_{25}H_{20}N_2O_5S$: C, 65.21; H, 4.38; N, 6.08.

2-Hydroxy-*N*-mesityl-1-oxo-3-(thiophene-2-carbonyl)pyrrolo[2,1-*b*][1,3]benzothiazole-3a(1*H*)-carboxamide (12hb)
Greenish yellow solid; yield (64 mg, 30%); mp 230–232 °C (toluene).
^{1}H NMR (400 MHz, DMSO-d_6): δ = 9.68 (s, 1H), 8.54 (m, 1H), 7.96 (m, 1H), 7.51 (m, 1H), 7.38 (m, 1H), 7.25 (m, 1H), 7.21–7.15 (m, 2H), 6.77 (m, 2H), 5.47 (br.s, 1H), 2.16 (s, 3H), 1.81 (s, 6H) ppm.
^{13}C NMR (100 MHz, DMSO-d_6): δ = 178.6, 166.4, 165.4, 154.5, 143.8, 135.7, 135.2, 134.8 (2C), 134.8, 134.0, 133.9, 131.8, 128.5, 128.2 (2C), 126.6, 125.6, 123.2, 118.0, 117.6, 81.3, 20.4, 17.2 (2C) ppm.
IR (mineral oil): 3331, 3168, 1740, 1653 cm^{-1}.

EA: Found: C, 63.14; H, 4.29; N, 5.97. Calc. for $C_{25}H_{20}N_2O_4S_2$: C, 63.01; H, 4.23; N, 5.88.

3-Benzoyl-2-hydroxy-*N*-(4-nitrophenyl)-1-oxopyrrolo[2,1-*b*][1,3]benzothiazole-3a(1*H*)-carboxamide (12ac)
Yellow solid; yield (96 mg, 45%); mp 220–222 °C (toluene).
^{1}H NMR (400 MHz, DMSO-d_6): δ = 10.87 (s, 1H), 8.17 (m, 2H), 7.81 (m, 4H), 7.57 (m, 2H), 7.47 (m, 2H), 7.40 (m, 1H), 7.26–7.12 (m, 3H), 6.67 (br.s, 1H) ppm.
^{13}C NMR (100 MHz, DMSO-d_6): δ = 187.7, 166.8, 166.3, 157.5, 144.4, 142.8, 137.6, 134.0, 132.3, 128.8 (2C), 128.1, 127.9 (2C), 126.7, 125.6, 124.6 (2C), 123.0, 120.0 (2C), 118.2, 115.5, 81.8 ppm.
IR (mineral oil): 3302, 3081, 1737, 1715, 1687 cm^{-1}.
EA: Found: C, 61.90; H, 3.42; N, 8.64. Calc. for $6C_{24}H_{15}N_3O_6S \cdot C_7H_8$: C, 61.84; H, 3.37; N, 8.60.

2-Hydroxy-3-(4-methylbenzoyl)-*N*-(4-nitrophenyl)-1-oxopyrrolo[2,1-*b*][1,3]benzothiazole-3a(1*H*)-carboxamide (12bc)
Yellow solid; yield (83 mg, 38%); mp 210–212 °C (toluene).
^{1}H NMR (400 MHz, DMSO-d_6): δ = 10.79 (s, 1H), 8.17 (m, 2H), 7.77 (m, 4H), 7.53 (m, 1H), 7.39 (m,1H), 7.28 (m, 2H), 7.21 (m, 2H), 2.37 (s, 3H) ppm.
^{13}C NMR (100 MHz, DMSO-d_6): δ = 187.5, 167.0, 166.2, 156.1, 144.3, 143.0, 142.8, 134.8, 134.3 133.9, 129.0 (2C), 128.6 (2C), 126.7, 125.5, 124.6 (2C), 123.0, 120.0 (2C), 118.3, 116.2, 82.0, 21.1 ppm.
IR (mineral oil): 3221, 3081, 1711, 1694, 1663 cm^{-1}.
EA: Found: C, 61.78; H, 3.63; N, 8.71. Calc. for $C_{25}H_{17}N_3O_6S$: C, 61.60; H, 3.52; N, 8.62.

2-Hydroxy-3-(4-methoxylbenzoyl)-*N*-(4-nitrophenyl)-1-oxopyrrolo[2,1-*b*][1,3]benzothiazole-3a(1*H*)-carboxamide (12cc)
Yellow solid; yield (75 mg, 33%); mp 224–226 °C (toluene).
^{1}H NMR (400 MHz, DMSO-d_6): δ = 10.71 (s, 1H), 8.17 (m, 2H), 7.87 (m, 2H), 7.79 (m, 2H), 7.53 (m, 1H), 7.39 (m, 1H), 7.21 (m, 2H), 7.02 (m, 2H), 3.84 (s, 3H) ppm.
^{13}C NMR (100 MHz, DMSO-d_6): δ = 186.5, 167.1, 166.1, 163.1, 154.3, 144.3, 142.9, 134.6, 133.8, 131.4 (2C), 129.8, 126.7, 125.5, 124.6 (2C), 123.0, 120.1 (2C), 118.4, 117.0, 113.5 (2C), 82.2, 55.5 ppm.
IR (mineral oil): 3306, 3145, 1732, 1715, 1697 cm^{-1}.
EA: Found: C, 59.79; H, 3.51; N, 8.42. Calc. for $C_{25}H_{17}N_3O_7S$: C, 59.64; H, 3.40; N, 8.35.

3-(4-Fluorobenzoyl)-2-hydroxy-*N*-(4-nitrophenyl)-1-oxopyrrolo[2,1-*b*][1,3]benzothiazole-3a(1*H*)-carboxamide (12dc)
Yellow solid; yield (66 mg, 30%); mp 224–226 °C (toluene).

^{1}H NMR (400 MHz, DMSO-d_6): δ = 11.03 (s, 1H), 8.17 (m, 2H), 7.92 (m, 2H), 7.76 (m, 2H), 7.54 (m, 1H), 7.38 (m, 1H), 7.27 (m, 2H), 7.25–7.16 (m, 2H) ppm.
^{13}C NMR (100 MHz, DMSO-d_6): δ = 185.7, 166.7, 166.6, 165.5, 163.0, 144.5, 142.7, 134.5, 134.2, 133.4, 131.6, 131.5, 126.5, 125.5, 124.7 (2C), 123.0, 119.7 (2C), 117.9, 114.8, 114.6, 113.6, 81.5 ppm.
IR (mineral oil): 3347, 3156, 1737, 1713, 1684 cm^{-1}.
EA: Found: C, 58.72; H, 2.92; N, 8.60. Calc. for $C_{24}H_{14}FN_3O_6S$: C, 58.66; H, 2.87; N, 8.55.

3-(4-Chlorobenzoyl)-2-hydroxy-*N*-(4-nitrophenyl)-1-oxopyrrolo[2,1-*b*][1,3]benzothiazole-3a(1*H*)-carboxamide (12ec)
Yellow solid; yield (78 mg, 34%); mp 221–223 °C (toluene).
^{1}H NMR (400 MHz, DMSO-d_6): δ = 11.00 (s, 1H), 8.59 (br.s, 1H), 8.17 (m, 2H), 7.83 (m, 2H), 7.76 (m, 2H), 7.53 (m, 3H), 7.38 (m, 1H), 7.24–7.16 (m, 2H) ppm.
^{13}C NMR (100 MHz, DMSO-d_6): δ = 186.0, 166.6, 166.5, 160.3, 144.5, 142.7, 136.8, 136.6, 134.2, 133.4, 130.6 (2C), 127.9 (2C), 126.6, 125.5, 124.7 (2C), 123.0, 119.8 (2C), 118.0, 113.7, 81.4 ppm.
IR (mineral oil): 3349, 3085, 1730, 1715, 1692 cm^{-1}.
EA: Found: C, 56.83; H, 2.91; N, 8.32. Calc. for $C_{24}H_{14}ClN_3O_6S$: C, 56.76; H, 2.78; N, 8.27.

3-(4-Bromobenzoyl)-2-hydroxy-*N*-(4-nitrophenyl)-1-oxopyrrolo[2,1-*b*][1,3]benzothiazole-3a(1*H*)-carboxamide (12fc)
Yellow solid; yield (80 mg, 32%); mp 214–216 °C (toluene).
^{1}H NMR (400 MHz, DMSO-d_6): δ = 11.02 (s, 1H), 9.84 (br.s, 1H), 8.17 (m, 2H), 7.76 (m, 4H), 7.66 (m, 2H), 7.55 (m, 1H), 7.38 (m, 1H), 7.24–7.16 (m, 2H) ppm.
^{13}C NMR (100 MHz, DMSO-d_6): δ = 186.6, 167.2, 167.1, 161.1, 145.0, 143.3, 137.7, 134.8, 133.9, 131.4 (2C), 131.3 (2C), 127.1, 126.17, 126.1, 125.3 (2C), 123.6, 120.4 (2C), 118.5, 114.2, 82.0 ppm.
IR (mineral oil): 3300, 3171, 1730, 1714, 1677 cm^{-1}.
EA: Found: C, 52.31; H, 2.63; N, 7.74. Calc. for $C_{24}H_{14}BrN_3O_6S$: C, 52.19; H, 2.55; N, 7.61.

2-Hydroxy-*N*-(4-nitrophenyl)-1-oxo-3-(thiophene-2-carbonyl)pyrrolo[2,1-*b*][1,3]benzothiazole-3a(1*H*)-carboxamide (12hc)
Yellow solid; yield (65 mg, 30%); mp 216–218 °C (toluene).
^{1}H NMR (400 MHz, DMSO-d_6): δ = 11.04 (s, 1H), 9.55 (br.s, 1H), 8.65 (m, 1H), 8.17 (m, 2H), 7.92 (m, 1H), 7.75 (m, 2H), 7.54 (m, 1H), 7.36 (m, 1H), 7.25 (m, 2H), 7.17 (m, 1H) ppm.
^{13}C NMR (100 MHz, DMSO-d_6): δ = 177.5, 167.0, 166.4, 160.0, 144.5 (2C), 142.6, 134.3, 133.9, 133.2, 133.0, 128.1, 126.4, 125.5, 124.7 (2C), 123.0, 119.7 (2C), 117.7, 113.5, 81.3 ppm.
IR (mineral oil): 3307, 3154, 1745, 1720, 1653 cm^{-1}.

EA: Found: C, 55.43; H, 2.75; N, 8.81. Calc. for $C_{22}H_{13}N_3O_6S_2$: C, 55.11; H, 2.73; N, 8.76.

***N*-(2-Acetamidophenyl)-3-benzoyl- 2-hydroxy-1-oxopyrrolo[2,1-*b*][1,3]benzothiazole-3a(1*H*)-carboxamide (12ad)**
Beige solid; yield (140 mg, 64%); mp 176–178°C (toluene).
^{1}H NMR (400 MHz, DMSO-d_6): δ = 9.92 (s, 1H), 9.71 (s, 1H), 9.10 (br.s, 1H), 7.82 (m, 2H), 7.62 (m, 1H), 7.55–7.48 (m, 5H), 7.25 (m, 2H), 7.15 (m, 3H), 2.01 (s, 3H) ppm.
^{13}C NMR (100 MHz, DMSO-d_6): δ = 188.1, 169.3, 166.3, 165.5, 154.6, 137.3, 133.7, 133.6, 132.6, 131.2, 130.2, 128.8 (2C), 128.1 (2C), 126.7, 125.8, 125.6, 125.5, 124.9, 124.5, 123.3, 118.2, 117.9, 80.6, 22.8 ppm.
IR (mineral oil): 3288, 3132, 1733, 1673, 1648, 1638 cm^{-1}.
EA: Found: C 64.46; H 3.99; N 8.70. Calc. for $C_{26}H_{19}N_3O_5S$: C 64.32; H 3.94; N 8.65.

***N*-(2-Acetamidophenyl)-3-(4-methylbenzoyl)-2-hydroxy-1-oxopyrrolo[2,1-*b*][1,3]benzothiazole-3a(1*H*)-carboxamide (12bd)**
Beige solid; yield (157 mg, 70%); mp 197–199°C (toluene).
^{1}H NMR (400 MHz, DMSO-d_6): δ = 9.88 (s, 1H), 9.71 (s, 1H), 7.74 (m, 2H), 7.54 (m, 1H), 7.44 (m, 2H), 7.32 (m, 2H), 7.24 (m, 2H), 7.15 (m, 3H), 2.39 (s, 3H), 2.02 (s, 3H) ppm.
^{13}C NMR (100 MHz, DMSO-d_6): δ = 188.4, 169.9, 166.9, 166.0, 154.1, 143.9, 135.2, 134.3, 134.3, 131.7, 130.8, 129.6 (2C), 129.3 (2C), 127.2, 126.4, 126.2, 126.1, 125.4, 125.1, 123.9, 119.2, 118.5, 81.2, 23.4, 21.7 ppm.
IR (mineral oil): 3288, 3188, 1717, 1673, 1651 cm^{-1}.
EA: Found: C, 65.15; H, 4.41; N, 8.50. Calc. for $C_{27}H_{21}N_3O_5S$: C, 64.92; H, 4.24; N, 8.41.

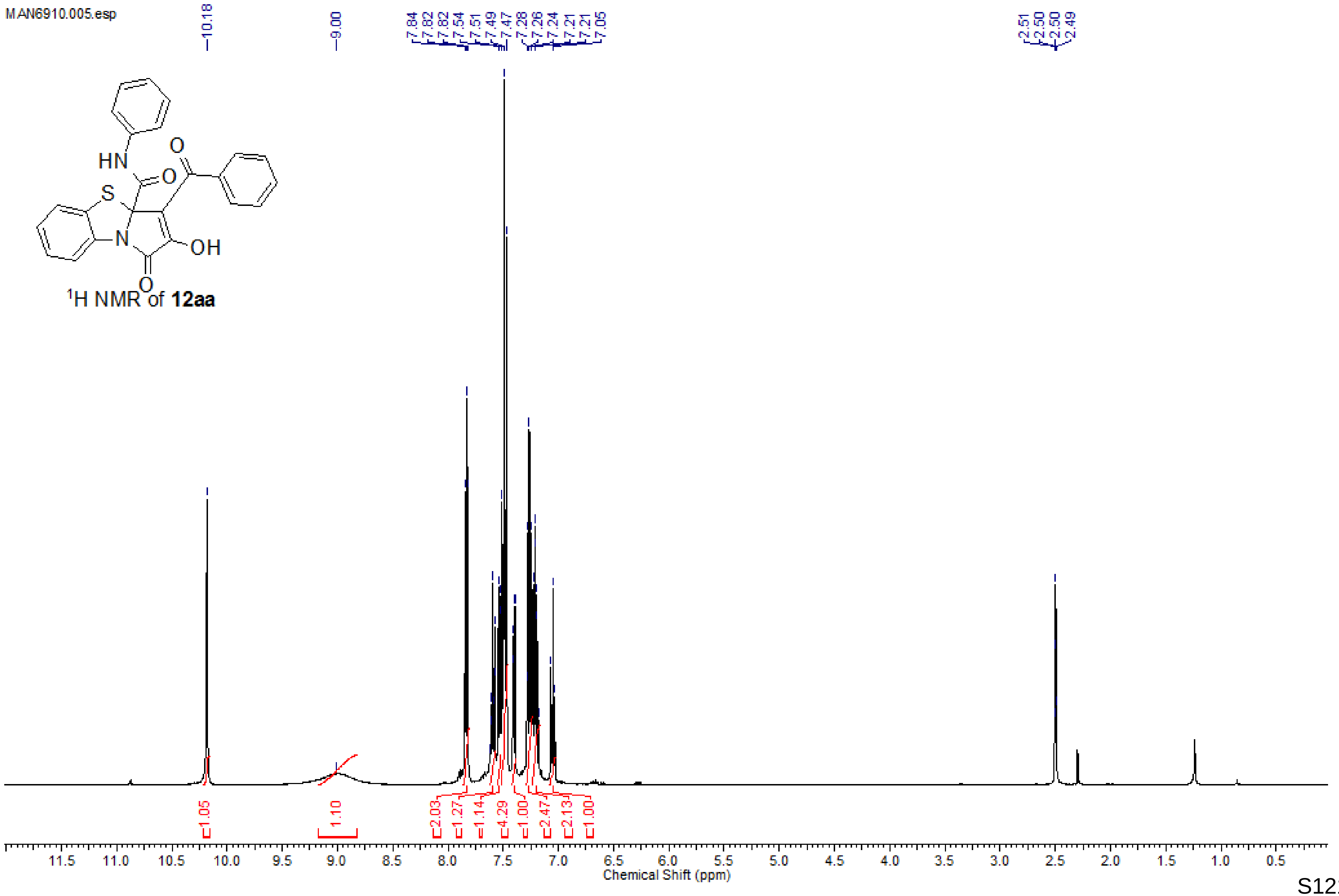

^{1}H NMR of **12aa**

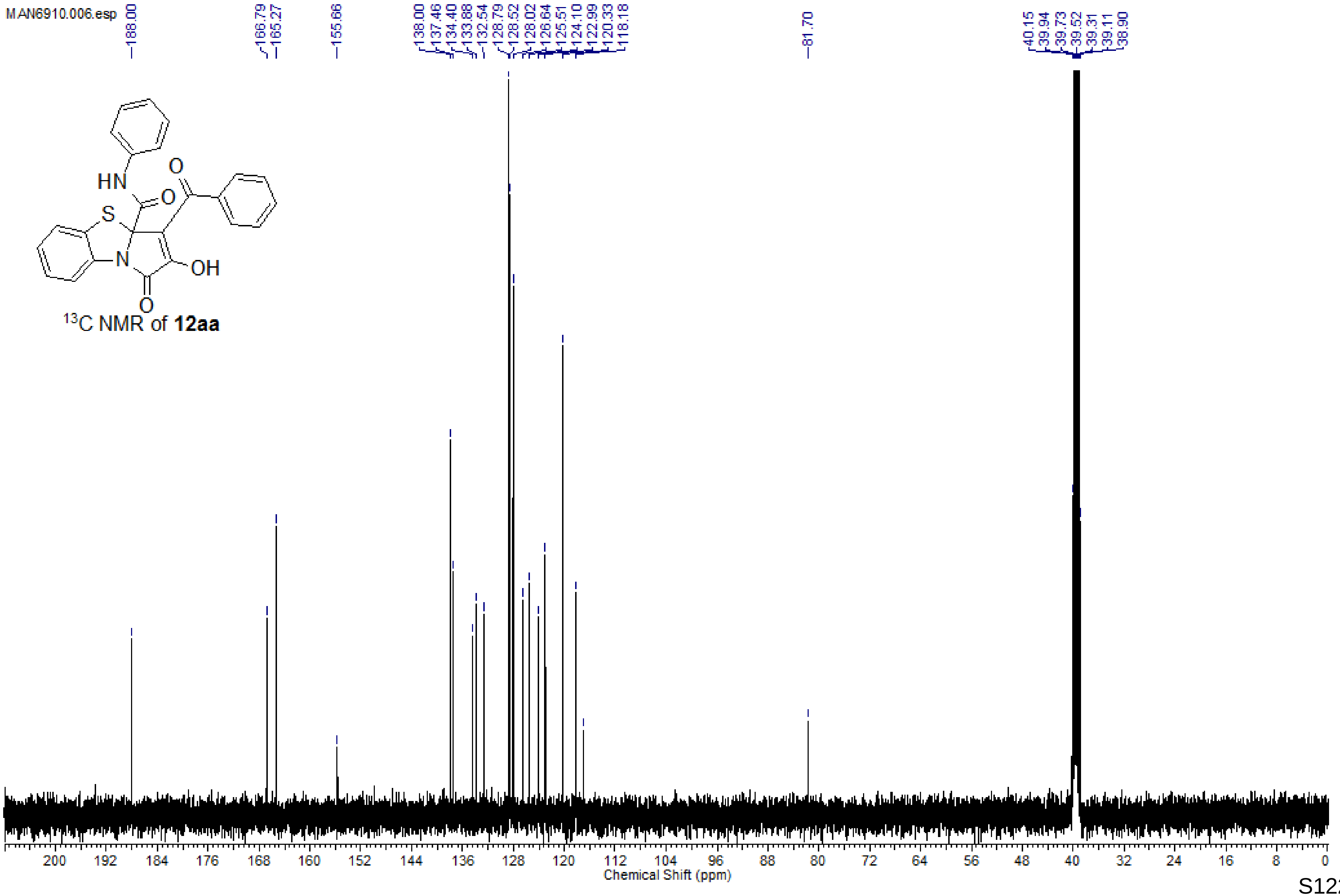

^{13}C NMR of **12aa**

MAN6910.007.esp

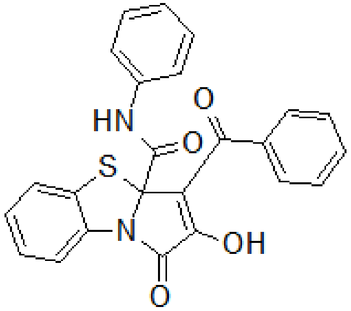

DEPT135 of **12aa**

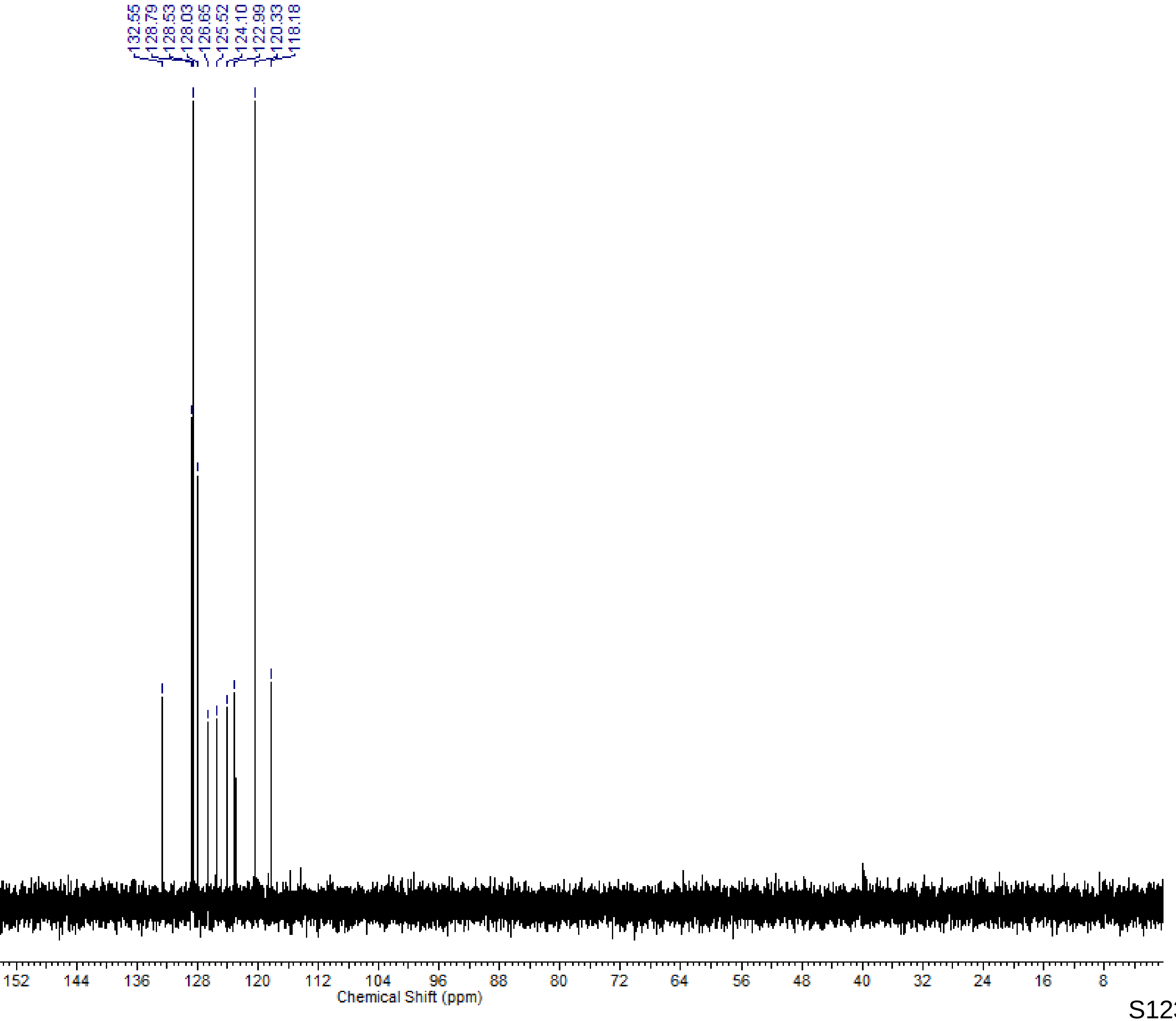

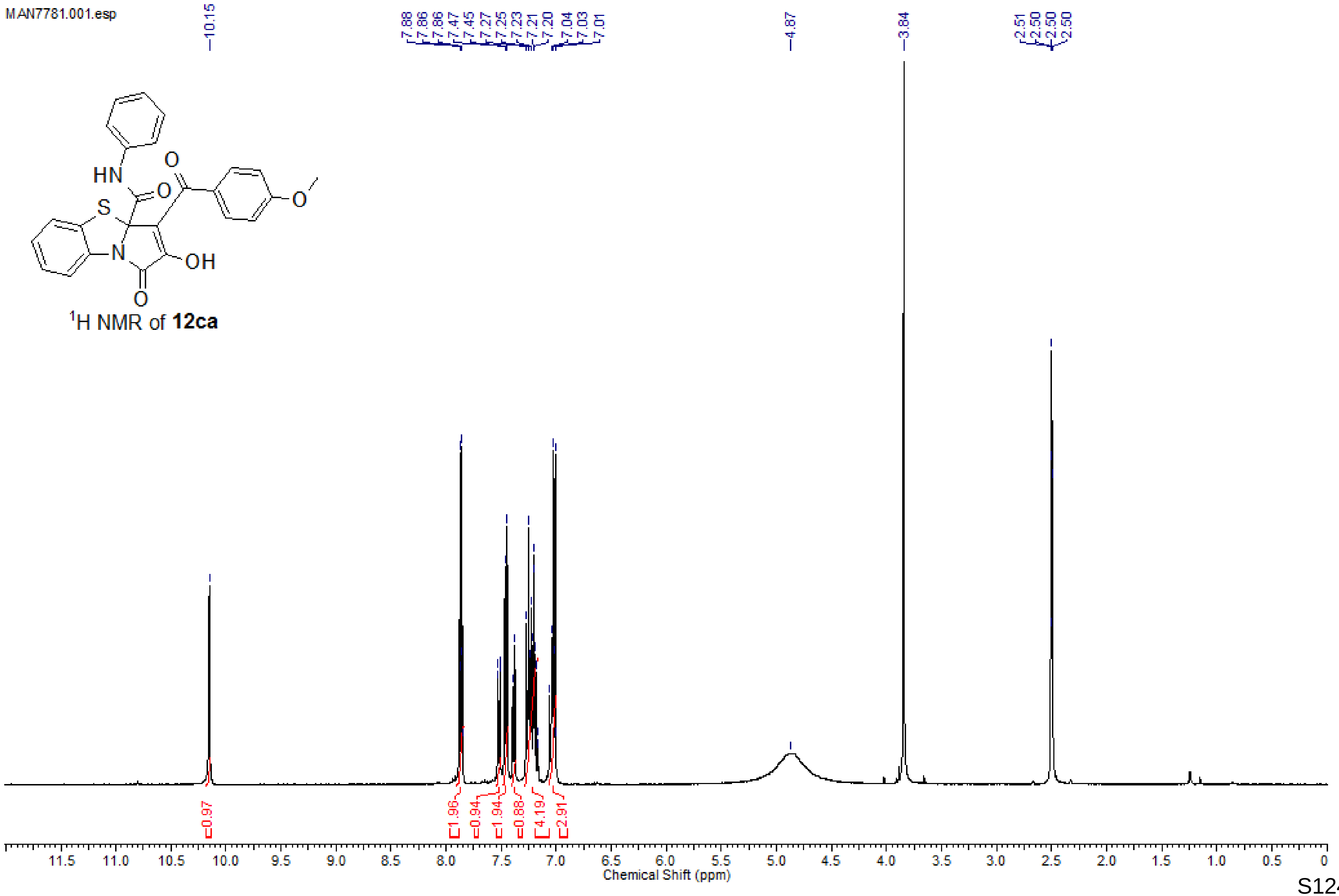

^{1}H NMR of **12ca**

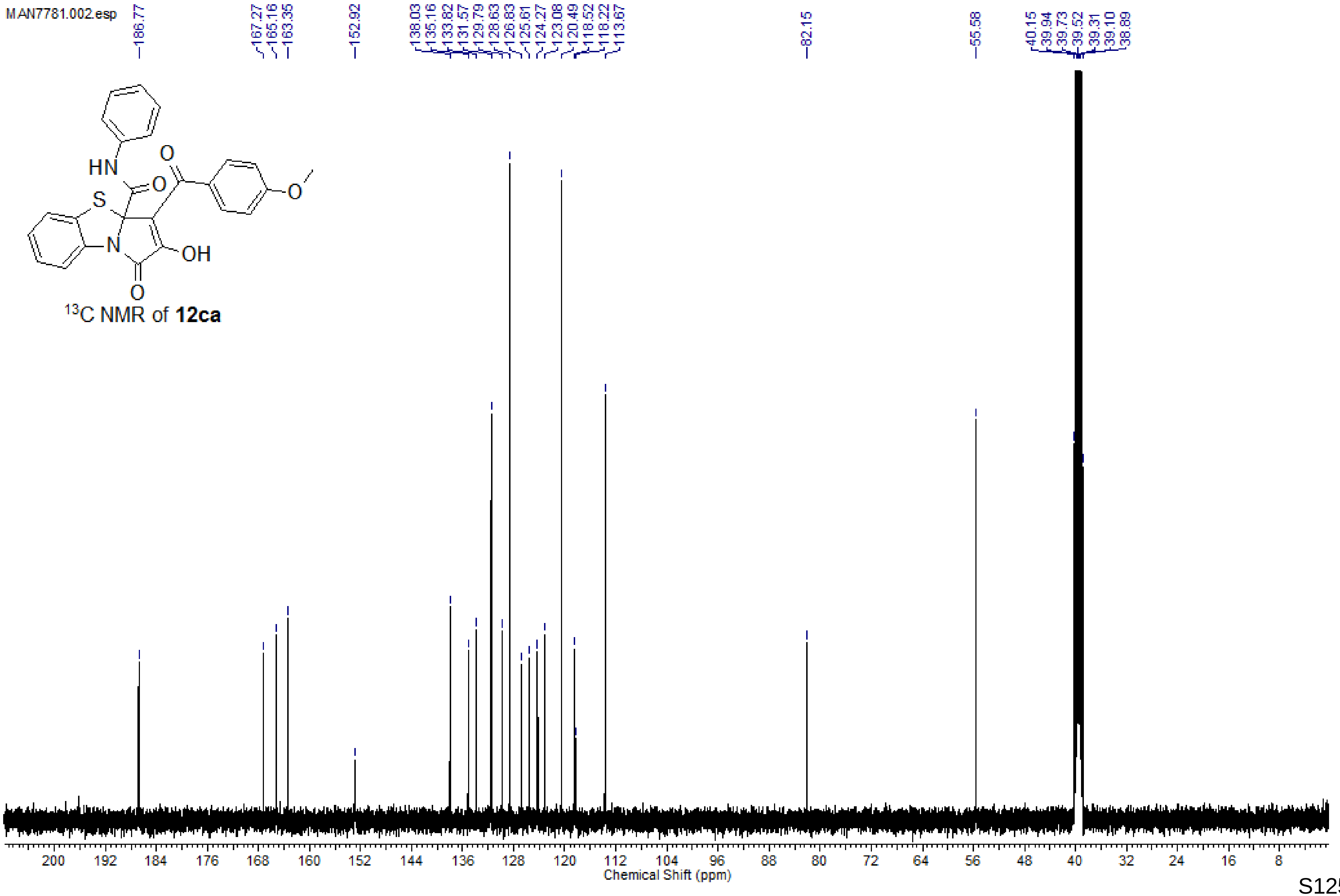

^{13}C NMR of **12ca**

MAN7781.003.esp

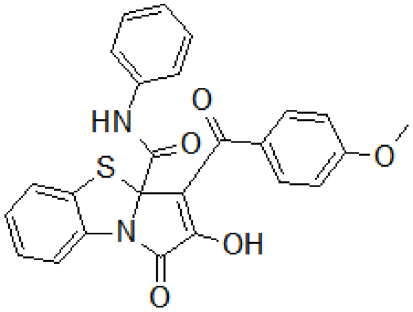

DEPT135 of **12ca**

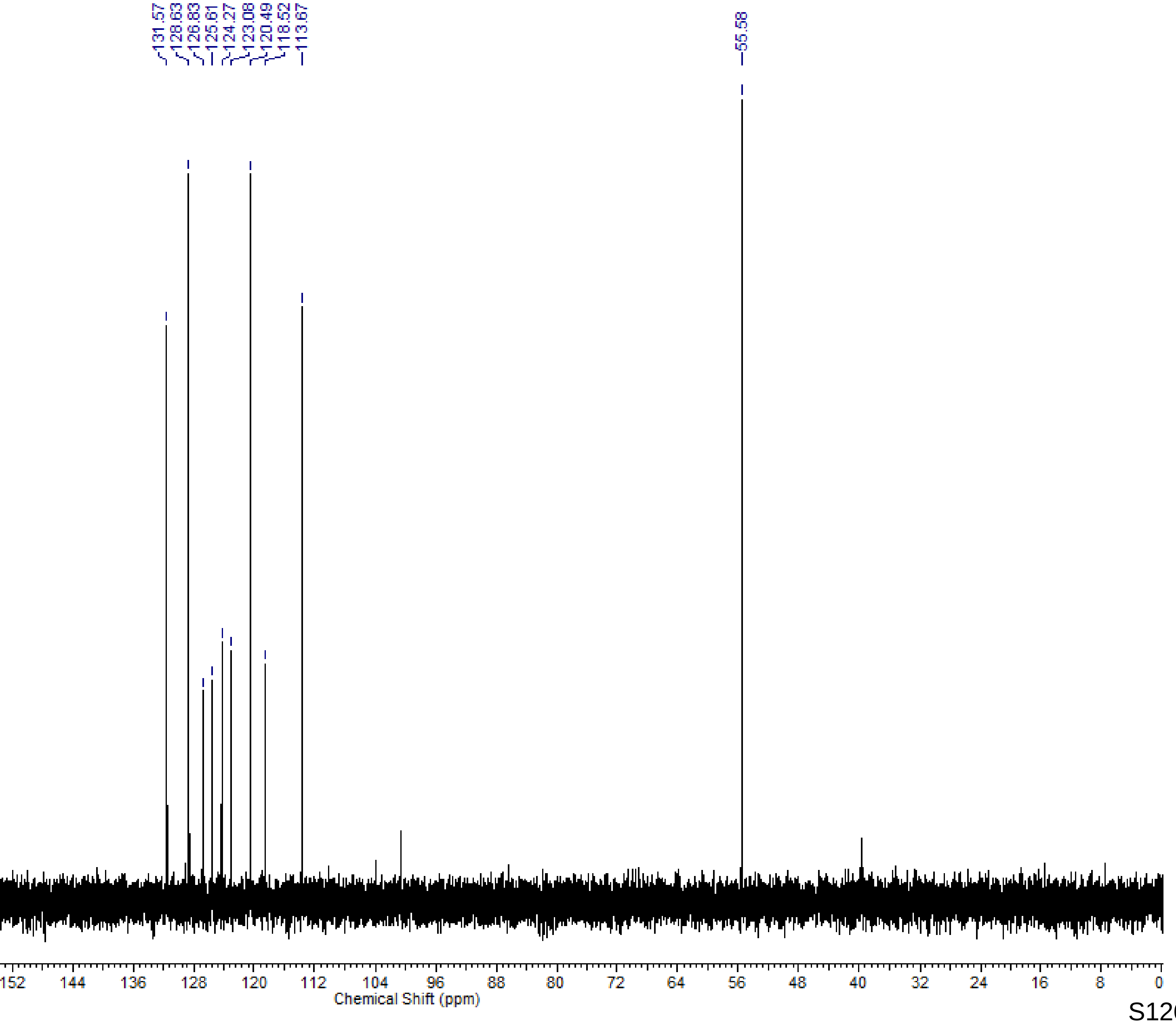

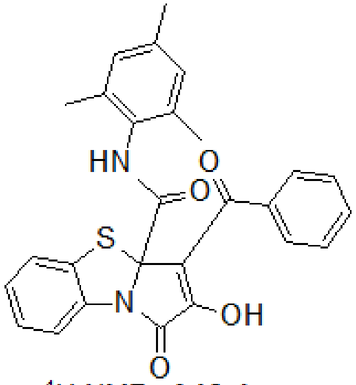

^{1}H NMR of **12ab**

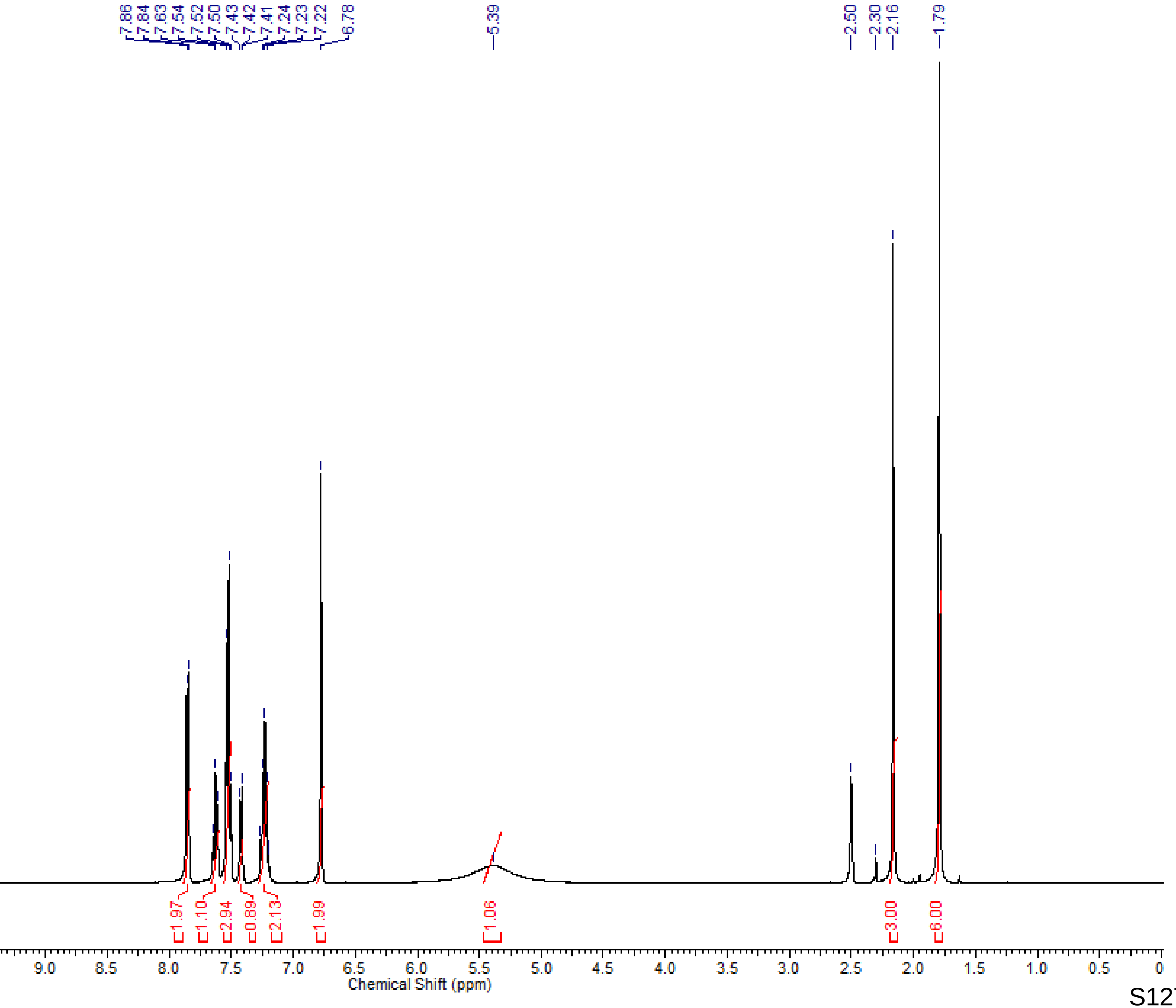

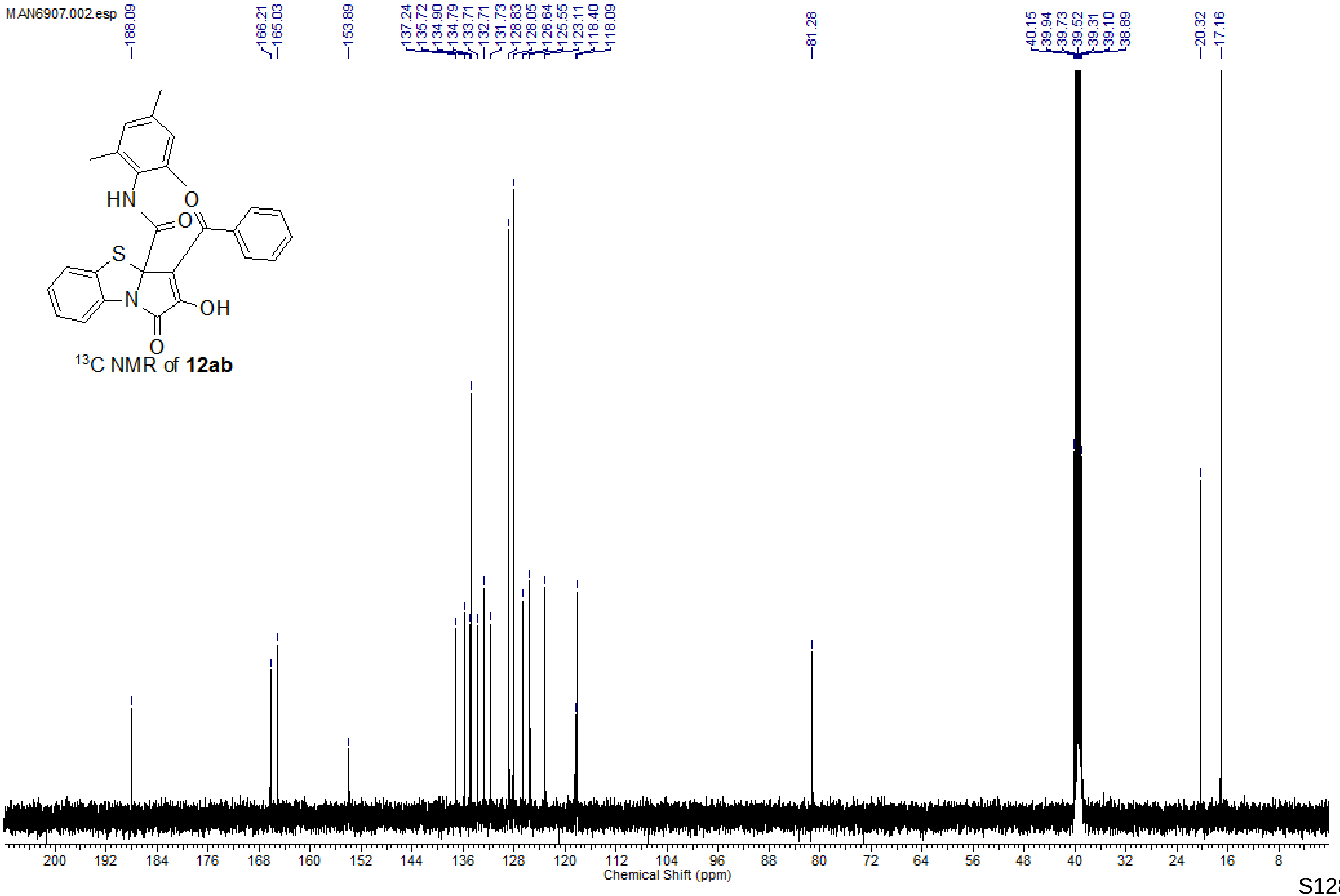

^{13}C NMR of **12ab**

MAN6907.003.esp

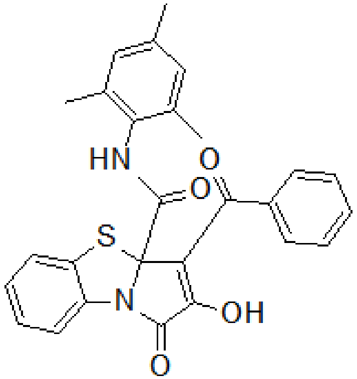

DEPT135 of **12ab**

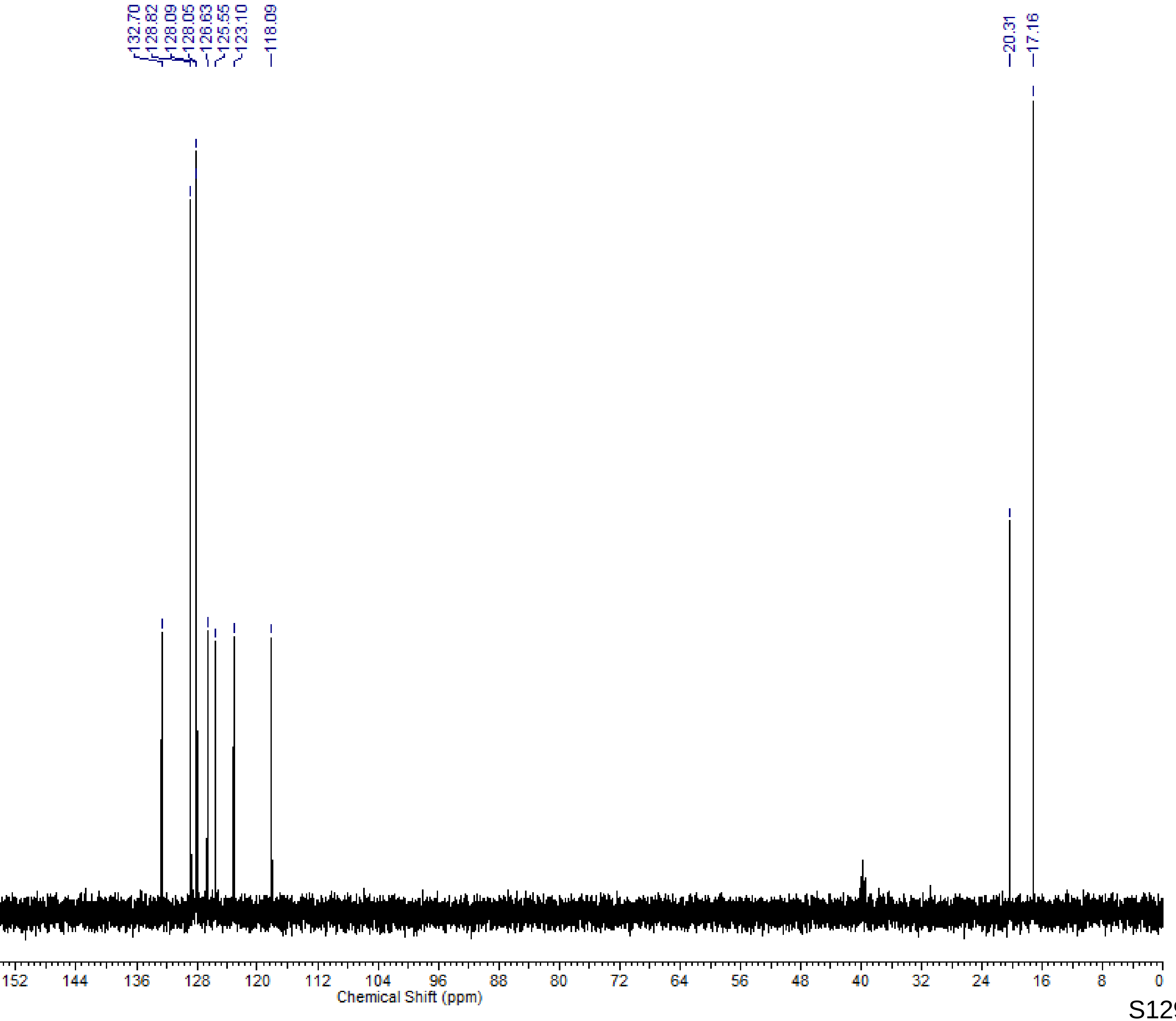

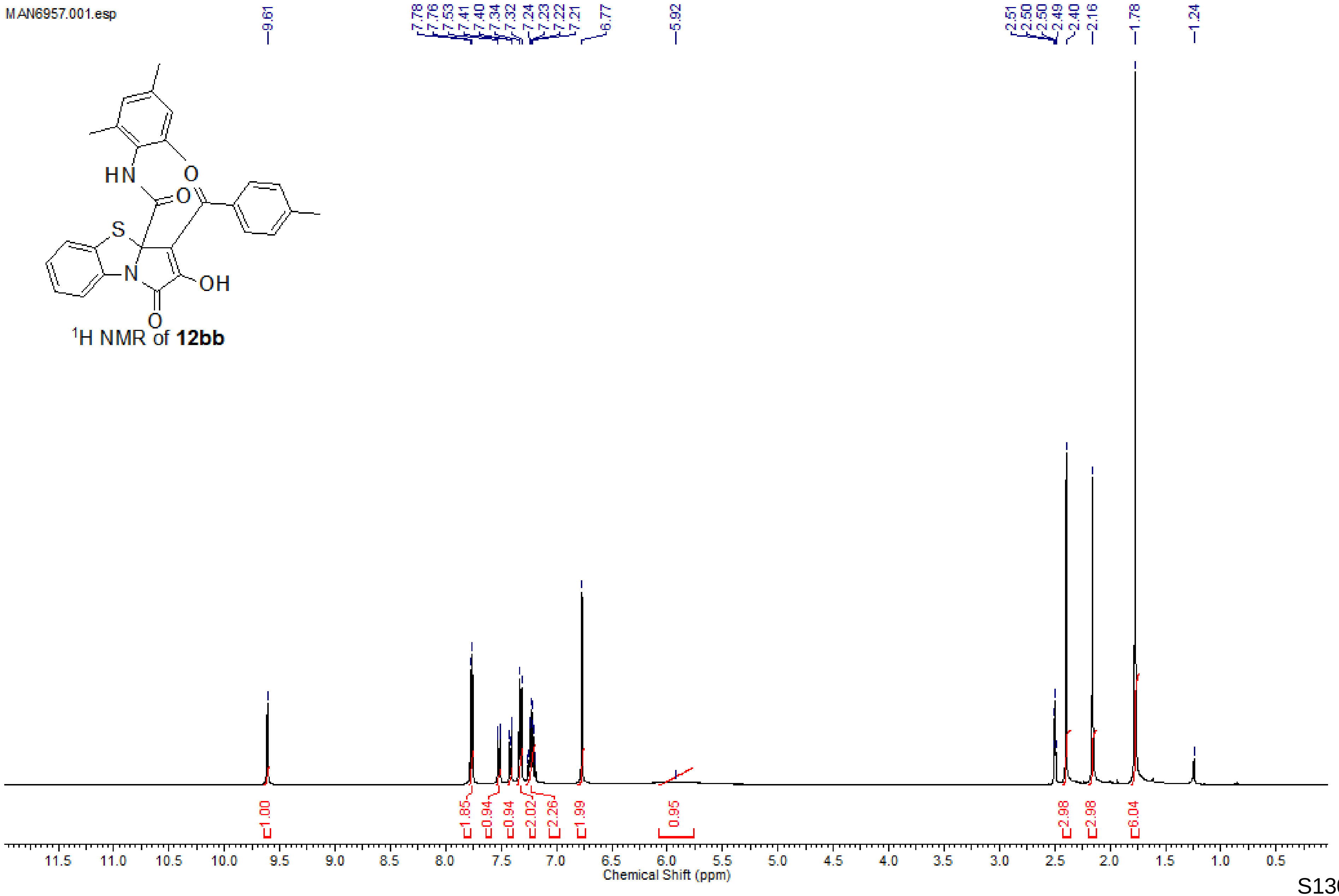

^{1}H NMR of **12bb**

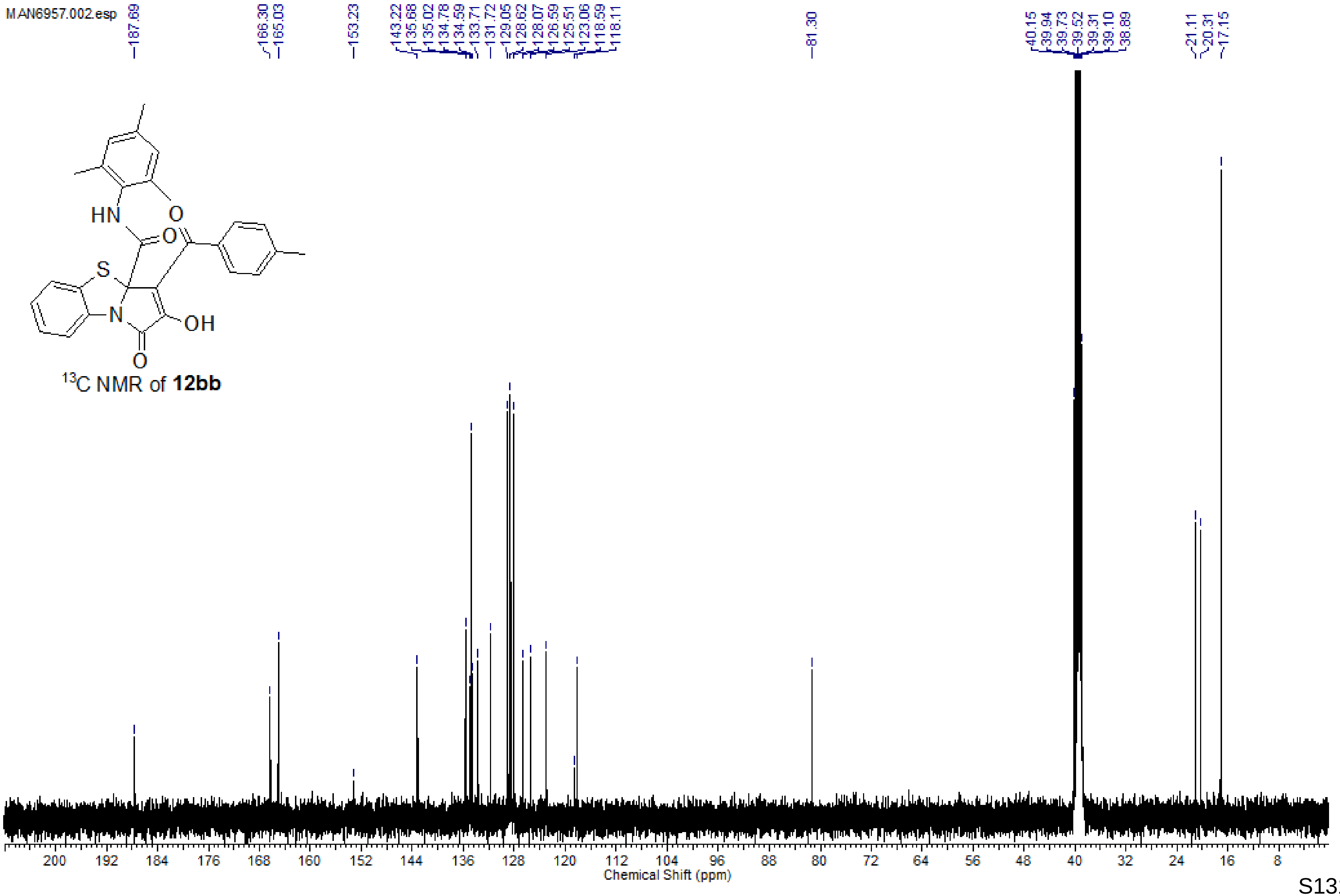

^{13}C NMR of **12bb**

MAN6957.003.esp

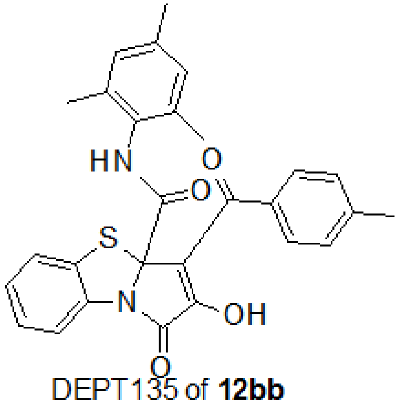

DEPT135 of **12bb**

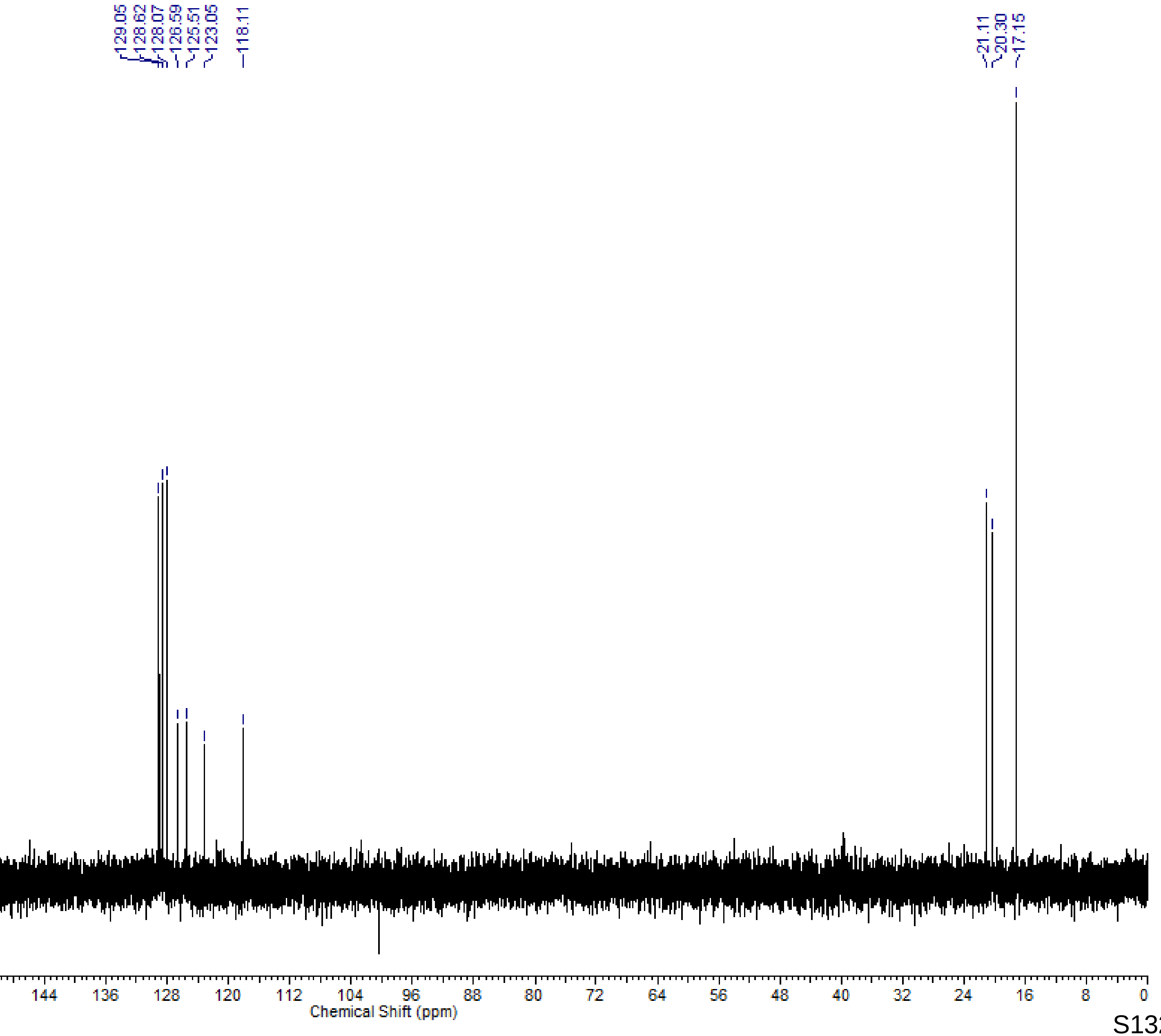

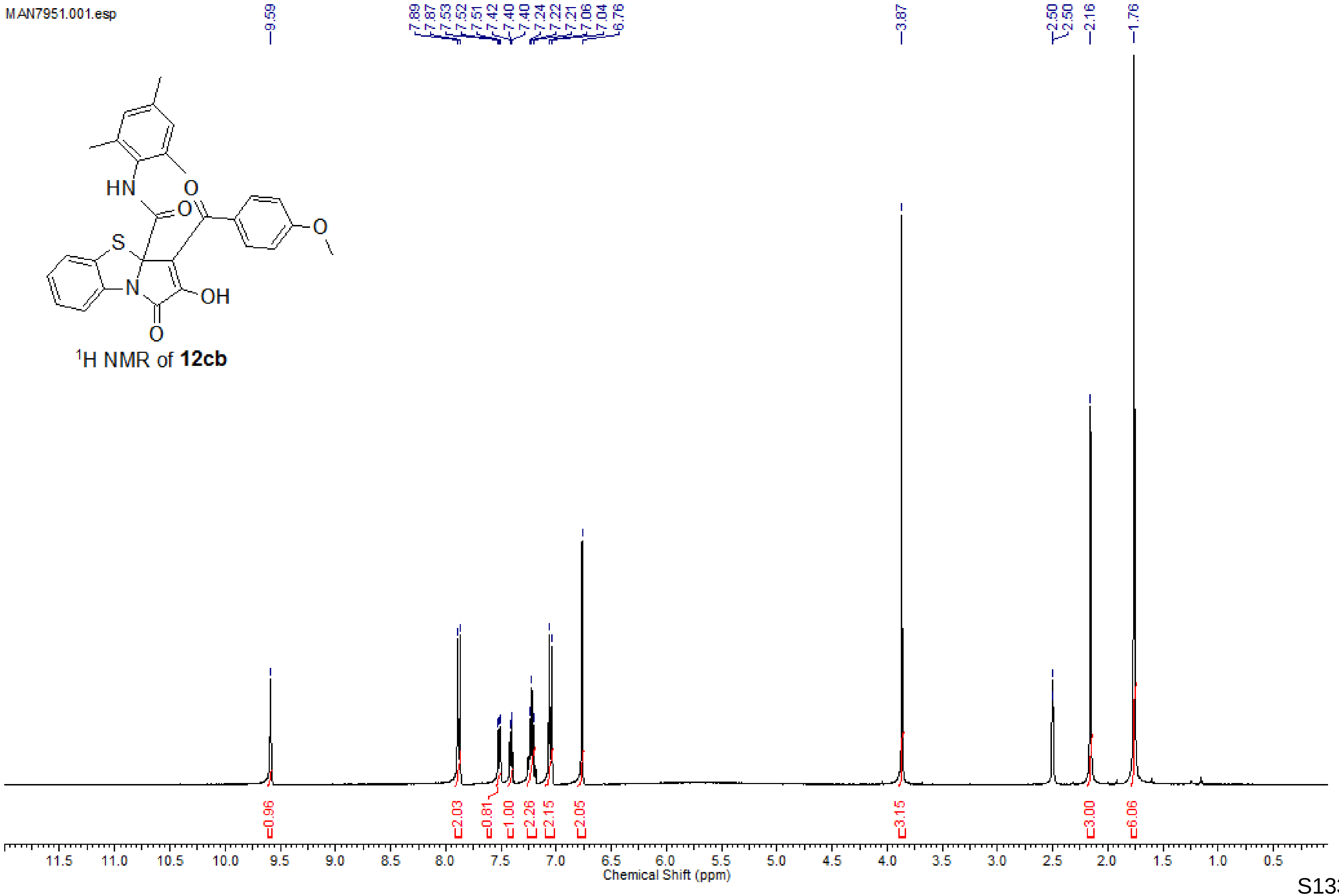

^{1}H NMR of **12cb**

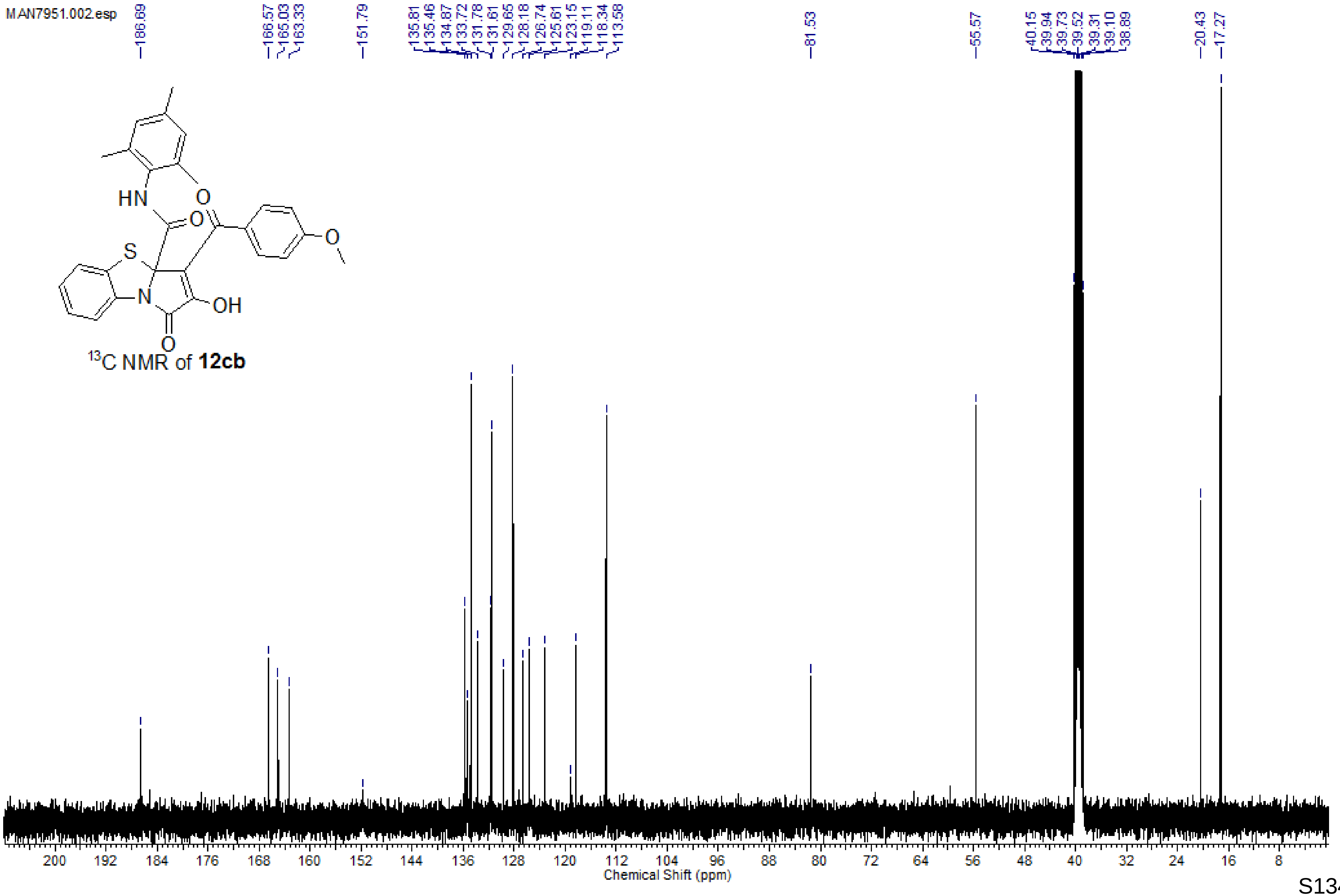

^{13}C NMR of **12cb**

MAN7951.003.esp

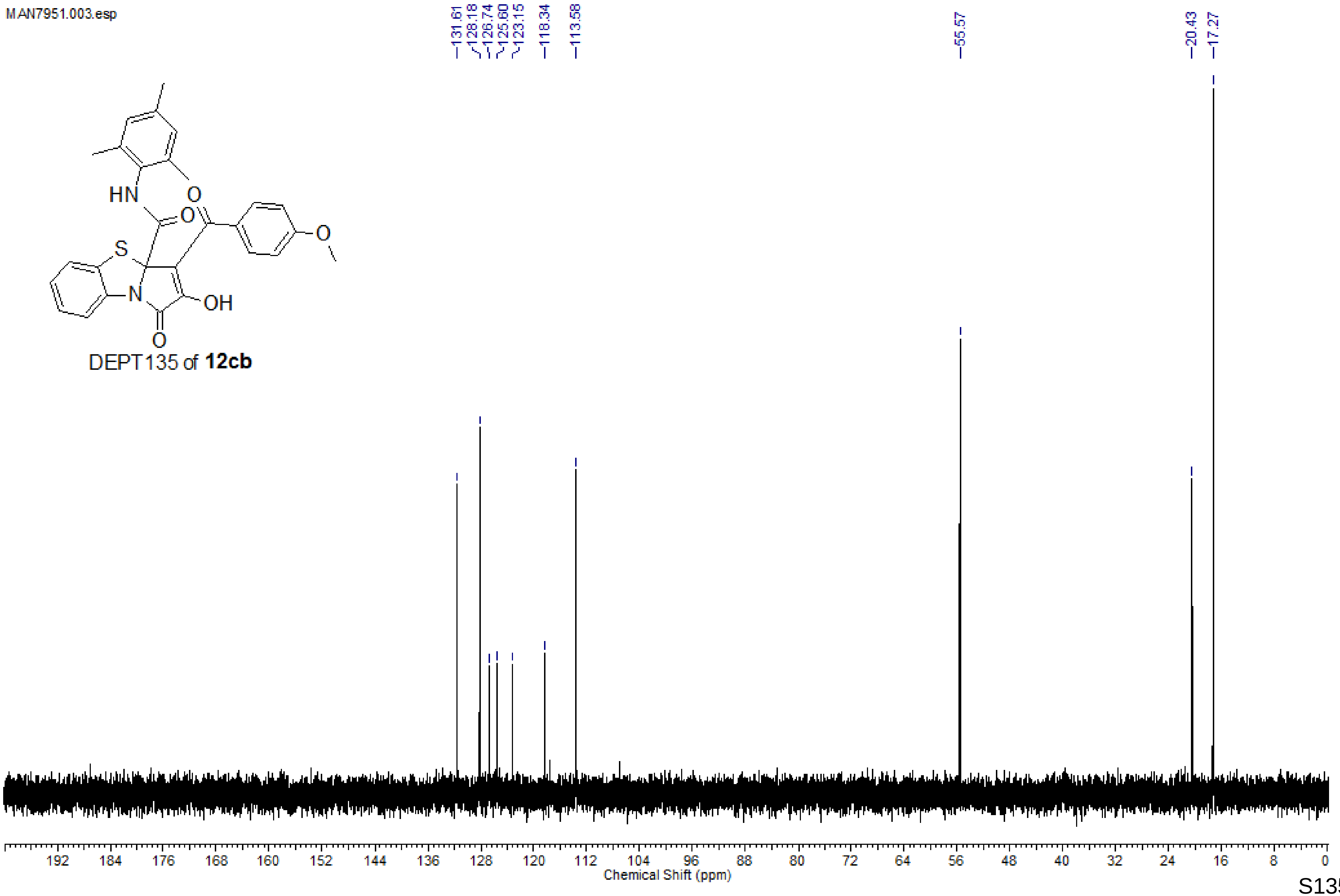

DEPT135 of **12cb**

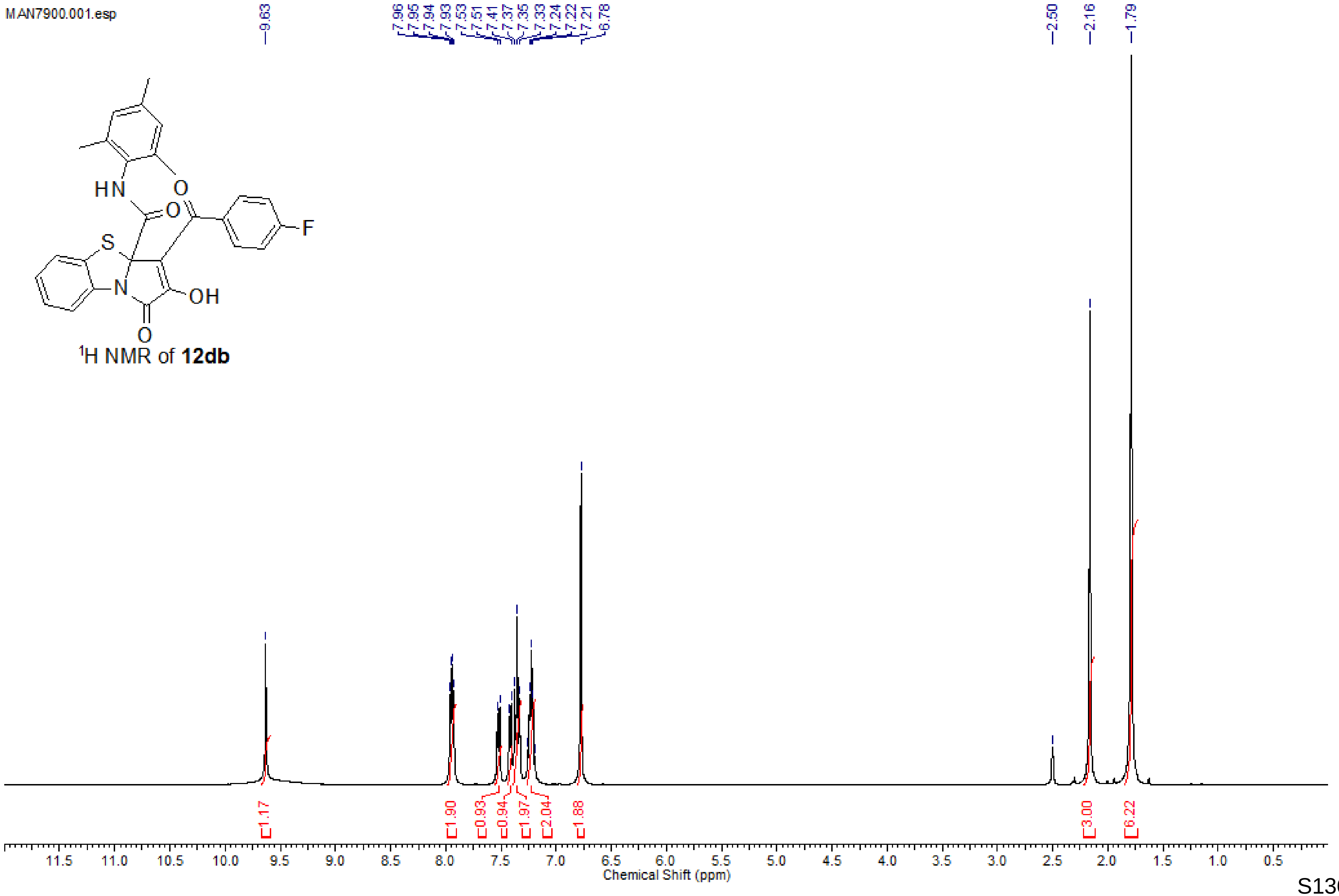

^{1}H NMR of **12db**

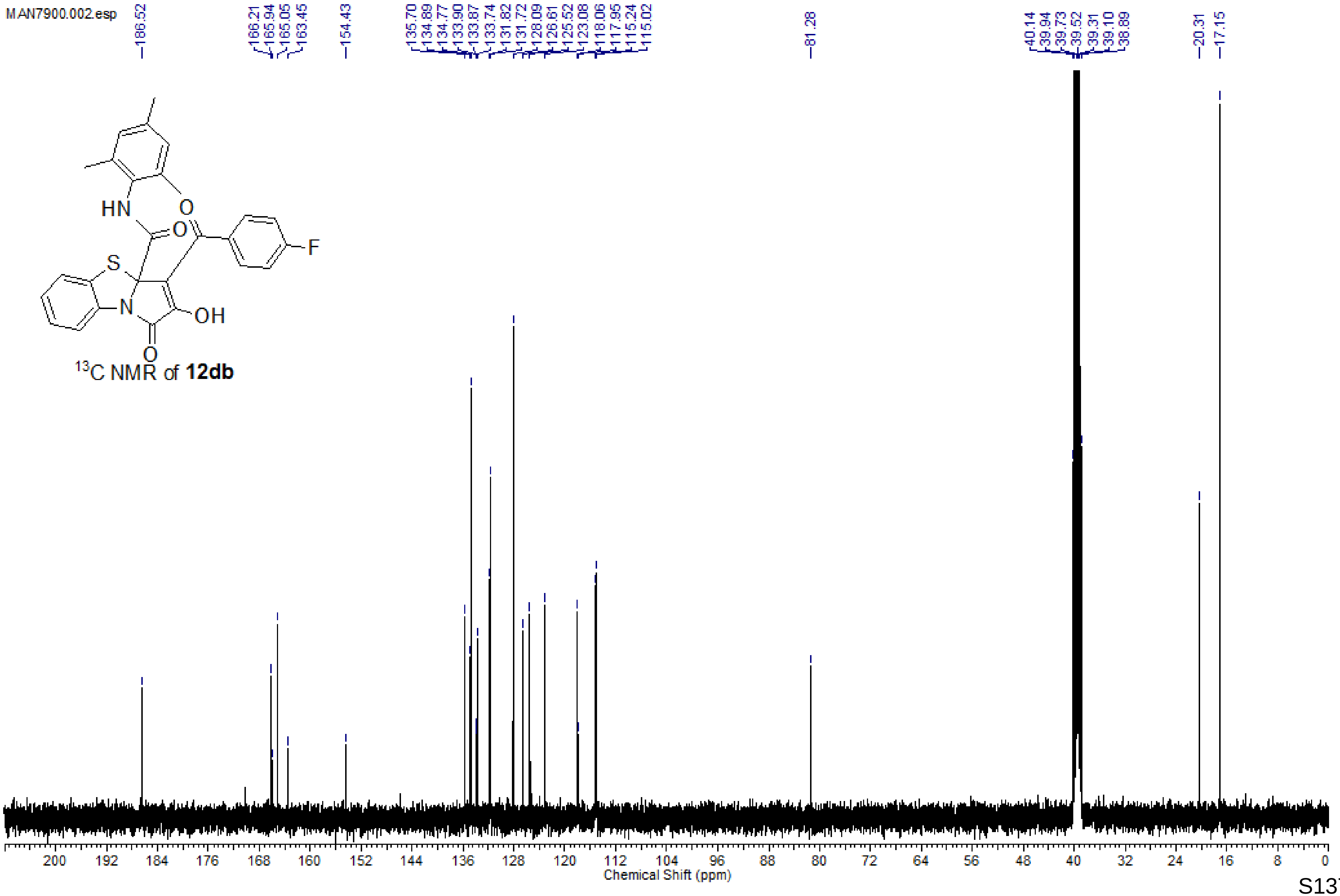

^{13}C NMR of **12db**

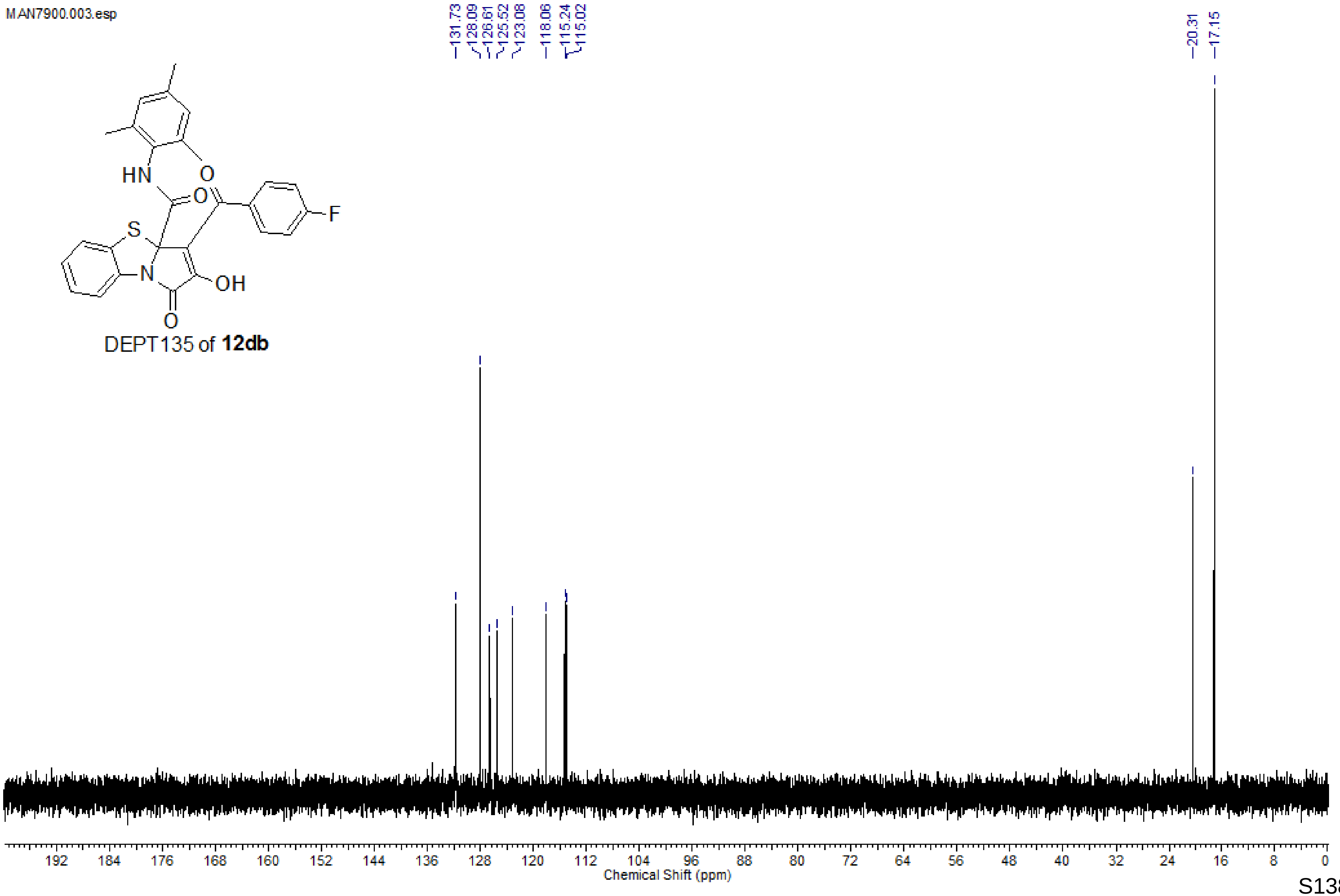

DEPT135 of **12db**

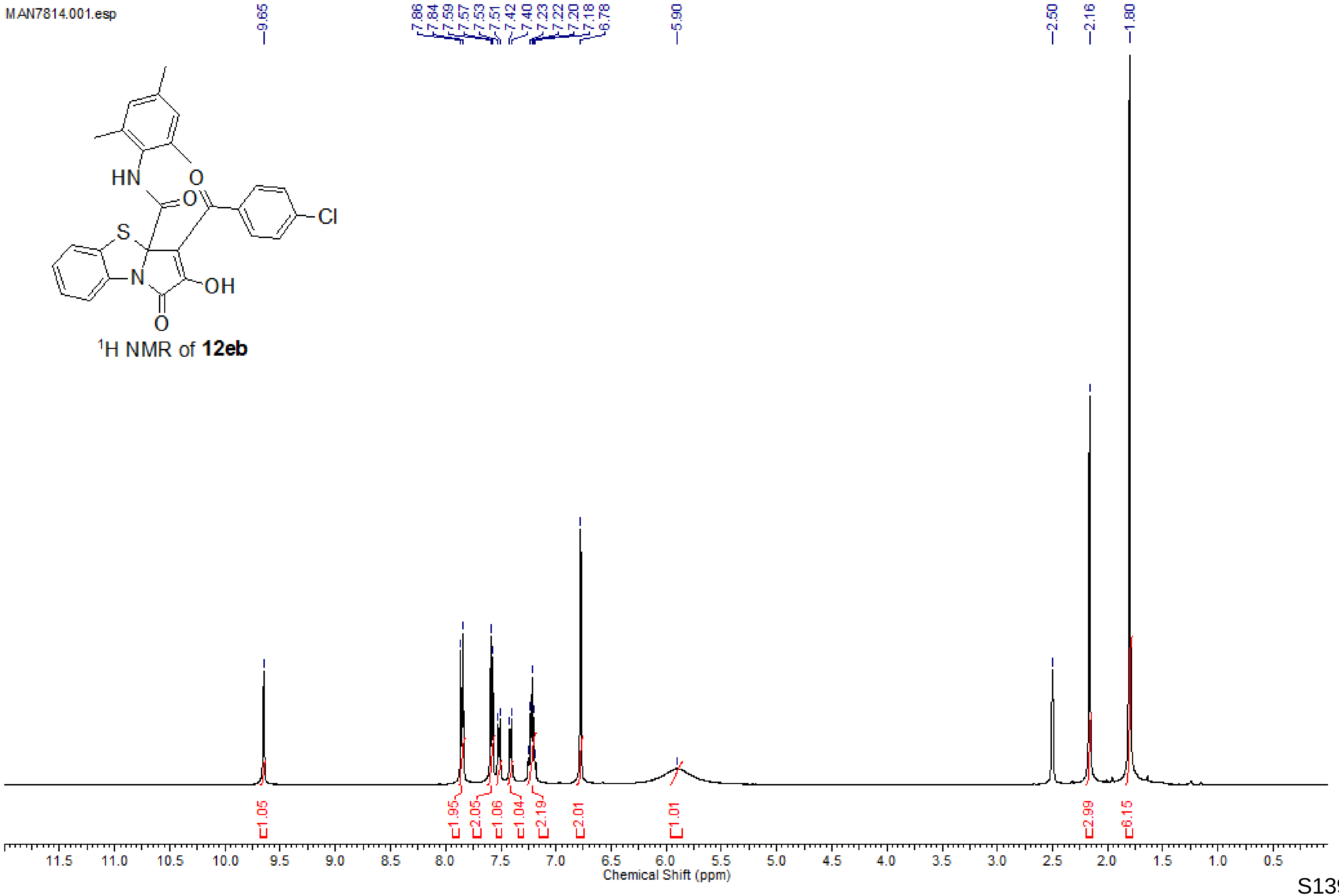

^{1}H NMR of **12eb**

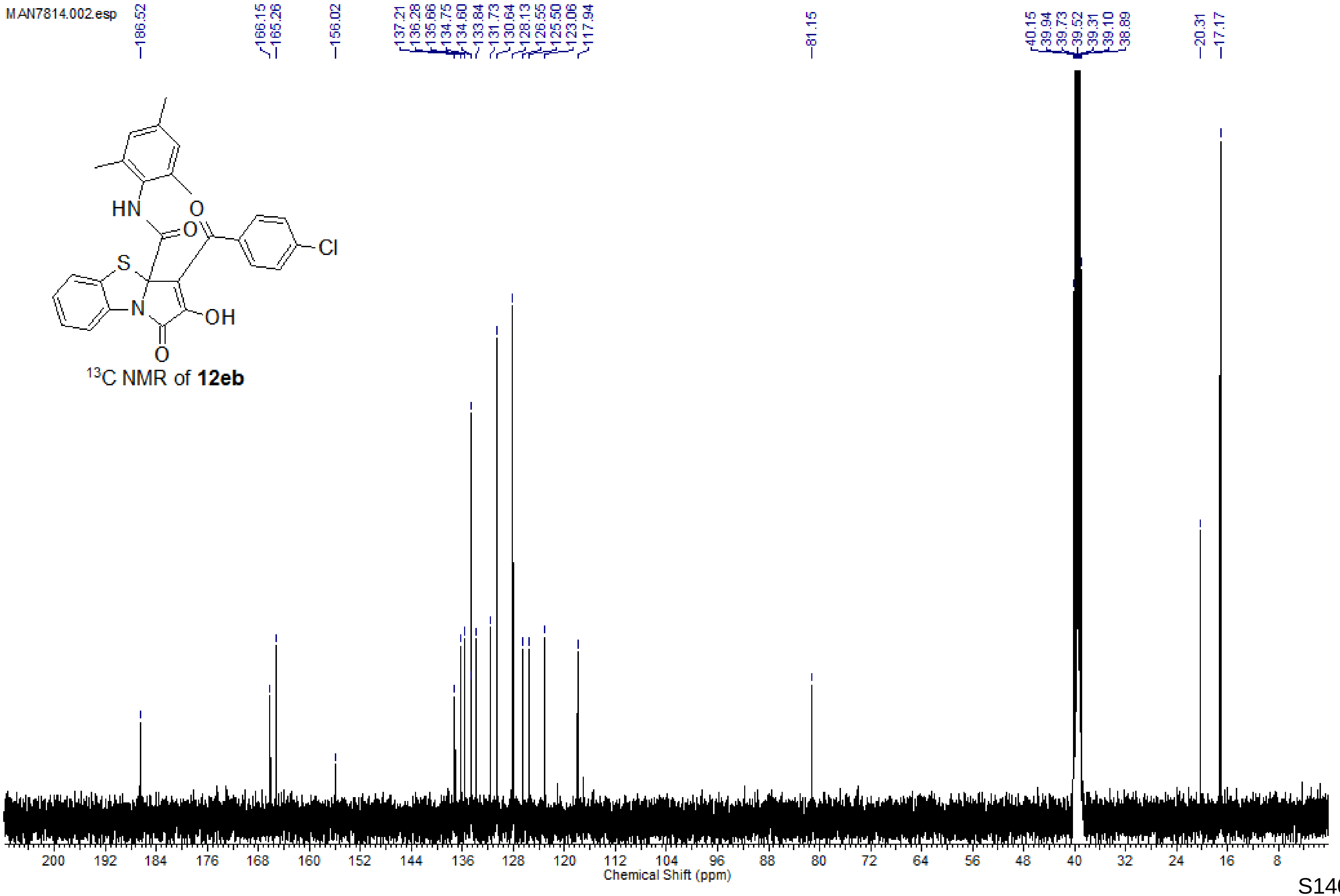
MAN7814.002.esp
186.52
166.15
165.26
156.02
137.21
136.28
135.66
134.75
134.60
133.84
131.73
130.64
128.13
126.55
125.50
123.06
117.94
81.15
40.15
39.94
39.73
39.52
39.31
39.10
38.89
20.31
17.17
HN
O
O
Cl
S
N
OH
O
^{13}C NMR of **12eb**
Chemical Shift (ppm)

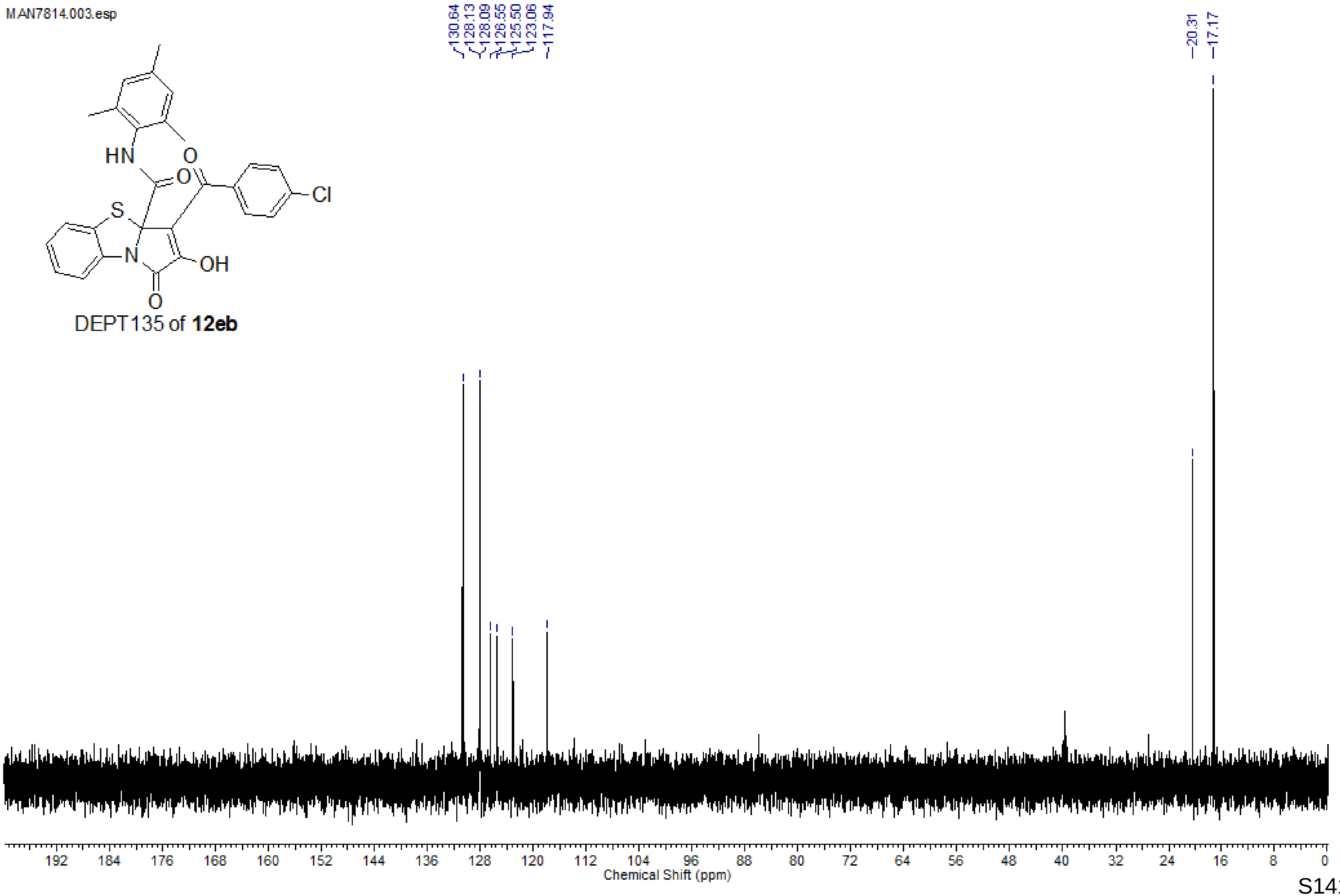

DEPT135 of **12eb**

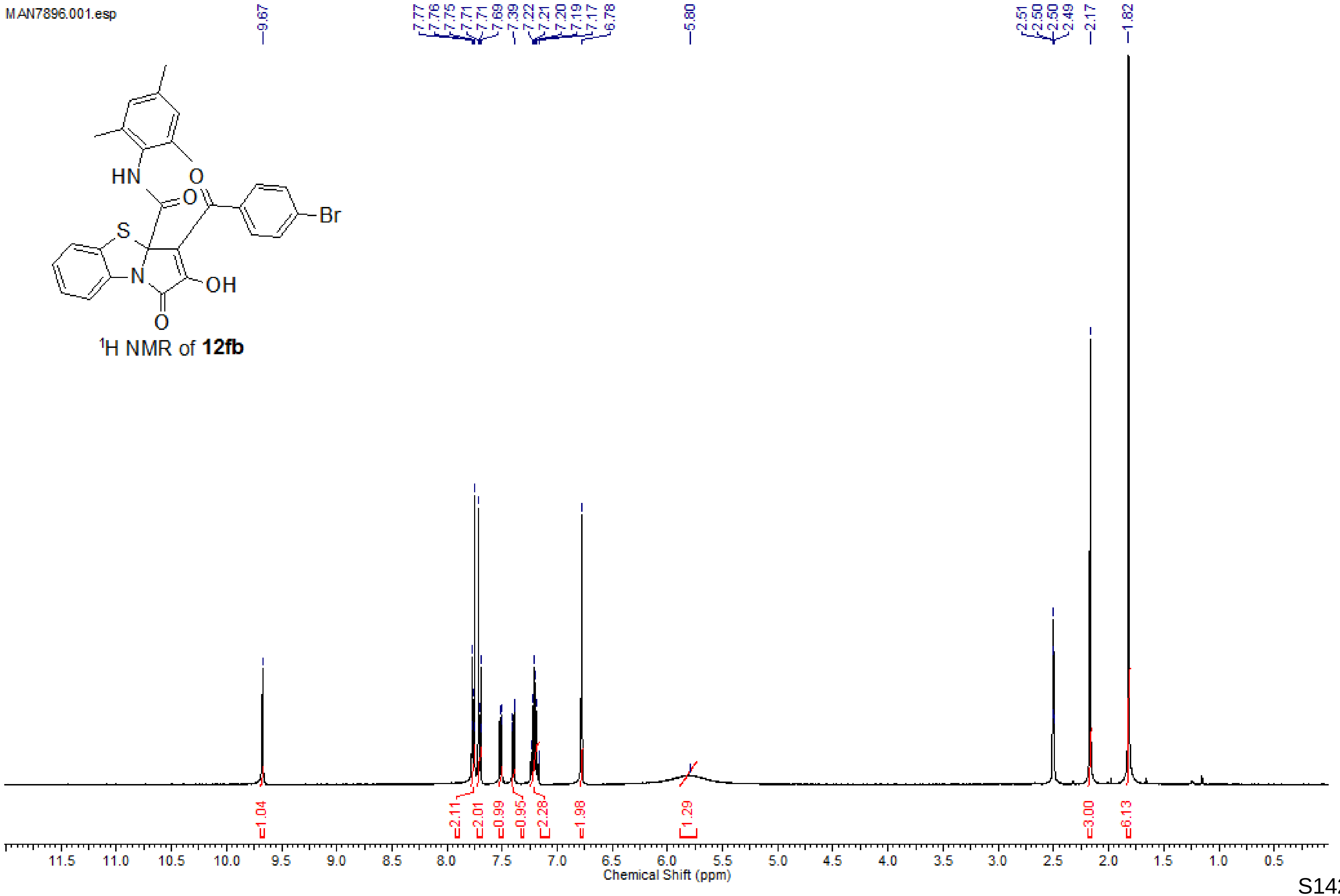

^{1}H NMR of **12fb**

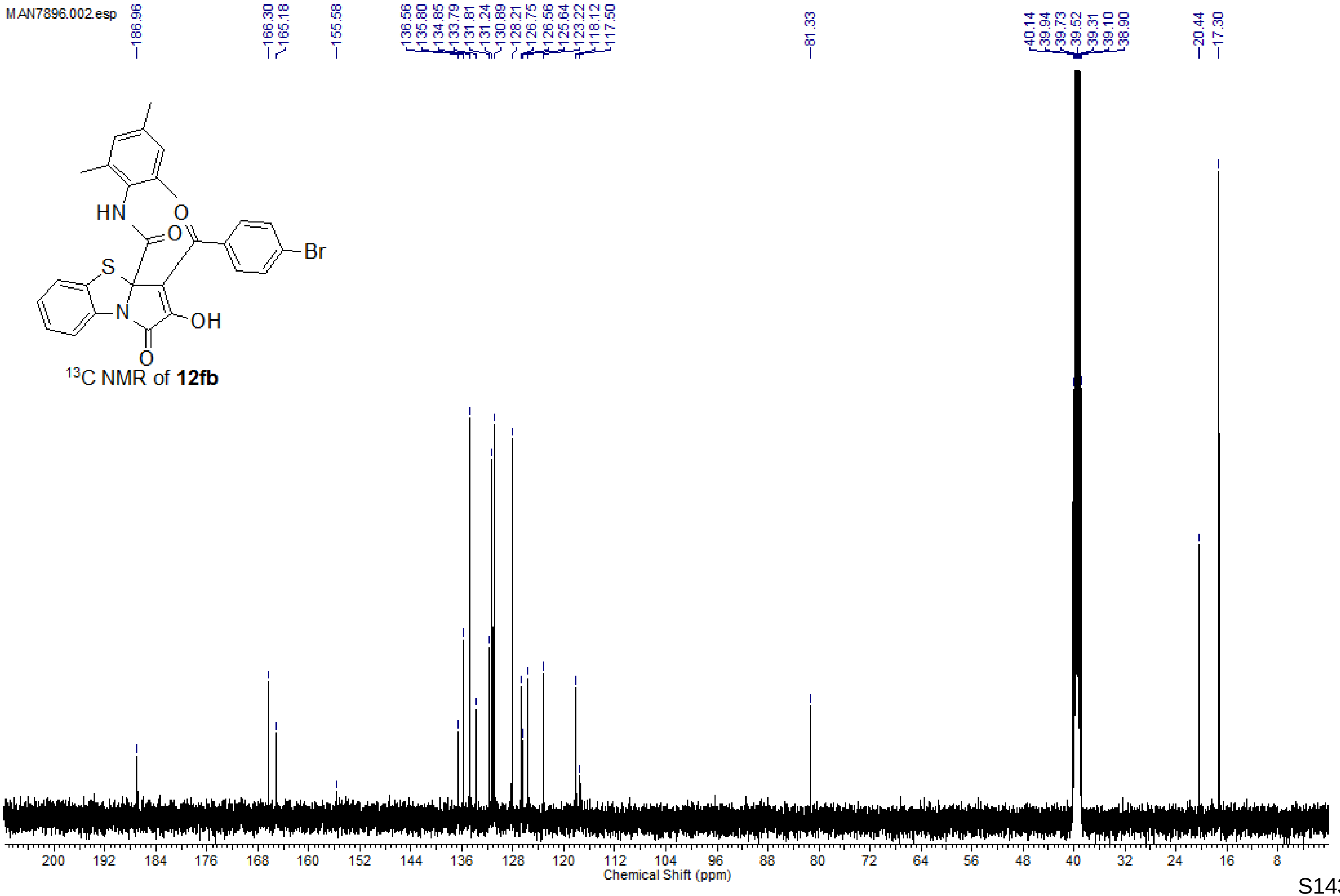

^{13}C NMR of **12fb**

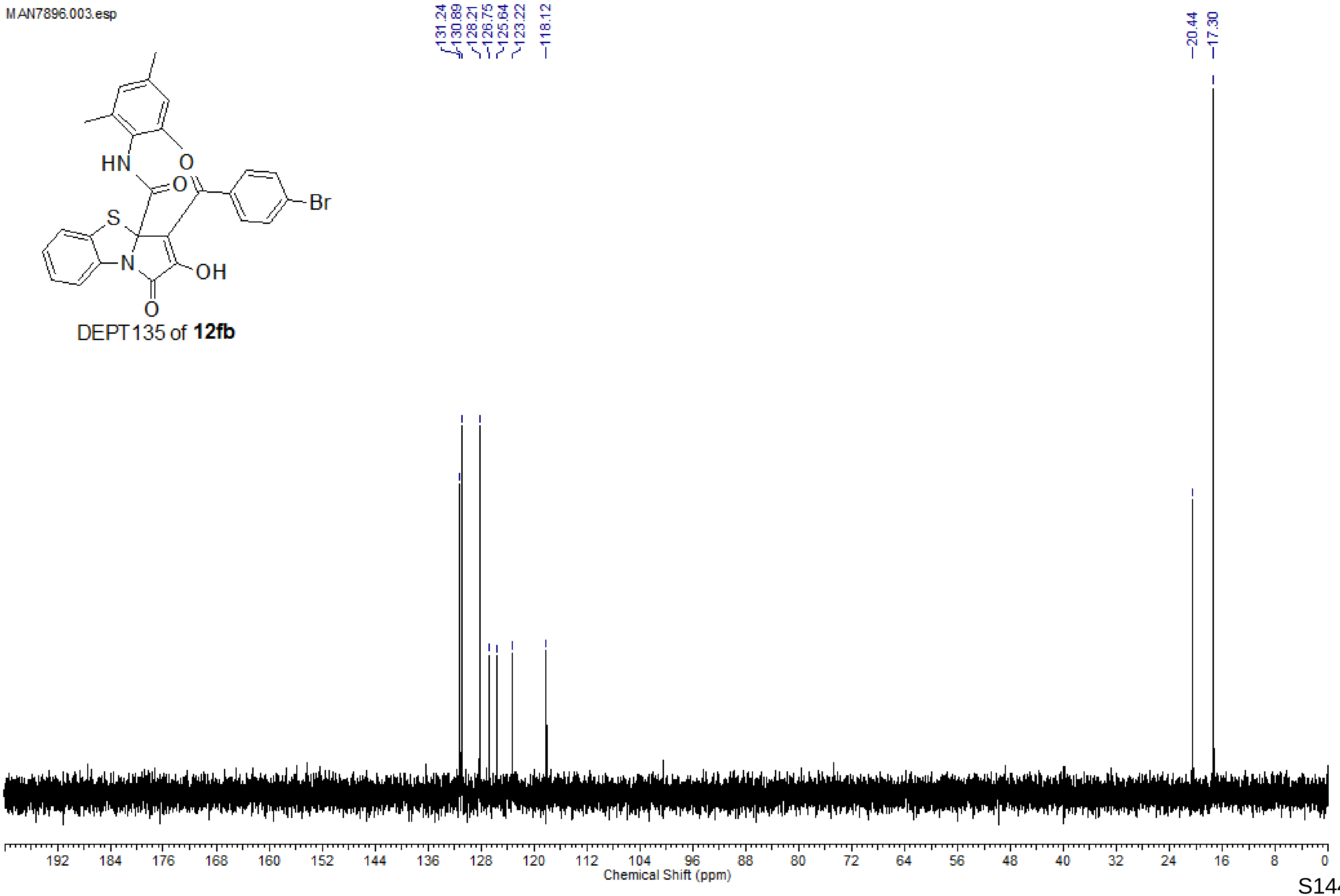
MAN7896.003.esp
HN
O
O
O
S
Br
N
OH
O
DEPT135 of 12fb
131.24
130.89
128.21
126.75
125.64
123.22
118.12
20.44
17.30
192
184
176
168
160
152
144
136
128
120
112
104
96
88
80
72
64
56
48
40
32
24
16
8
0
Chemical Shift (ppm)

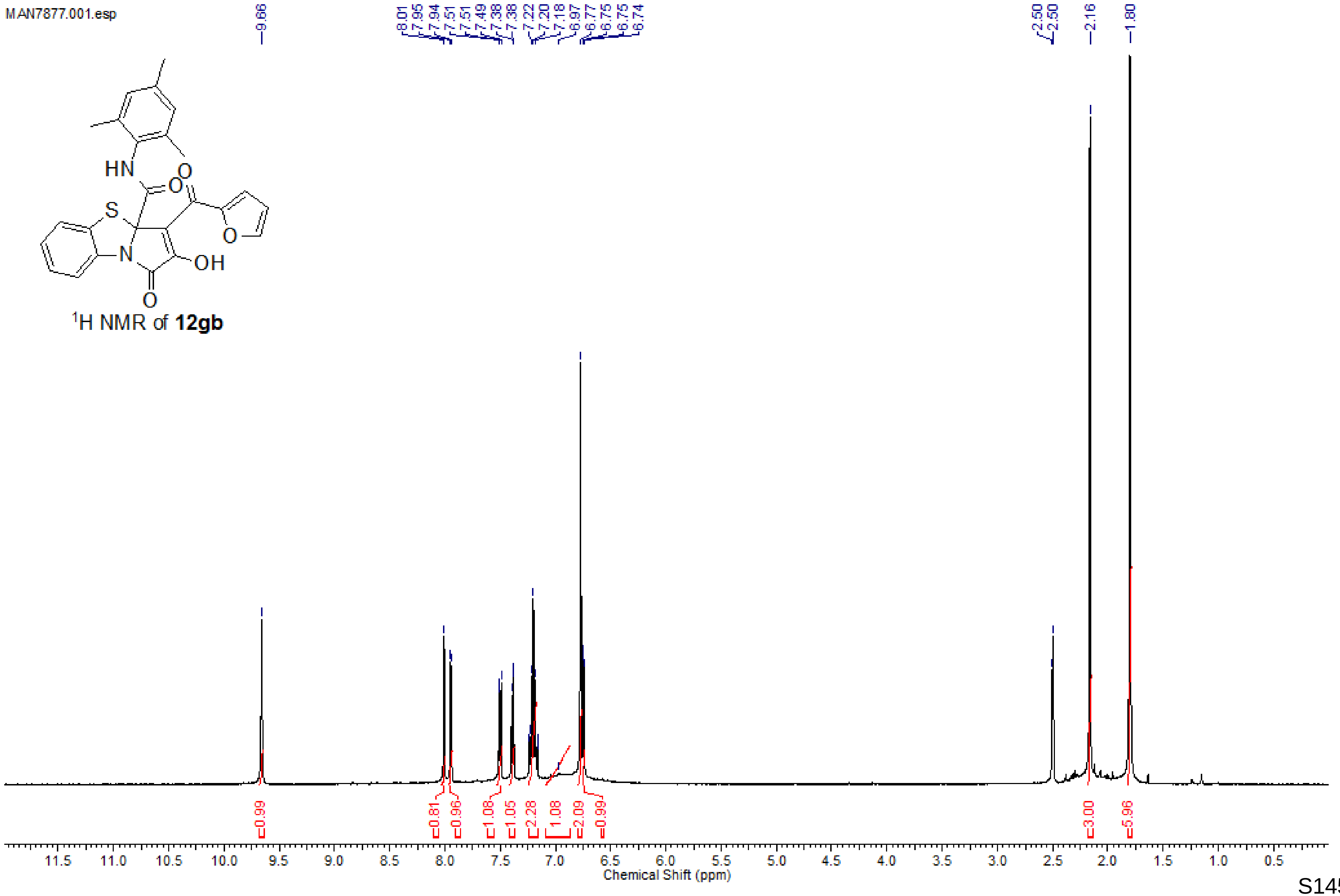

^{1}H NMR of **12gb**

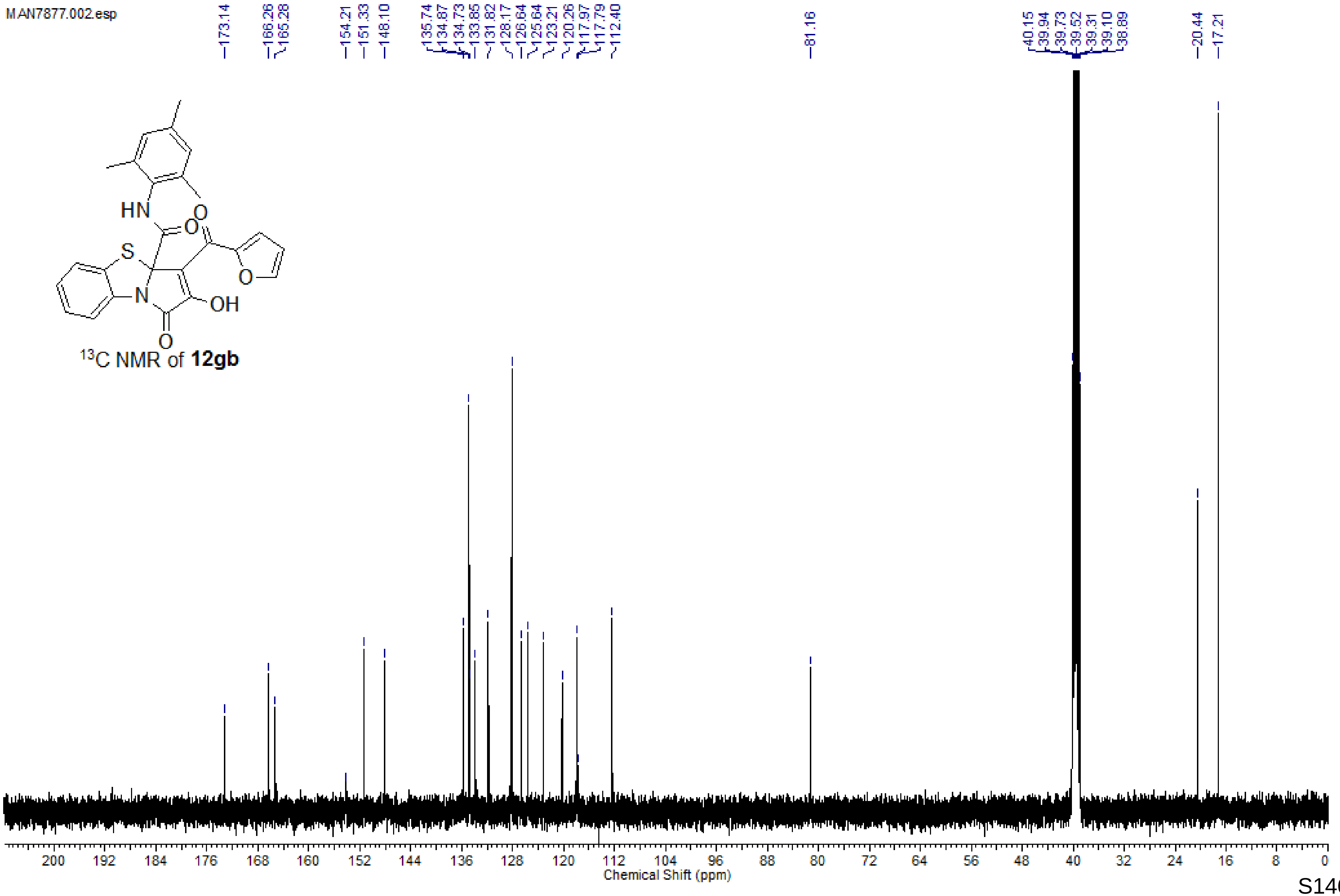
MAN7877.002.esp
173.14
166.26
165.28
154.21
151.33
148.10
135.74
134.87
134.73
133.85
131.82
128.17
126.64
125.64
123.21
120.26
117.97
117.79
112.40
81.16
40.15
39.94
39.73
39.52
39.31
39.10
38.89
20.44
17.21
HN
O
S
N
O
OH
O
O
^{13}C NMR of **12gb**
Chemical Shift (ppm)

MAN7877.003.esp

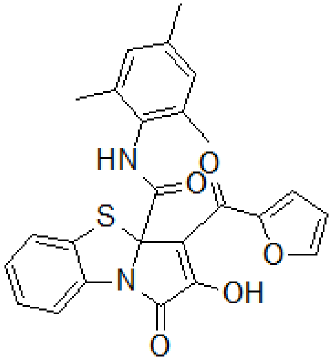

DEPT 135 of **12gb**

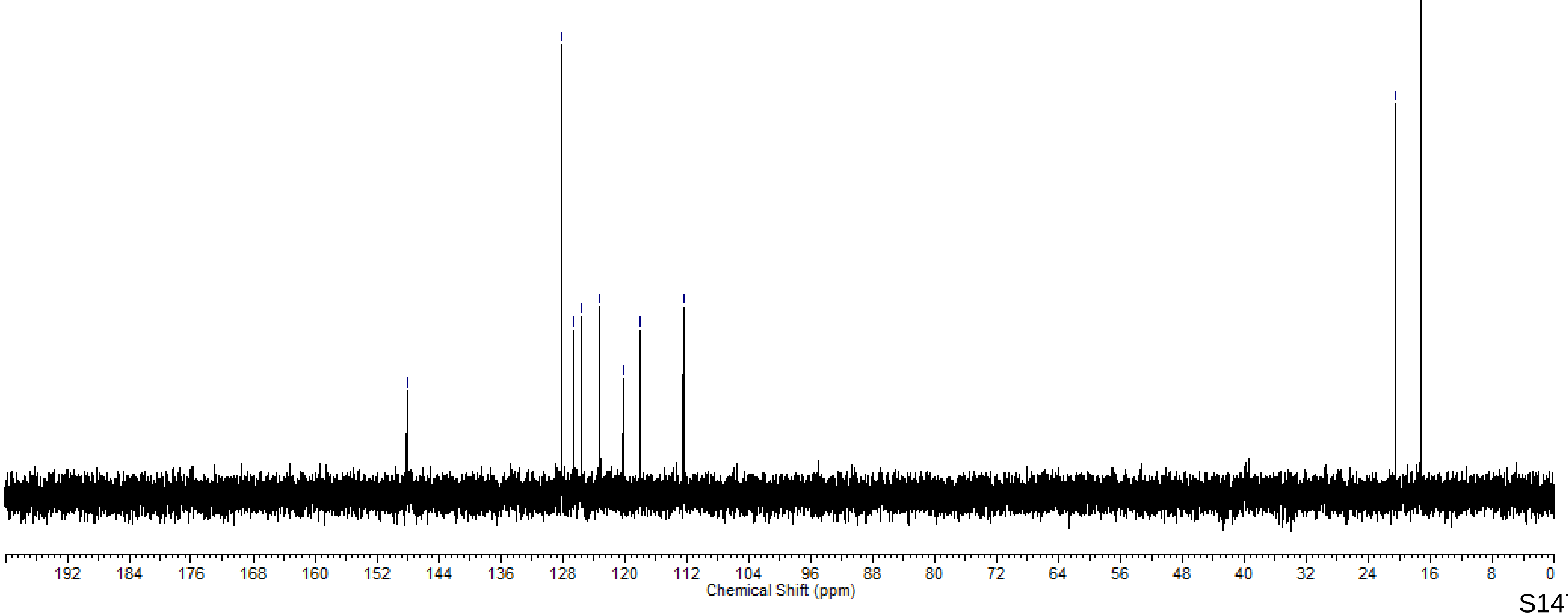

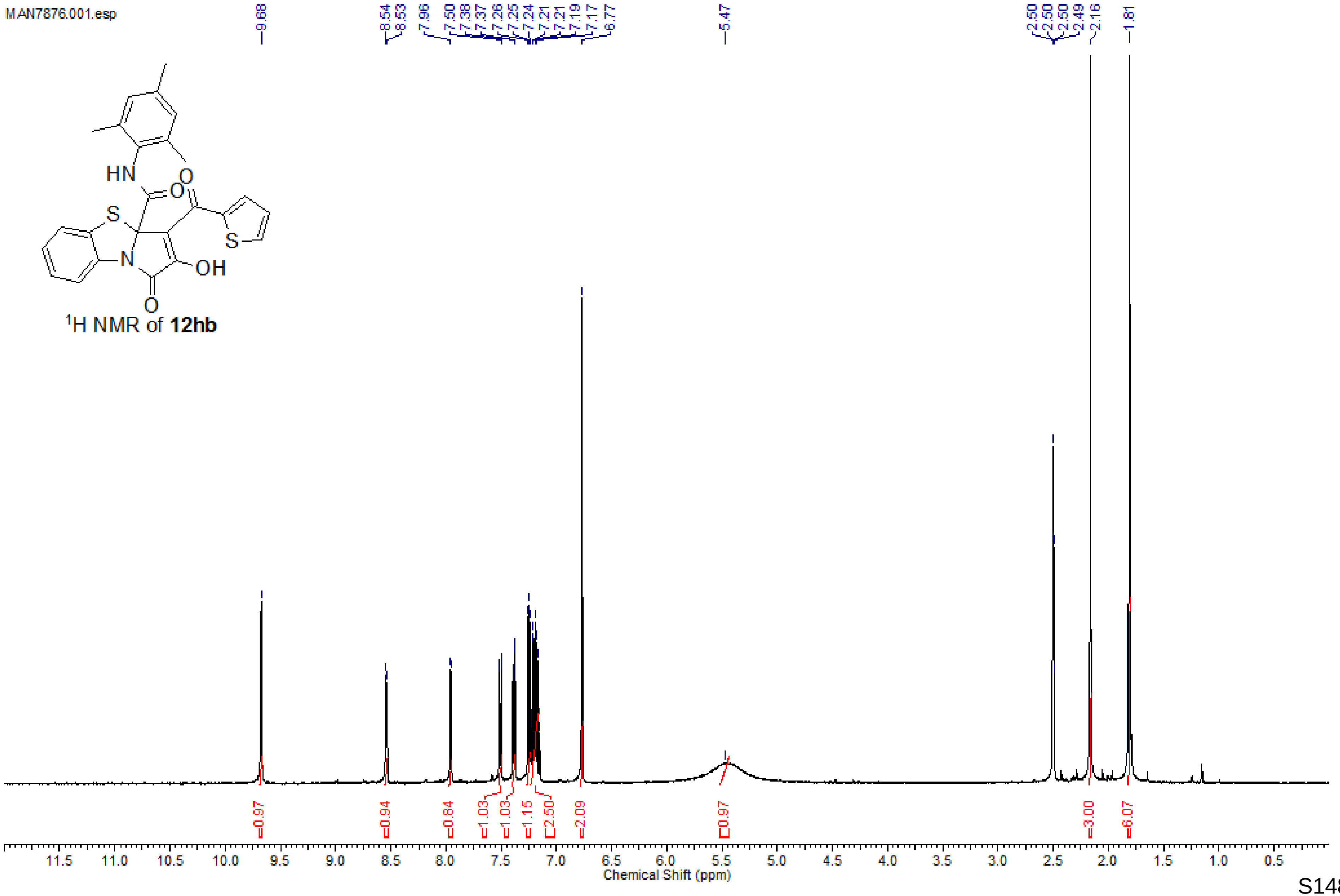

^{1}H NMR of **12hb**

MAN7876.002.esp

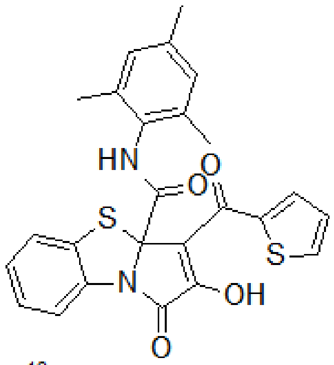

^{13}C NMR of **12hb**

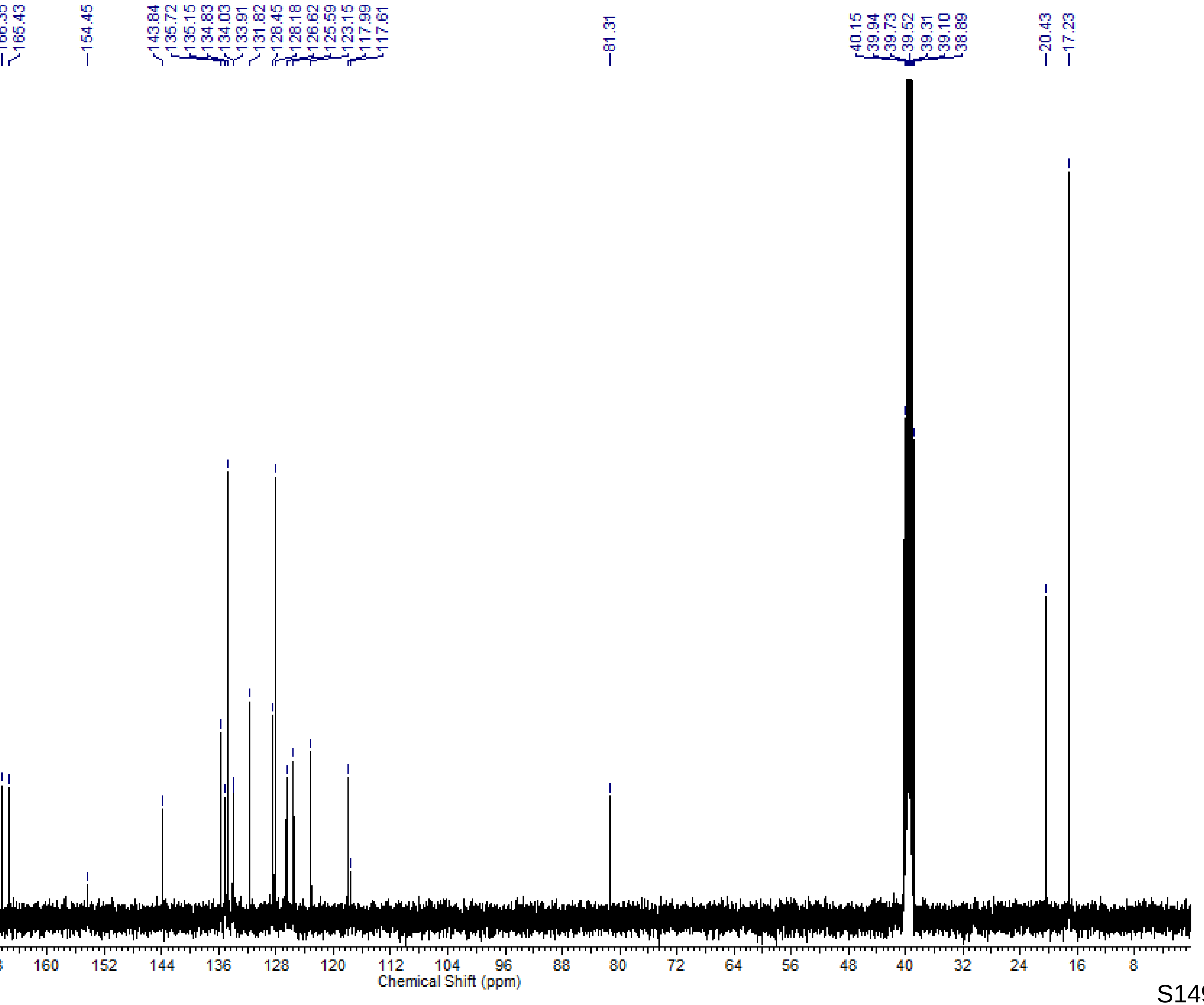

MAN7876.003.esp

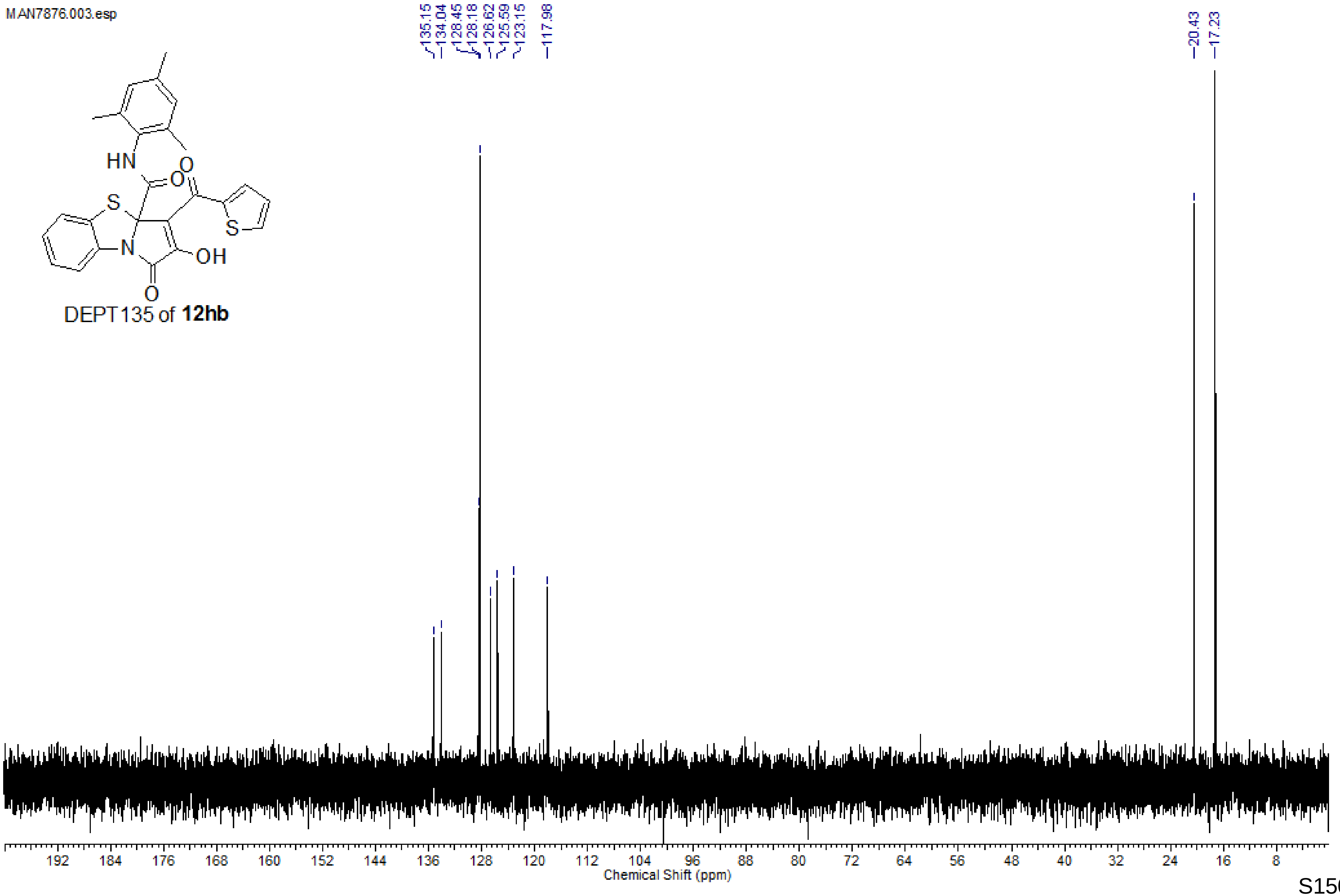

DEPT135 of **12hb**

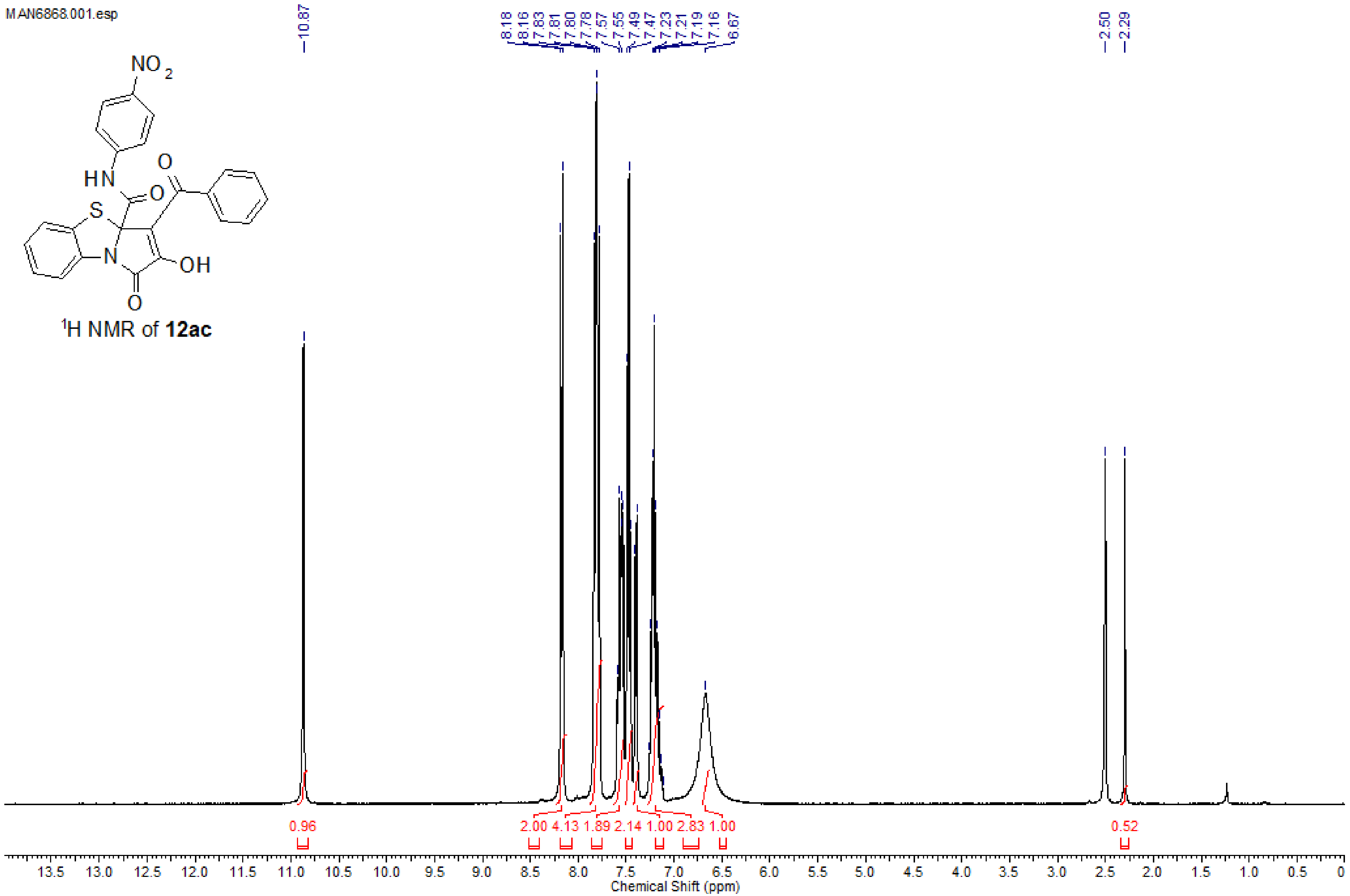

^{1}H NMR of **12ac**

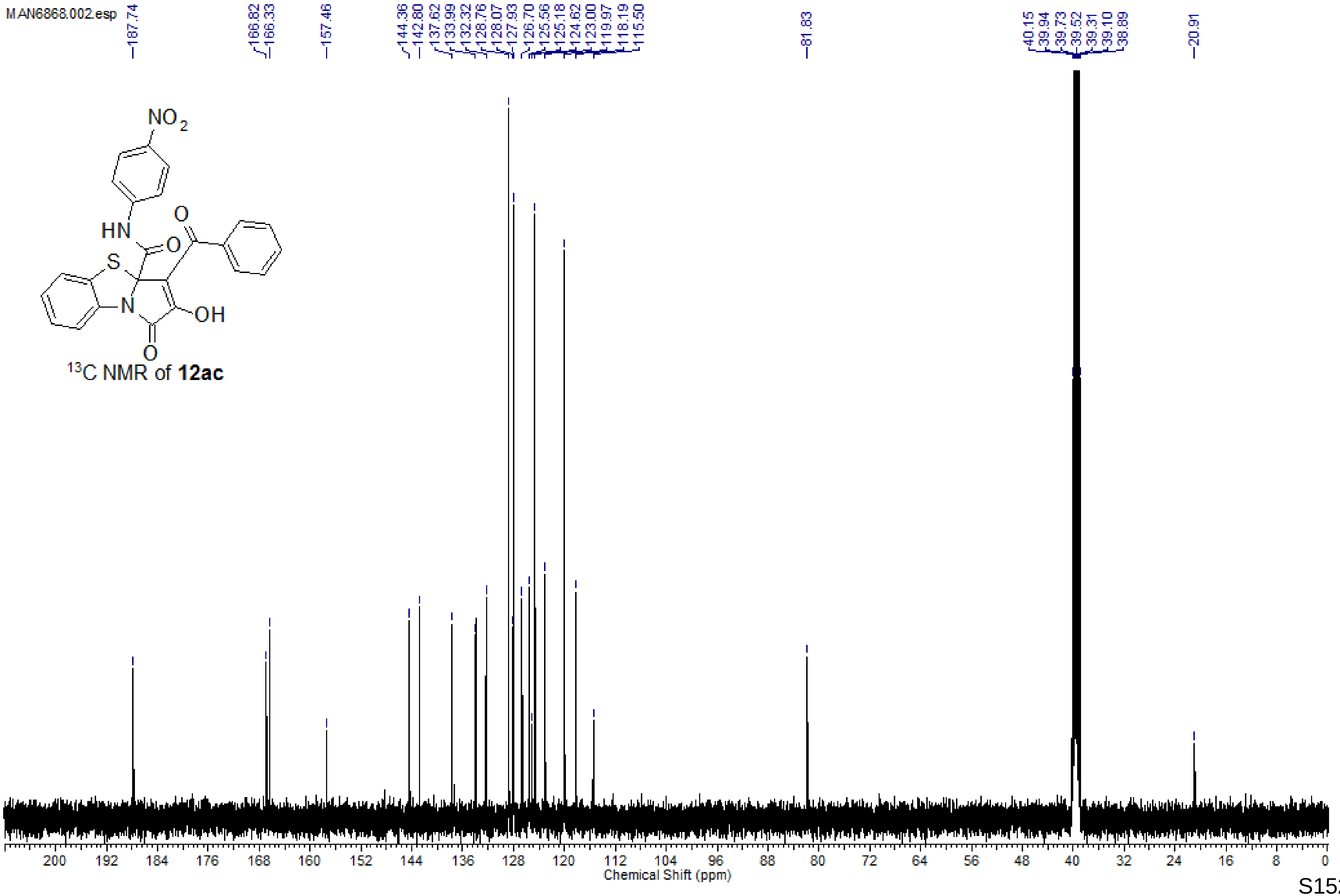
MAN6868.002.esp
187.74
166.82
166.33
157.46
144.36
142.80
137.62
133.99
132.32
128.76
128.07
127.93
126.70
125.56
125.18
124.62
123.00
119.97
118.19
115.50
81.83
40.15
39.94
39.73
39.52
39.31
39.10
38.89
20.91
NO2
HN
O
O
S
N
OH
O
^{13}C NMR of **12ac**
Chemical Shift (ppm)

MAN6868.003.esp

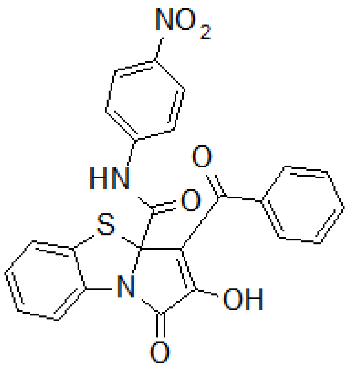

DEPT135 of **12ac**

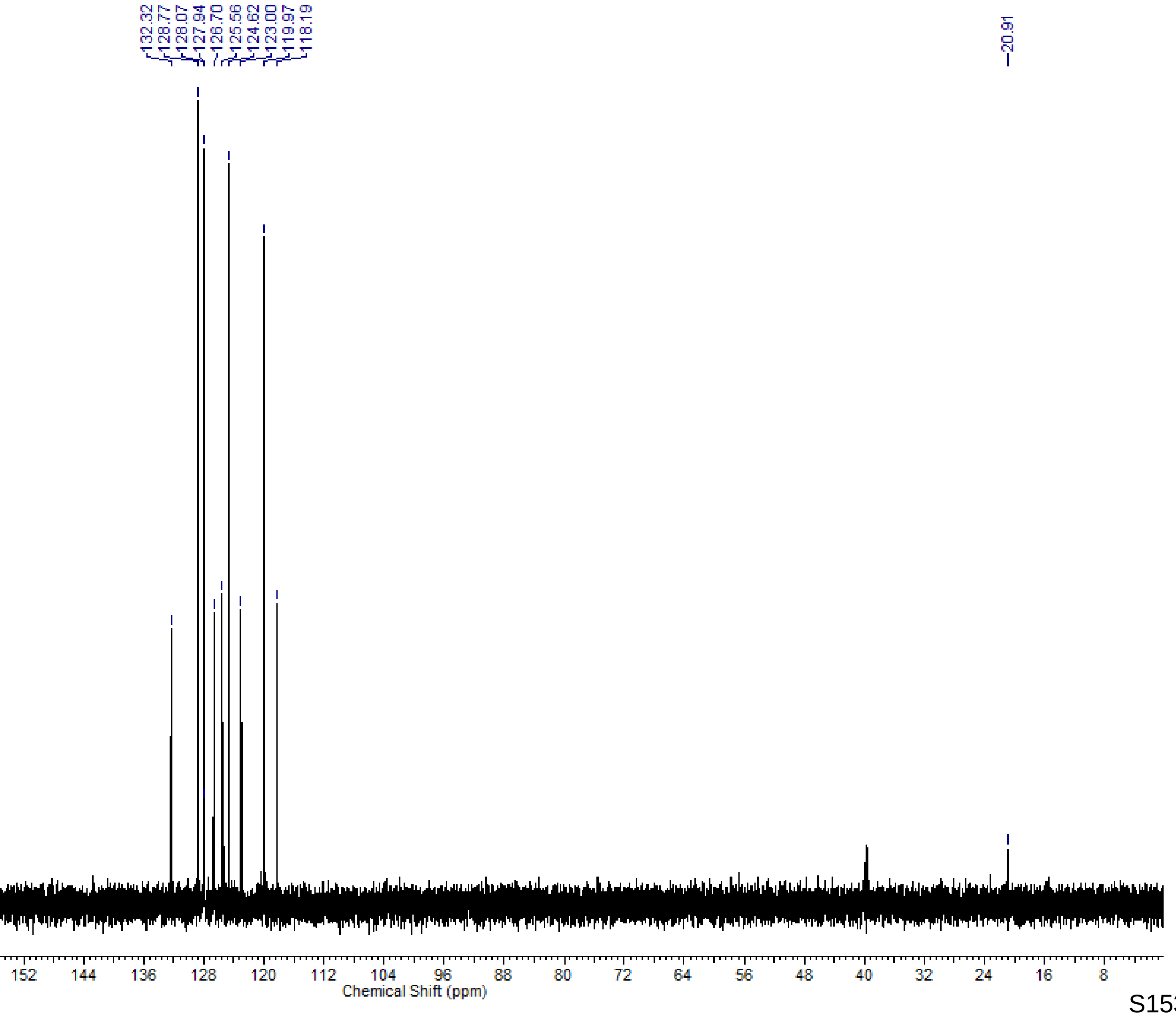

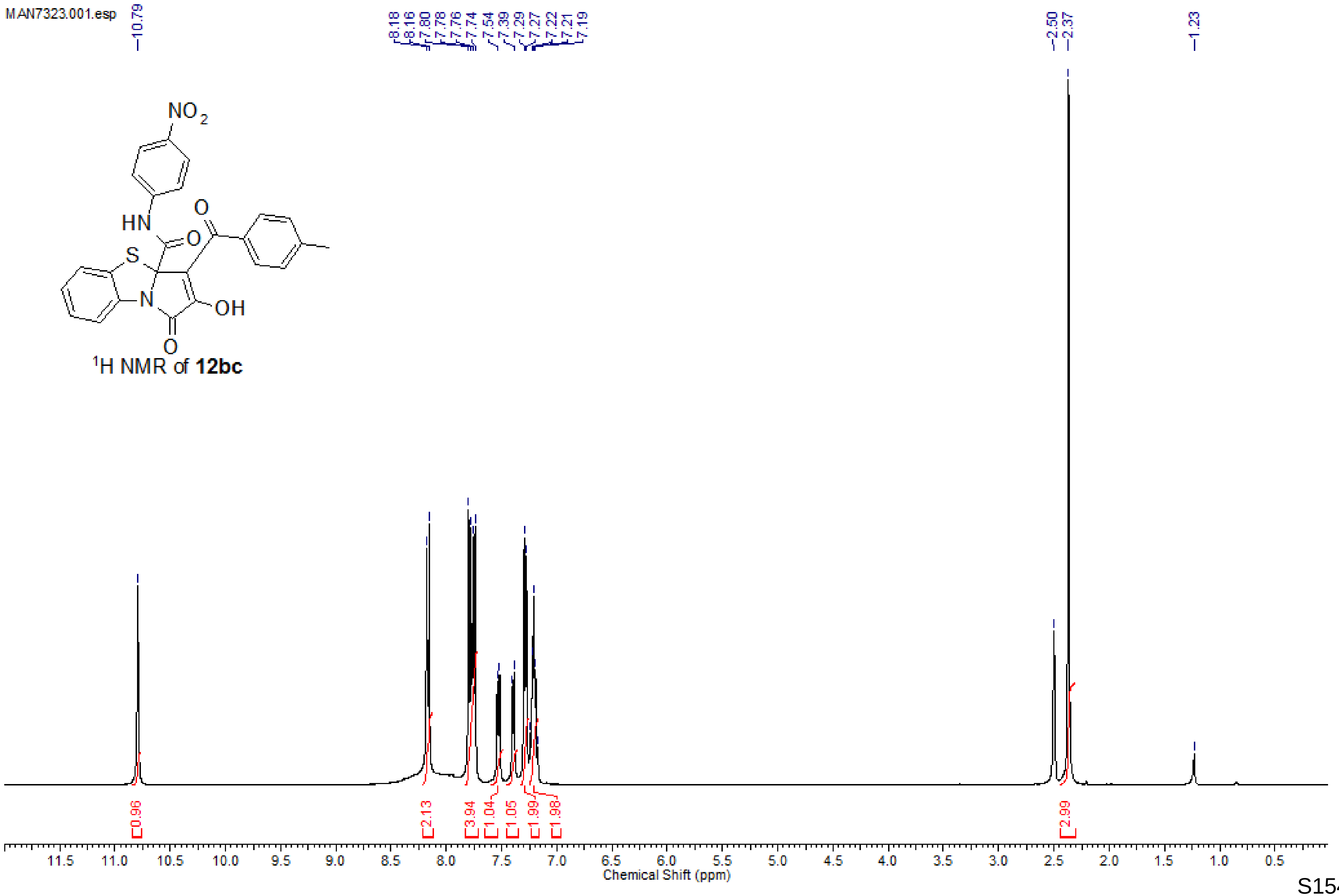

^{1}H NMR of **12bc**

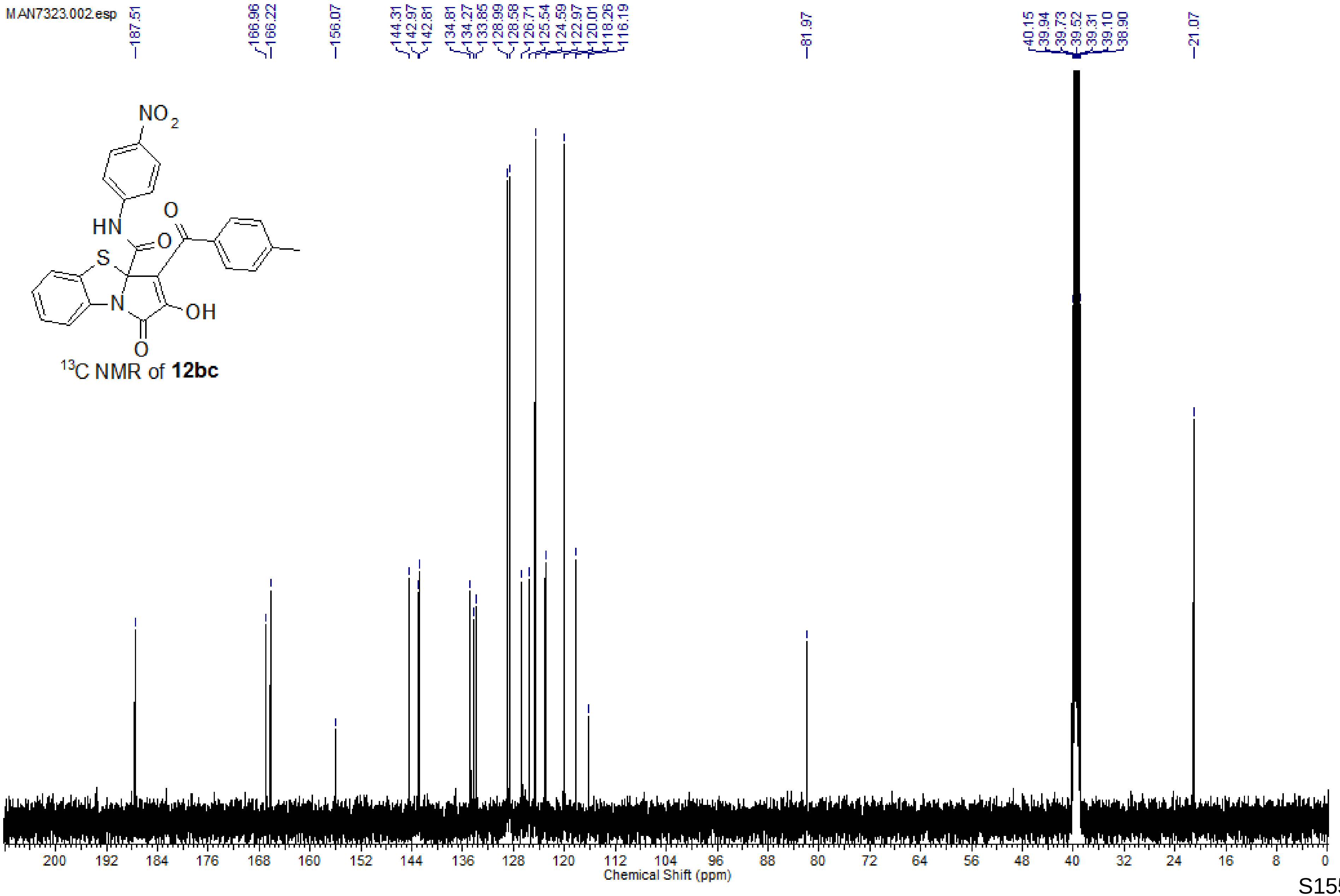

^{13}C NMR of **12bc**

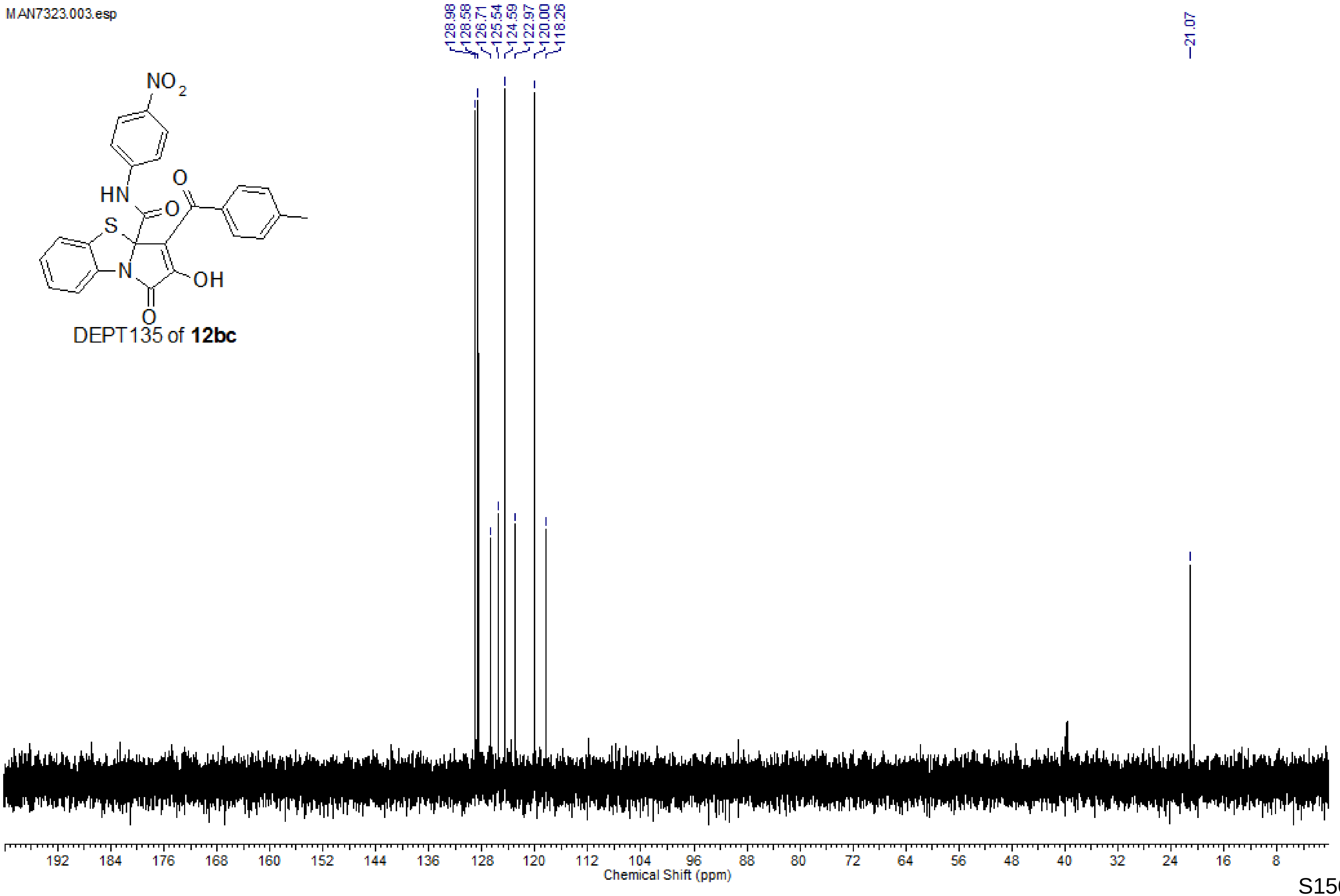

DEPT135 of **12bc**

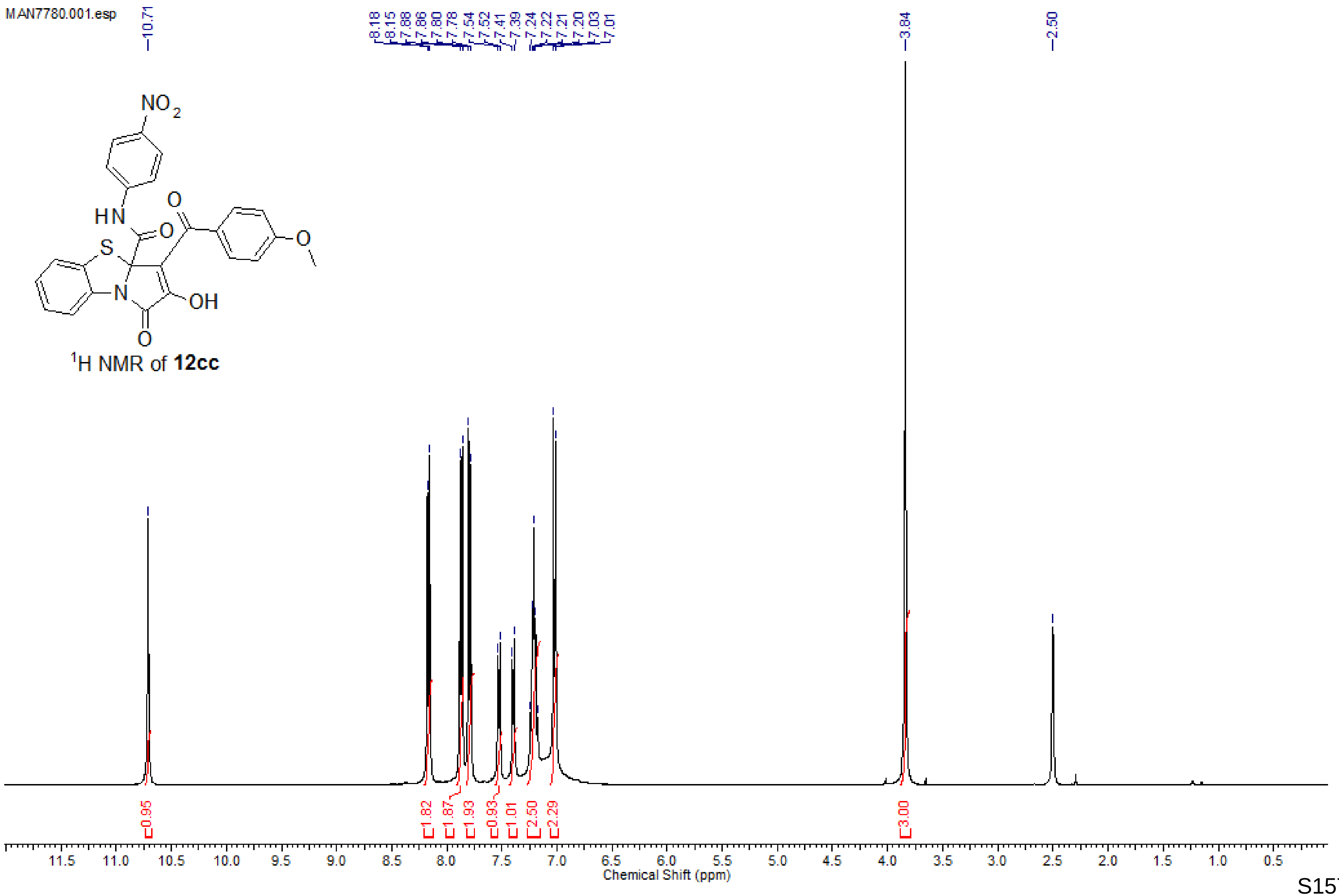
MAN7780.001.esp
10.71
8.18
8.15
7.88
7.86
7.80
7.78
7.54
7.52
7.41
7.39
7.24
7.22
7.21
7.20
7.03
7.01
3.84
2.50
NO2
HN
O
O
S
N
OH
O
O
^{1}H NMR of **12cc**
0.95
1.82
1.87
1.93
0.93
1.01
2.50
2.29
3.00
Chemical Shift (ppm)

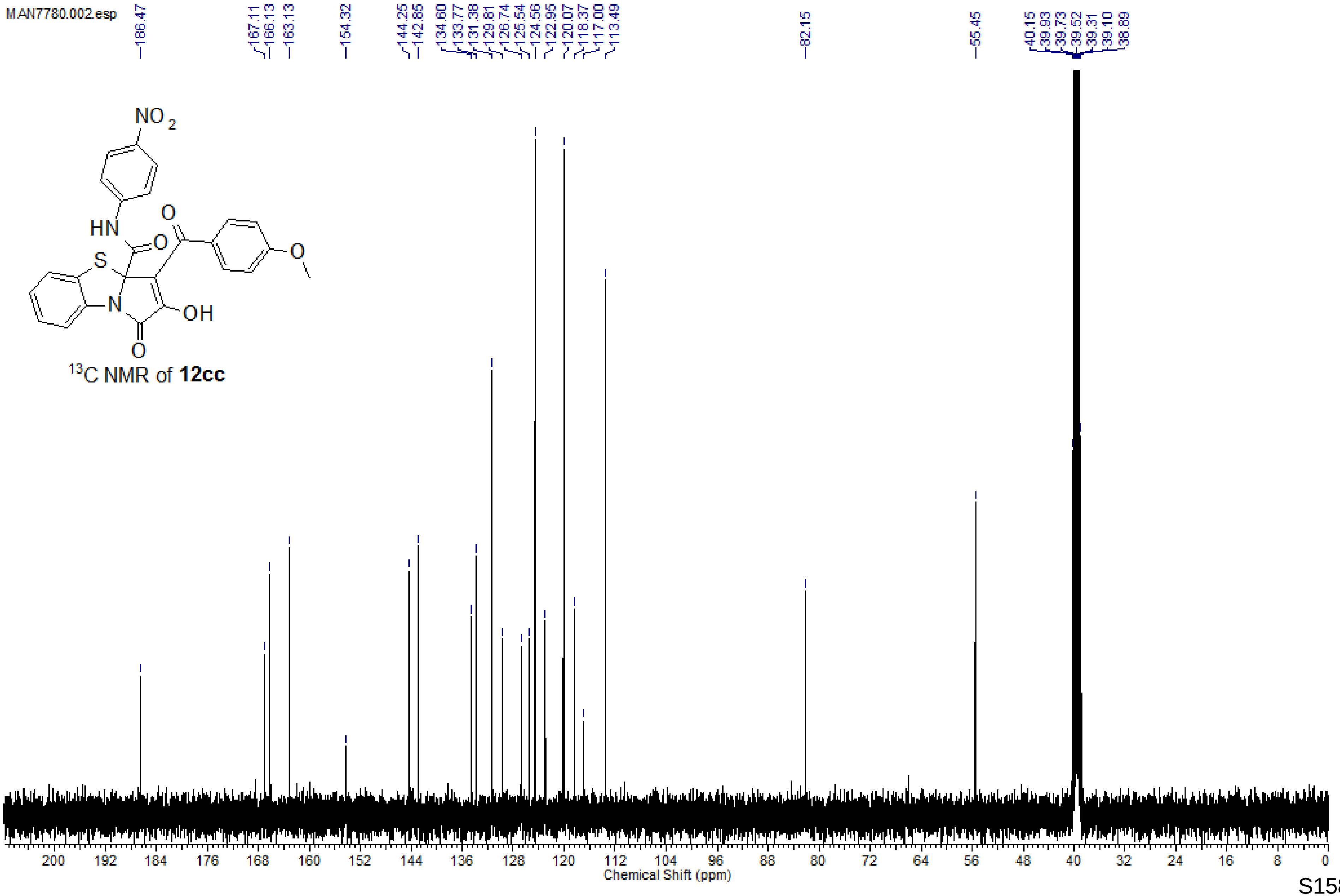

^{13}C NMR of **12cc**

MAN7780.003.esp

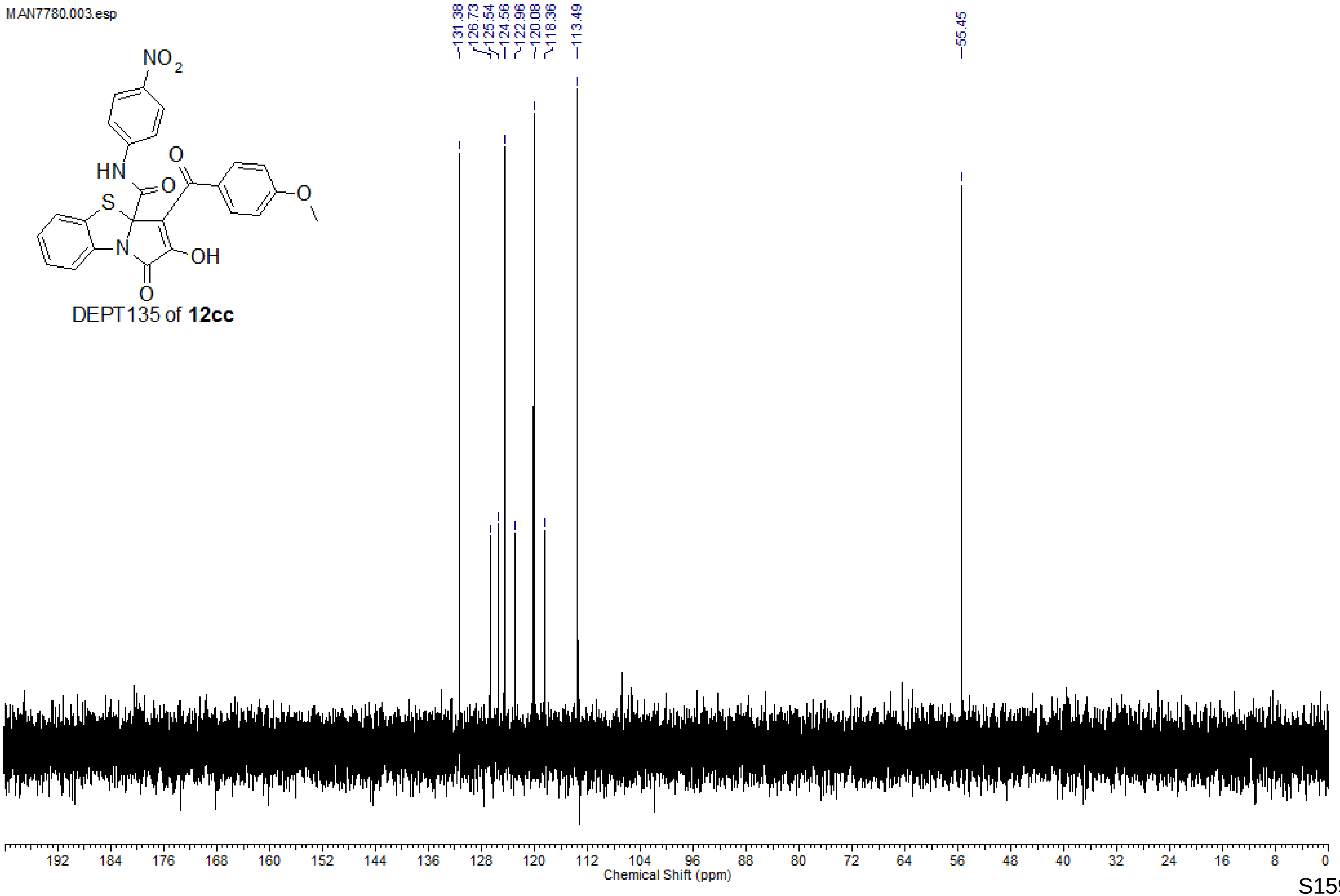

DEPT135 of **12cc**

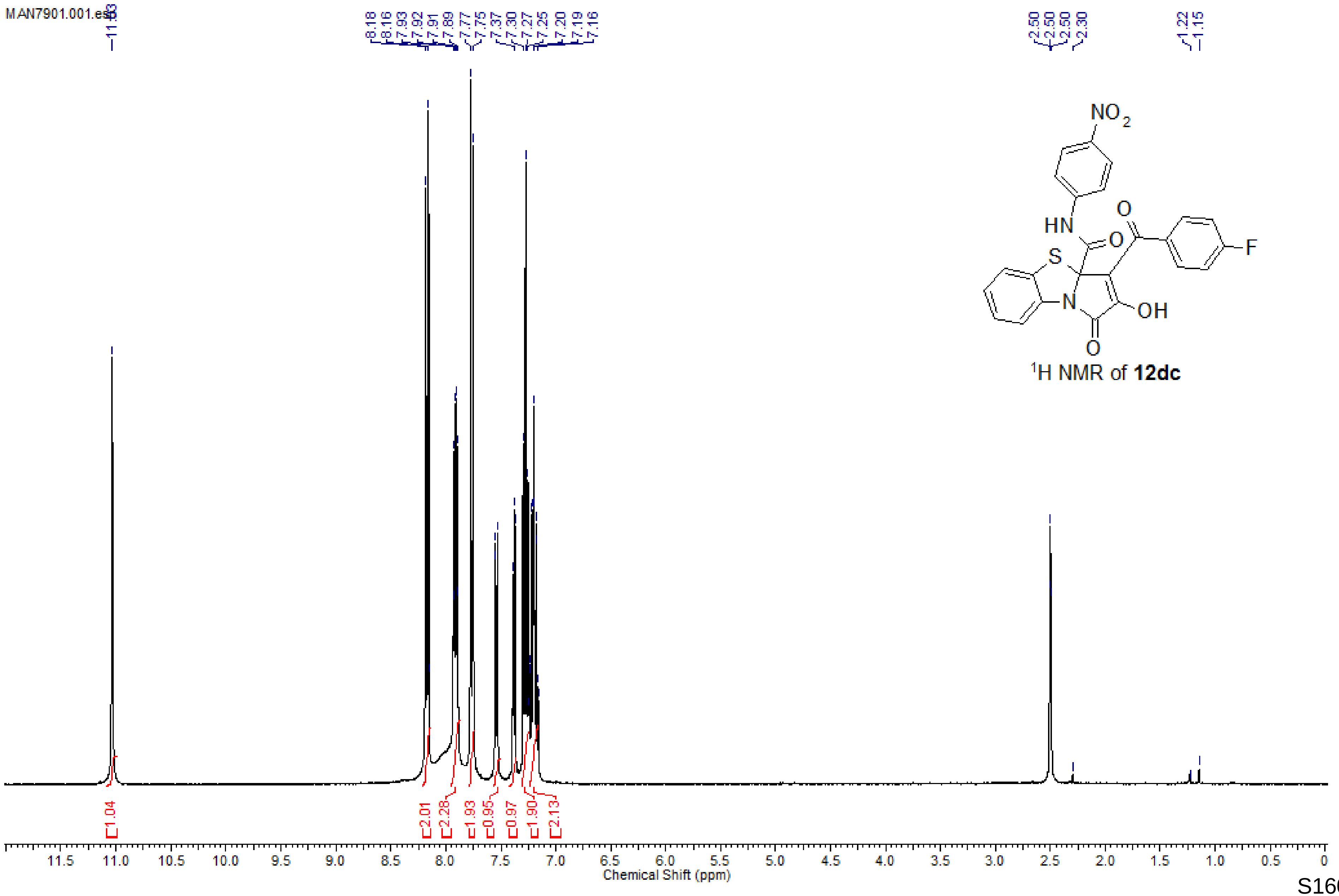

^{1}H NMR of **12dc**

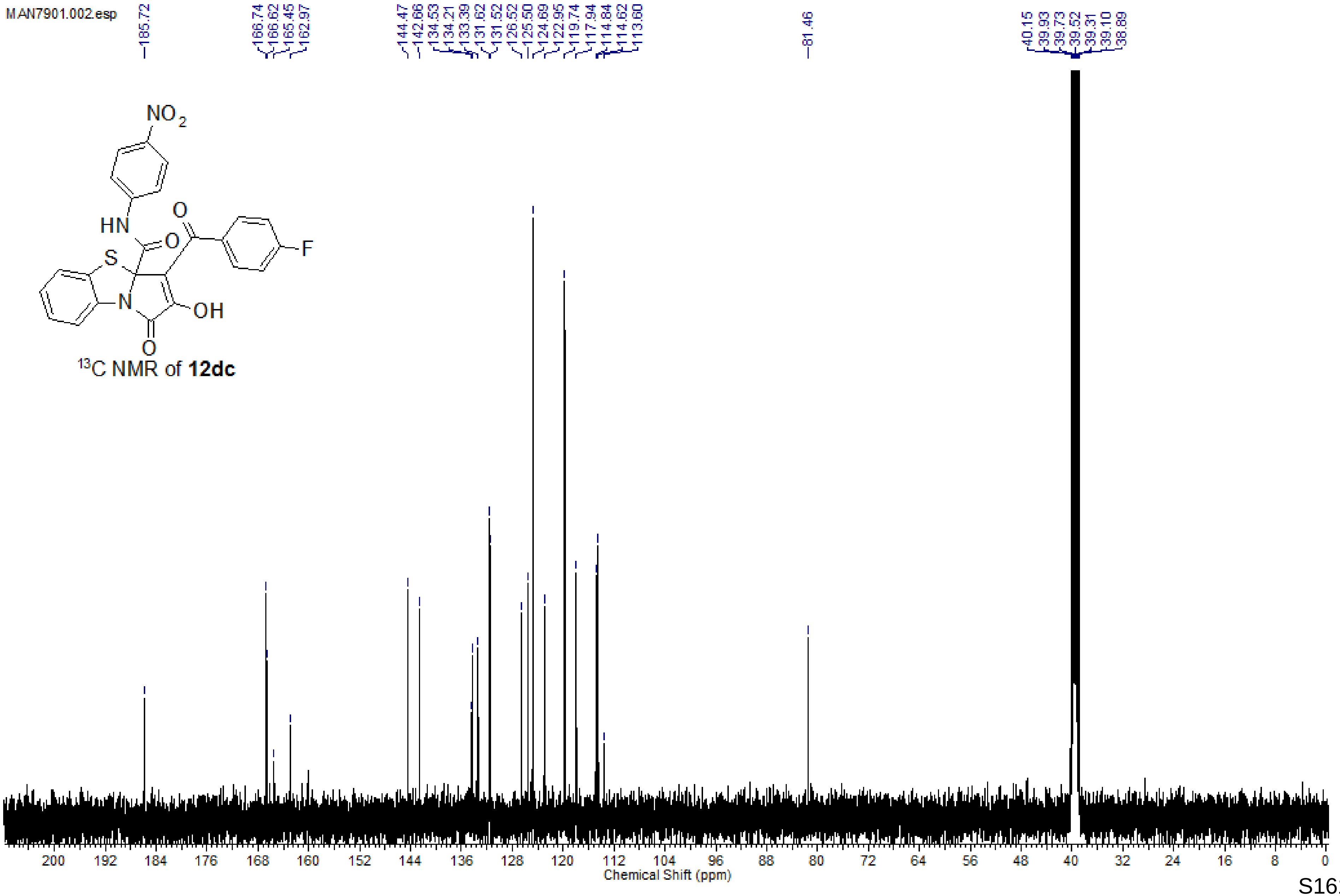

^{13}C NMR of **12dc**

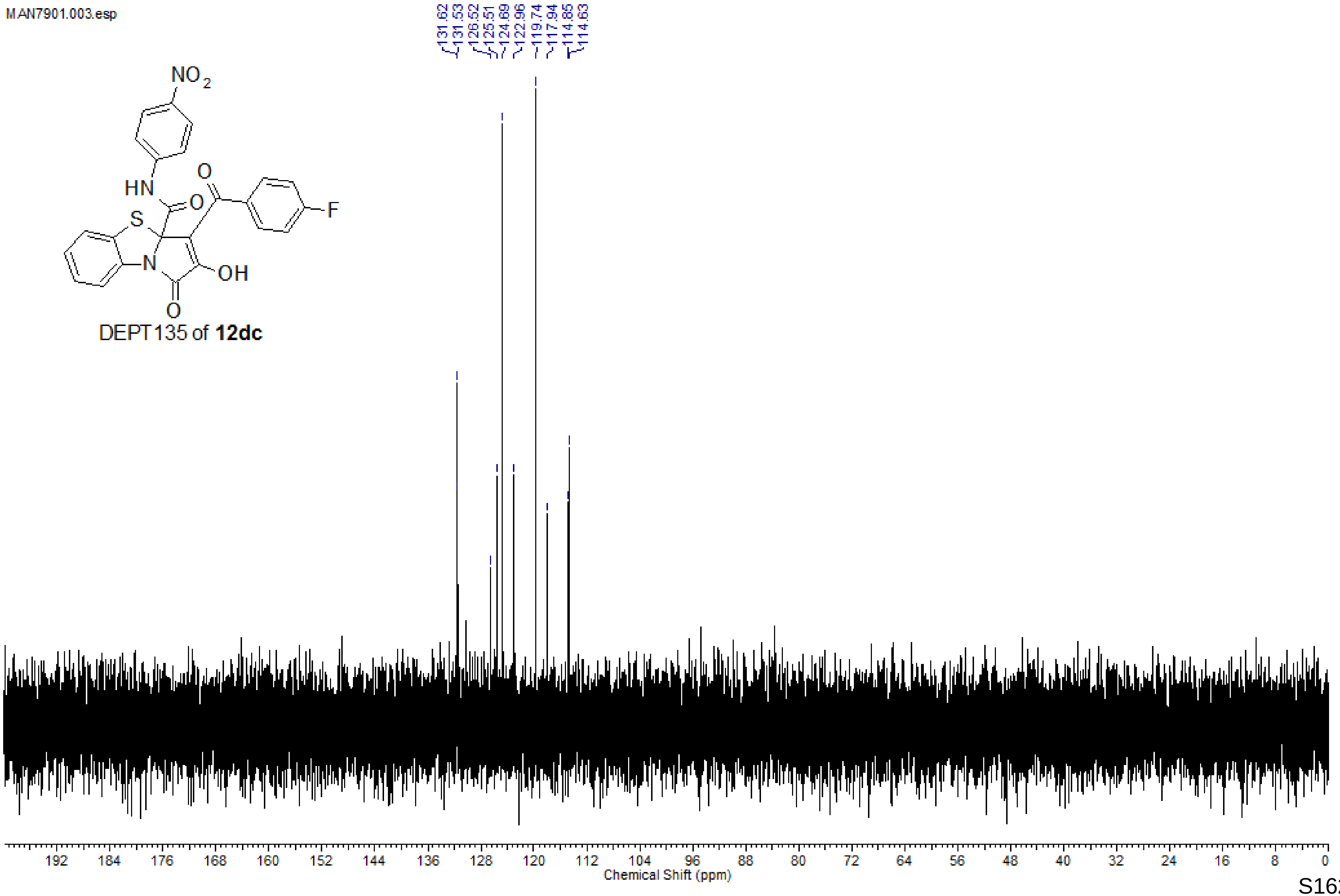

DEPT135 of **12dc**

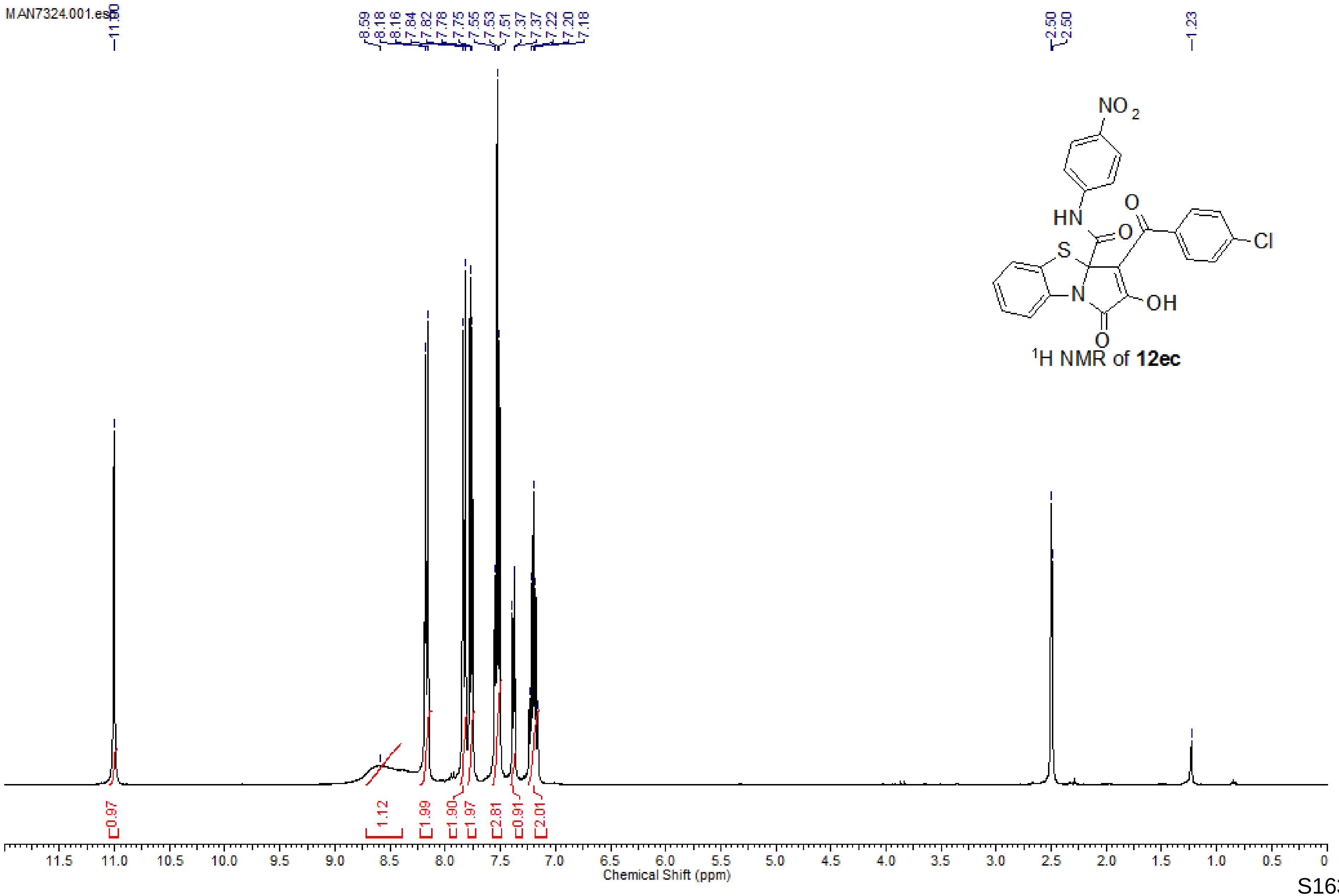

^{1}H NMR of **12ec**

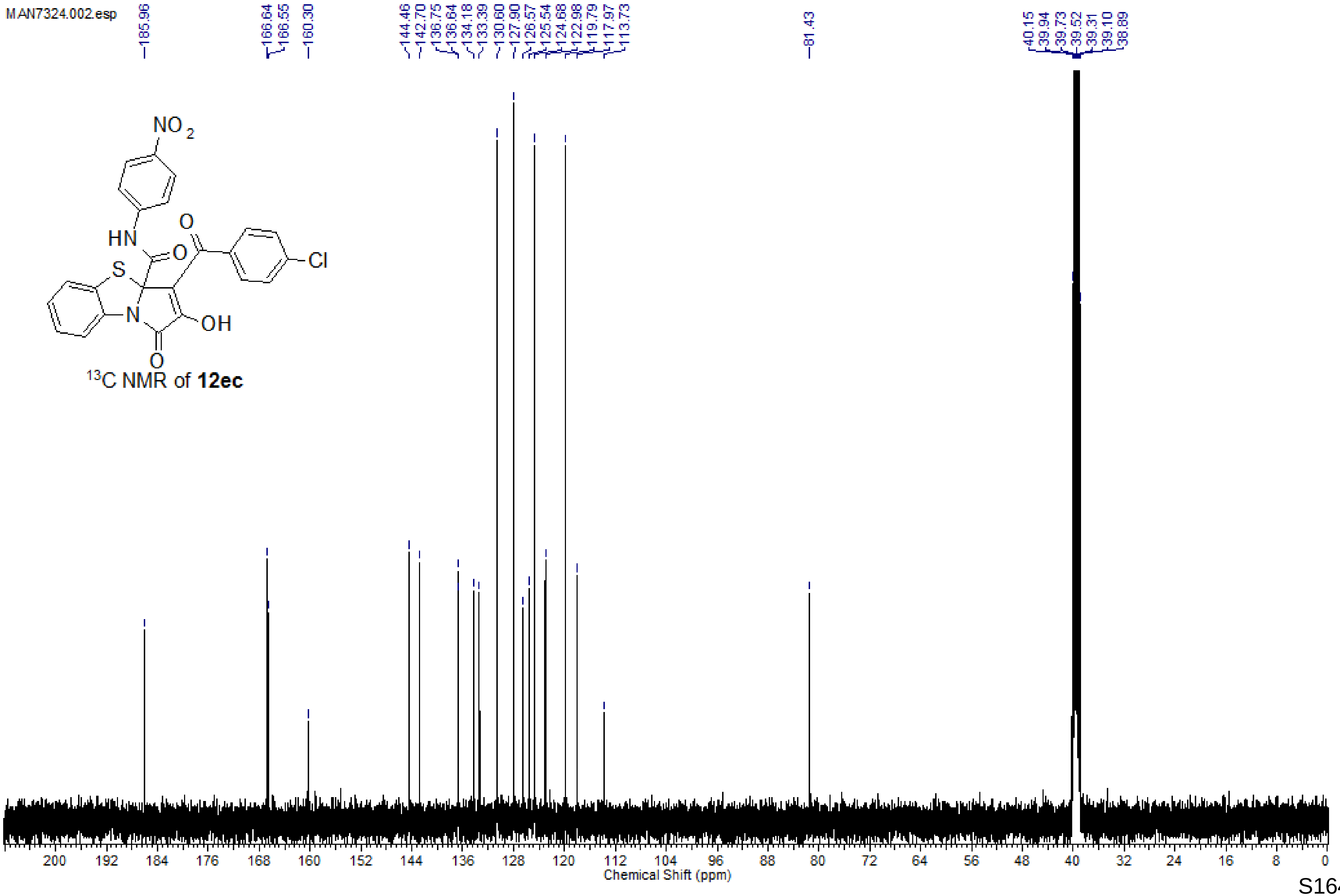

^{13}C NMR of **12ec**

MAN7324.003.esp

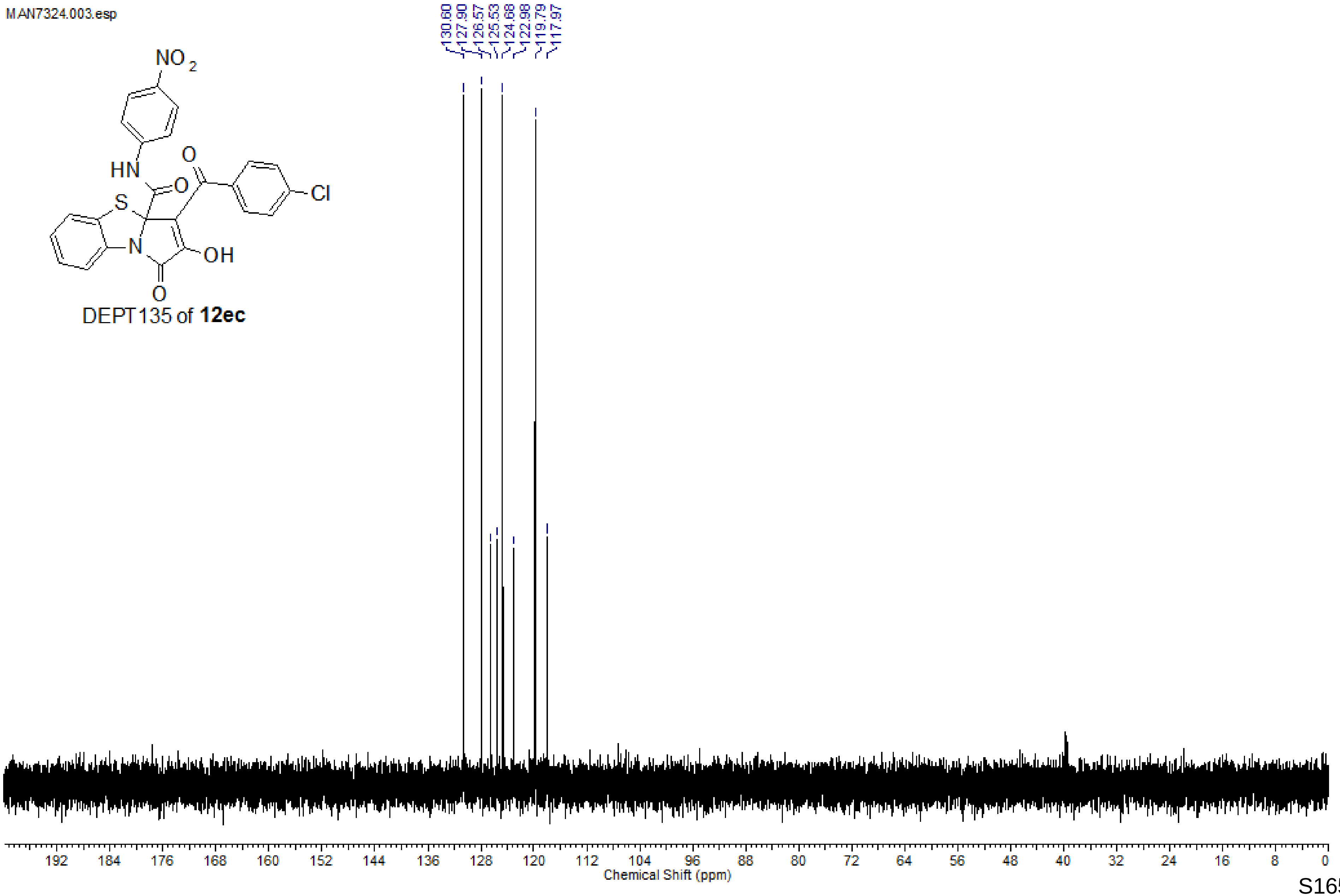

DEPT135 of **12ec**

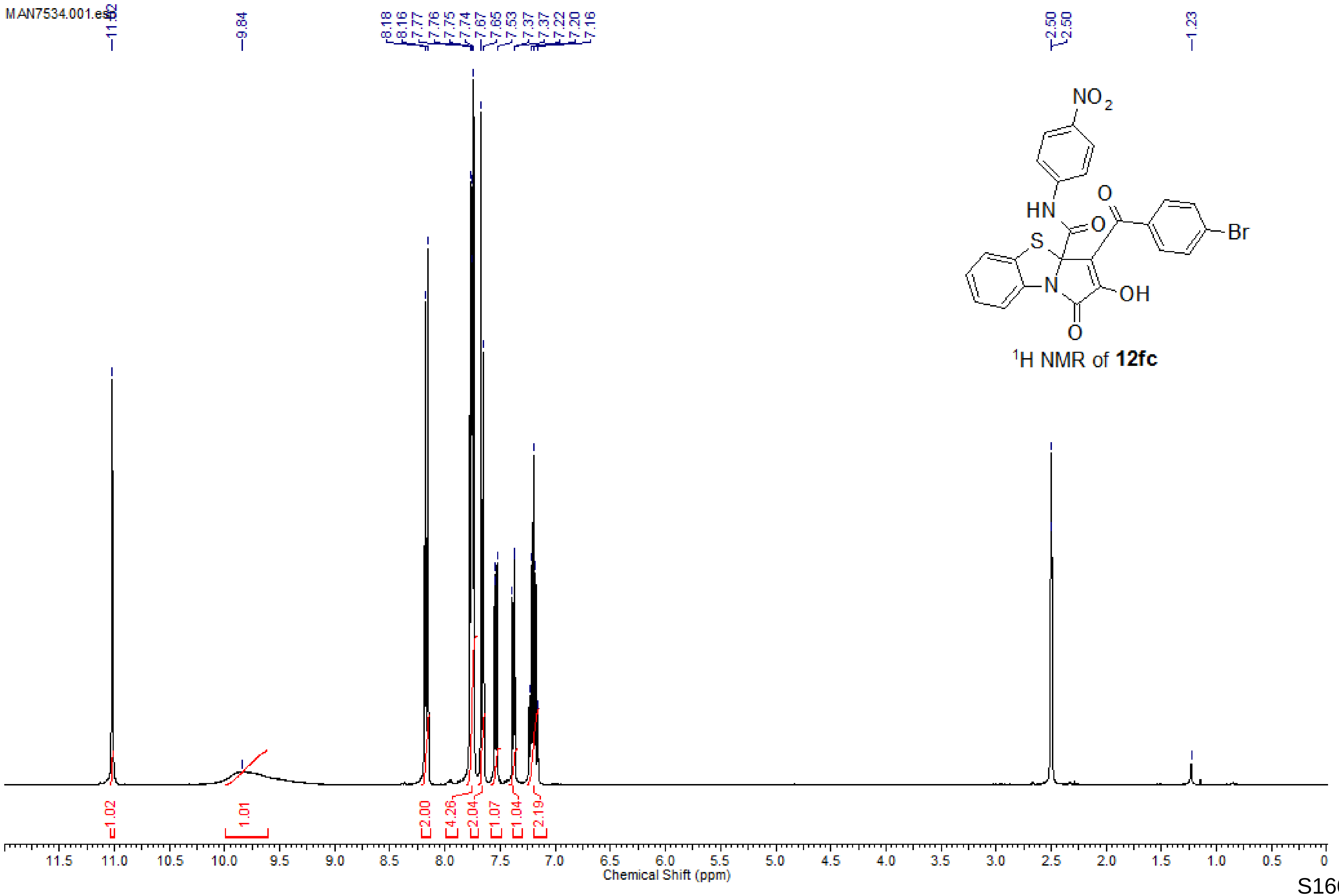

^{1}H NMR of **12fc**

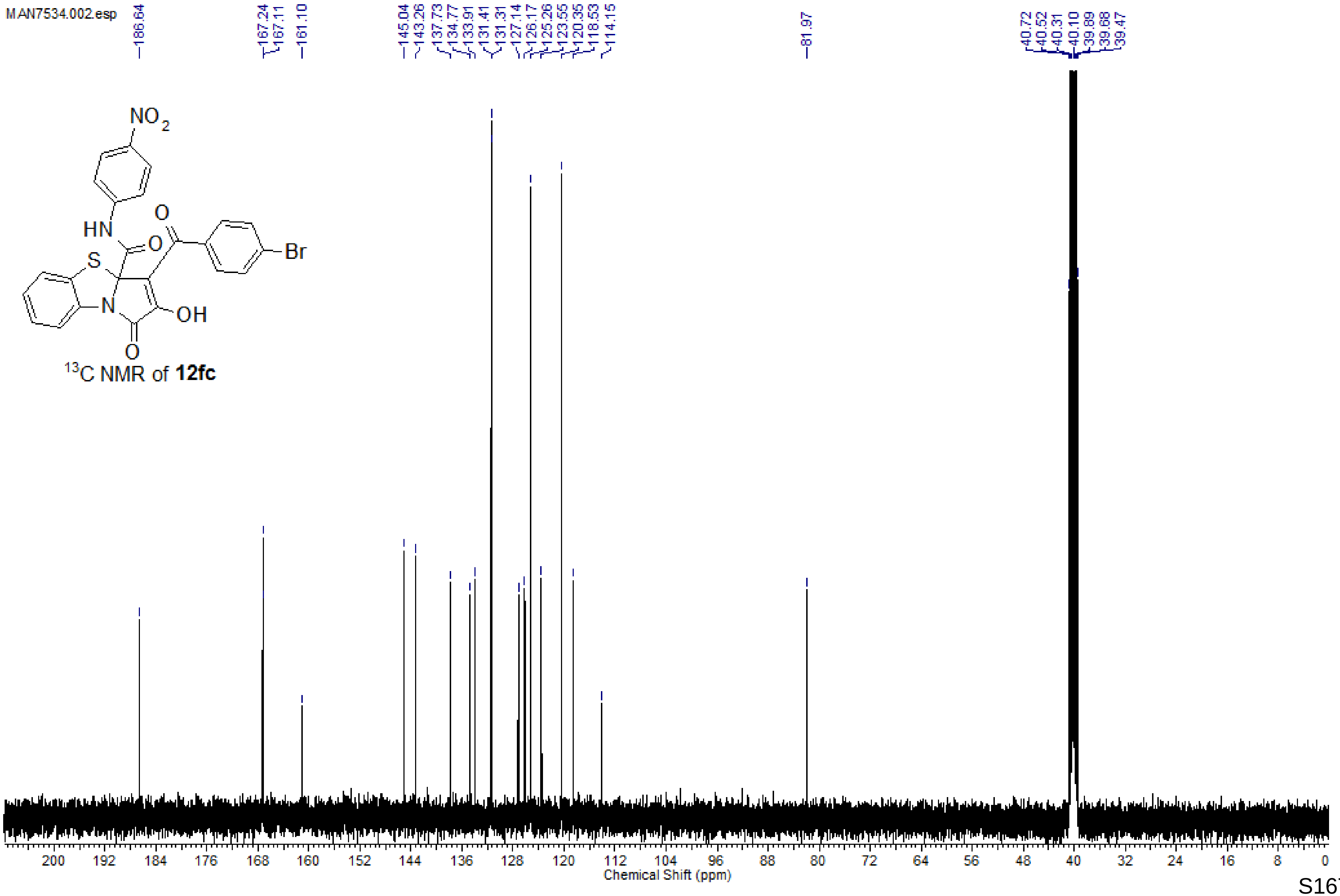

^{13}C NMR of **12fc**

MAN7534.003.esp

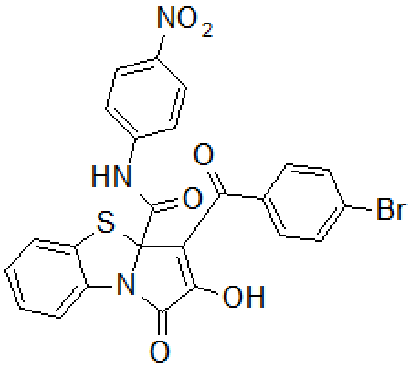

DEPT135 of **12fc**

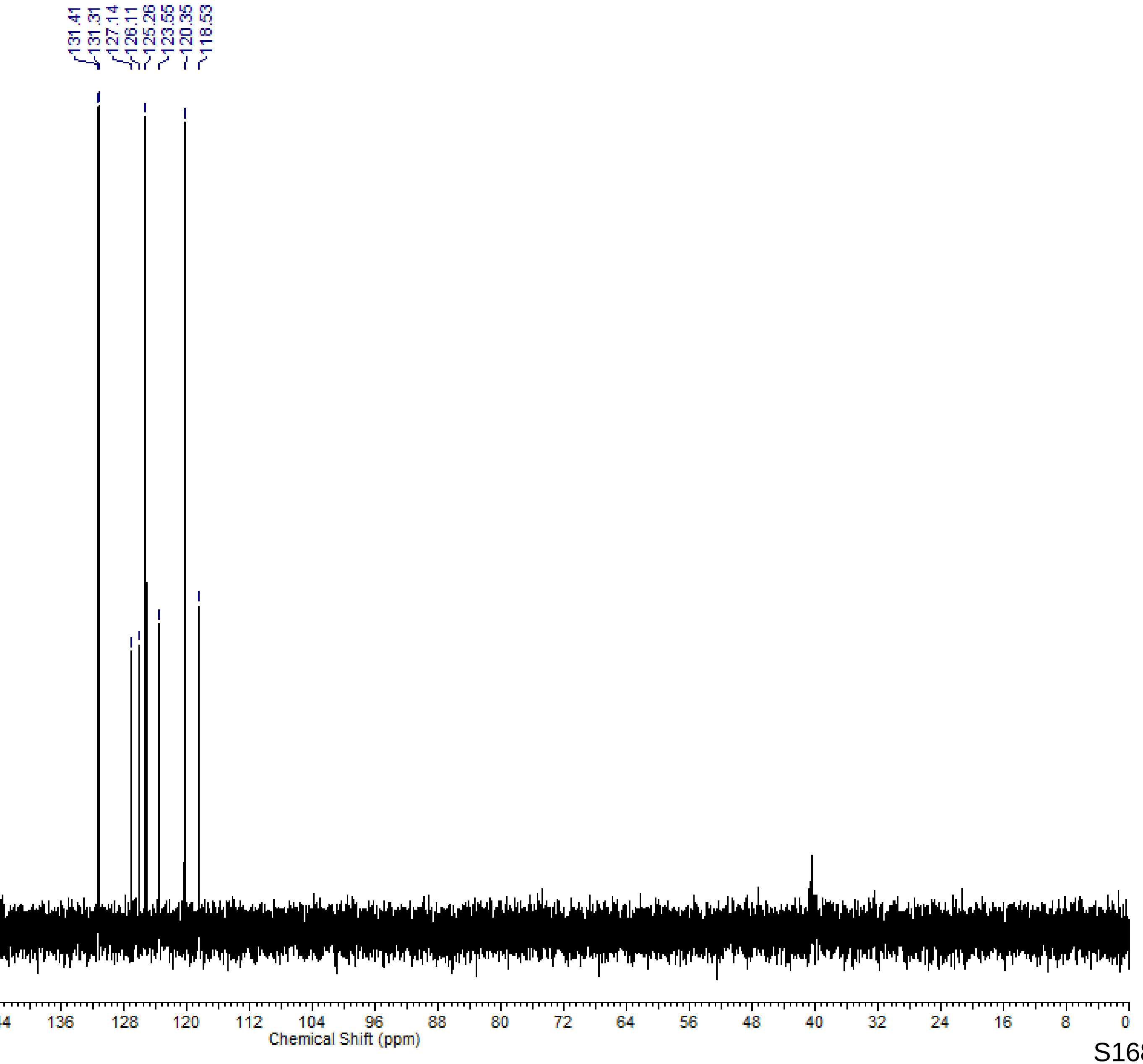

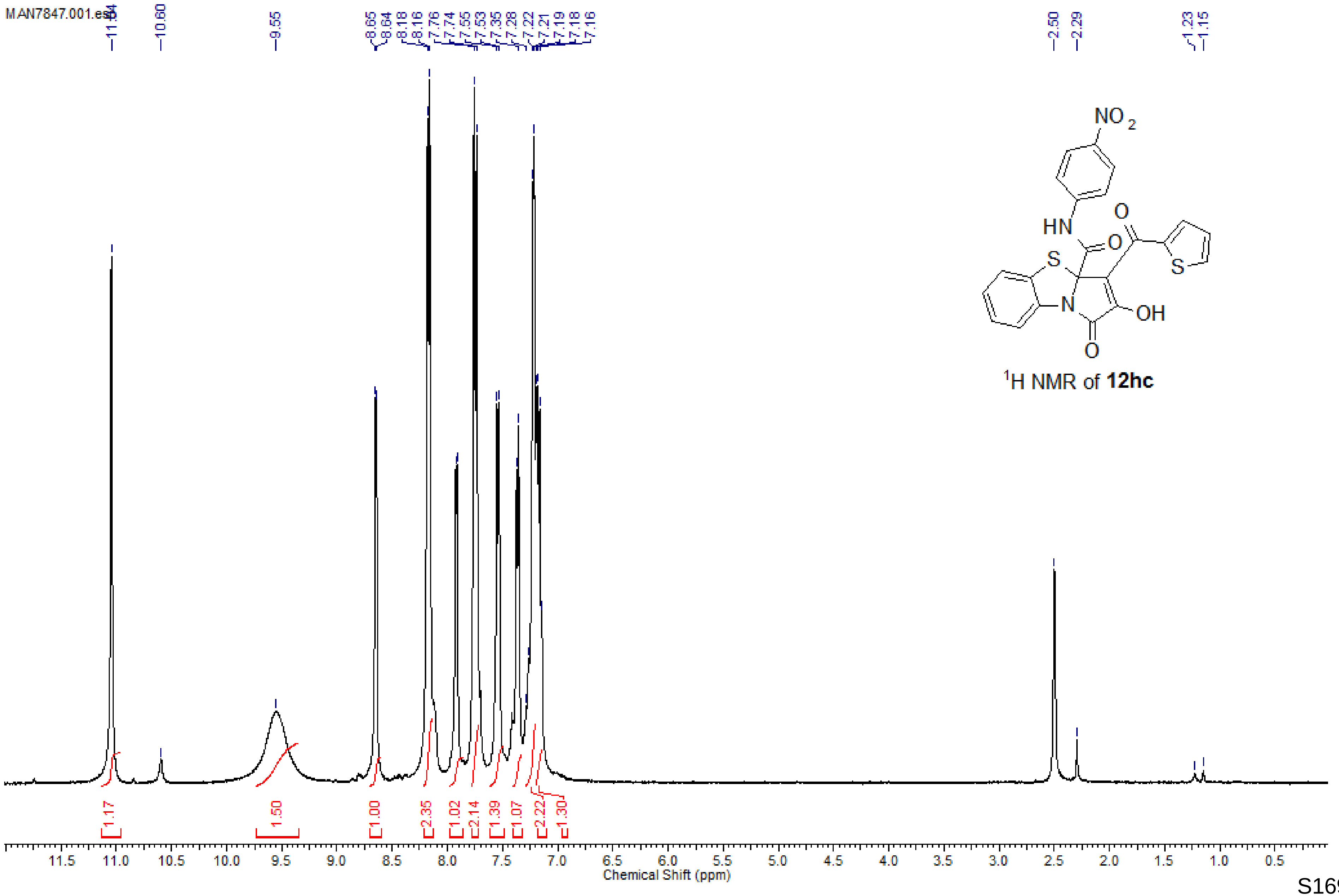

1H NMR of **12hc**

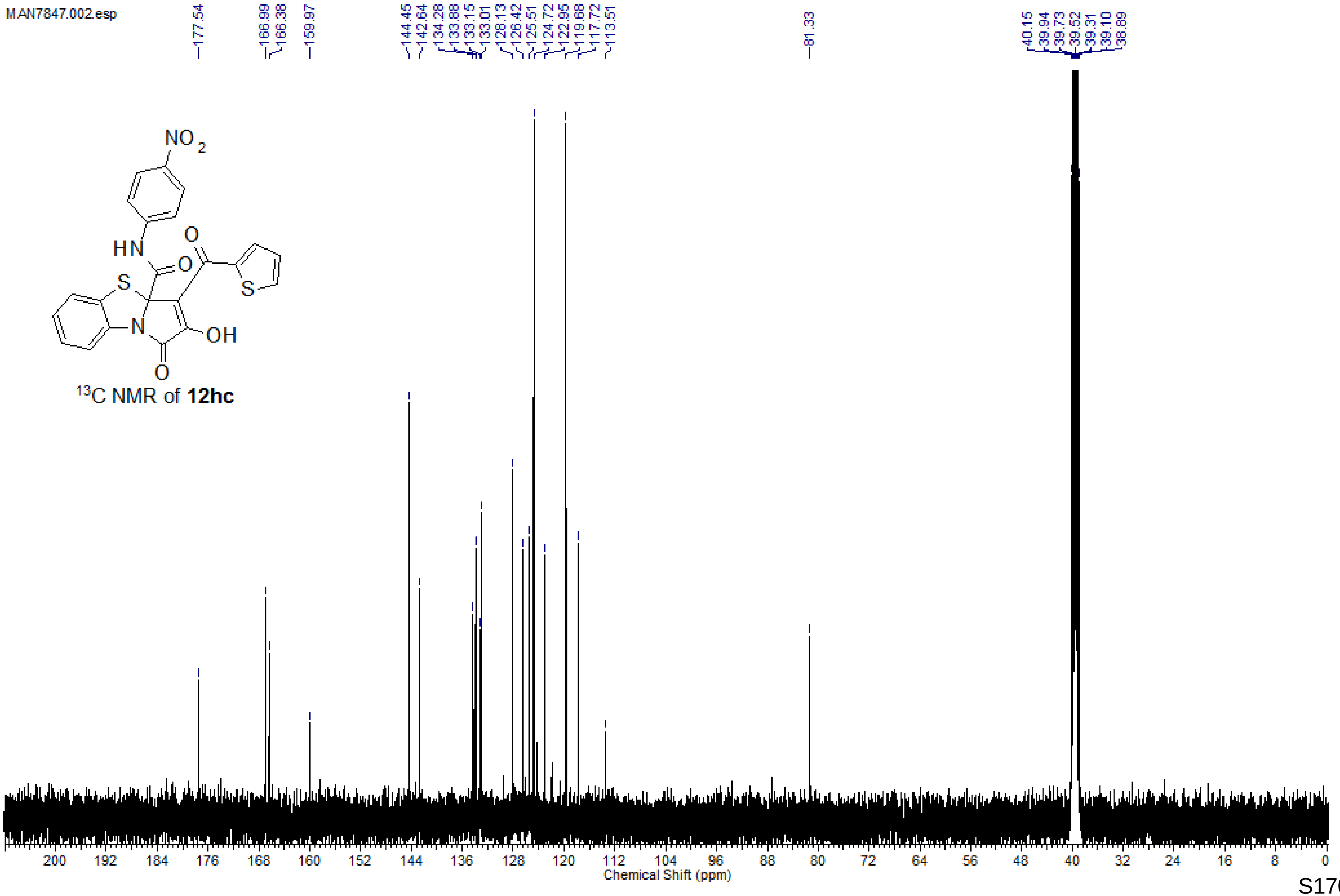

^{13}C NMR of **12hc**

MAN7847.003.esp

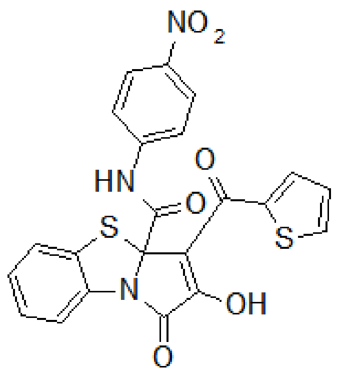

DEPT135 of **12hc**

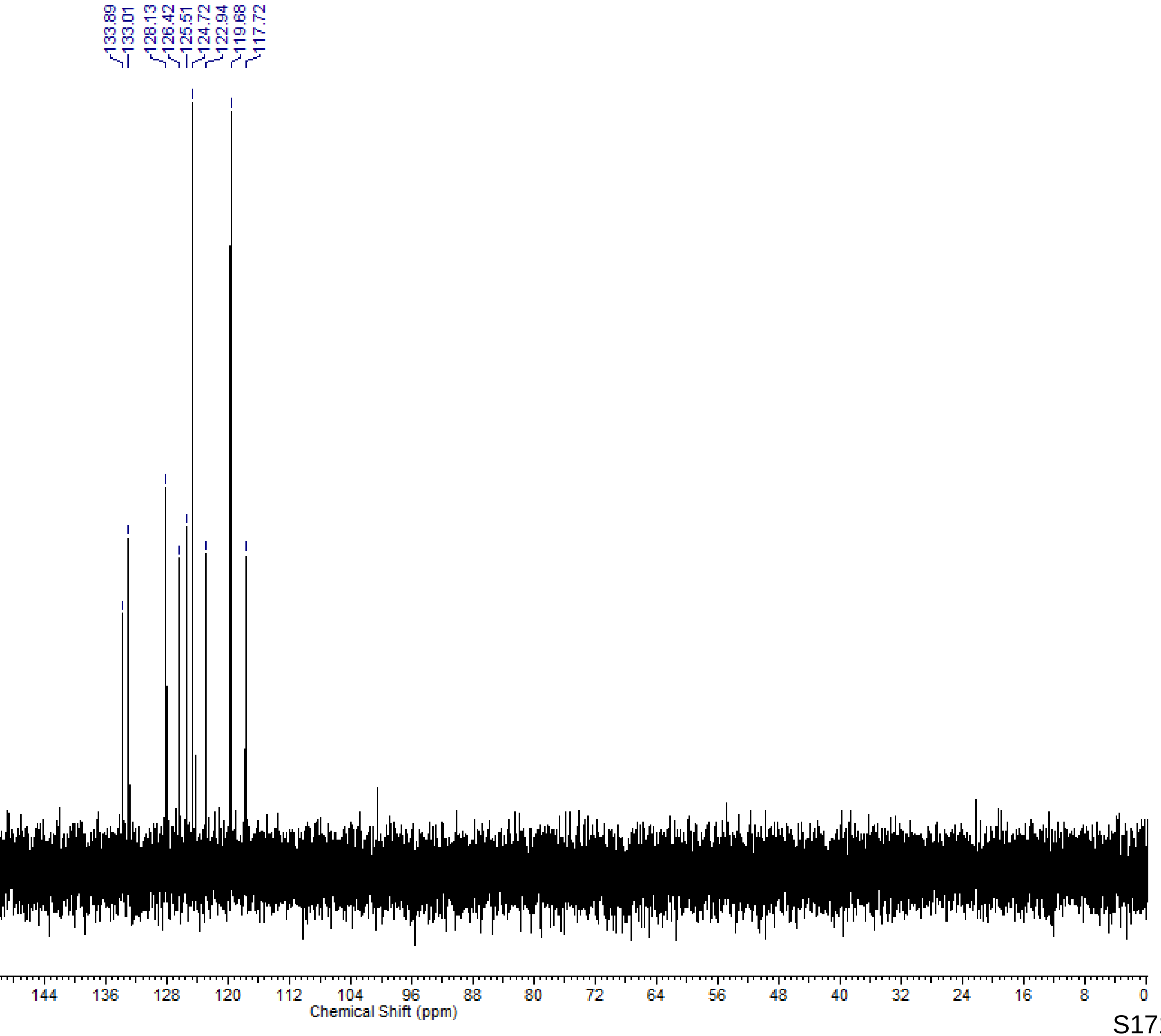

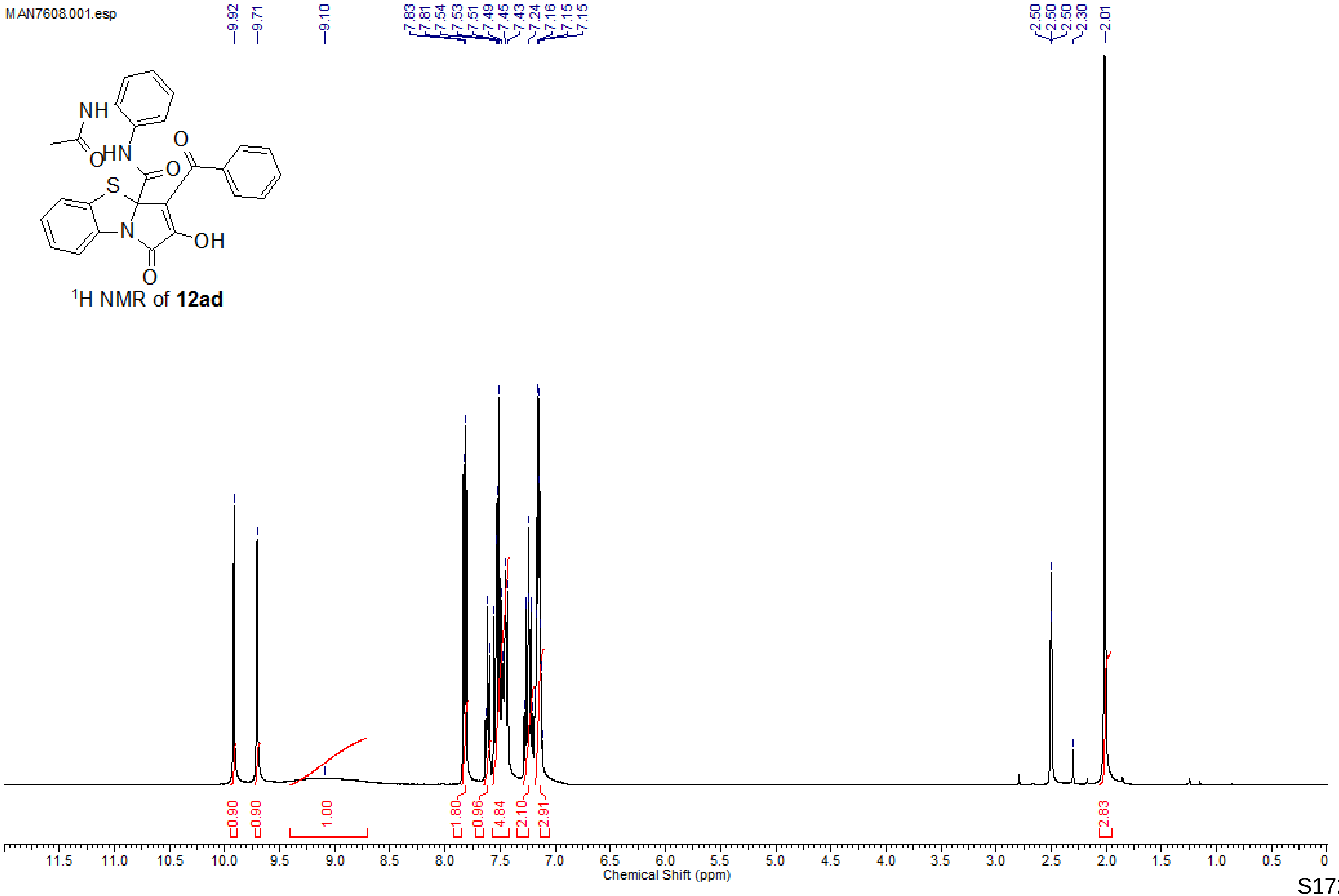

^{1}H NMR of **12ad**

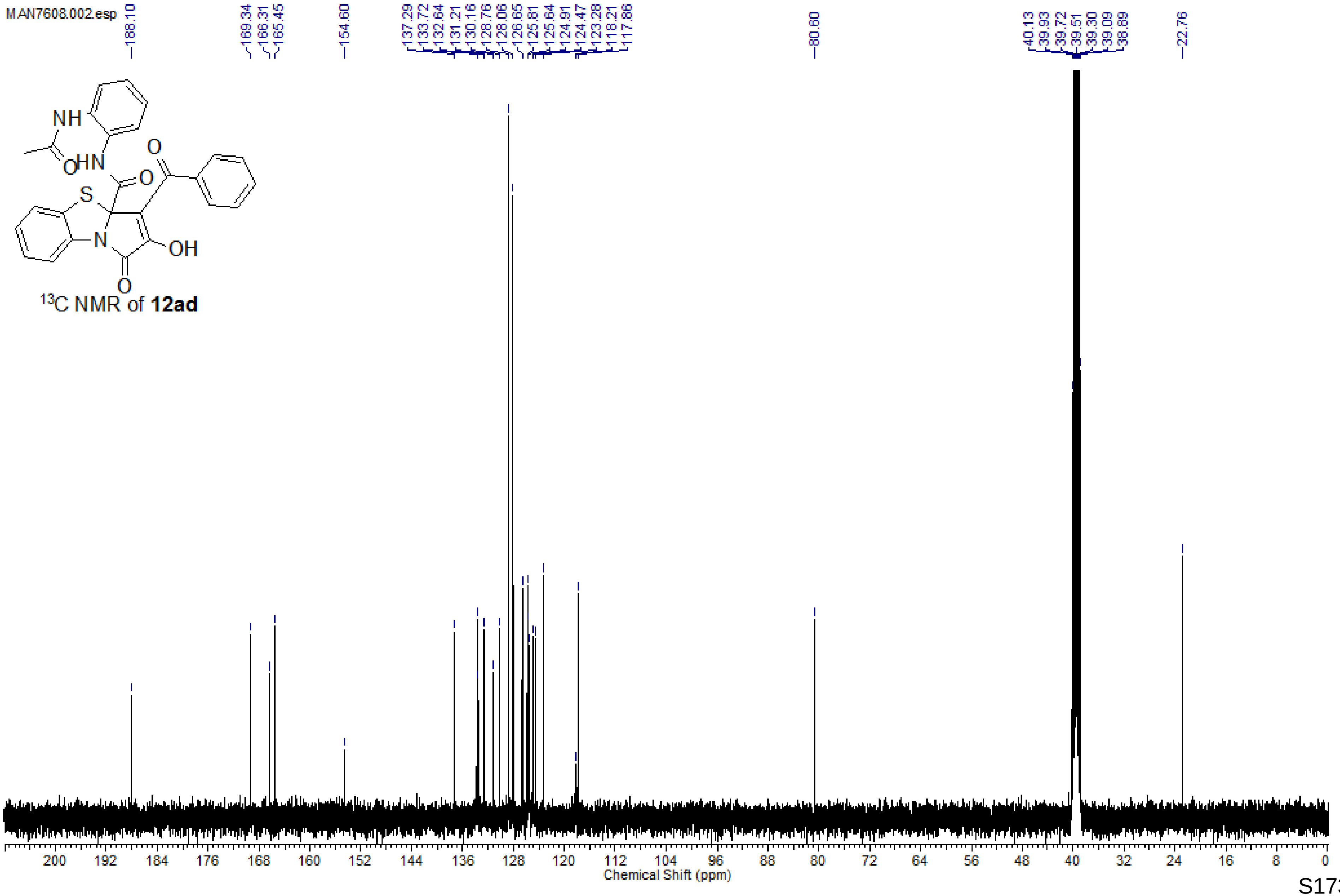

^{13}C NMR of **12ad**

MAN7608.003.esp

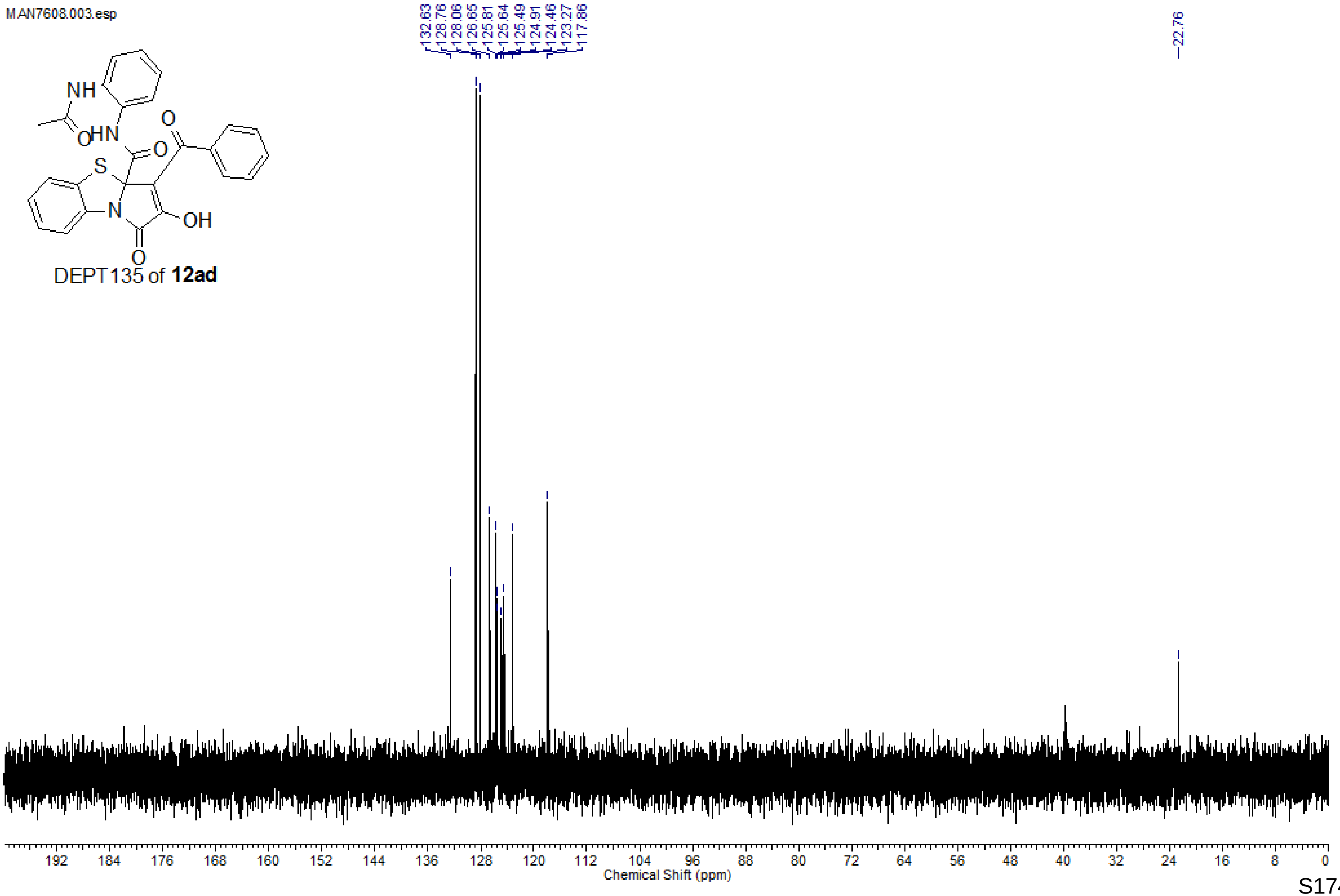

DEPT135 of **12ad**

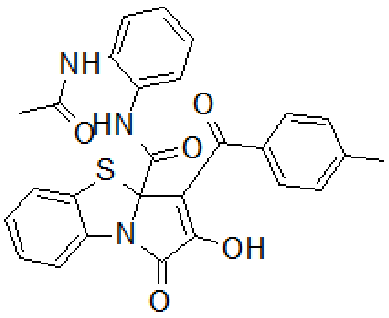

^{1}H NMR of **12bd**

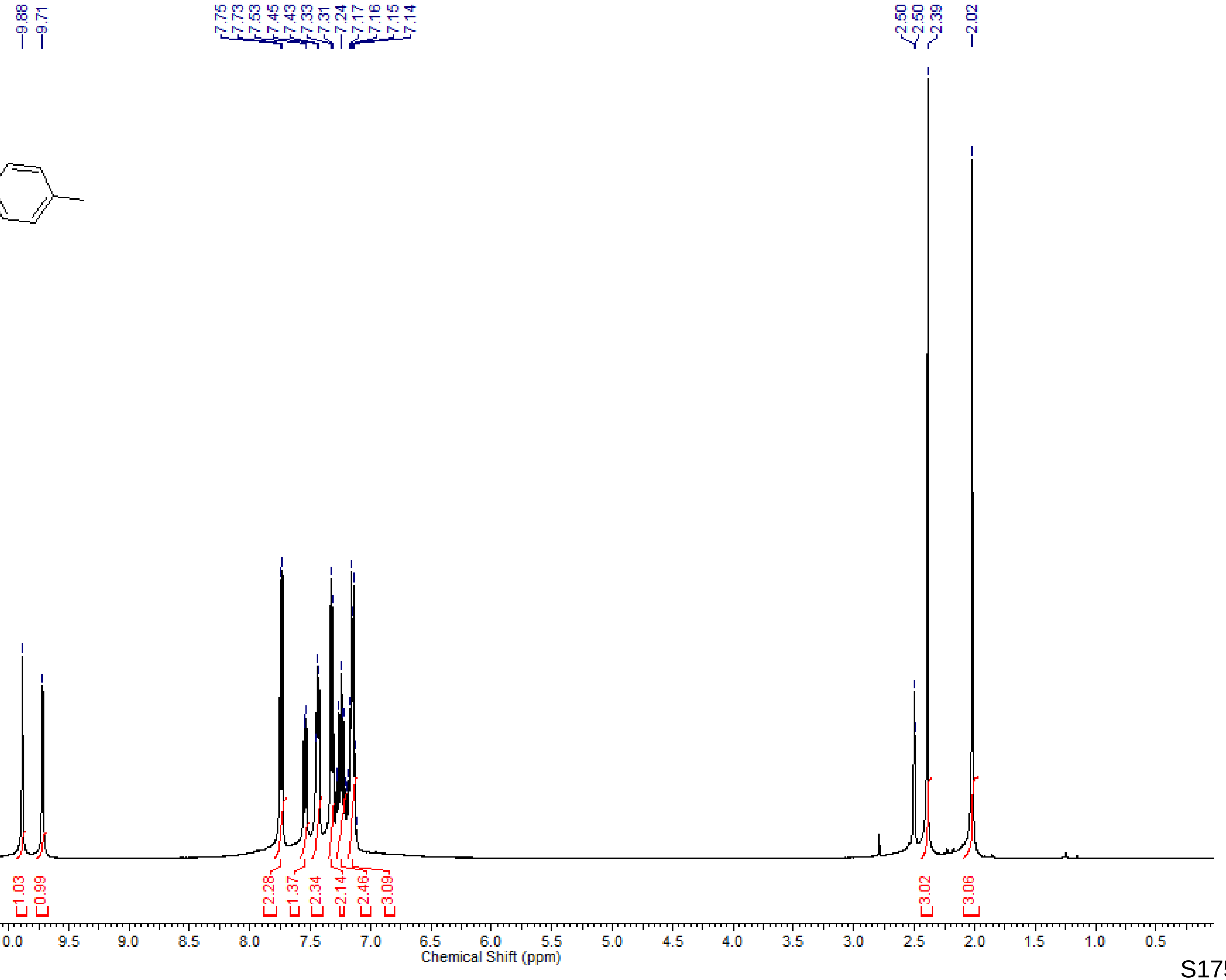

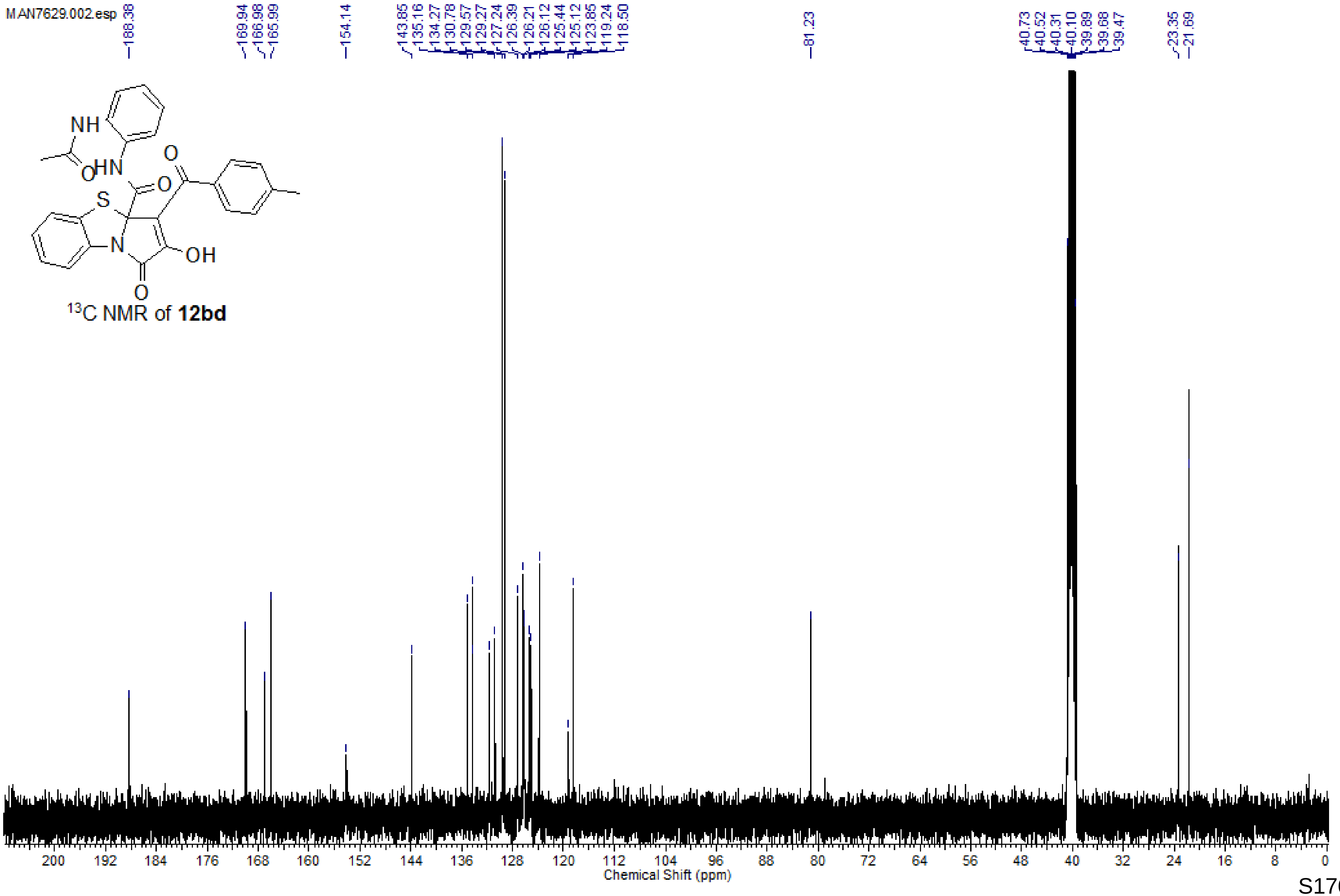

^{13}C NMR of **12bd**

MAN7629.003.esp

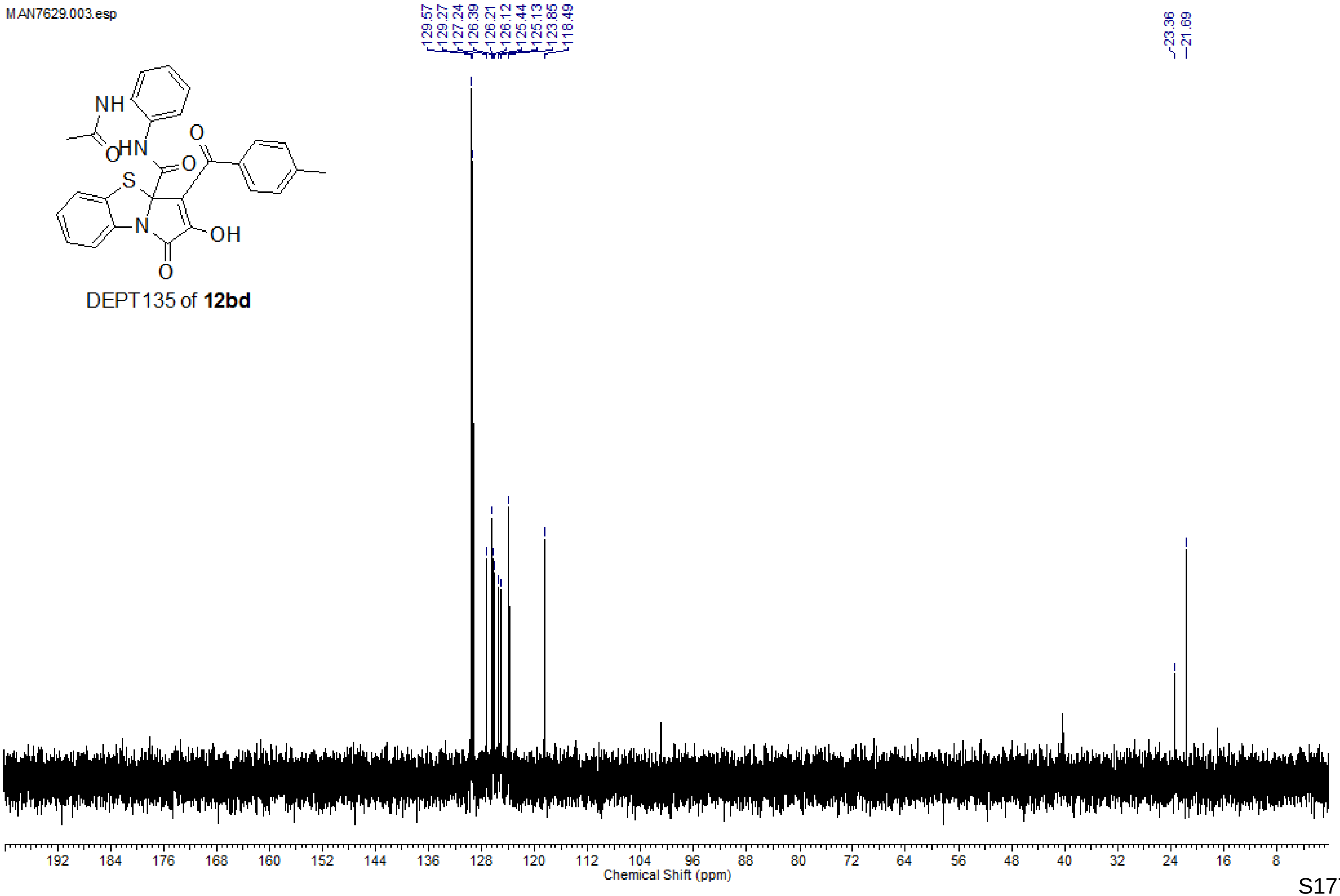

DEPT135 of **12bd**

_N_-Mesityl-2,4-dioxo-3-(2-oxo-2_H_-1,4-benzothiazin-3(4_H_)-ylidene)-4-phenylbutanamide (14ab)
A mixture of compound **1a** (150 mg, 0.45 mmol), mesitylamine **11b** (90 µL, 0.90 mmol), and anhydrous toluene (3 mL) is stirred in a closed microreaction V-vial at room temperature for 24 h. Then, the formed precipitate of compound **14ab** is filtered off and washed with toluene. According to NMR spectra of compound **14ab**, in a solution, it exists as a mixture of tautomers (Scheme 1) in a ratio of ~ 1:1, which is typical for similar compounds [4].

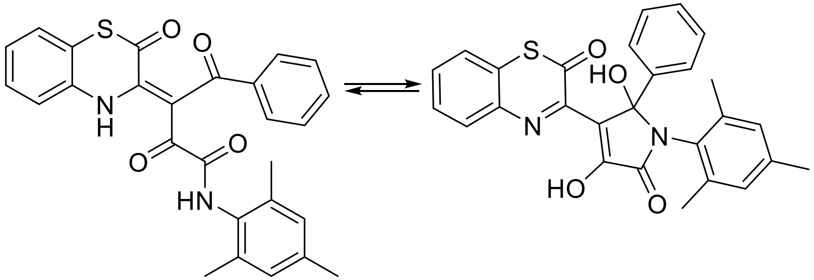

Scheme 1: Tautomers of compound **14ab**.

_N_-Mesityl-2,4-dioxo-3-(2-oxo-2_H_-1,4-benzothiazin-3(4_H_)-ylidene)-4-phenylbutanamide (14ab)
Orange solid; yield (30 mg, 14%); mp 177–178 °C (decomp.).
^{1}H NMR (400 MHz, DMSO-d_6): δ = 14.45 (s, 1H), 9.95 (s, 1H), 7.82 (m, 3H), 7.78 (m, 2H), 7.62 (m, 2H), 7.51 (m, 5H), 7.39 (m, 2H), 7.34 (m, 1H), 6.98 (m, 1H), 6.85 (m, 2H), 6.74 (m, 2H), 6.59 (m, 1H), 6.48 (m, 1H), 2.22 (s, 3H), 2.15 (s, 3H), 2.04 (s, 12H) ppm.
^{13}C NMR (100 MHz, DMSO-d_6): δ = 190.8, 187.4, 170.1, 163.7, 138.2, 137.7, 137.4, 137.2, 136.8, 134.9, 132.2, 129.4, 128.9 (2C), 128.5, 128.2, 128.1, 127.8 (2C), 127.1, 124.8, 118.8, 114.9, 113.6, 79.6, 20.2, 17.9, 17.6 ppm.
IR (mineral oil): 3255, 3054, 1785, 1728, 1683, 1645 cm^{-1}.
EA: Found: C, 69.21; H, 4.81 N, 5.80. Calc. for $C_{27}H_{22}N_2O_4S$: C, 68.92; H, 4.71; N, 5.95.

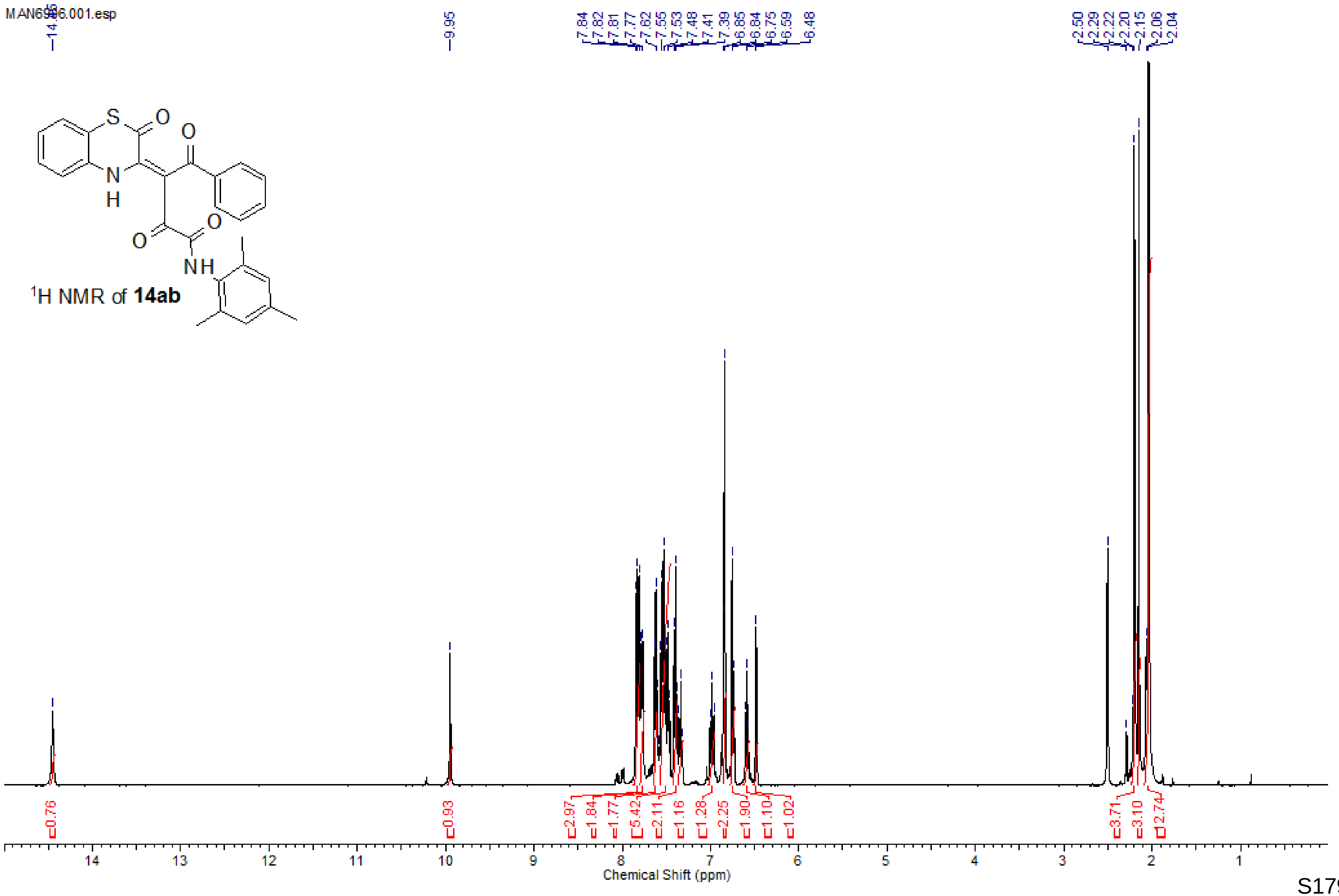
MAN6986.001.esp
14.45
9.95
7.84 7.82 7.81 7.77 7.62 7.55 7.53 7.48 7.41 7.39 6.85 6.84 6.75 6.59 6.48
2.50 2.29 2.22 2.20 2.15 2.06 2.04
S
O
O
N
H
O
O
NH
^{1}H NMR of **14ab**
0.76 0.93 2.97 1.84 1.77 5.42 2.11 1.16 1.28 2.25 1.90 1.10 1.02 3.71 3.10 12.74
Chemical Shift (ppm)

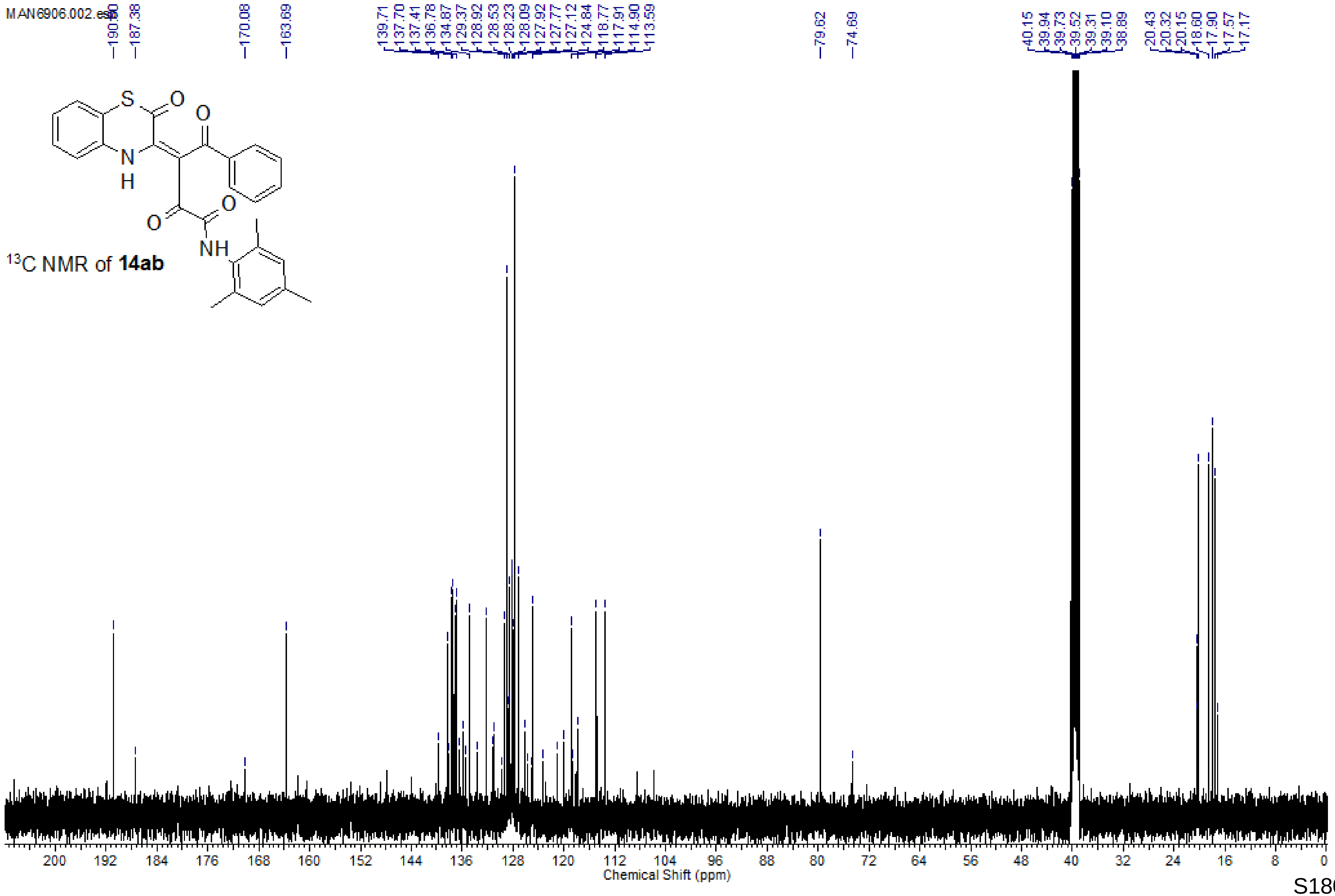

^{13}C NMR of **14ab**

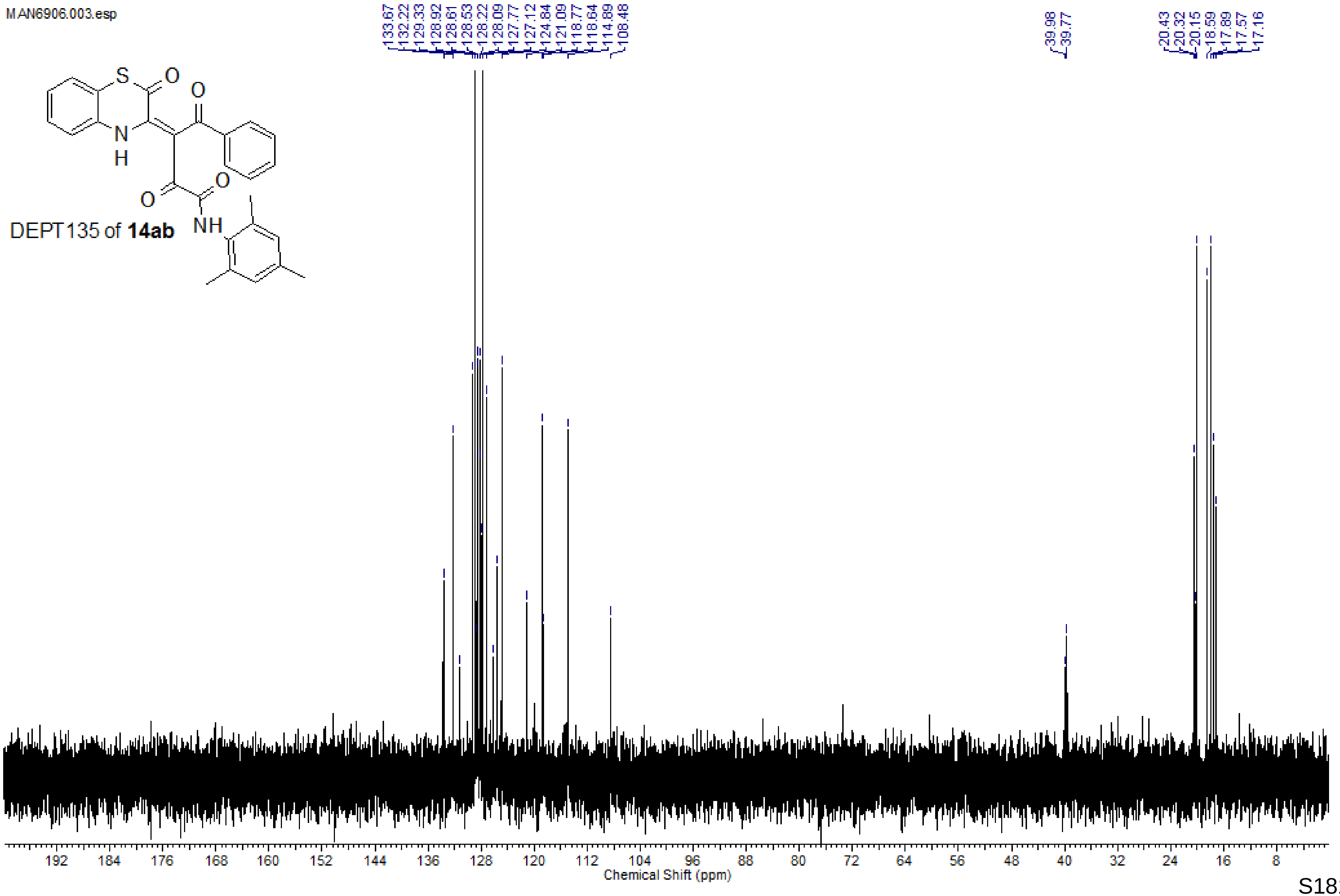
MAN6906.003.esp
133.67
132.22
129.33
128.92
128.61
128.53
128.22
128.09
127.77
127.12
124.84
121.09
118.77
118.64
114.89
108.48
39.98
39.77
20.43
20.32
20.15
18.59
17.89
17.57
17.16
S
O
O
N
H
O
O
NH
DEPT135 of **14ab**
192
184
176
168
160
152
144
136
128
120
112
104
96
88
80
72
64
56
48
40
32
24
16
8
Chemical Shift (ppm)

3-Benzoylpyrrolo[2,1-*b*][1,3]benzothiazole-1,2-dione (17a)

A mixture of APBTT **1a** (150 mg, 0.45 mmol), benzhydrol (83 mg, 0.45 mmol), and anhydrous toluene (3 mL) was stirred in a closed microreaction V-vial at 113 °C for 2 h. Then, the resulting solution is cooled to room temperature. In 24 h, the formed precipitate of compound **17a** is filtered off and washed with anhydrous toluene.

Red solid; yield (97 mg, 70%); mp 176–178 °C (decomp., toluene).
^{1}H NMR (400 MHz, DMSO-d_6): δ = 8.07 (m, 1H), 7.98 (m, 1H), 7.94 (m, 1H), 7.61 (m, 2H), 7.57–7.40 (m, 4H).
^{13}C NMR (100 MHz, $CDCl_3$): δ = 185.1, 184.4, 171.7, 154.1, 136.7, 133.3, 132.8, 129.4 (2C), 129.3, 128.5, 128.2 (2C), 127.0, 123.6, 114.8, 107.2.
IR (mineral oil): 1784, 1742, 1658 cm^{-1}.
EA: Found: C, 66.53; H, 2.99; N, 4.61. Calc. for $C_{17}H_9NO_3S$: C, 66.44; H, 2.95; N, 4.56.

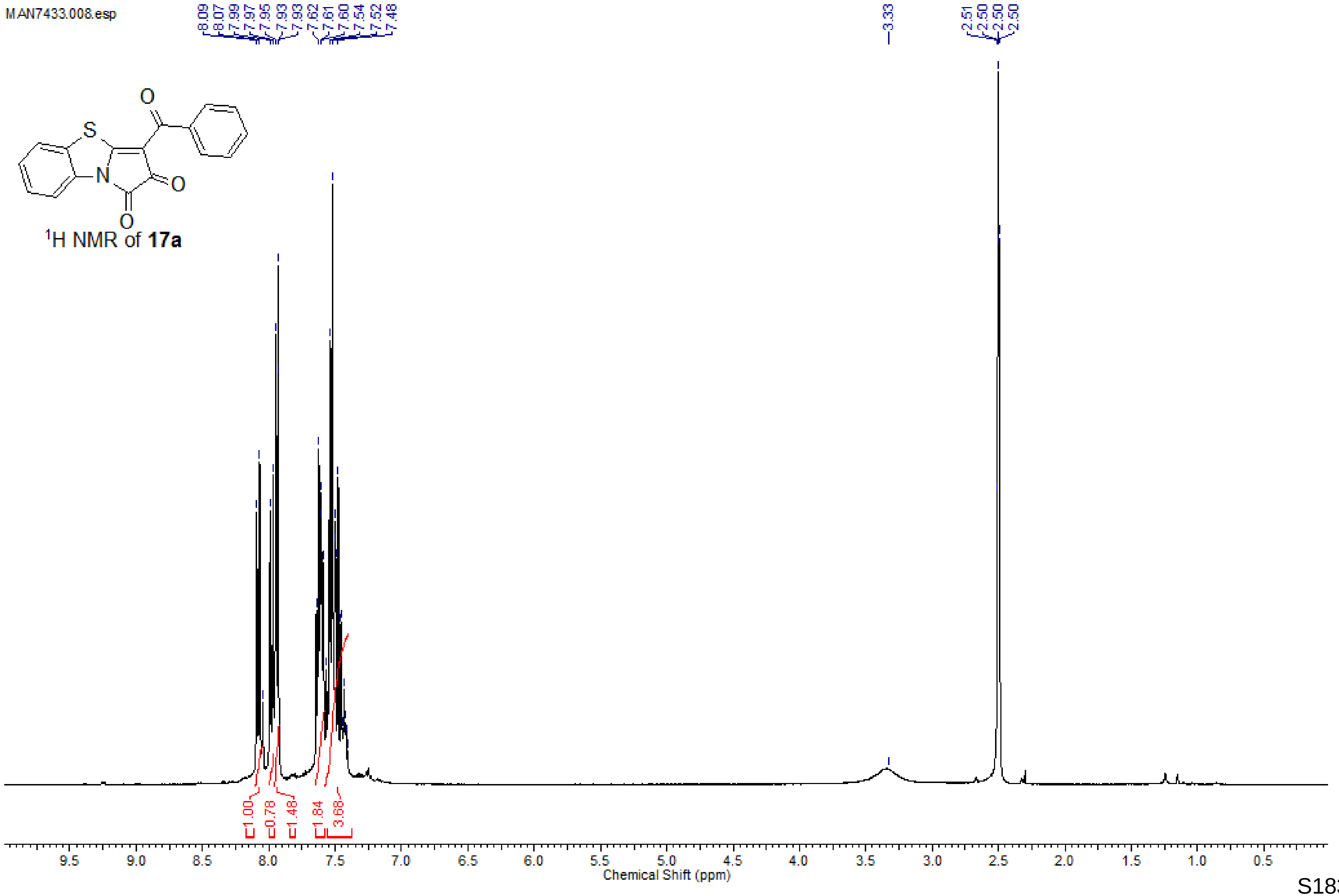

MAN7433.008.esp
8.09
8.07
7.99
7.97
7.95
7.93
7.93
7.62
7.61
7.60
7.54
7.52
7.48
3.33
2.51
2.50
2.50
2.50
S
N
O
O
O
1.00
0.78
1.48
1.84
3.68
9.5
9.0
8.5
8.0
7.5
7.0
6.5
6.0
5.5
5.0
4.5
4.0
3.5
3.0
2.5
2.0
1.5
1.0
0.5
Chemical Shift (ppm)

^{1}H NMR of **17a**

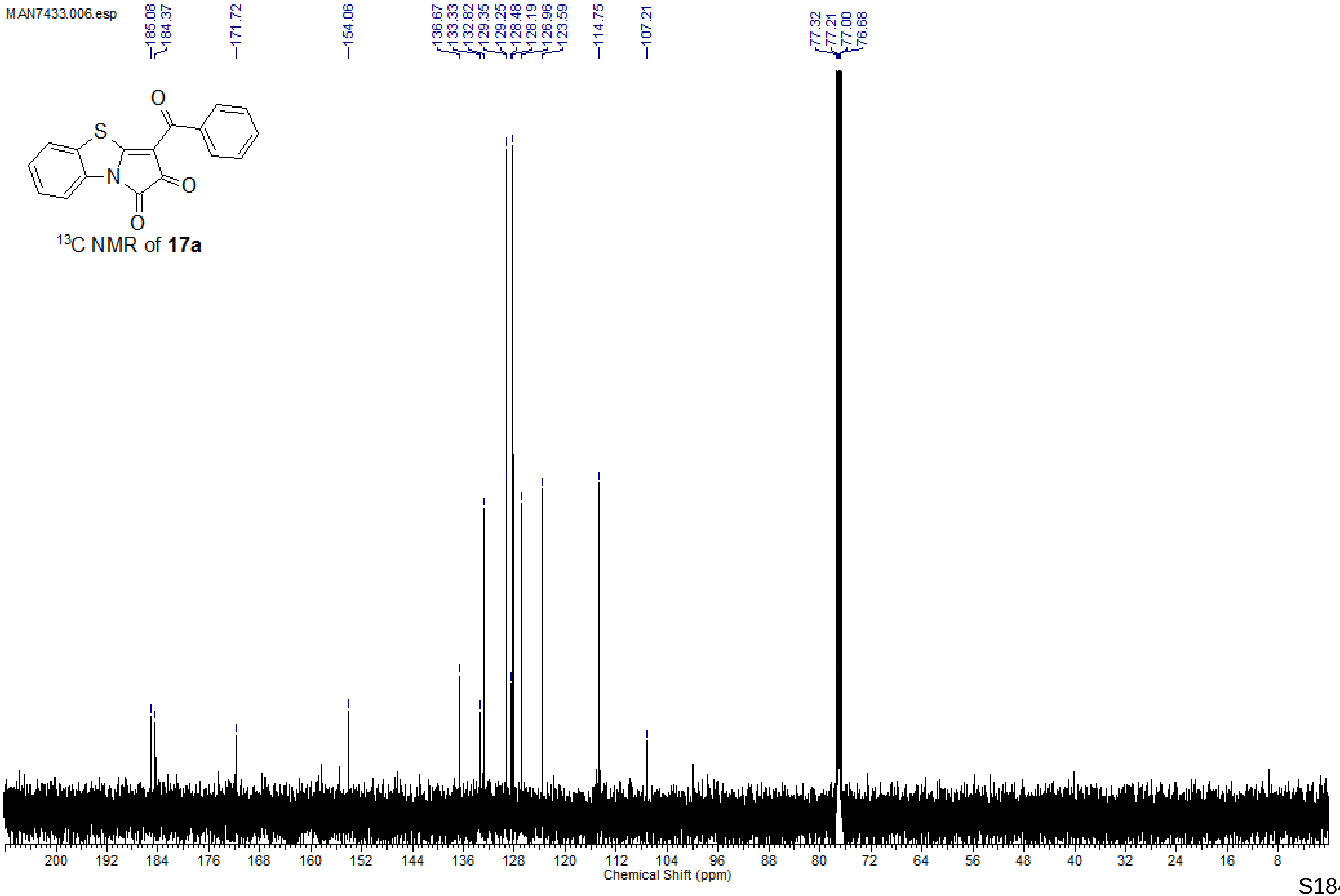
MAN7433.006.esp
185.08
184.37
171.72
154.06
136.67
133.33
132.82
129.35
129.25
128.48
128.19
126.96
123.59
114.75
107.21
77.32
77.21
77.00
76.68
O
S
N
O
O
13C NMR of 17a
200 192 184 176 168 160 152 144 136 128 120 112 104 96 88 80 72 64 56 48 40 32 24 16 8
Chemical Shift (ppm)

MAN7433.007.esp

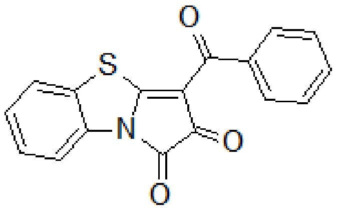

DEPT135 of **17a**

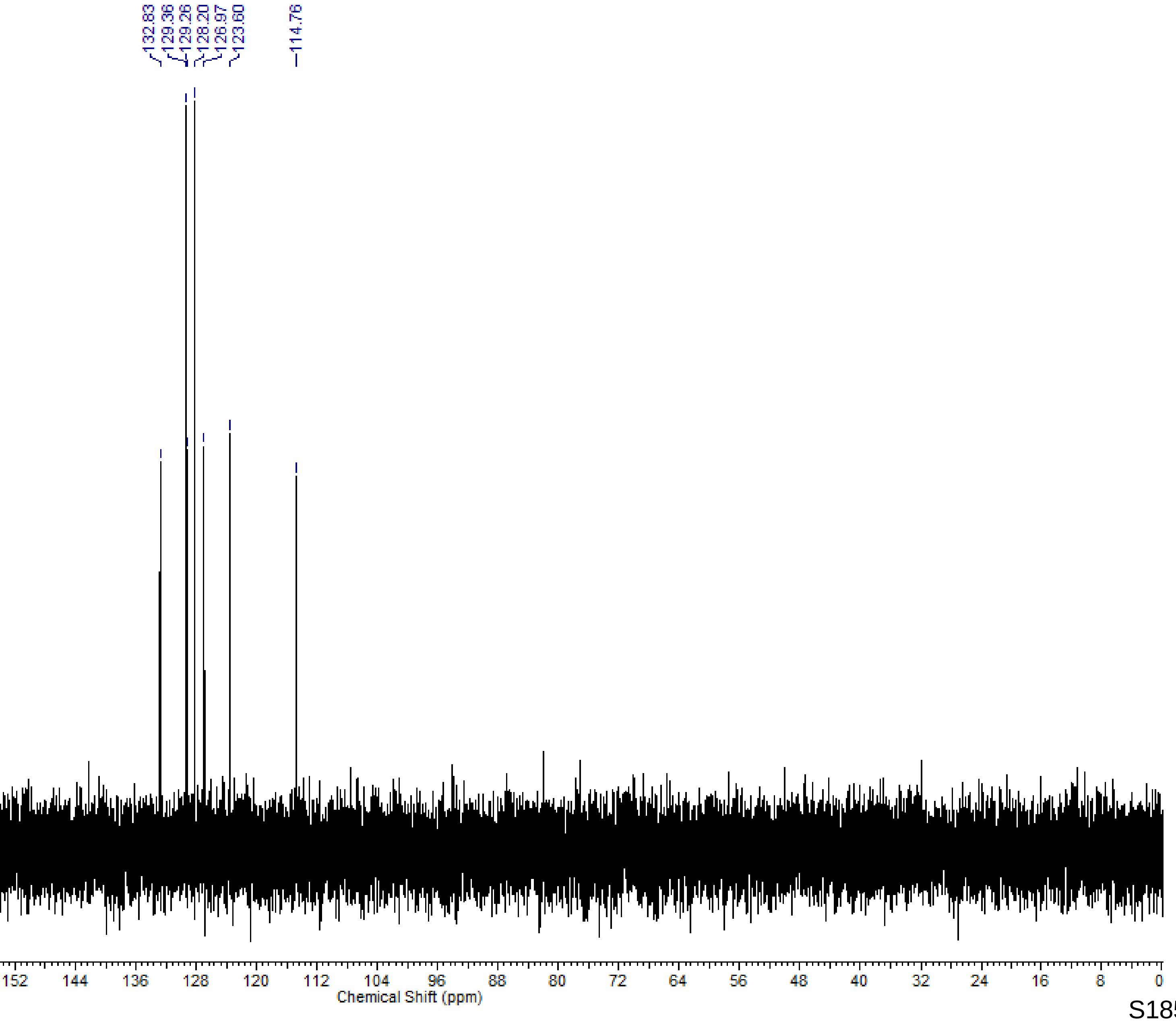

Preparation of samples for HPLC-UV analyses for optimization. General procedure to compounds 4, 8, 13.

Reaction mixtures are obtained according scales and conditions from Tables 1-3 of the main manuscript. Then, solvents are removed from the reaction mixtures at room temperature. After that, anhydrous toluene (0.5 mL) and DCC (1.1 equiv) are added to the reaction mixtures. The obtained mixtures are stirred for 1 h at 113 °C. The resulting reaction mixtures are cooled to room temperature. Then, biphenyl (10 mg) (internal standard) and chloroform (0.5 mL) are added (dicyclohexylurea precipitate is observed). Next, aliquots (0.1 mL) of the obtained mixtures are diluted with acetonitrile (0.9 mL). The obtained solutions are used for HPLC-UV.

The treatment with DCC is necessary to derivatize compounds **3aa**, **7a**, **12aa** to corresponding compounds **4, 8, 13** by a protocol developed by us earlier [5] in order to obtain good HPLC-UV signals.

General procedure to compounds 4, 8, 13.
A mixture of compounds **3aa**, **7a** or **12aa** (0.54 mmol) and DCC (0.59 mmol) in toluene (3 mL) was stirred in an oven-dried closed microreaction V-vial at 113 °C for 1 h. Then, the resulting solution is cooled to room temperature. The formed precipitate of dicyclohexylurea is filtered off, washed with toluene. Then, the solvent is removed under vacuum from the obtained solution. The resulting reaction mixture is recrystallized from ethanol to afford compound **4**. Compounds **8, 13** are purified with a column chromatography (toluene/ethyl acetate, 25:1) and should not be dried in an oven since they can decompose.

Methyl 3-benzoyl-2-[cyclohexyl(cyclohexylcarbamoyl)amino]-1-oxopyrrolo[2,1-*b*][1,3]benzothiazole-3a(1*H*)-carboxylate (4)
Yellow solid; yield (307 mg, 99%); mp 184–186 °C (decomp., EtOH).
^{1}H NMR (400 MHz, DMSO-*d*$_6$): δ = 7.94 (m, 2H), 7.72 (m, 1H), 7.53 (m, 2H), 7.45 (m, 2H), 7.28–7.17 (m, 2H), 6.50 (m, 1H), 3.55 (m, 4H), 3.35 (m, 1H), 3.26 (s, 1H), 1.72 (m, 3H), 1.60–1.43 (m, 4H), 1.35 (m, 2H), 1.26–1.18 (m, 4H), 1.16–1.03 (m, 5H), 0.89–0.79 (m, 1H) ppm.
^{13}C NMR (100 MHz, DMSO-*d*$_6$): δ = 190.3, 168.4, 166.3, 154.0, 141.3, 137.8, 135.7, 135.2, 134.5, 134.3, 129.2 (2C), 128.6 (2C), 126.2, 126.0, 122.9, 118.1, 77.8, 58.3, 53.6, 49.6, 32.5, 32.4, 31.8, 30.1, 25.3, 25.2 (2C), 25.0, 25.0, 24.8 ppm.
IR (mineral oil): 3418, 1749, 1721, 1656, 1639 cm^{-1}.
EA: Found: C, 67.10; H, 6.20; N, 7.33. Calc. for $C_{32}H_{35}N_3O_5S$: C, 66.99; H, 6.15; N, 7.32.

3-Benzoyl-*N*-benzyl-2-[cyclohexyl(cyclohexylcarbamoyl)amino]-1-oxopyrrolo[2,1-*b*][1,3]benzothiazole-3a(1*H*)-carboxamide (8)
Yellow solid; yield (347 mg, 99%); mp 113–115 °C (decomp.).

^{1}H NMR (400 MHz, $CDCl_3$): δ = 7.90 (m, 2H), 7.83 (m, 1H), 7.74 (m, 1H), 7.69 (m, 1H), 7.54 (m, 2H), 7.35 (m, 3H), 7.27 (m, 4H), 7.15 (m, 1H), 5.27 (m, 1H), 4.59–4.39 (m, 2H), 3.92 (m, 1H), 3.51 (m, 1H), 2.47 (m, 1H), 1.98 (m, 1H), 1.87 (m, 2H), 1.77 (m, 5H), 1.64 (m, 3H), 1.54 (m, 1H), 1.39 (m, 2H), 1.25 (m, 4H), 1.11–0.94 (m, 1H) ppm.
^{13}C NMR (100 MHz, $CDCl_3$): δ = 190.6, 167.9, 166.1, 154.2, 142.6, 140.0, 136.8, 135.9, 134.7, 134.0, 132.8, 129.0, 128.6, 128.6 (2C), 128.2 (2C), 127.6, 127.2, 126.8, 126.6, 125.3, 123.2, 118.6, 81.3, 59.3, 50.0, 44.7, 33.2, 33.0, 31.3, 31.2, 26.2, 26.1, 25.6, 25.3, 24.9, 24.8 ppm.
IR (thin layer obtained from $CHCl_3$): 3380, 1732, 1677, 1639 cm^{-1}.
EA: Found: C, 71.35; H, 6.37; N, 8.33. Calc. for $3C_{38}H_{40}N_4O_4S \cdot C_7H_8$: C, 71.29; H, 6.33; N, 8.25.

3-Benzoyl- 2-[cyclohexyl(cyclohexylcarbamoyl)amino]-1-oxo-*N*-phenylpyrrolo[2,1-*b*][1,3]benzothiazole-3a(1*H*)-carboxamide (13)
Yellow solid; yield (339 mg, 88%, purity 90%); mp 116–118 °C (decomp.).
^{1}H NMR (400 MHz, $CDCl_3$): δ = 9.17 (s, 1H), 7.84 (m, 2H), 7.79 (m, 1H), 7.60 (m, 1H), 7.47 (m, 3H), 7.28 (m, 3H), 7.22 (m, 2H), 7.15–7.09 (m, 2H), 5.10 (m, 1H), 3.82 (m, 1H), 3.46 (m, 1H), 2.59 (m, 2H), 2.04 (m, 2H), 1.81 (m, 2H), 1.73 (m, 4H), 1.58 (m, 3H), 1.48 (m, 1H), 1.34 (m, 2H), 1.24–1.08 (m, 3H), 0.92 (m, 1H) ppm.
^{13}C NMR (100 MHz, $CDCl_3$): δ = 190.6, 168.2, 164.0, 154.2, 142.5, 140.2, 136.7, 135.9, 134.7, 134.1, 132.4, 129.1, 129.0 (2C), 128.7, 128.3, 127.0, 126.8, 125.4, 123.3, 121.6, 120.2 (2C), 118.8, 81.8, 59.5, 50.0, 33.2, 33.0, 31.4, 31.2, 26.3, 26.1, 25.6, 25.3, 24.9, 24.8 ppm.
IR (thin layer obtained from $CHCl_3$): 3346, 1735, 1682, 1639 cm^{-1}.
EA: Found: C, 70.33; H, 6.25; N, 8.97. Calc. for $C_{37}H_{38}N_4O_4S$: C, 70.01; H, 6.03; N, 8.83.

MAN7599.001.esp

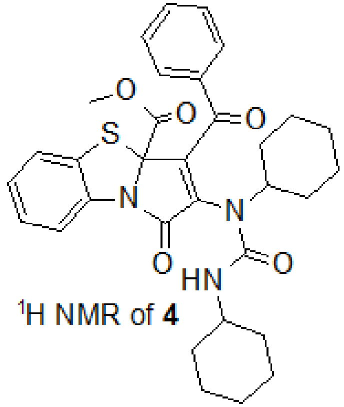

^{1}H NMR of **4**

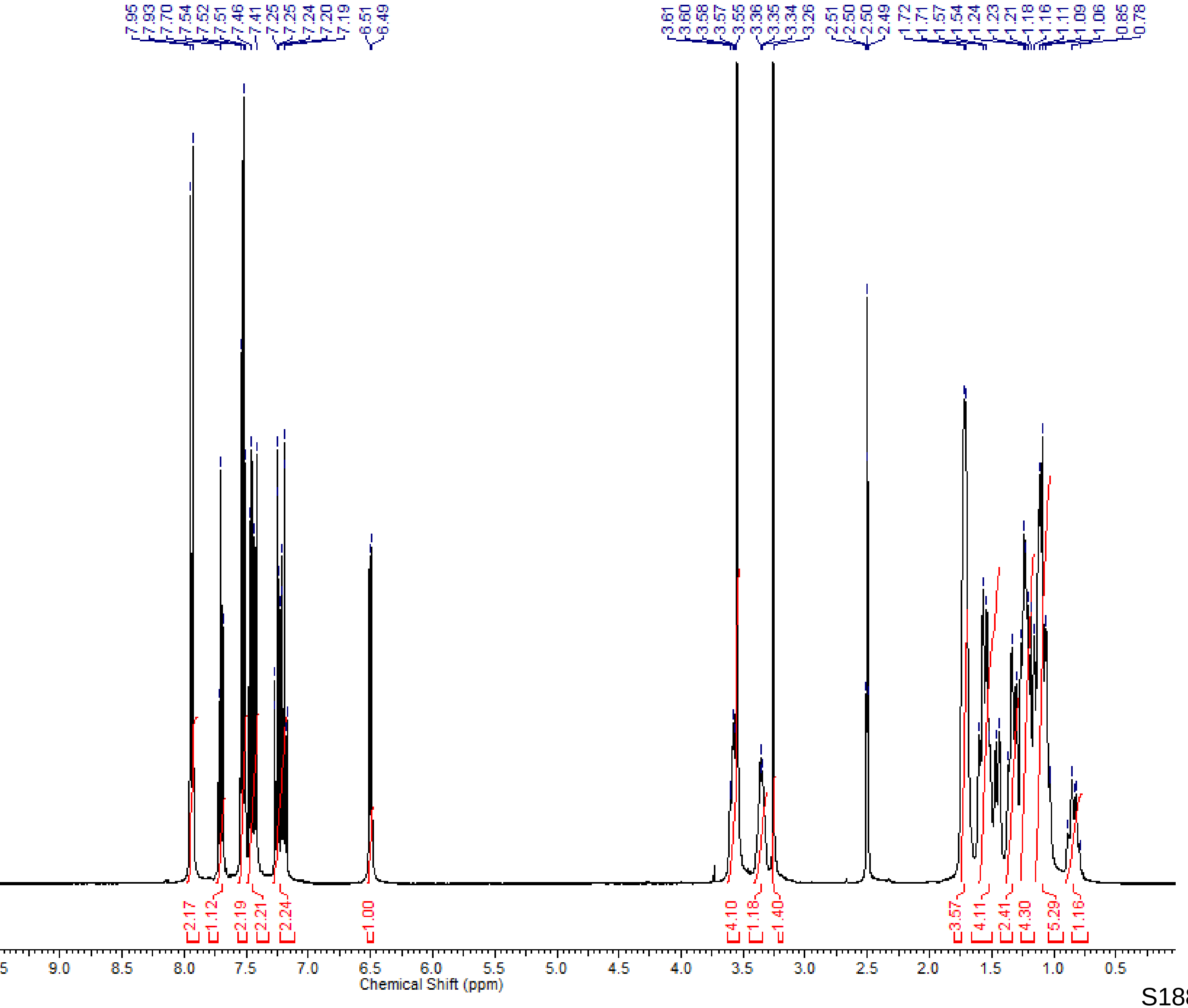

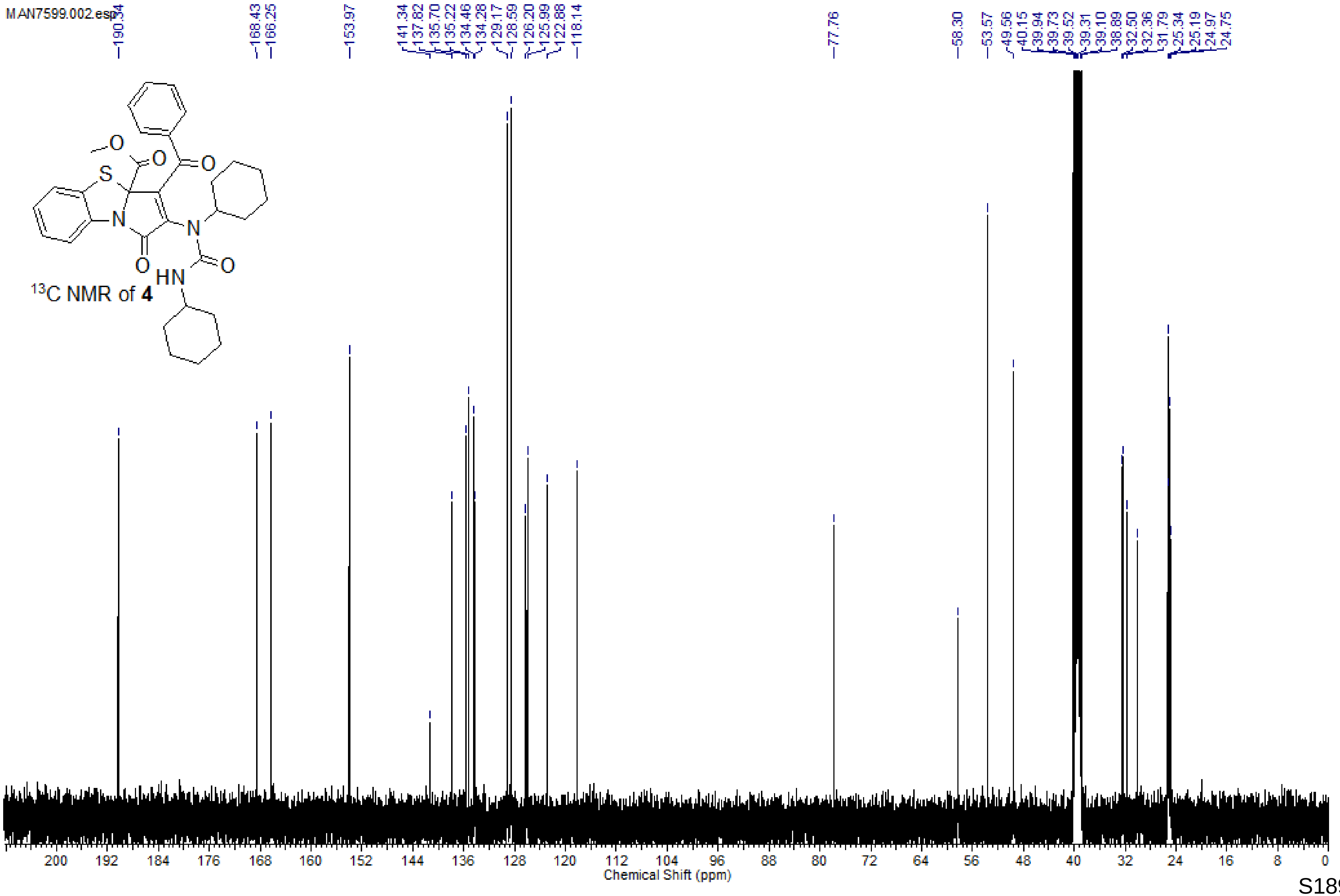
MAN7599.002.esp
190.34
168.43
166.25
153.97
141.34
137.82
135.70
135.22
134.46
134.28
129.17
128.59
126.20
125.99
122.88
118.14
77.76
58.30
53.57
49.56
40.15
39.94
39.73
39.52
39.31
39.10
38.89
32.50
32.36
31.79
25.34
25.19
24.97
24.75
13C NMR of 4
200 192 184 176 168 160 152 144 136 128 120 112 104 96 88 80 72 64 56 48 40 32 24 16 8 0
Chemical Shift (ppm)

MAN7599.003.esp

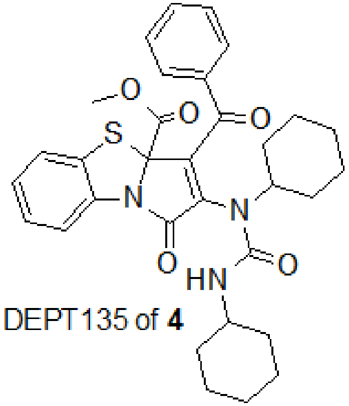

DEPT135 of **4**

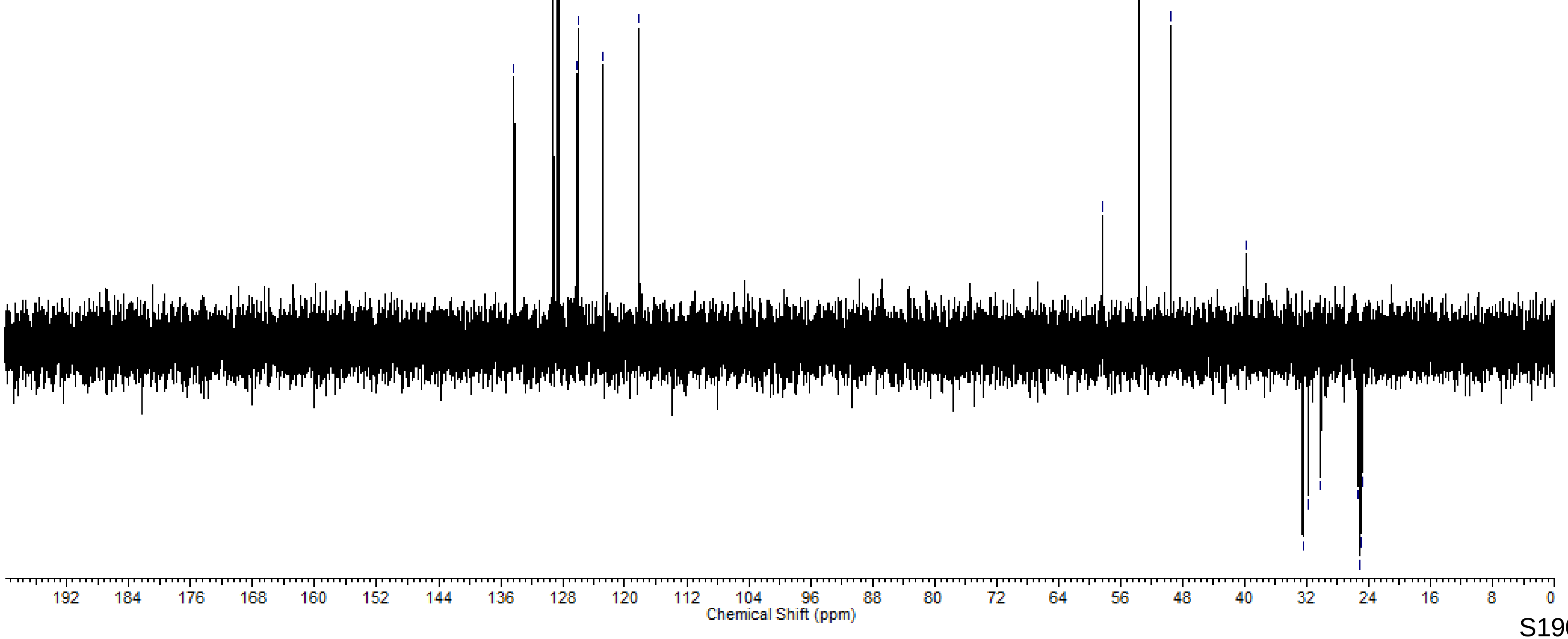

MAN7862.001.esp

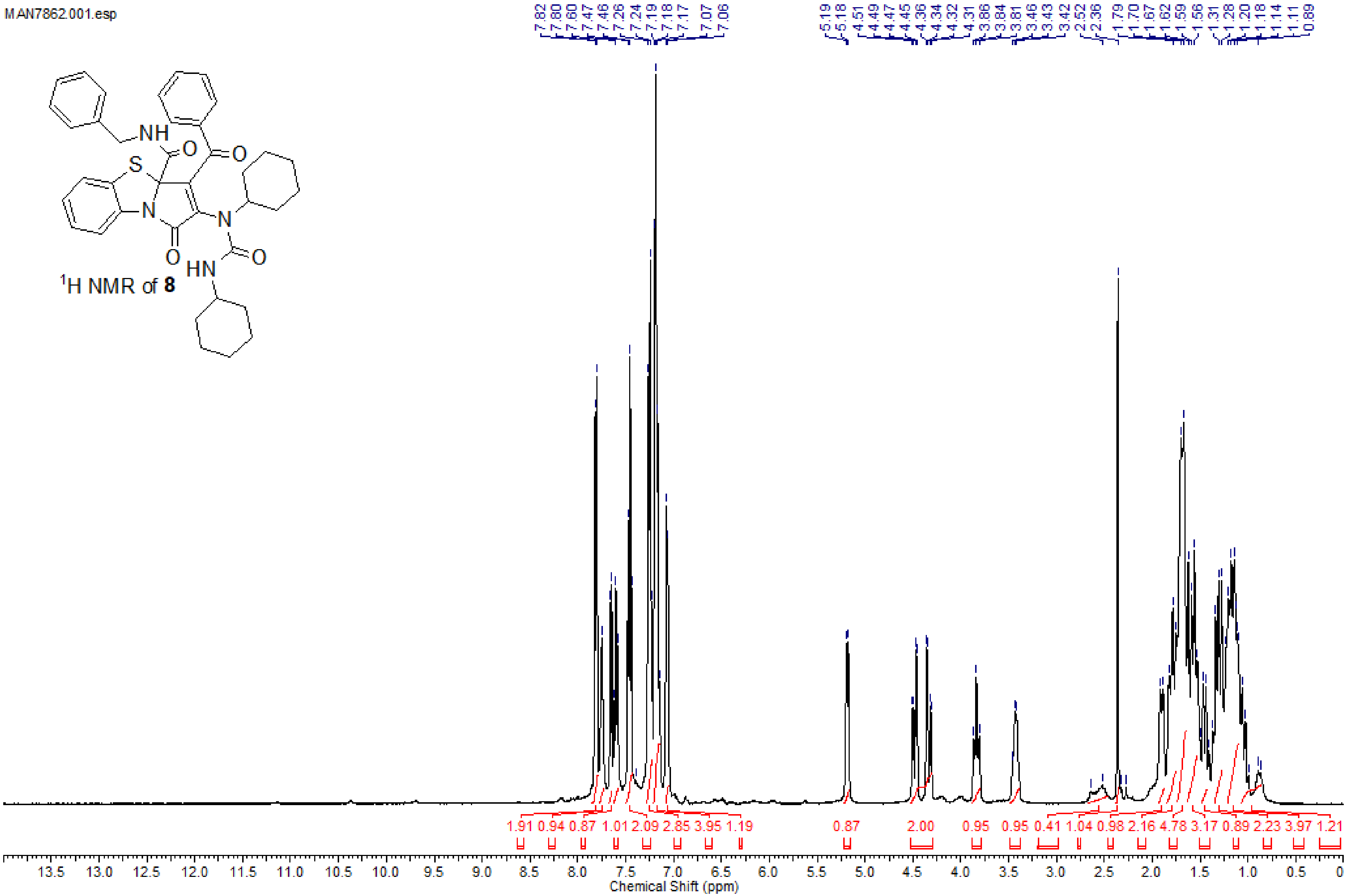

^{1}H NMR of **8**

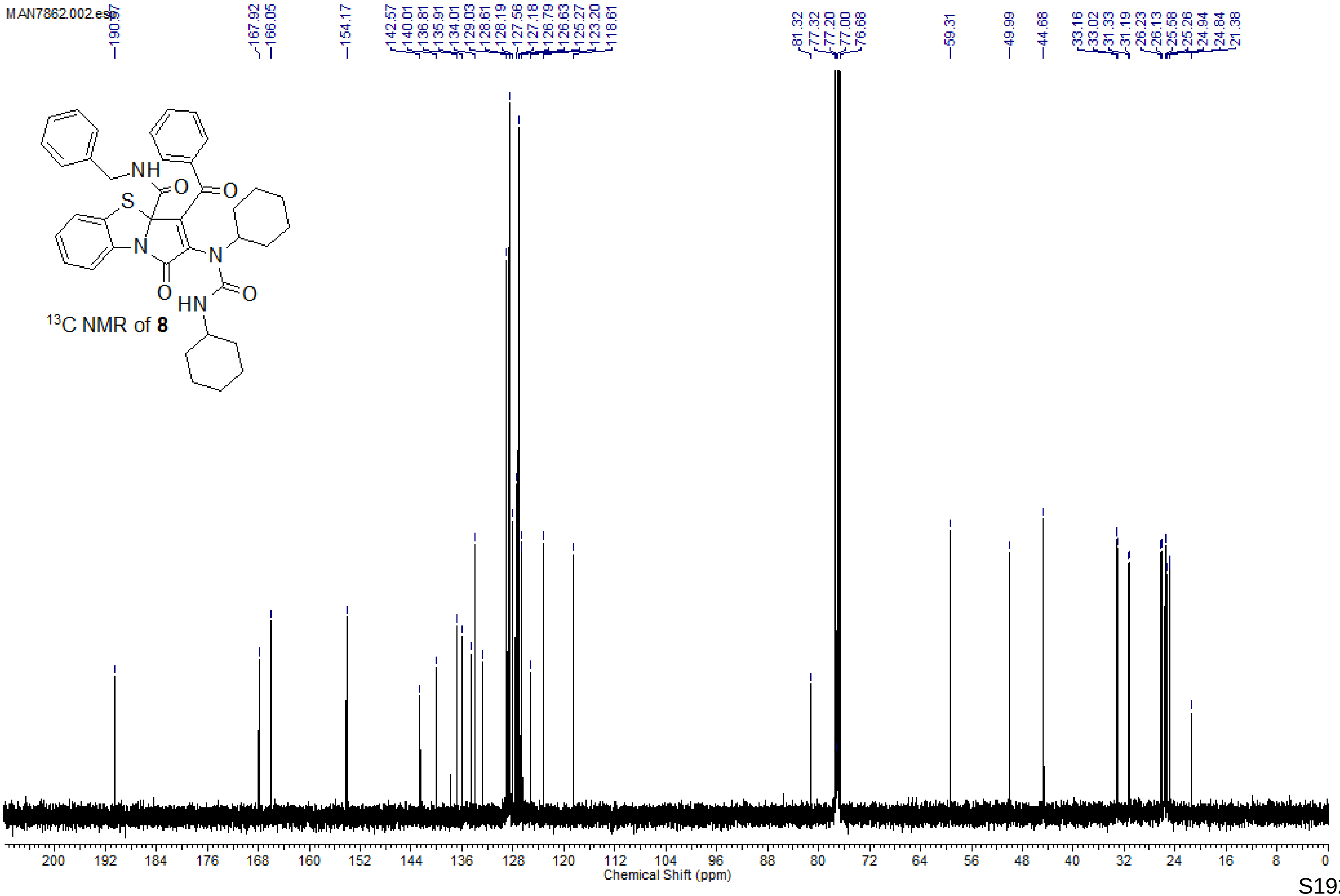
MAN7862.002.esp
190.97
167.92
166.05
154.17
142.57
140.01
136.81
135.91
134.01
129.03
128.61
128.19
127.56
127.18
126.79
126.63
125.27
123.20
118.61
81.32
77.32
77.20
77.00
76.68
59.31
49.99
44.68
33.16
33.02
31.33
31.19
26.23
26.13
25.58
25.26
24.94
24.84
21.38
NH
O
S
N
O
N
O
HN
O
200 192 184 176 168 160 152 144 136 128 120 112 104 96 88 80 72 64 56 48 40 32 24 16 8 0
Chemical Shift (ppm)

^{13}C NMR of **8**

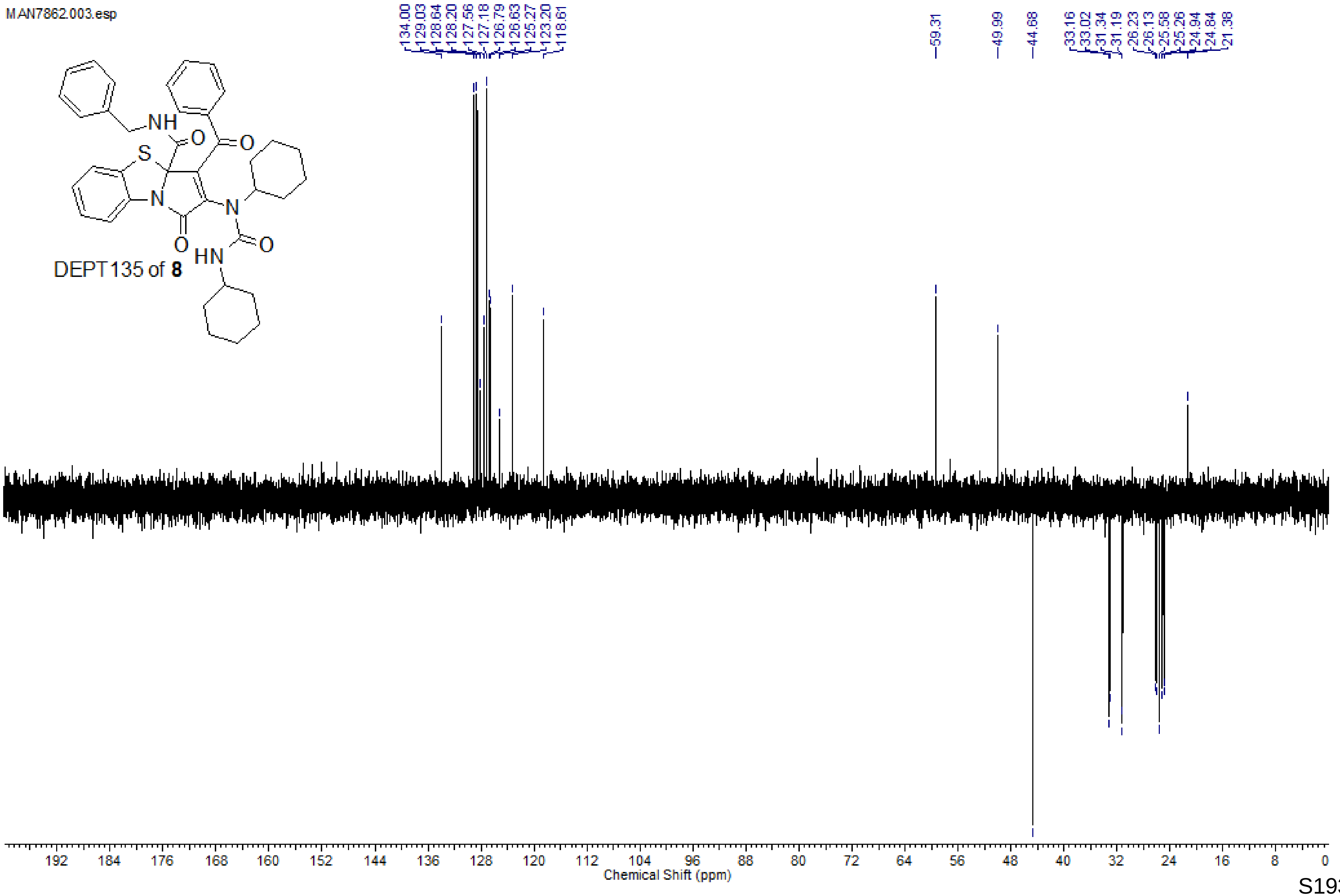

MAN7862.003.esp
134.00
129.03
128.64
128.20
127.56
127.18
126.79
126.63
125.27
123.20
118.61
59.31
49.99
44.68
33.16
33.02
31.34
31.19
26.23
26.13
25.58
25.26
24.94
24.84
21.38
NH
O
S
O
N
N
O
HN
O
192
184
176
168
160
152
144
136
128
120
112
104
96
88
80
72
64
56
48
40
32
24
16
8
0
Chemical Shift (ppm)

DEPT135 of **8**

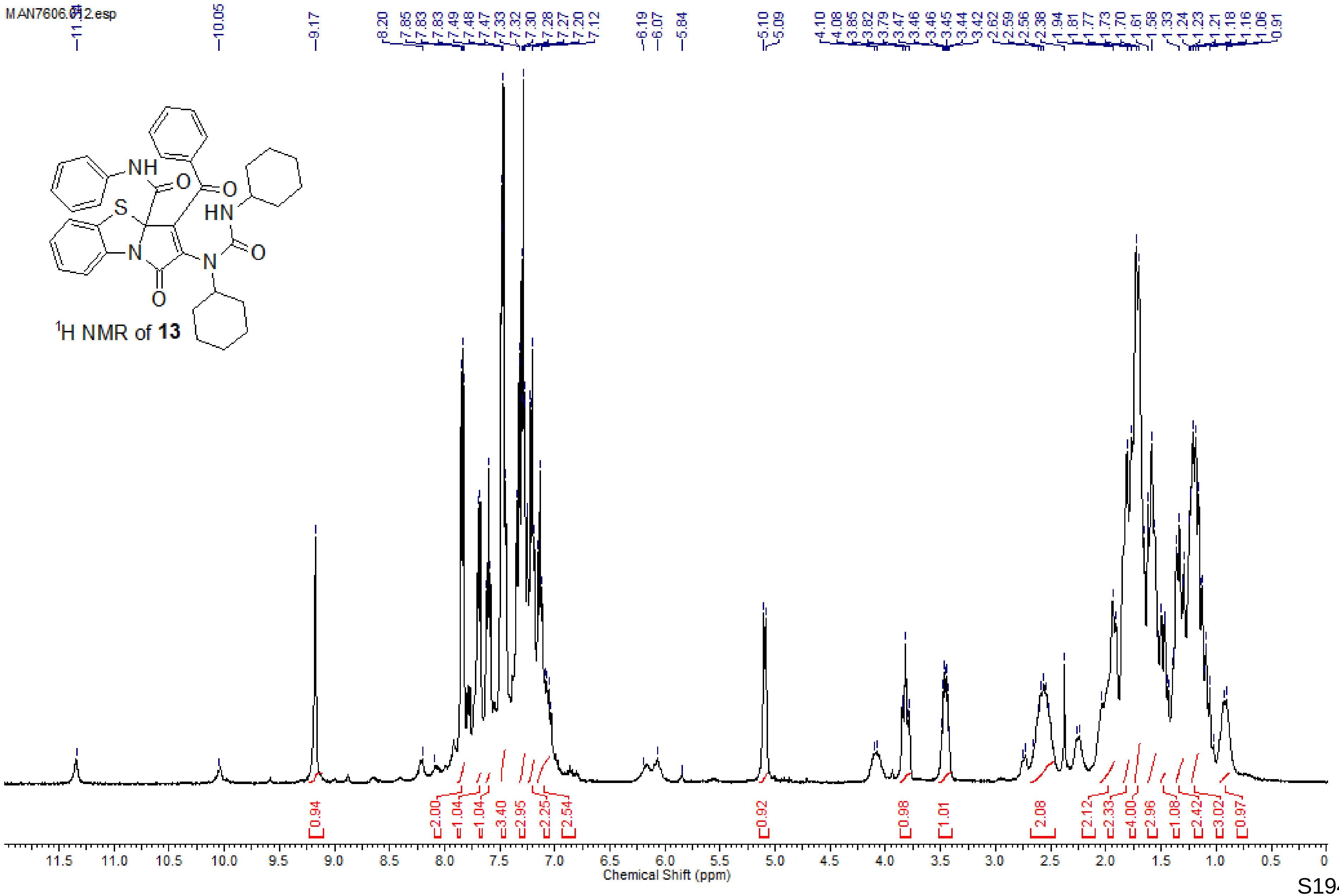

^{1}H NMR of **13**

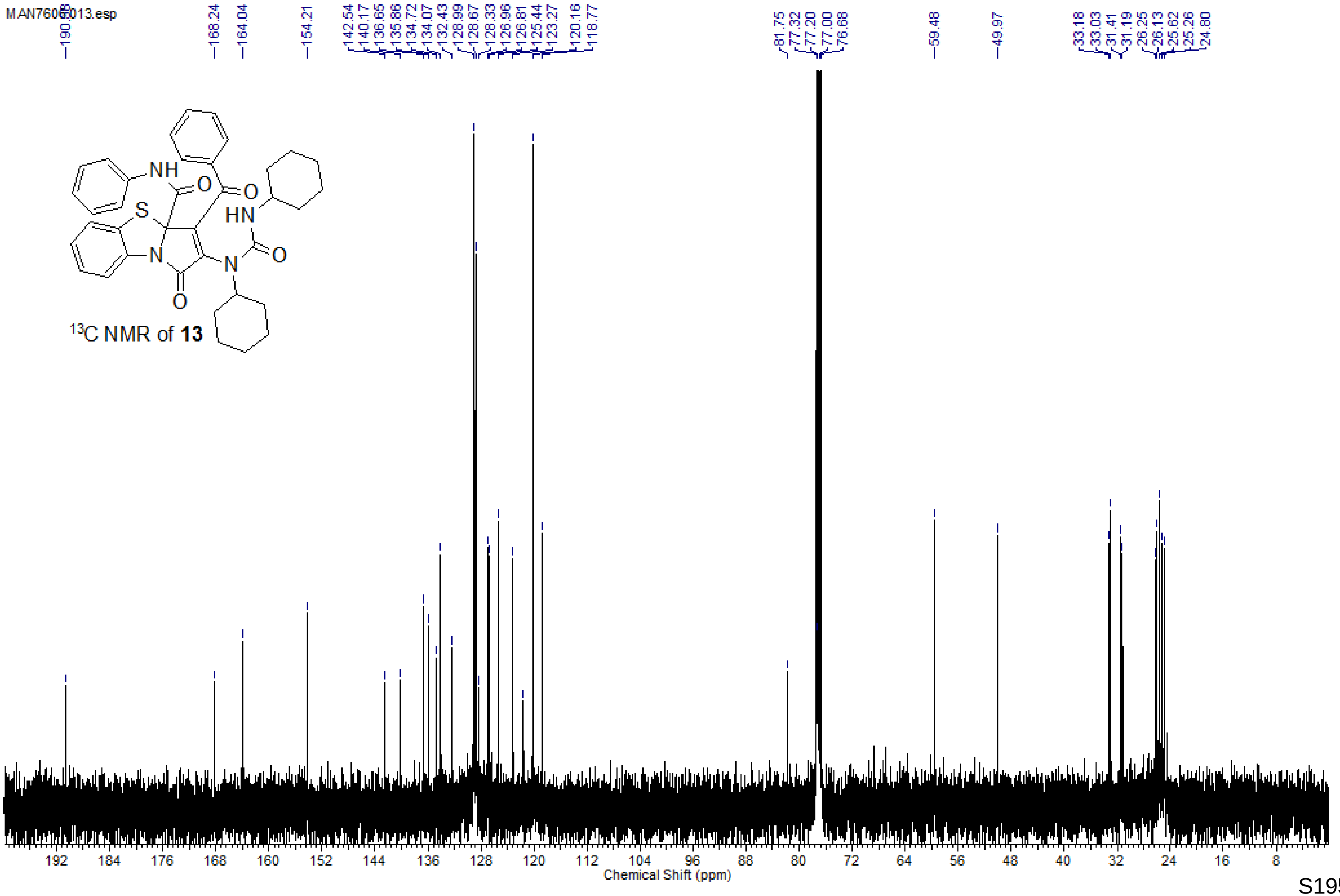
MAN760[illegible]013.esp
190.[illegible]
168.24
164.04
154.21
142.54
140.17
136.65
135.86
134.72
134.07
132.43
128.99
128.67
128.33
126.96
126.81
125.44
123.27
120.16
118.77
81.75
77.32
77.20
77.00
76.68
59.48
49.97
33.18
33.03
31.41
31.19
26.25
26.13
25.62
25.26
24.80
NH
O
S
N
O
HN
O
N
O
192 184 176 168 160 152 144 136 128 120 112 104 96 88 80 72 64 56 48 40 32 24 16 8
Chemical Shift (ppm)

^{13}C NMR of **13**

MAN7606.014.esp

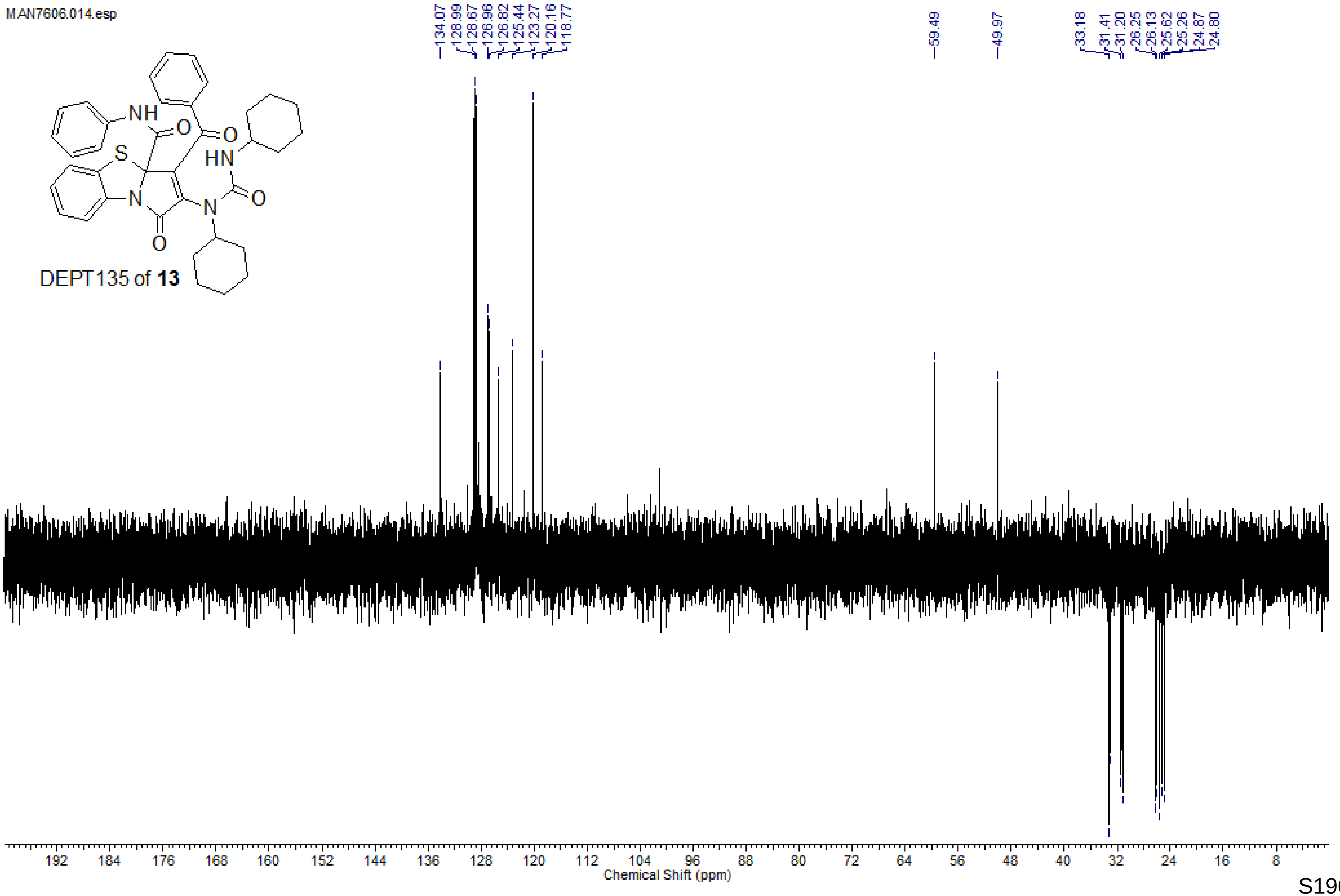

DEPT135 of **13**

Crystal structure determination

The unit cell parameters and the X-ray diffraction intensities were measured on a Xcalibur Ruby diffractometer. The empirical absorption correction was introduced by multi-scan method using SCALE3 ABSPACK algorithm [6]. Using the Olex2 [7], the structures were solved with the SHELXS [8] or SUPERFLIP [9] or SHELXT [10] programs and refined by the full-matrix least-squares method in the anisotropic approximation for all non-hydrogen atoms with the SHELXL program [11]. Hydrogen atoms bound to carbon were positioned geometrically and refined using a riding model. The hydrogen atoms of NH and OH groups were refined independently with isotropic displacement parameters.

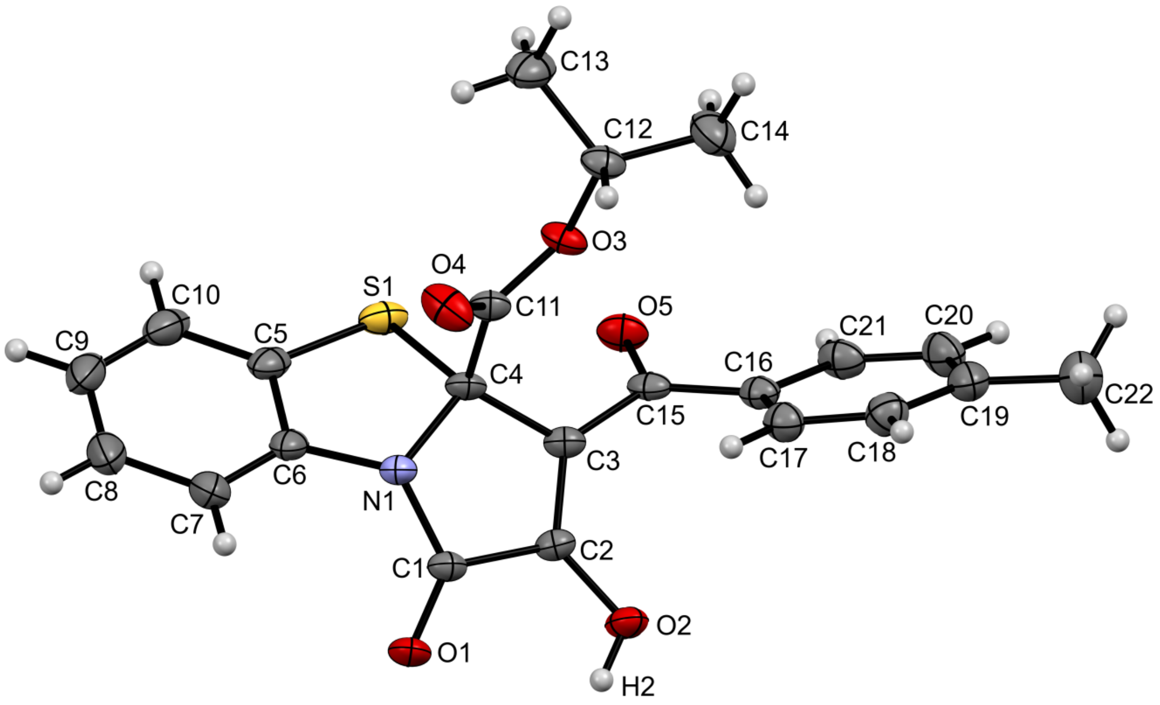

Figure S1. *Molecular structure of compound* **3bb** *showing 30% probability amplitude displacement ellipsoids (CCDC 2241415).*

Table S1. Crystal data and structure refinement for compound **3bb** (CCDC 2241415).

Empirical formula	$C_{22}H_{19}NO_5S$
Formula weight	409.44

Temperature, K	295.15
Crystal system	triclinic
Space group	P-1
a, Å	9.537(2)
b, Å	10.544(2)
c, Å	11.320(2)
α, °	77.678(18)
β, °	77.451(18)
γ, °	64.45(2)
Volume, $Å^3$	993.2(4)
Z	2
Density (calculated), g/cm^3	1.369
Absorption coefficient, mm^{-1}	0.197
F(000)	428.0
Crystal size, mm^3	0.45 × 0.22 × 0.1
Radiation	MoKα (λ = 0.71073)
2Θ range for data collection, °	6.076 to 58.958
Index ranges	$-11 \le h \le 12$, $-14 \le k \le 14$, $-15 \le l \le 12$
Reflections collected	8137
Independent reflections	4635 [R_{int} = 0.0415, R_{sigma} = 0.0658]
Data/restraints/parameters	4635/0/269
Goodness-of-fit on F^2	1.031
Final R indexes [I>=2σ (I)]	R_1 = 0.0567, wR_2 = 0.1359
Final R indexes [all data]	R_1 = 0.0871, wR_2 = 0.1668
Largest diff. peak/hole, $eÅ^{-3}$	0.37/-0.43

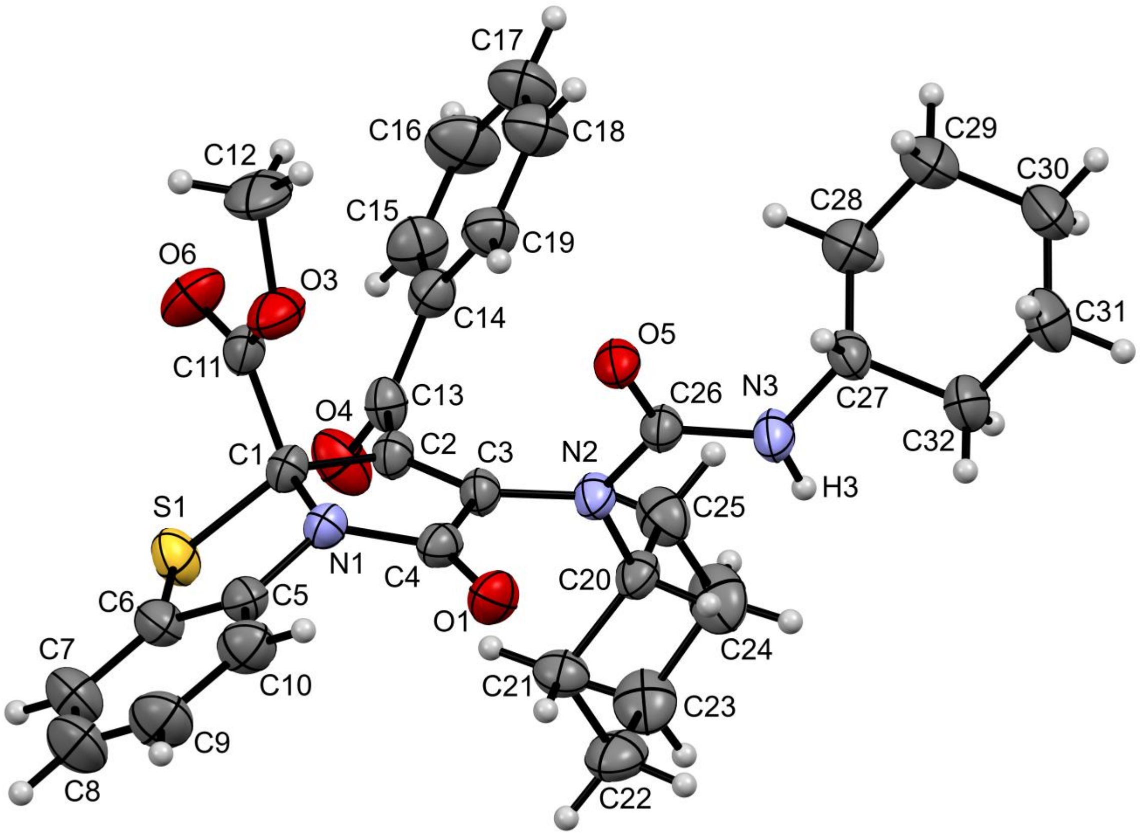

Figure S2. *Molecular structure of compound **4** showing 30% probability amplitude displacement ellipsoids (CCDC 2241420). Only one independent molecule is shown.*

Table S2. Crystal data and structure refinement for compound **4** (CCDC 2241420).

Empirical formula	$C_{32}H_{35}N_3O_5S$
Formula weight	573.69
Temperature, K	295(2)
Crystal system	monoclinic
Space group	$P2_1/c$

a, Å	21.418(3)
b, Å	10.8298(12)
c, Å	26.026(4)
α, °	90
β, °	97.009(14)
γ, °	90
Volume, $Å^3$	5991.6(15)
Z	8
Density (calculated), g/cm^3	1.272
Absorption coefficient, mm^{-1}	0.153
F(000)	2432.0
Crystal size, mm^3	0.48 × 0.26 × 0.03
Radiation	Mo Kα (λ = 0.71073)
2Θ range for data collection, °	3.884 to 59.078
Index ranges	-17 ≤ h ≤ 29, -11 ≤ k ≤ 14, -32 ≤ l ≤ 33
Reflections collected	37522
Independent reflections	14218 [R_{int} = 0.0662, R_{sigma} = 0.0881]
Data/restraints/parameters	14218/51/862
Goodness-of-fit on F^2	1.014
Final R indexes [I>=2σ (I)]	R_1 = 0.0719, wR_2 = 0.1690
Final R indexes [all data]	R_1 = 0.1724, wR_2 = 0.2375
Largest diff. peak/hole, $eÅ^{-3}$	0.27/-0.37

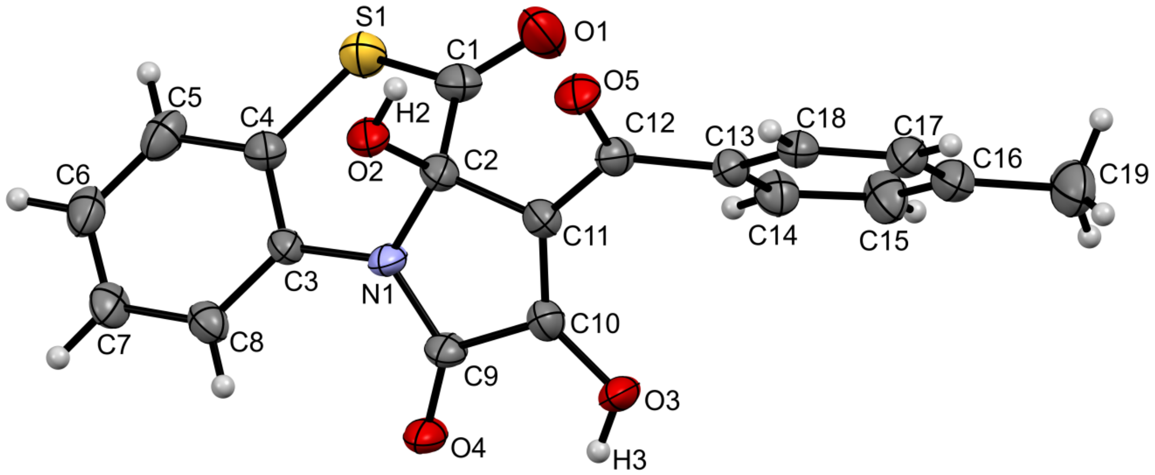

Figure S3. *Molecular structure of compound* ***6b*** *showing 30% probability amplitude displacement ellipsoids (CCDC 2241419). Toluene solvate molecule is not shown.*

Table S3. Crystal data and structure refinement for compound **6b** (CCDC 2241419).

Empirical formula	$C_{45}H_{34}N_2O_{10}S_2$
Formula weight	826.86
Temperature, K	295.15
Crystal system	monoclinic
Space group	$P2_1/c$
a, Å	13.007(5)
b, Å	7.100(3)
c, Å	21.848(7)
α, °	90
β, °	103.36(4)
γ, °	90
Volume, Å^3	1963.1(14)
Z	2

Density (calculated), g/cm^3	1.399
Absorption coefficient, mm^{-1}	0.200
F(000)	860.0
Crystal size, mm^3	0.2 × 0.15 × 0.1
Radiation	Mo Kα (λ = 0.71073)
2Θ range for data collection, °	5.546 to 58.886
Index ranges	-16 ≤ h ≤ 15, -9 ≤ k ≤ 8, -20 ≤ l ≤ 29
Reflections collected	9806
Independent reflections	4671 [R_{int} = 0.0963, R_{sigma} = 0.1465]
Data/restraints/parameters	4671/62/290
Goodness-of-fit on F^2	1.018
Final R indexes [I>=2σ (I)]	R_1 = 0.1048, wR_2 = 0.2618
Final R indexes [all data]	R_1 = 0.2127, wR_2 = 0.3520
Largest diff. peak/hole, eÅ^{-3}	0.44/-0.36

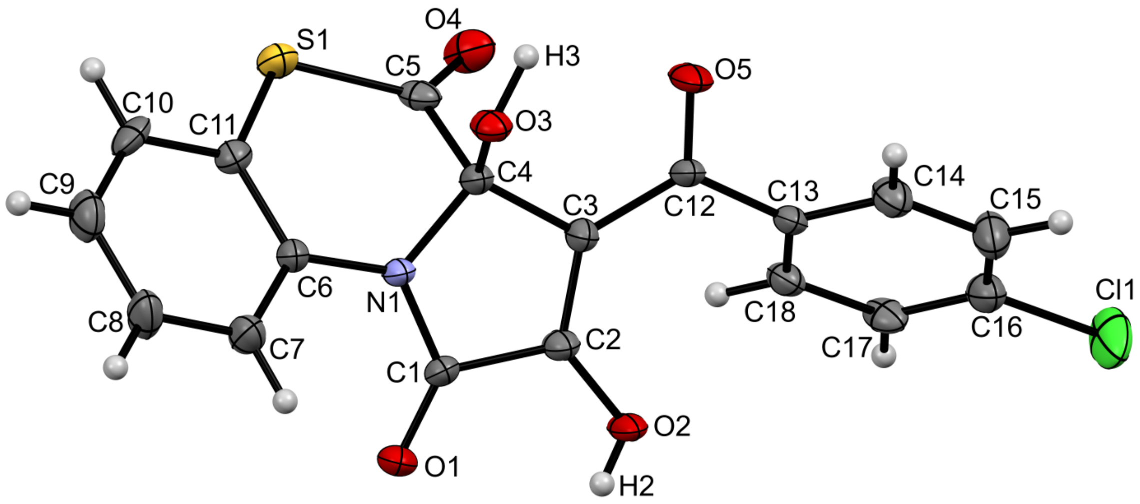

Figure S4. *Molecular structure of compound* ***6e*** *showing 30% probability amplitude displacement ellipsoids (CCDC 2241423).*

Table S4. Crystal data and structure refinement for compound ***6e*** (CCDC 2241423).

Empirical formula	$C_{18}H_{10}ClNO_5S$
Formula weight	387.78
Temperature, K	295.15
Crystal system	monoclinic
Space group	Cc
a, Å	15.639(5)
b, Å	15.684(7)
c, Å	6.932(2)
α, °	90
β, °	101.27(3)
γ, °	90
Volume, $Å^3$	1667.4(10)
Z	4
Density (calculated), g/cm^3	1.545
Absorption coefficient, mm^{-1}	0.385
F(000)	792.0
Crystal size, mm^3	0.5 × 0.04 × 0.03
Radiation	Mo Kα (λ = 0.71073)
2Θ range for data collection, °	6.596 to 58.576
Index ranges	-14 ≤ h ≤ 20, -21 ≤ k ≤ 19, -9 ≤ l ≤ 9
Reflections collected	3836
Independent reflections	2644 [R_{int} = 0.0403, R_{sigma} = 0.0668]
Data/restraints/parameters	2644/2/243
Goodness-of-fit on F^2	0.976
Final R indexes [I>=2σ (I)]	R_1 = 0.0536, wR_2 = 0.1360
Final R indexes [all data]	R_1 = 0.0717, wR_2 = 0.1618
Largest diff. peak/hole, $eÅ^{-3}$	0.26/-0.33

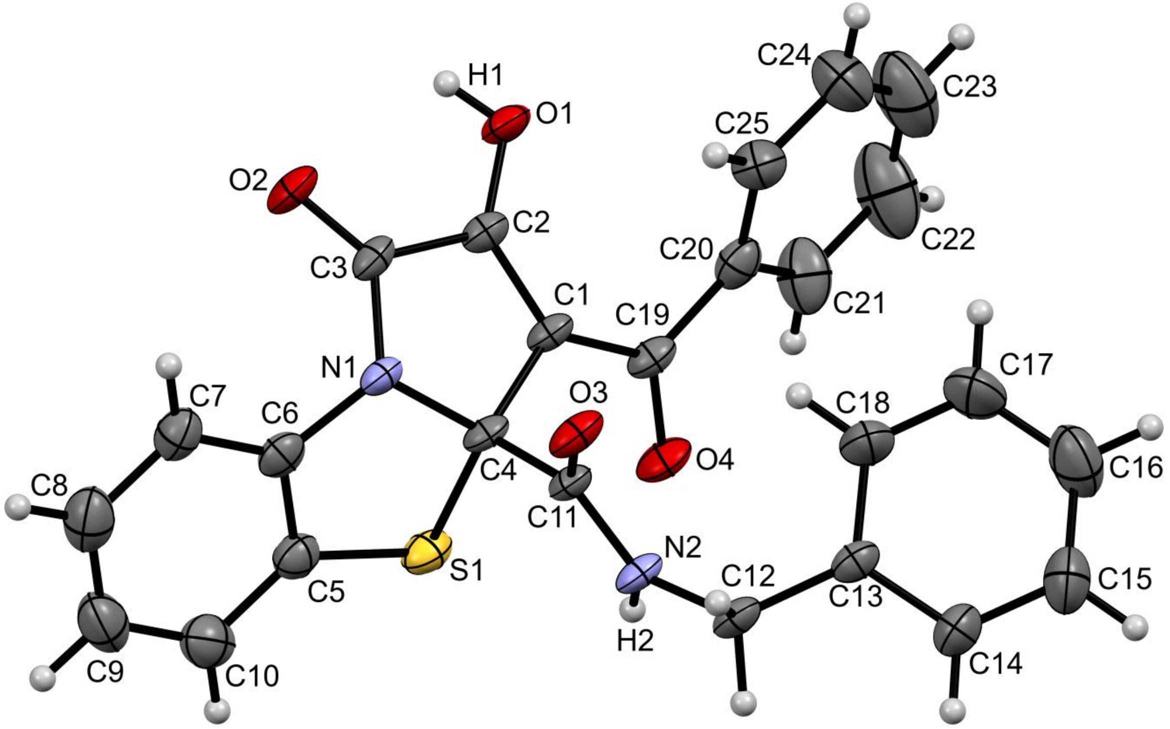

Figure S5. *Molecular structure of compound **7a** showing 30% probability amplitude displacement ellipsoids (CCDC 2241418). Only one independent molecule is shown.*

Table S5. Crystal data and structure refinement for compound **7a** (CCDC 2241418).

Empirical formula	$C_{25}H_{18}N_2O_4S$
Formula weight	442.47
Temperature, K	295.15
Crystal system	monoclinic
Space group	$P2_1/n$
a, Å	25.308(6)

b, Å	20.678(4)
c, Å	8.2996(14)
α, °	90
β, °	90.66(2)
γ, °	90
Volume, $Å^3$	4343.1(16)
Z	8
Density (calculated), g/cm^3	1.353
Absorption coefficient, mm^{-1}	0.184
F(000)	1840.0
Crystal size, mm^3	0.45 × 0.2 × 0.05
Radiation	Mo Kα (λ = 0.71073)
2Θ range for data collection, °	5.512 to 59.072
Index ranges	-32 ≤ h ≤ 23, -26 ≤ k ≤ 28, -11 ≤ l ≤ 11
Reflections collected	24909
Independent reflections	10334 [R_{int} = 0.0737, R_{sigma} = 0.1009]
Data/restraints/parameters	10334/0/593
Goodness-of-fit on F^2	1.044
Final R indexes [I>=2σ (I)]	R_1 = 0.0936, wR_2 = 0.2133
Final R indexes [all data]	R_1 = 0.1866, wR_2 = 0.2756
Largest diff. peak/hole, $eÅ^{-3}$	0.59/-0.36

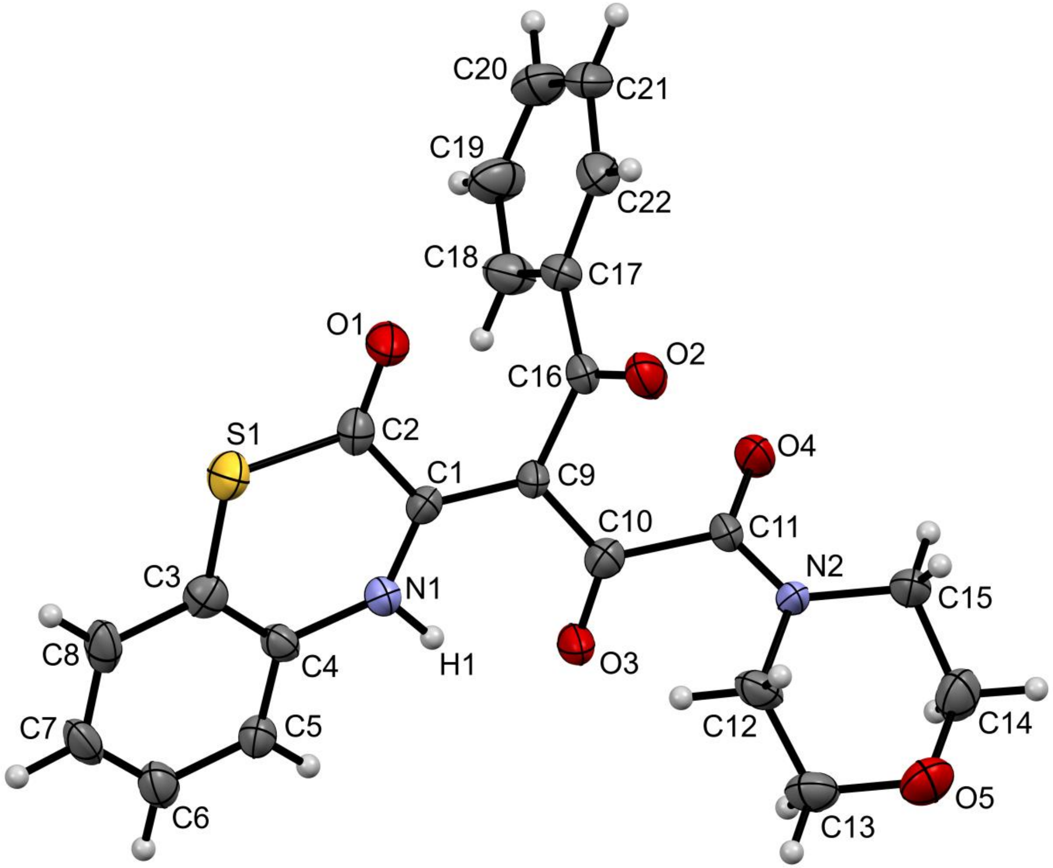

Figure S6. *Molecular structure of compound* ***10a*** *showing 30% probability amplitude displacement ellipsoids (CCDC 2241424).*

Table S6. Crystal data and structure refinement for compound **10a** (CCDC 2241424).

Empirical formula	$C_{22}H_{18}N_2O_5S$
Formula weight	422.44
Temperature, K	295.15
Crystal system	monoclinic
Space group	$P2_1/c$

a, Å	10.138(6)
b, Å	19.732(10)
c, Å	10.477(6)
α, °	90
β, °	110.91(7)
γ, °	90
Volume, $Å^3$	1958(2)
Z	4
Density (calculated), g/cm^3	1.433
Absorption coefficient, mm^{-1}	0.204
F(000)	880.0
Crystal size, mm^3	0.18 × 0.12 × 0.02
Radiation	Mo Kα (λ = 0.71073)
2Θ range for data collection, °	4.3 to 59.088
Index ranges	-13 ≤ h ≤ 7, -26 ≤ k ≤ 17, -9 ≤ l ≤ 14
Reflections collected	10529
Independent reflections	4689 [R_{int} = 0.1091, R_{sigma} = 0.2422]
Data/restraints/parameters	4689/0/274
Goodness-of-fit on F^2	1.003
Final R indexes [I>=2σ (I)]	R_1 = 0.0938, wR_2 = 0.1588
Final R indexes [all data]	R_1 = 0.2970, wR_2 = 0.2309
Largest diff. peak/hole, $eÅ^{-3}$	0.30/-0.29

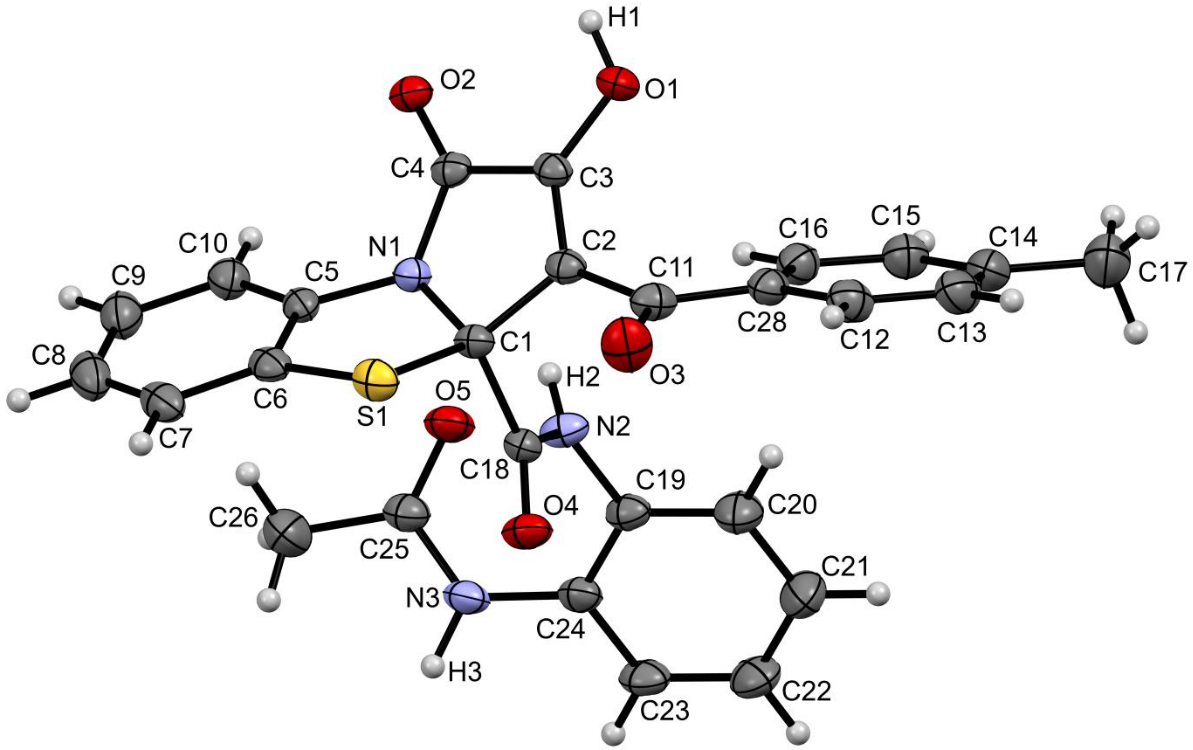

Figure S7. *Molecular structure of compound* ***12bd*** *showing 30% probability amplitude displacement ellipsoids (CCDC 2241422). Toluene solvate molecule is not shown.*

Table S7. Crystal data and structure refinement for compound **12bd** (CCDC 2241422).

Empirical formula	$C_{61}H_{50}N_6O_{10}S_2$
Formula weight	1091.19
Temperature, K	295.15
Crystal system	triclinic
Space group	P-1
a, Å	8.7224(14)
b, Å	12.811(2)

c, Å	13.709(2)
α, °	62.649(16)
β, °	80.597(13)
γ, °	77.287(14)
Volume, $Å^3$	1323.9(4)
Z	1
Density (calculated), g/cm^3	1.369
Absorption coefficient, mm^{-1}	0.169
F(000)	570.0
Crystal size, mm^3	0.4 × 0.08 × 0.06
Radiation	Mo Kα (λ = 0.71073)
2Θ range for data collection, °	4.8 to 58.982
Index ranges	-11 ≤ h ≤ 11, -15 ≤ k ≤ 16, -18 ≤ l ≤ 18
Reflections collected	12095
Independent reflections	6193 [R_{int} = 0.0432, R_{sigma} = 0.0725]
Data/restraints/parameters	6193/71/402
Goodness-of-fit on F^2	1.061
Final R indexes [I>=2σ (I)]	R_1 = 0.0661, wR_2 = 0.1525
Final R indexes [all data]	R_1 = 0.1112, wR_2 = 0.1859
Largest diff. peak/hole, $eÅ^{-3}$	0.30/-0.43

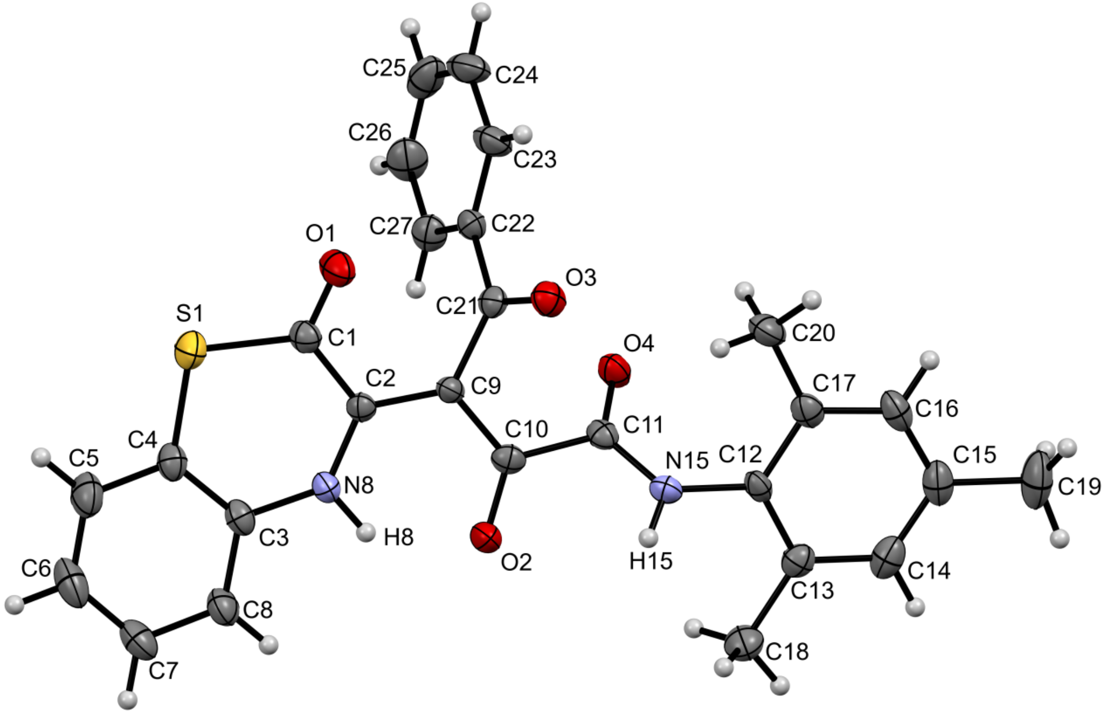

Figure S8. *Molecular structure of compound* ***14ab*** *showing 30% probability amplitude displacement ellipsoids (CCDC 2241416). Dichloromethane solvate molecule is not shown.*

Table S8. Crystal data and structure refinement for compound **14ab** (CCDC 2241416).

Empirical formula	$C_{28}H_{24}Cl_2N_2O_4S$
Formula weight	555.45
Temperature, K	295
Crystal system	triclinic
Space group	P-1
a, Å	10.927(2)
b, Å	10.962(2)
c, Å	12.606(2)

α, °	101.113(19)
β, °	95.903(16)
γ, °	115.32(2)
Volume, Å^3	1309.7(5)
Z	2
Density (calculated), g/cm^3	1.409
Absorption coefficient, mm^{-1}	0.366
F(000)	576.0
Crystal size, mm^3	0.35 × 0.25 × 0.1
Radiation	Mo Kα (λ = 0.71073)
2Θ range for data collection, °	4.218 to 59.14
Index ranges	-14 ≤ h ≤ 15, -14 ≤ k ≤ 14, -16 ≤ l ≤ 16
Reflections collected	9308
Independent reflections	9308 [R_{sigma} = 0.1393]
Data/restraints/parameters	9308/0/346
Goodness-of-fit on F^2	0.836
Final R indexes [I>=2σ (I)]	R_1 = 0.0619, wR_2 = 0.1364
Final R indexes [all data]	R_1 = 0.1564, wR_2 = 0.1550
Largest diff. peak/hole, eÅ^{-3}	0.24/-0.29
Refined twin ratio	0.5771(12) : 0.4229(12)

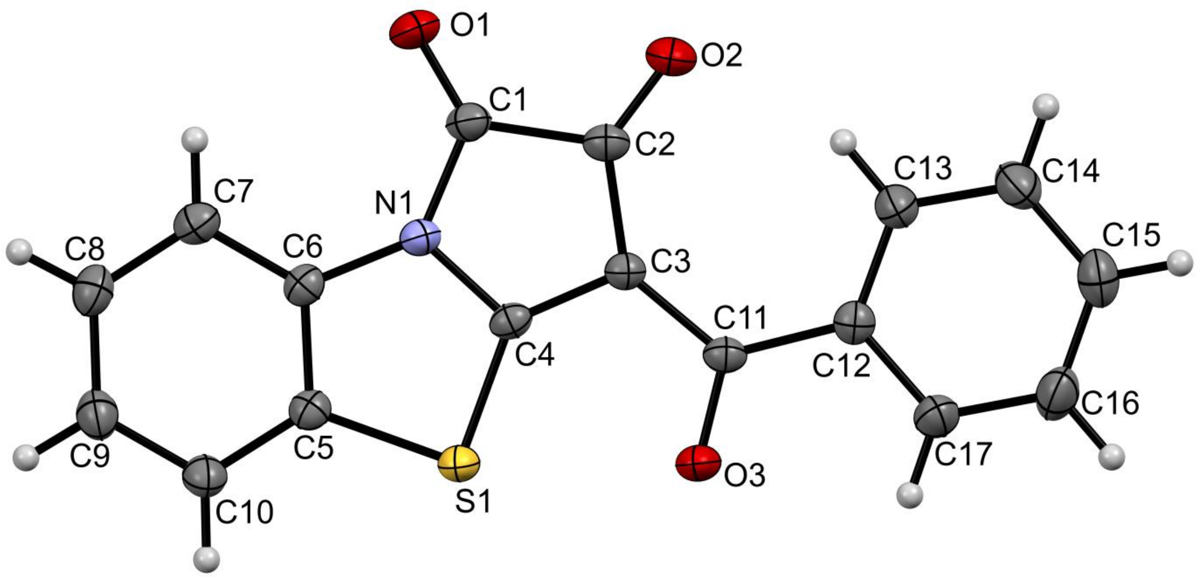

Figure S9. *Molecular structure of compound* ***17a*** *showing 30% probability amplitude displacement ellipsoids (CCDC 2241421).*

Table S9. Crystal data and structure refinement for compound **17a** (CCDC 2241421).

Empirical formula	$C_{17}H_9NO_3S$
Formula weight	307.31
Temperature, K	295.15
Crystal system	monoclinic
Space group	I2/c
a, Å	20.969(6)
b, Å	5.1385(14)
c, Å	25.282(6)
α, °	90
β, °	102.34(3)
γ, °	90
Volume, Å^3	2661.3(13)

Z	8
Density (calculated), g/cm^3	1.534
Absorption coefficient, mm^{-1}	0.256
F(000)	1264.0
Crystal size, mm^3	0.5 × 0.14 × 0.03
Radiation	Mo Kα (λ = 0.71073)
2Θ range for data collection, °	5.684 to 58.546
Index ranges	-19 ≤ h ≤ 28, -7 ≤ k ≤ 6, -33 ≤ l ≤ 34
Reflections collected	7468
Independent reflections	3158 [R_{int} = 0.0389, R_{sigma} = 0.0554]
Data/restraints/parameters	3158/0/199
Goodness-of-fit on F^2	1.037
Final R indexes [I>=2σ (I)]	R_1 = 0.0538, wR_2 = 0.1158
Final R indexes [all data]	R_1 = 0.0819, wR_2 = 0.1388
Largest diff. peak/hole, eÅ^{-3}	0.24/-0.43

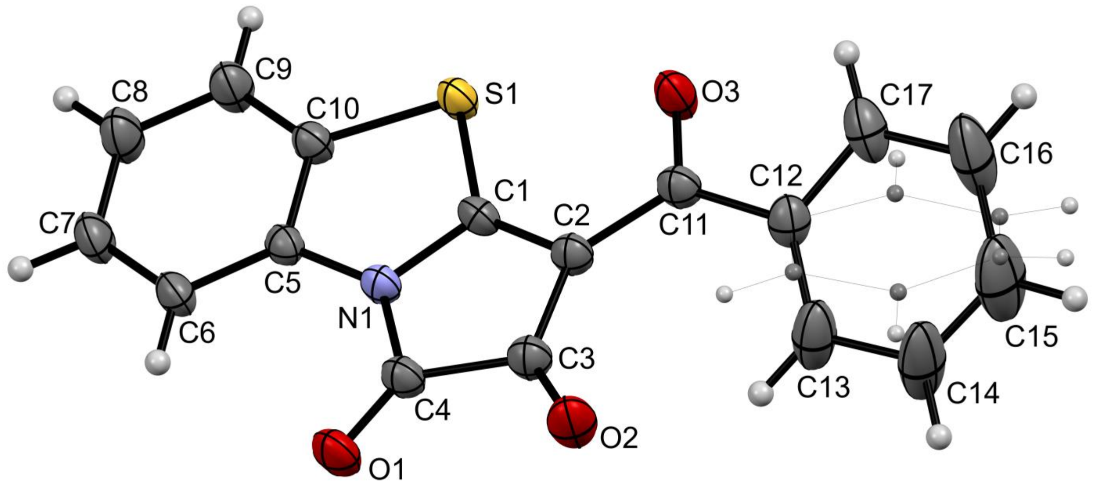

Figure S10. *Molecular structure of compound **17a** showing 30% probability amplitude displacement ellipsoids (CCDC 2241417).*

Table S10. Crystal data and structure refinement for compound **17a** (CCDC 2241417).

Empirical formula	$C_{17}H_9NO_3S$
Formula weight	307.31
Temperature, K	295.15
Crystal system	monoclinic
Space group	$P2_1/c$
a, Å	5.4696(15)
b, Å	26.985(8)
c, Å	9.099(3)
α, °	90
β, °	93.87(3)
γ, °	90
Volume, $Å^3$	1340.0(7)
Z	4
Density (calculated), g/cm^3	1.523
Absorption coefficient, mm^{-1}	0.254
F(000)	632.0
Crystal size, mm^3	0.3 × 0.1 × 0.03
Radiation	Mo Kα (λ = 0.71073)
2Θ range for data collection, °	6.04 to 58.478
Index ranges	-7 ≤ h ≤ 6, -36 ≤ k ≤ 20, -12 ≤ l ≤ 11
Reflections collected	6425
Independent reflections	3152 [R_{int} = 0.0544, R_{sigma} = 0.0820]
Data/restraints/parameters	3152/135/245
Goodness-of-fit on F^2	1.041
Final R indexes [I>=2σ (I)]	R_1 = 0.0683, wR_2 = 0.1592
Final R indexes [all data]	R_1 = 0.1262, wR_2 = 0.2119
Largest diff. peak/hole, $eÅ^{-3}$	0.32/-0.35

References

1. Khramtsova, E. E.; Lystsova E. A.; Dmitriev, M. V.; Maslivets, A. N.; Jasinski, R. *ChemistrySelect*, **2021**, *6*, 6295–6301. doi:10.1002/slct.202101990.
2. Stepanova, E. E.; Dmitriev, M. V., Maslivets, A. N. *Beilstein J. Org. Chem.,* **2020**, *16*, 2322–2331. doi:10.3762/bjoc.16.193.
3. Gumerova, D. F.; Mashevskaya, I. V.; Maslivets, A. N.; Kozlov, A. P. *Russ. J. Org. Chem*. **2003**, *39*, 995–997. doi:10.1023/B:RUJO.0000003192.96463.ca.
4. Mashevskaya, I. V.; Kol'tsova, S. V.; Maslivets, A. N. *Chem. Heterocycl. Compd.* **2000**, *36*, 1355–1356. doi:10.1023/A:1017500324352.
5. Khramtsova, E. E.; Lystsova, E. A.; Khokhlova, E. V.; Dmitriev, M. V.; Maslivets, A. N. *Molecules*, **2021**, *26*, 7179. doi:10.3390/molecules26237179.
6. CrysAlisPro, Agilent Technologies, Version 1.171.37.33 (release 27-03-2014 CrysAlis171 .NET).
7. Dolomanov, O. V.; Bourhis, L. J.; Gildea, R. J.; Howard, J. A. K.; Puschmann, H. *J. Appl. Cryst.*, **2009**, *42*, 339–341. doi:10.1107/S0021889808042726.
8. Sheldrick, G. M. *ActaCryst*., **2008**, *A64*, 112–122. doi:10.1107/S0108767307043930.
9. Palatinus, L.; Chapuis, G. *J. Appl. Cryst.,* **2007***, 40*, 786–790. doi:10.1107/S0021889807029238.
10. Sheldrick, G. M. *Acta Crystallogr., Sect. A: Found. Adv.*, **2015**, *71*, 3–8. doi:10.1107/S2053273314026370.
11. Sheldrick, G. M. *ActaCryst*., **2015**, *C71*, 3–8. doi:10.1107/S2053229614024218.
